# Supplementary material for: Impact of lifetime opioid exposure on arterial stiffness and vascular age: cross-sectional and longitudinal studies in men and women
Source: BMJ Open. 2014 Jun 2;4(6):e004521. doi: 10.1136/bmjopen-2013-004521 (PMC4054659; doi:10.1136/bmjopen-2013-004521)
Supplement: Author's manuscript [file bmjopen-2013-004521.draft_revisions.pdf]

Lifetime Opiate Exposure is an Interactive Cardiovascular Risk Factor for Vascular Ageing Related to Power Functions of Exposure Duration Particularly Marked in Females: Cross-sectional and Longitudinal Studies

Journal:	*BMJ Open*
Manuscript ID:	bmjopen-2013-004521
Article Type:	Research
Date Submitted by the Author:	21-Nov-2013
Complete List of Authors:	Reece, Albert; University of Western Australia, School of Psychiatry and Clinical Neurosciences
**Primary Subject Heading**:	Pharmacology and therapeutics
Secondary Subject Heading:	Cardiovascular medicine
Keywords:	arterial stiffness, opiate dependence, vascular ageing, biological age, acceleration of ageing, heroin

SCHOLARONE™ Manuscripts

For peer review only

Lifetime Opiate Exposure is an Interactive Cardiovascular Risk Factor for Vascular Ageing Related to Power Functions of Exposure Duration Particularly Marked in Females: Cross-sectional and Longitudinal Studies

Short Title:

Opiates as a Cardiovascular Risk Factor

Albert Stuart Reece

Gary Kenneth Hulse

School of Psychiatry and Clinical Neurosciences,

University of Western Australia.

Crawley WA 6009, Australia.

* **Correspondence to**:

Albert Stuart Reece

39 Gladstone Rd.,

Highgate Hill,

Brisbane, Queensland, Australia.

Ph: (617) 3844-4000

FAX: (617) 3844-4015

Email: sreece@bigpond.net.au

Word Count: 8,656.

Keywords – arterial stiffness, opiate dependence, vascular ageing, biological age, acceleration of ageing

For peer review only

Structured Abstract

Objective. To compare the potentiation of opiate exposure on vascular / biological ageing and arterial stiffness between males and females.

Design. Cross-sectional and longitudinal studies of 576 clinical controls and 687 opiate dependent patients (ODP) on 710 and 1,305 occasions over a total of 2,382 days (6.52 years), 2006-2011.

Setting. Primary care.

Participants. Controls: General practice patients with non-cardiovascular disorders, and university student controls. ODP: Patients undergoing clinical management of their opiate dependence. Controls had lower chronologic ages (CA) than ODP (29.96±0.45 vs. 34.55±0.32, mean±S.E.M., P<0.0001). Subjects were 69.62% and 67.69% male, and 15.97% and 92.29% were smokers (P<0.0001) for controls and ODP respectively. 86.32%, 10.33% and 3.35% of ODP were treated with buprenorphine (6.98±0.21mg), methadone (63.04±4.01mg) or implant naltrexone respectively. BMI was depressed in ODP.

Interventions. Radial Pulse Wave Analysis was undertaken with Atcor SphygmoCor software (Sydney) and Miller microtonometer.

Primary Outcome Measures - Vascular Reference Age (RA) and the ratio of the vascular age to the chronological age (RA/CA). Secondary outcome measures: Arterial Stiffness including Augmentation Index.

Results. After Body Mass Index -adjustment, RA in ODP was higher as a function of CA and of time (both P<0.05). Modelled ages in control and ODP males was 35.61 and 36.31 years, and in females 34.54 and 39.18 years. Changes in RA and major arterial stiffness indices were worse with CA in females (P<0.01). Quadratic, cubic and quartic functions of opiate exposure duration out-performed linear models with RA/CA over CA and over time.

For peer review only

The opiate dose-response relationship persisted longitudinally after multivariate adjustment from P=0.0013 in males and P=0.0073 in females.

Conclusion. Data show that lifetime opiate exposure is an interactive cardiovascular risk factor, particularly in females, is related to linear, quadratic, cubic and quartic functions of treatment duration and is consistent with other literature of accelerated ageing in OD patients.

For peer review only

Article Summary

Article Focus

- Despite striking consistency between several large epidemiological studies of the cardiovascular toxicity of log term opiate dependence, the subject is not well studied nor widely understood.
- There is only a single study of gender differences in significantly exposed populations known in the published literature.
- The implications of organism-wide xenobiotic-induced inflammation have not been incorporated into the clinical practice of addiction medicine, our understanding of the inflammatory basis of atherogenesis, or the fact that vascular ageing is a major surrogate for systemic ageing across the whole organism.

Key Messages

- Opiate dependent patients (ODP) had and advance of mean vascular age from control of 35.61 to 36.31 years in males and 34.54 to 39.18 years in females which, after adjustment for BMI, were significant in both males and females cross-sectionally and longitudinally (all $P<0.05$). Both the acceleration of vascular ageing and arterial stiffness were worse in females ($P<0.01$).
- A dose-response relationship between lifetime opiate exposure and measures of arterial stiffness and vascular age was demonstrated in both sexes, which was robust to multivariate adjustment for known cardiovascular risk factors.
- Use of quadratic, cubic and quartic functions of opiate duration exposure significantly improved the statistical fit of models of the ratio of vascular age (RA) to chronological age (CA, RA/CA ratio), thereby further highlighting the significance of the duration for which opiate agonism is administered.

For peer review only

Strengths and Limitations

- With 576 controls and 687 OPD the large size of this study was a signficant advantage, as was its combined cross-sectional and longitudinal design, its ability to control for many cardiovascular risk factors, the sex specific analysis, and the diverse output of cardiovascular parameters of the Atcor / SphygmoCor Pulse Wave Analysis technology employed.

- The use of advanced statistical modelling of relationships between factors, dose-response modelling, polynomial modelling in opiate exposure duration, inter-quartile trend effects, and multivariate adjustment added further analytical power to the study.

- As the major study limitation was its lack of other measures of organism ageing and its lack of mechanistic and ageing medicine-specific biomarkers, further studies incorporating these refinements are indicated.

For peer review only

Introduction

Opiate dependence (OD) is a condition of increasing public health importance.

OD is associated with an impressive array of traditional and novel cardiovascular risk factors and associations including hypertension [1], hyperlipidaemia [2-3], tobacco consumption, increased body weight [4], hyperglycaemia [3] , hyperfibrinogenaemia [3], diffuse polyclonal gammopathy and immune stimulation [5-6], elevated high sensitivity CRP [7], elevated cytokines [8], relative hypercalcaemia and hyperphosphataemia [9] and reduced circulating endothelial progenitor cells [10]. Furthermore OD has been associated with atherosclerotic disease in both the coronary [11-18] and cerebral [14 18] circulations. In a 33-year review of an opioid maintenance program in California in 2004, death from cardiovascular disease was noted to account for three times as many years of life lost in the treatment group than in the general population [18]. In 2007 an Iranian surgical group identified that opium dependent patients were at increased risk of requiring open coronary revascularization with an odds ratio of 1.8 after adjustment for other risk factors [15]. Moreover in this series there was a dose-response relationship between the amount of opiates consumed and the severity of the coronary disease. It was also shown that the coronary disease occurred two years earlier in OD persons than in controls [16]. This group further identified that opium use was the strongest cardiovascular risk factor in their population of men, with effects greater than traditional risk factors [17]. The sex specific finding in this study was felt to be related only to a reduced prevalence of opium consumption amongst females.

In reviewing over 800 fatal cases of opiate toxicity in Australia in 2006 Darke et al. found that 17% of decedents over the age of 44 years had coronary stenoses in excess of 75% [12]. Similarly when comparing 1193 post mortem cases of heroin and methadone toxicity the Sydney group found 2-3 times elevated adjusted odds ratios of ventricular hypertrophy, and myocardial, interstitial and perivascular fibrosis in the methadone treated group, consistent with the effects of more consistent opiate agonism in such patients [11]. In a 21-year follow-up of 42,676 patients undergoing maintenance opioid agonist treatment in Australia, including 425,998 person-years in 2009, Degenhardt et al. identified cardiovascular death as occurring 2.2 times as often as in the wider community [13]. Indeed in this study elevated rates of death

For peer review only

with severe organ dysfunction were noted which appeared to act as a backdrop to the very high rates of death by overdose generally reported in such cohorts (see Web Appendix 6 of this citation).

In 2012, a prospective population based study of 50,045 people including 234,098 person-years reported hazard ratios of death from coronary artery disease (CAD) of 1.9-fold (95%C.I. 1.57-2.29) and of cerebrovascular disease (CeVD) of 1.68-fold (C.I. 1.29-2.18) [14]. Both were worse in females (CAD: male adjusted HR (AHR) 1.58, female AHR 2.90; CeVD male AHR 1.49, female AHR 1.97). Furthermore recent reports of 1263 patients found elevated rates of vascular age in patients treated with methadone compared to other agents [19] and improved vascular ages accompanying the long term drug-free condition [20].

Moreover opiate patients dependent on short acting opiates such as morphine, oxycodone and heroin undergo the clinical syndrome of opiate withdrawal several times daily. This syndrome is a hyper-adrenergic state characterized by neurological and cardiovascular stimulation which in its more severe manifestations includes hypertension and tachycardia [21] and a reduction of subendocardial perfusion [22]. This negative aversive experience is one of the key reasons many opiate dependent patients (ODP) continue to use narcotics.

Important as the above collected findings are, other evidence suggests that the impact of elevated vascular age has implications on the health and ageing of the organism as a whole beyond those limited primarily to the cardiovascular system. It has been repeatedly proposed that vascular ageing is a principal component of biological organismal ageing, by virtue of the contribution made by cardiovascular causes to the overall death rate in developed nations, and also the importance of the vascular niche common to many stem cell beds [23-24]. This view closely accords with the finding in many of the above studies and particularly that by Degenhardt et al [13] that elevated rates of overdose occur on the background of advanced disease in other organs [1 12 18]. Opiate dependent patients have many stigmata of accelerated ageing including increased rates of: hair greying, erosion of alveolar bone from chronic periodontitis [25], osteoporosis [26], chronic respiratory disease [11 13 18], chronic renal disease [11], diffuse senescent-mimetic immune stimulation [5-6 27], diabetes [3], neuropsychiatric disorders, multi-system disease [11-12], reduced circulating stem cell counts [10], and many malignancies [13 28]. Thus the call has been made for geriatricians to be appointed to the care of opiate

For peer review only

dependent patients beyond the age of 50 years, due to the high prevalence of multi-system disease [1].

The exciting possibility therefore arises that an improved understanding of the increased and accelerated profile of degenerative disease seen in opiate dependent patients may enhance our understanding, and in time our management, of age related disorders in the rest of the community.

As this clinic sees both opiate ODP and the general community, we were ideally situated to compare these two groups. The availability of the mobile SphygmoCor system from Atcor in Sydney implies that a host of central cardiovascular data is available to the clinician in a non-invasive and rapid manner, providing quantitation of important subclinical endophenotypes of central vascular stiffness and cardiovascular and therefore organismal ageing. The central investigative question to be prospectively assessed was whether there were changes in major central cardiovascular parameters in the opiate dependent group, and if so are they robust to multivariate adjustment for other confounding factors. Several earlier papers from our group have considered this issue in both cross-sectional [29-30] and longitudinal [31-32] studies in each sex separately and drawn conclusions generally confirmatory of this hypothesis. It has been shown that the type of opioid pharmacotherapy affects the severity of opiate related potentiation of arterial stiffness [19], and that induction of the drug free state after implantable opiate antagonists reverses this finding [20]. However a formal comparison of the effects by gender has not been possible in these earlier reports. Gender comparison therefore forms the major focus of the present report which is a compilation of much of this earlier data.

For peer review only

Methods

Patient Selection. Control subjects (N=576) were recruited opportunistically from patients presenting for health examination checks for insurance or employment medical examinations or from patients presenting with minor health problems such as ear blockages, minor psychological disorders, or University students. Patients with known cardiovascular disorders such as hypertension, renal disease or diabetes or diseases known to perturb the CVS subacutely such as infections or pregnancy, were excluded. Opiate dependent patients (N=687) were also recruited opportunistically and were under treatment with either methadone (N=71), buprenorphine (N=593) maintenance, or naltrexone implant (N=23). All were sampled opportunistically at the time of their clinic visits May 2006 - December 2012.

Patient Treatment. Opiate dependent patients maintained on, methadone or buprenorphine were managed by accepted clinical algorithms by their usual health providers for their drug dependency. The naltrexone implants used for the management of heroin dependence were manufactured by "Go Medical" industries in Perth, Western Australia under Commonwealth of Australia Therapeutic Goods Administration (TGA) GMP conditions and administered under the compassionate Access arrangements of the Special Access Scheme authorized by the TGA as described elsewhere [33]. Patients were recalled for re-testing at two and five years post baseline assessment. Any patient with a known chronic or subacute cardiovascular condition were excluded from participation. Frequency and quantity of substance use by substance type (inclusive heroin) was assessed at the initial time and subsequently. Other opiates were converted into morphine equivalents, and then into heroin equivalents at the rate of 1g of street heroin = 0.5g morphine. The duration of opiate use was counted from the time of first use. If it became known that alcohol had been consumed in the few hours prior to the test or that stimulants had been used in the three days prior, studies were excluded.

PWA Studies. Patients were positioned supine for the performance of the test and allowed to rest for five minutes prior to the test. Consumption of food, drink, alcohol or tobacco was not restricted, but patients were not allowed either to sleep or talk during the testing procedure. Pulse wave analysis by applanation tonometry was performed on the right radial artery unless it was unavailable. The brachial blood pressure was taken in the opposite arm using the

For peer review only

oscillometric Omron HEM 907 device. The data was collected and analyzed by the SphygmoCor software. Patients were studied in quintuplicate, and adequate studies were averaged for that day. Acceptable studies had an Operator index of greater than 70%, and were not inconclusive. Details of patients' prior drug use was also taken at the time of the study performance and entered into the database. Time in days was measured from the time of the first PWA study.

The SphygmoCor software used for the PWA studies calculates a number of major indices including Vascular Reference Age (RA), Chronologic Age (CA), Central Augmentation Pressure at Heart Rate 75 (C_AP_HR75) the Central Augmentation Pressure / Pulse Height Ratio at Heart Rate 75 (C_AGPH_HR75) also known as the Augmentation Index, Central Pulse Height (C_PH), Central Augmentation Load (C_AL), Central Augmentation Time Index (C_ATI), Peripheral–Central Pulse Pressure Amplification Ratio (PPAmpRatio), Maximal Peripheral Rise of dP/dT (P_MAX_DPDT), Central Systolic Pressure (C_SP), Central Diastolic Pressure (C_DP), Central End Systolic Pressure (C_ESP), Central Mean Pressure (C_MEANP), the Central Diastolic Time Index (C_DTI), the Central Tension Time index (C_TTI), and an index of subendocardial perfusion known variously as the Subendocardial Perfusion Ratio (SEVR), the Central Stroke Volume Index (C_SVI) or the Buckberg ratio defined as the C_TTI / C_DTI.

Statistics. Data are presented as the mean ± S.E.M. Categorical data were studied using "EpiInfo" 7.0.8.3 using the Chi squared test obtained from the CDC Atlanta, Georgia or the Fisher Exact test if the numbers in a cell were less than 20. Continuous categorized data were analyzed in "Statistica" 7.1 from Statsoft, Oklahoma using Student's t-tests. T-tests with separate variances were used where Levene's test was significant. Continuous variables such as RA, CA, RA/CA, variously corrected RA, SP, Weight, HDL, BMI, and systolic pressure were log transformed in accordance with normality assumptions based on the Shapiro-Wilks test. High sensitivity CRP and the heroin dose were arcsinh transformed which is a similar transform to logarithmic, but accepts arguments of zero. Time (as days) was measured from the time of the initial PWA study. The time since the last cigarette was recorded and analyzed as a factor with cut-off points at 30, 60, 120, 180 and > 180minutes, and a non-smoking category. For some analyses lifetime heroin exposure was cut into quartile

For peer review only

categories with divisions at 0, 0.01, 2.49, 6.59 and 350 g-years respectively, yielding 583, 207, 226 and 227 patients in each group respectively. Multiple regression was performed in "R" 2.15.2 obtained from the University of Melbourne Central "R" Archive Network mirror. Mean model estimates for various groups were prepared by inserting the mean parameter values into the untransformed final models. Multiple linear regression of truncated variables such as RA (minimum value of 20, maximum value of >80) were analyzed using tobit multiple regression techniques from the Applied Econometrics library in "R" (AER). RA's of individual studies returned as >80 were assigned as 88. Random effects were assigned to unity and the patient identification code. Time-dependent repeated measures analysis was performed using non-linear mixed effects (nlme) regression module in "R" using restricted equation maximum likelihood (REML) techniques. When nlme models were compared they were converted to maximum likelihood (ML) methods to allow Anova testing. In each case model reduction was performed manually by the classical method involving removing the least significant term. The log-likelihood value was abbreviated to LogLik. Graphs were prepared with reshape2 and ggplot2. T-tests were two-sided. $P<0.05$ was considered significant.

Ethical Approval. All patients provided their informed consent to participate in the study and for their medical treatment. The study was approved by the Human Research Committee of the Southcity Family Medical Centre, which is registered with the National Health and Medical Research Council.

For peer review only

Results

576 control patients were compared with 687 opiate dependent patients. The mean ages were 29.96±0.45 and 34.55±0.32 (mean ± S.E.M.) years (t=-8.262, dF=1082.22, P<0.0001, Table 1.), with 401 (69.62%) and 465(67.69%) males respectively (Chi^2 = 0.54, P=0.46). 15.97% and 92.29% smoked tobacco (Chi^2=746.56, P<0.0001). The mean CA of the whole group was 32.45±0.27years. The mean CA of the males was 32.14±0.33 and of females 33.16±0.53 years. Racial stratification was not performed as our population is around 90% Caucasian in background. Other baseline biometric, drug use and laboratory values are given in Supplementary Table 1. Patients were studied longitudinally on 710 and 1,305 occasions. The period of repeat study, measured from the time of the first PWA study, was 624.11 ± 18.74 days (range 1-2382 days; 495 (60.36%) studies performed beyond 360 days).

Comparative PWA values are given in Supplementary Table 2. Most augmentation values and the C_SP and C_ESP are noted to be higher in the OD group consistent with their being an average of 4.59 years older.

Figure 1 shows different vascular age and augmentation parameters corrected for various features, against CA. This figure shows that the BMI was lower in the OD group than the controls both as a factor (factor est.=0.0719, t=2.197, P=0.0282) and in interaction with CA (BMI: CA est.=-0.0023, t=-2.350, P=0.0189; model F=34.94, dF=3, 1258, P<2.2E-16). In the longitudinal series BMI was lower as a factor (est.=-0.0174, dF=812, P=0.0470; model AIC= -3722.55, LogLik=1867.27). This result may be the effect of cumulative diversion of resources over time. Figure 2 shows similar indices over time. Supplementary Table 3 presents the results of the cross-sectional data where BMI corrected RA is regressed against CA and addictive status. In the whole group both OD status (P=0.0265) and the CA: addiction interaction (P=0.0205) are significant. Similar results were found in each sex. Similar results were also found in the longitudinal study when the RA/CA ratio was regressed against CA, time and dependency status both for OD as a factor (P=0.0102) and in interaction with CA (P=0.0028; details in Supplementary Table 4).

Inclusion of tobacco exposure along with opiate exposure in these models did not alter these findings (data not shown).

For peer review only

As shown in Figures 3 and 4, these changes were found to be more pronounced with CA in females. These data are presented corrected for the changes in BMI in Figures 5 and 6. Details for these significant differences are presented in Table 1.

Figure 7 shows these data by sex with loess curves fitted (span=0.95). This strongly suggests that an inflection point is reached about the age of 30 in females, which is not seen in males until about 40 years of age. This was quantified by dichotomizing CA from age 41 in males and 29 in females. As shown in Table 2 in males in the older age category, the addictive status and their interaction were significant (P= 0.0010, 0.0021 and 0.0008 respectively), whilst in females <55 years the older age: addiction interaction was significant (P=0.0195).

The regression changes are quantified for some of the major indices of arterial stiffness in cross-sectional data in Supplementary Table 5 (all P<0.005). Similar significantly different sex specific data were found in the longitudinal dataset when the RA was regressed against CA, time, sex and addictive status (most P<0.02, Supplementary Table 6).

When the mean sex-specific values for CA and BMI are inserted into the equation of Table 3 the mean ages of the control groups amongst all patients, males and females was 35.42, 35.61 and 34.45 years, whilst the corresponding ages of the ODP's was 37.24, 36.31 and 39.18 years, representing incremental gains of 5.14%, 1.97% and 13.43%.

When quartiles are defined for lifetime heroin exposure the results shown in Figure 8 are found. Similar effects longitudinally are shown in Supplementary Figure 1. These results are quantitated in Supplementary Table 7 for both cross-sectional and longitudinal data compared to non-exposed controls. In the cross-sectional analysis the RA/CA ratio was regressed against the CA and Opiate exposure quartile. In the longitudinal analysis the RA/CA ratio was regressed against time, CA and the opiate exposure quartile. Whilst the CA: Level 3 opiate exposure interaction is of borderline significance in all patients and females (P=0.0546 and 0.0568, but not in males) in the longitudinal study the age: level 2 exposure is significant in all patients and males (P=0.0003 and 0.0110) and level 3 opiate exposure approaches significance in females both as a factor and in interaction with age (P=0.0501 and 0.0547). Supplementary Table 8 presents the results of a longitudinal regression of age-corrected

For peer review only

indices of cardiovascular stiffness against time and opiate exposure quartile by gender, and notes a highly significant effect by exposure quartile in both genders.

The data was formally interrogated for a trend between the different quartiles of lifetime opiate exposure using the contrasts polynomial function in "R". Significant contrasts between the various quartiles for lifetime opiate exposure for the age: opiate exposure interaction was indeed demonstrated in all patients (est. = 0.1657, S.E. = 0.0787, t = 2.104, P = 0.0356; Model Adj. R^2 = 0.4265, F = 133.0, dF = 7, 1235, P < 2.2×10^{-16}), such that moving up a quartile level at age 60 increased the modelled RA by 8.42% to 65.051 years, and movement from the first (non-exposed) quartile to the highest (level 3) quartile increased the modelled RA 25.26% to 75.154 years.

Some environmental exposure – pathology relationships have been shown to be power functions of the duration of exposure and are therefore particularly sensitive to the periods for which exposure occurs [34]. Since opiate administration is often prescribed and recommended as being of indefinite duration this is particularly relevant to the present discussion. Therefore a detailed study of the power relationships of the duration of opiate administration was undertaken. Table 3 shows that simple regression models of RA/CA against the dose-duration interaction were significantly related to linear, quadratic, cubic and quartic terms in opiate exposure duration. Moreover when RA/CA was regressed against a model including interactive terms in linear, quadratic, cubic and quartic terms, all four were significant. When these models were compared by ANOVA studies the quadratic (AIC=82.888) was noted to be significantly better than the linear, cubic or quartic models, but not significantly different from the combined model (AIC=83.731, P=0.87; Supplementary Table 9).

When a similar exercise was performed in the longitudinal dataset, similar results were obtained (Supplementary Table 10). In this study RA/CA was regressed against time and the opiate exposure quartile. As a combined linear-quadratic-cubic-quartic model failed to converge, the highest order model used was linear-quadratic-quartic. Again quadratic and quartic terms in exposure duration persist in the final model. In this case the quadratic model (AIC=1040.85) was significantly superior to all the others but was not significantly better than the linear-quadratic-quartic model (AIC= 1041.09, P=0.082; Supplementary Table 11).

For peer review only

The data was interrogated for the presence of a dose-response relationship directly by examining continuous terms in opiate dose and duration regressed against the RA/CA ratio in both datasets. As shown in Table 4 the dose-duration interaction is significant in all patients considered together, in males and in females (P=0.0.000037, 0.00029 and 0.0236) in the cross-sectional dataset, whilst in the longitudinal dataset the parallel results are P = 0.0004, 0.0008 and 0.0127.

The final step of the analysis was to consider whether the significance of the opiate dose and duration might persist after multivariate adjustment for all known cardiovascular risk factors. In preliminary analyses lipidic factors were invariably eliminated during the model reduction process. Moreover, as discussed in the Introduction, opiates themselves are likely to perturb both lipid function, and also have a robust immunostimulatory action. Therefore including immune markers is likely to over-correct the model. Similarly when the time since the last cigarette consumption was included in the cross-sectional and longitudinal models it was eliminated during model reduction. For these reasons the final cross-sectional formula used regressed RA/CA against interactive terms in opiate dose and duration, height, tobacco consumption, systolic blood pressure and weight. The longitudinal model was similar but had an additive term in weight.

The results shown in Table 5 were found for the cross-sectional data. This table shows that significant interactions including opiate dose-duration terms are found in all patients (P=0.0261) and in males (P=0.0017; Adj. R^2=0.0815), whilst in females this interaction approaches significance (P=0.0516). When a similar exercise is performed on the longitudinal data, significant interactions are seen from P = 0.0011, 0.0013 and 0.0073 and above in each group respectively (Table 6).

Supplementary Table 12 presents the results of final models in which complete multiple regression was performed in the cross-sectional dataset for all pertinent variables. RA/CA was regressed against interactive terms in opiate dose and duration, height, tobacco use, systolic pressure and high sensitivity CRP together with additive terms in weight, cholesterol and HDL. Opiate dose and duration persist in the final model in males and in all patients, but not in females.

For peer review only

Discussion

The major findings of this study are that indices of vascular age and arterial stiffness are worse in opiate dependence compare to opiate naïve controls with mean calculated ages elevated by 1.97% in males and 13.43% in females. The effect was thus more marked in females. A significant effect was found on both vascular age and augmentation index by exposure quartile after correction for CA and BMI. The RA/CA ratio was found to be related to power functions of the opiate duration of exposure in both cross-sectional and longitudinal analyses. A dose-response relationship was demonstrated with lifetime opiate exposure. And the effect of opiate exposure was robust, and remained after multivariate adjustment in cross-sectional and longitudinal studies.

These findings should be interpreted in the light of the relatively modest degree of opiate exposure to which these patients were exposed. Whilst the dose and duration of opiates used by patients in this study is typical of that seen in many published series, the treatment of these patients was mostly with the relatively mild partial agonist buprenorphine, with only modest doses employed (mean 6.98±0.21mg): this dose level being much lower than the commonly recommended (18-24mg) [35]. Moreover buprenorphine has been shown to be more mild in its cardiovascular effects than the full agonist agent methadone . For this reason the results reported herein should be seen as a lower bound on the effects which may be observed.

The findings in relation to central vascular stiffness and age being related to power functions of the treatment duration is of great importance. Chronic opiate agonist treatment is often seen as relatively benign and partly for this reason is frequently recommended for very long term or indefinite administration [36-37]. However the demonstration here that the effects likely compound with time have major clinical practice implications, and have not been previously noted. The 1.97% effect described here for males, compounded over 30 years equates cumulatively to 179.5% gain and in diminution to 44.95% loss. Similarly the 13.43% gain seen in females compounded over 30 years is a 4349.02% gain and a 98.66% loss in reduction. The effects therefore of these process maintained over the long term on stem cell diminution or immune stimulation are potentially very considerable.

The exacerbation of the effect in females was clearly demonstrated in Figures 3-7, and Supplementary Tables 4 and 5. This finding has, to the authors knowledge, only been

For peer review only

reported previously by an Iranian group [14]. If one looks closely at Figure 7 several fascinating findings emerge. The vascular age algorithm used by the SphygmoCor software is based upon the augmentation index C_AGPH_HR75. This index has a clear curvilinear convex upward relationship to CA as shown in Figure 7 in the lower panels, unlike the C_AP_HR75 which is directly linear [38]. Hence the reduction at the higher ages on the C_AGPH_HR75 curve may be somewhat artefactual. If one looks at the upper panels of Figure 7 the rise in RA with age in ODP may be seen to be broadly exponential in form, if the decline in slope above age 45 years is discounted. In females it appears that the rise in RA occurs some 10 years prior to that seen in males, a finding emphasized by the middle panels. This fits with a dramatic clinical transformation which is seen in OD females at around age 30, and in males at about age 40. Whilst the cause of this is unknown it may possibly be related to a more aggressive immune system in females which is in part hormonally mediated [39].

As noted above, vascular age is frequently said to be a surrogate for organismal ageing. The present data are consistent with that view, and also with the literature cited of accelerated ageing in these patients. The present technique provides a mechanism to quantify these changes at the whole human level.

As no structural imaging techniques were performed it is not possible to say with certainty if the observed elevations in vascular stiffness were associated with fixed atherosclerotic lesions. However a large and weighty literature confirms the relevance of significance of elevations in vascular stiffness [40-44]. Structural correlation must await further work.

Some of the most interesting questions to emerge from the present work relate to the possible mechanisms underlying the plethora of changes which have been identified in OD as cardiovascular risk factors, as potentially atherogenic processes and as accelerants of ageing processes. Opiates have been linked with blockade of the cell cycle at cyclin dependent kinases 2, 4 and 6 by $P16^{INK4A}$ and $P21^{WAF1/CIP1}$ [45-46]. P16 is the gene product of CDKN2A located at the senescence locus on Chromosome 9p21.3 which is a hotspot for many GWAS studies, and from which three senescence related proteins and one senescence associated long non-coding RNA are transcribed [47]. Furthermore opiate agonists have been shown to directly ligate the myeloid differentiation factor 2 (MD2)- toll-like receptor (TLR) 4 heterodimer leading to the downstream activation of the numerous powerful pathways linked to

For peer review only

stimulation of the endotoxin receptor [48]. Indeed systemic endotoxaemia was recently shown to modulate the expression of 6,025 genes [49]. Some of the pathways activated downstream by TLR4 signalling include the cyclooxygenase, lipooxygenase, MAP kinase (JNK and P38), AP-1, inducible nitric oxide synthase (iNOS), phosphoinositol-3 kinase (PI3K), and TGFβ pathways [50], many of which have been noted to be stimulated within the first few minutes of TLR4 ligation [51], to be stimulated by opiates [48 50], and to be associated with potentiating the effects of ageing [52-53]. Many of these immune products directly exacerbate the stem cell blockade [54]. This then implies that ODP may be conceptualized as chronically, if largely subclinically, ill.

It is very exciting that new research has identified that these processes also act particularly in proopioimelanocortin (POMC) neurons of the paraventricular and arcuate nuclei of the mediobasal hypothalamus which are key determinants of the body's metabolic homeostasis, glucostatic and lipostatic functions, energy balance, appetitive drives, sympathoadrenal activation state, and even lifespan modulation in some organisms [55-57], and that derangements in this region cause metabolic syndrome and its numerous related disorders in model organisms including mammals and likely also humans [58]. These workers have shown that activation of the MD2/TLR4/IKKβ/NF-κB cascade induces endoplasmic reticulum, mitochondrial and autophagic stress, which along with reduced neurogenesis, increased POMC neuronal apoptosis [56], and glial activation and reactive gliosis [58-59] underlie these numerous disorders and derangements. Whilst this research focuses on TLR4 ligation of unsaturated fatty acids and oxidized low density lipoproteins, opiates have been shown to act similarly as xenobiotics through MD2-TLR4 [60], and opiate agonists have been shown to produce POMC cell hyperpolarization and relative refractoriness [61], whilst opiate antagonists have the reverse effect [62]. Small molecule TLR4 inhibitors are under development for several therapeutic applications [63-64]. The confluence then of these two important lines of recent research has the potential to produce major conceptual and therapeutic advances in this field. A clear understanding of direct links from xenobiotic-TLR4-NF-κB signalling to sympathoadrenal tone and hypothalamic lifespan control has the potential to revolutionize the direction of therapeutic endeavours in the treatment of opiate dependence.

It is fascinating that the promoters for two of the key stem cell genes, Oct4 and Nanog have response elements in their promoters for STAT-1 and -3 which are commonly produced as a result of cytokine signalling, particularly by gp-130 cytokines such as IL-6 and leukaemia

For peer review only

inhibitory factor (LIF) [65-66]. Furthermore there is three way cross-talk between stem cell and metabolic –immune genes including STAT-3 and PPARγ [67]. Other cytokines, such as IL-1 have been noted in OD [8] and are usually indicative of inflammasome activation, and fractalkine and MCP-1/CCL2 have also been found to be elevated and are relevant both to neuropathological and atherogenic processes [48]. As TLR4 shares many ligands and co-chaperones with the receptor for advanced glycation end (RAGE) products this auto-amplificatory system may also be involved [68-71].

This study had various strengths and limitations. The application of sophisticated cardiovascular measurement technology to the problem of drug addiction is unique to this group. Extended follow-up and recruitment of opiate dependent patients is also notoriously difficult. The statistical techniques employed herein are advanced and robust. Design strengths include its complimentary cross-sectional and longitudinal design. Limitations of the present work relate to difficulties quantitating frequently variable amounts of past drug use in the absence of a formal measurement instrument. Use of a formal instrument such as the addiction severity index, and spot tests for drugs and alcohol are indicated in future replications of this study. Employment of structural tests, and mechanistic studies to investigate pathophysiological pathways, would be advisable in future iterations of this work.

In summary the present study documents elevated vascular stiffness and age associated with opiate use which is robust to multivariate adjustment to other known risk factors. A strong dose-response effect was noted, and the duration of treatment administration was noted to be particularly important, being related to elevations in vascular age of the first second, third and fourth order. This finding indicates limitation of treatment duration wherever clinically possible. The effect was also worse in females. Overall this study raises many further mechanistic questions which indicate further study on this complex but important model condition.

For peer review only

Acknowledgements

The authors would like to thank Dr Mervyn Thomas of Emphron for assistance with the statistics and graphical design.

Conflict of Interest

Nil declared.

Sources of Funding

Nil.

Statement of Authorship

This work is the work of the authors in its entirety.

For peer review only

References

1. Rosen D, Smith ML, Reynolds CF, 3rd. The prevalence of mental and physical health disorders among older methadone patients. *Am J Geriatr Psychiatry* 2008;16(6):488-97.
2. Cooper OB, Brown TT, Dobs AS. Opiate drug use: a potential contributor to the endocrine and metabolic complications in human immunodeficiency virus disease. *Clin Infect Dis* 2003;37 Suppl 2:S132-6.
3. Ceriello A, Quatraro A, Giugliano D. Opiate addict as diabetic patient? *Diabetes Care* 1988;11(5):443.
4. Kolarzyk E, Pach D, Wojtowicz B, Szpanowska-Wohn A, Szurkowska M. Nutritional status of the opiate dependent persons after 4 years of methadone maintenance treatment. *Przeglad lekarski* 2005;62(6):373-7.
5. Brunton LL, Lazo JS, Parker KL, editors. *Goodman and Gilman's the Pharmacologic Basis of Therapeutics*. Eleventh Edition ed. New York: McGraw Hill, 2006.
6. Reece A. S. Relative and Age Dependent Stimulation of Soluble and Cellular Immunity in Opiate Dependence. *Journal of Addiction Medicine* 2012;6(1):10-17.
7. Reece A. S. High Sensitivity CRP in Opiate Addiction: Relative and Age-Dependent Elevations. *Cardiovascular Toxicology* 2012;12(2):149-57.
8. Neri S, Bruno CM, Pulvirenti D, Malaguarnera M, Italiano C, Mauceri B, et al. Randomized clinical trial to compare the effects of methadone and buprenorphine on the immune system in drug abusers. *Psychopharmacology (Berl)* 2005;179(3):700-4.
9. Reece A. S. Absolute and Age Dependent Elevations of Serum Calcium and Phosphate and their Products in Clinical Opiate Dependence. *J. Subs. Abuse* 2013;Accepted 27th November 2012.
10. Reece AS, Davidson P. Deficit of circulating stem--progenitor cells in opiate addiction: a pilot study. *Substance abuse treatment, prevention, and policy* 2007;2:19-28.
11. Darke S, Duflou J, Torok M. The comparative toxicology and major organ pathology of fatal methadone and heroin toxicity cases. *Drug Alcohol Depend* 2010;106(1):1-6.
12. Darke S, Kaye S, Duflou J. Systemic disease among cases of fatal opioid toxicity. *Addiction* 2006;101(9):1299-305.
13. Degenhardt L., Randall D., Hall W., Law M., Butler T., Burns L. Mortality among clients of a state-wide opioid pharmacotherapy program over 20 years: Risk factors and lives saved. *Drug Alcohol Depend* 2009;105(1-2):9-15.
14. Khademi H, Malekzadeh R, Pourshams A, Jafari E, Salahi R, Semnani S, et al. Opium use and mortality in Golestan Cohort Study: prospective cohort study of 50,000 adults in Iran. *BMJ (Clinical research ed* 2012;344:e2502.
15. Sadeghian S, Darvish S, Davoodi G, Salarifar M, Mahmoodian M, Fallah N, et al. The association of opium with coronary artery disease. *Eur J Cardiovasc Prev Rehabil* 2007;14(5):715-7.
16. Sadeghian S, Dowlatshahi S, Karimi A, Tazik M. Epidemiology of opium use in 4398 patients admitted for coronary artery bypass graft in Tehran Heart Center. *J Cardiovasc Surg (Torino)* 2011;52(1):140-1.
17. Sadeghian S, Graili P, Salarifar M, Karimi AA, Darvish S, Abbasi SH. Opium consumption in men and diabetes mellitus in women are the most important risk factors of premature coronary artery disease in Iran. *International journal of cardiology* 2010;141(1):116-8.
18. Smyth B, Hoffman V, Fan J, Hser YI. Years of potential life lost among heroin addicts 33 years after treatment. *Preventive medicine* 2007;44(4):369-74.
19. Reece A.S., Hulse G.K. Impact of Opioid Pharmacotherapy on Arterial stiffness and Vascular Ageing: Cross-sectional and Longitudinal Studies. *Cardiovascular Toxicology* 2013.
20. Reece A. S., Hulse G.K. Reduction in Arterial Stiffness and Vascular Age by Naltrexone-Induced Interruption of Opiate Agonism. *British Medical Journal - Open* 2013.

For peer review only

21. Charney DS, Redmond DE, Jr., Galloway MP, Kleber HD, Heninger GR, Murberg M, et al. Naltrexone precipitated opiate withdrawal in methadone addicted human subjects: evidence for noradrenergic hyperactivity. *Life Sci* 1984;35(12):1263-72.
22. Reece AS, Hulse GK. Elevation of Central Arterial Stiffness and Vascular Ageing in Opiate Withdrawal: Cross-sectional and Longitudinal Studies. *Cardiovasc Toxicol* 2012.
23. Chien KR, Karsenty G. Longevity and lineages: toward the integrative biology of degenerative diseases in heart, muscle, and bone. *Cell* 2005;120(4):533-44.
24. Lakatta EG, Wang M, Najjar SS. Arterial aging and subclinical arterial disease are fundamentally intertwined at macroscopic and molecular levels. *Med Clin North Am* 2009;93(3):583-604, Table of Contents.
25. Reece AS. Hair graying in substance addiction. *Archives of dermatology* 2007;143(1):116-8.
26. Kim TW, Alford DP, Malabanan A, Holick MF, Samet JH. Low bone density in patients receiving methadone maintenance treatment. *Drug Alcohol Depend* 2006;85(3):258-62.
27. Reece AS. Evidence of Accelerated Ageing in Clinical Drug Addiction from Immune, Hepatic and Metabolic Biomarkers. *Immun Ageing* 2007;4(1):6-15.
28. Randall D, Degenhardt L, Vajdic CM, Burns L, Hall WD, Law M, et al. Increasing cancer mortality among opioid-dependent persons in Australia: a new public health challenge for a disadvantaged population. *Australian and New Zealand journal of public health* 2011;35(3):220-5.
29. Reece A.S., Hulse G.K. Lifetime Opiate Exposure as an Independent and Interactive Cardiovascular Risk Factor in Males: A Cross-Sectional Clinical Study. *Vascular Health and Risk Management* 2013;In Press.
30. Reece A.S., Hulse G.K. Opiate Exposure Increases Arterial Stiffness, Advances Vascular Age and is an Independent Cardiovascular Risk Factor in Females: A Cross-Sectional Clinical Study. *World J. Cardiovascular Diseases* 2013;In Press.
31. Reece A. S., Hulse G.K. Duration of Opiate Exposure as a Determinant of Arterial Stiffness and Vascular Age in Male Opiate Dependence: A Longitudinal Study. *Journal of Clinical Pharmacy and Therapeutics* 2013(In Press).
32. Reece A. S., Hulse G.K. Opiate Dependence as an Independent and Interactive Risk Factor for Arterial Stiffness and Cardiovascular Ageing - A Longitudinal Study in Females. *J Clinical Medical Research* 2013;In Press.
33. Reece AS. Psychosocial and treatment correlates of opiate free success in a clinical review of a naltrexone implant program. *Subst Abuse Treat Prev Policy* 2007;2:35-49.
34. Breslow N.E., Day N.E. *Statistical Methods in Cancer Research Volume II The Design and Analysis of Cohort Studies*. 1 ed. Lyon: International Agency for Cancer Research,, 1987.
35. Kacinko SL, Jones HE, Johnson RE, Choo RE, Huestis MA. Correlations of maternal buprenorphine dose, buprenorphine, and metabolite concentrations in meconium with neonatal outcomes. *Clinical pharmacology and therapeutics* 2008;84(5):604-12.
36. Hulse G., White J., Cape G., editors. *Management of Drug and Alcohol Problems*. Sydney: Oxford University Press, 2002.
37. Oviedo-Joekes E, Brissette S, Marsh DC, Lauzon P, Guh D, Anis A, et al. Diacetylmorphine versus methadone for the treatment of opioid addiction. *N Engl J Med* 2009;361(8):777-86.
38. SphygmoCor, editor. *Software Operator's Guide, Pulse Wave Analysis System, SCOR-Px*. Sydney: AtCor Medical, 2006.
39. Klein SL. Sex influences immune responses to viruses, and efficacy of prophylaxis and treatments for viral diseases. *Bioessays* 2012;34(12):1050-9.
40. Nichols W.W., O'Rourke M.F., editors. *McDonald's Blood Flow in Arteries: Theoretical, Experimental and Clinical Principals*. London: Hodder Arnold, 2005.
41. O'Rourke MF, Pauca AL. Augmentation of the aortic and central arterial pressure waveform. *Blood Press Monit* 2004;9(4):179-85.
42. Weber T, Auer J, O'Rourke MF, Kvas E, Lassnig E, Berent R, et al. Arterial stiffness, wave reflections, and the risk of coronary artery disease. *Circulation* 2004;109(2):184-9.
43. Williams B, Lacy PS, Thom SM, Cruickshank K, Stanton A, Collier D, et al. Differential impact of blood pressure-lowering drugs on central aortic pressure and clinical outcomes: principal

For peer review only

results of the Conduit Artery Function Evaluation (CAFE) study. *Circulation* 2006;113(9):1213-25.
44. Nichols WW. Clinical measurement of arterial stiffness obtained from noninvasive pressure waveforms. *Am J Hypertens* 2005;18(1 Pt 2):3S-10S.
45. Cheng F, McLaughlin PJ, Verderame MF, Zagon IS. The OGF-OGFr axis utilizes the p16INK4a and p21WAF1/CIP1 pathways to restrict normal cell proliferation. *Molecular biology of the cell* 2009;20(1):319-27.
46. Zagon IS, Verderame MF, McLaughlin PJ. The biology of the opioid growth factor receptor (OGFr). *Brain research* 2002;38(3):351-76.
47. Pasmant E, Sabbagh A, Vidaud M, Bieche I. ANRIL, a long, noncoding RNA, is an unexpected major hotspot in GWAS. *FASEB J* 2010;25(2):444-48.
48. Hutchinson MR, Shavit Y, Grace PM, Rice KC, Maier SF, Watkins LR. Exploring the neuroimmunopharmacology of opioids: an integrative review of mechanisms of central immune signaling and their implications for opioid analgesia. *Pharmacological reviews* 2011;63(3):772-810.
49. Starr ME, Hu Y, Stromberg AJ, Carmical JR, Wood TG, Evers BM, et al. Gene expression profile of mouse white adipose tissue during inflammatory stress: age-dependent upregulation of major pro-coagulant factors. *Aging Cell* 2012:n/a-n/a.
50. Coller JK, Hutchinson MR. Implications of central immune signaling caused by drugs of abuse: mechanisms, mediators and new therapeutic approaches for prediction and treatment of drug dependence. *Pharmacol Ther* 2012;134(2):219-45.
51. Grzanna R., Brown R.M. NIDA Research Monograph 125: Activation of Intermediate Early Genes by Drugs of Abuse. In: National Institutes of Drug Abuse, editor. Bethesda, Maryland: NIH, 1993:1-220.
52. Hadley EC, Lakatta EG, Morrison-Bogorad M, Warner HR, Hodes RJ. The future of aging therapies. *Cell* 2005;120(4):557-67.
53. Kirkwood TB. Understanding the odd science of aging. *Cell* 2005;120(4):437-47.
54. Hoing S, Rudhard Y, Reinhardt P, Glatza M, Stehling M, Wu G, et al. Discovery of inhibitors of microglial neurotoxicity acting through multiple mechanisms using a stem-cell-based phenotypic assay. *Cell Stem Cell* 2012;11(5):620-32.
55. Zhang G, Li J, Purkayastha S, Tang Y, Zhang H, Yin Y, et al. Hypothalamic programming of systemic ageing involving IKK-[bgr], NF-[kgr]B and GnRH. *Nature* 2013;497(7448):211-16.
56. Li J, Tang Y, Cai D. IKKbeta/NF-kappaB disrupts adult hypothalamic neural stem cells to mediate a neurodegenerative mechanism of dietary obesity and pre-diabetes. *Nat Cell Biol* 2012;14(10):999-1012.
57. Purkayastha S, Zhang G, Cai D. Uncoupling the mechanisms of obesity and hypertension by targeting hypothalamic IKK-beta and NF-kappaB. *Nat Med* 2011;17(7):883-7.
58. Thaler JP, Yi CX, Schur EA, Guyenet SJ, Hwang BH, Dietrich MO, et al. Obesity is associated with hypothalamic injury in rodents and humans. *J Clin Invest* 2012;122(1):153-62.
59. Czapski GA, Gajkowska B, Strosznajder JB. Systemic administration of lipopolysaccharide induces molecular and morphological alterations in the hippocampus. *Brain Res* 2010;1356:85-94.
60. Wang X, Loram LC, Ramos K, de Jesus AJ, Thomas J, Cheng K, et al. Morphine activates neuroinflammation in a manner parallel to endotoxin. *Proc Natl Acad Sci U S A* 2012;109(16):6325-30.
61. Cowley MA, Smart JL, Rubinstein M, Cerdan MG, Diano S, Horvath TL, et al. Leptin activates anorexigenic POMC neurons through a neural network in the arcuate nucleus. *Nature* 2001;411(6836):480-4.
62. Billes SK, Greenway FL. Combination therapy with naltrexone and bupropion for obesity. *Expert opinion on pharmacotherapy* 2011;12(11):1813-26.
63. Neal MD, Jia H, Eyer B, Good M, Guerriero CJ, Sodhi CP, et al. Discovery and validation of a new class of small molecule Toll-like receptor 4 (TLR4) inhibitors. *PLoS ONE* 2013;8(6):e65779.

For peer review only

64. Theberge FR, Li X, Kambhampati S, Pickens CL, St Laurent R, Bossert JM, et al. Effect of chronic delivery of the Toll-like receptor 4 antagonist (+)-naltrexone on incubation of heroin craving. *Biol Psychiatry* 2013;73(8):729-37.
65. Kim CG, Chung IY, Lim Y, Lee YH, Shin SY. A Tcf/Lef element within the enhancer region of the human NANOG gene plays a role in promoter activation. *Biochem Biophys Res Commun* 2011;410(3):637-42.
66. Yang HM, Do HJ, Oh JH, Kim JH, Choi SY, Cha KY, et al. Characterization of putative cis-regulatory elements that control the transcriptional activity of the human Oct4 promoter. *J Cell Biochem* 2005;96(4):821-30.
67. Folmes CD, Dzeja PP, Nelson TJ, Terzic A. Metabolic plasticity in stem cell homeostasis and differentiation. *Cell Stem Cell* 2012;11(5):596-606.
68. Zurolo E, Iyer A, Maroso M, Carbonell C, Anink JJ, Ravizza T, et al. Activation of Toll-like receptor, RAGE and HMGB1 signalling in malformations of cortical development. *Brain* 2011;134(Pt 4):1015-32.
69. Nogueira-Machado JA, Volpe CM, Veloso CA, Chaves MM. HMGB1, TLR and RAGE: a functional tripod that leads to diabetic inflammation. *Expert opinion on therapeutic targets* 2011;15(8):1023-35.
70. Sakaguchi M, Murata H, Yamamoto K, Ono T, Sakaguchi Y, Motoyama A, et al. TIRAP, an adaptor protein for TLR2/4, transduces a signal from RAGE phosphorylated upon ligand binding. *PLoS ONE* 2011;6(8):e23132.
71. Veloso CA, Fernandes JS, Volpe CM, Fagundes-Netto FS, Reis JS, Chaves MM, et al. TLR4 and RAGE: similar routes leading to inflammation in type 2 diabetic patients. *Diabetes Metab* 2011;37(4):336-42.

For peer review only

Figure Captions:

Figure 1: Ageing and Stiffness Indices by Chronologic Age by Opiate Dependency Status

Figure 2: Corrected Ageing and Stiffness Indices by Time by Opiate Dependency Status

Figure 3: Ageing Indices by Chronologic Age by Addictive Status by Sex

Figure 4: Ageing Indices by Chronologic Age by Sex by Addictive Status

Figure 5: BMI-Related Ageing Indices by Chronologic Age by Addictive Status by Sex

Figure 6: BMI-Related Ageing Indices by Chronologic Age by Sex by Addictive Status

Figure 7: Ageing Indices by Chronologic Age by Sex by Addictive Status – Loess Curves

Figure 8: Vascular Age Indices by Chronologic Age by Opiate Dependency Level, Loess curves

Supplementary Figure Captions:

Suppl. Fig. 1: Vascular Age Indices by Time by Opiate Dependency Level

For peer review only

TABLES

For peer review only

Table 1.: Effects of Gender on Key Measures of Arterial Stiffness - Cross-sectional Dataset

Dependent Variable	Group	Parameter	Adj. R^2	F	dF	Model P	Estimate	Std. Error	t-Value	P
RA-CA Difference	Opiate	Chron.Age :Female.Sex	0.0189	7.596	2, 684	0.0005459	0.6324	0.3469	1.823	0.0688
BMI	Control	Chron.Age :Female.Sex	0.1523	52.58	2, 572	<2.2E-16	-0.0092	0.0040	-2.310	0.0213
BMI	Opiate	Female.Sex	0.0713	27.35	2, 684	3.75E-12	-0.0827	0.0139	-5.940	4.6E-09
RA/BMI	Opiate	Chron.Age :Female.Sex	0.2617	122.6	2, 684	<2.2E-16	0.0361	0.0091	3.948	8.7E-05
C_AP_HR75/BMI	Control	Chron.Age :Female.Sex	0.4882	274.3	2, 571	<2.2E-16	0.0312	0.0040	7.737	4.6E-14
C_AP_HR75/BMI	Control	Chron.Age :Female.Sex	0.4882	274.3	2, 571	<2.2E-16	0.0312	0.0040	7.737	4.6E-14
C_AP_HR75/BMI	Opiate	Chron.Age :Female.Sex	0.3712	203.4	2, 684	<2.2E-16	0.0467	0.0039	11.950	<2e-16
C_AGPH_HR75/BMI	Control	Chron.Age :Female.Sex	0.4603	245.4	2, 571	<2.2E-16	0.0924	0.0112	8.283	8.6E-16
C_AGPH_HR75/BMI	Opiate	Chron.Age :Female.Sex	0.3980	227.8	2, 684	<2.2E-16	0.1265	0.0095	13.370	<2e-16
C_AP_HR75/(CA*BMI)	Control	Female.Sex	0.2809	112.9	2, 571	<2.2E-16	0.0033	0.0005	6.286	6.5E-10
C_AP_HR75/(CA*BMI)	Opiate	Female.Sex	0.2489	114.7	2, 684	<2.2E-16	0.0047	0.0004	11.428	<2e-16
C_AGPH_HR75/(CA*BMI)	Control	Female.Sex	0.2567	99.96	2, 571	<2.2E-16	0.0096	0.0015	6.350	4.4E-10
C_AGPH_HR75/(CA*BMI)	Opiate	Female.Sex	0.2604	121.8	2, 684	<2.2E-16	0.0130	0.0011	12.307	<2e-16

For peer review only

Table 2.:

Age: Addictive Status Relationships with CA Discretized

About 41 Years in Males and 29 Years in Females

With Females <55 Years of Age

Parameter	Variable Statistics				Model Summaries		
	Estimate	Std. Error	t Value	Pr(>|t|)	F	dF	P
Males							
Older Age	-0.1837	0.0557	-3.296	0.0010	5.492	3, 862	0.00097
Opiates	-0.0846	0.0275	-3.082	0.0021			
Older Age: Opiates	0.2310	0.0687	3.364	0.0008			
Females							
Older Age: Opiates	0.1248	0.0532	2.346	0.0195	2.169	3, 378	0.09123

For peer review only

Table 3.: Cross-sectional Models Polynomial in Heroin Duration

	Parameter				Model		
Model / Parameter	Value	Std.Error	t-value	P-value	F	dF	P
1 Order- Linear							
H.Dura.n	-0.0059	0.0021	-2.779	0.0056	8.628	2, 664	0.0002
H.Dose: H.Dura.n	0.0091	0.0022	4.152	3.7.E-05			
2 Order- Quadratic							
H.Dura.n	-0.0171	0.0055	-3.136	0.0018	7.443	3, 663	6.60E-05
H.Dura.n^2	0.0004	0.0002	2.229	0.0261			
H.Dose: H.Dura.n	0.0084	0.0022	3.802	0.0002			
3 Order - Cubic							
H.Dura.n	-0.0116	0.0035	-3.263	0.0012	7.102	3 663	0.00011
H.Dura.n^3	6.3E-06	3.1E-06	1.993	0.0466			
H.Dose: H.Dura.n	0.0084	0.0022	3.836	0.0001			
4 Order- Quartic							
H.Dura.n	-0.0059	0.0021	-2.779	0.0056	8.628	2, 664	0.0002
H.Dose: H.Dura.n	0.0091	0.0022	4.152	3.7.E-05			
1, 2, 3 & 4 Order							
H.Dose: H.Dura.n^3	6.5E-05	2.0E-05	3.252	0.0012	3.353	7, 659	0.0016
H.Dura.n^2 : H.Dura.n^3	-1.0E-06	3.1E-07	-3.398	0.0007			
H.Dura.n^2 : H.Dura.n^4	9.2E-08	2.9E-08	3.216	0.0014			
H.Dura.n^3: H.Dura.n^4	-2.7E-09	8.8E-10	-3.029	0.0026			
H.Dose: H.Dura.n^2 : H.Dura.n^4	-4.5E-09	1.8E-09	-2.452	0.0145			
H.Dura.n: H.Dura.n^3: H.Dura.n^4	2.5E-11	9.0E-12	2.831	0.0048			
H.Dose: H.Dura.n: H.Dura.n^3: H.Dura.n^4	2.6E-12	1.2E-12	2.159	0.0312			

For peer review only

Table 4: Dose-Response Relationships

Cross-Sectional Studies

Parameter	Variable Statistics				Model Summaries		
	Estimate	Std. Error	t Value	Pr(>|t|)	F	dF	P
Both Sexes							
H.Dura.n	-0.0059	0.0021	-2.779	0.0056	8.628	2, 664	0.0002
H.Dose: H.Dura.n	0.0091	0.0022	4.152	0.0000			
Males							
H.Dura.n	-0.0089	0.0028	-3.147	0.0018	7.105	2, 441	0.0009
H.Dose: H.Dura.n	0.0124	0.0034	3.658	0.0003			
Females							
H.Dose: H.Dura.n	0.0052	0.0023	2.281	0.0236	5.201	1, 210	0.0236

Longitudinal Studies

-

Group	Variable Statistics					Model	
**Parameter**	**Value**	**Std.Error**	**DF**	**t-value**	**P-value**	**AIC***	**LogLik***
Both Sexes							
H.Dose:H.Dura.n	0.0045	0.0013	814	3.5512	0.0004	1068.74	-530.37
Males							
H.Dura.n	-0.0036	0.0016	552	-2.2069	0.0277	753.15	-371.58
H.Dose:H.Dura.n	0.0075	0.0022	552	3.3754	0.0008		
Females							
H.Dose:H.Dura.n	0.0052	0.0021	260	2.5091	0.0127	347.43	-169.72

For peer review only

Table 5.: Cross-Sectional Adjusted Multivariate Models

Group	Variable Statistics				Model		
Parameter	Estimate	Std. Error	t Value	Pr(>|t|)	F	dF	P
Both Sexes							
H.Dura.n: Cigs.d: SP : Weight	0.00023	0.00006	3.919	0.0001	7.561	9, 657	1.38E-10
H.Dura.n: Height: Cigs.d: SP	0.00030	0.00011	2.840	0.0047			
H.Dura.n: Height: Cigs.d	-0.00143	0.00051	-2.828	0.0048			
H.Dura.n: Height: Cigs.d: SP : Weight	-0.00007	0.00002	-2.808	0.0051			
H.Dura.n: Height: Cigs.d: Weight	0.00032	0.00012	2.741	0.0063			
H.Dose: H.Dura.n: Height: Cigs.d: Weight	-0.00037	0.00016	-2.229	0.0261			
H.Dose: H.Dura.n: Height: Cigs.d: SP : Weight	0.00008	0.00003	2.229	0.0262			
H.Dose: H.Dura.n: Height: Cigs.d	0.00155	0.00071	2.194	0.0286			
H.Dose: H.Dura.n: Height: Cigs.d: SP	-0.00032	0.00015	-2.188	0.0290			
Males							
H.Dura.n: Weight	-0.0024	0.0007	-3.434	0.0007	5.485	10, 438	1.07E-07
H.Dose: H.Dura.n: Weight	0.0032	0.0010	3.158	0.0017			
H.Dose: Cigs.d: SP : Weight	-4.4770	1.7470	-2.563	0.0107			
H.Dose: Height: Cigs.d: SP : Weight	0.0253	0.0099	2.557	0.0109			
H.Dose: Cigs.d: SP	19.460	7.6160	2.556	0.0109			
H.Dose: Cigs.d: Weight	21.370	8.3900	2.548	0.0112			
H.Dose: Height: Cigs.d: Weight	-0.1209	0.0476	-2.542	0.0114			
H.Dose: Height: Cigs.d: SP	-0.1098	0.0432	-2.540	0.0114			
H.Dose: Cigs.d	-92.680	36.520	-2.538	0.0115			
H.Dose: Height: Cigs.d	0.5230	0.2073	2.523	0.0120			
Females							
Height: Cigs.d: Weight	-0.00041	0.00009	-4.327	0.00002	8.25	3, 214	3.20E-05
Height: Cigs.d: SP : Weight	0.00008	0.00002	4.272	0.00003			
H.Dose: H.Dura.n: Height: Cigs.d	2.2E-06	1.1E-06	1.957	0.05160			

For peer review only

Table 6.: Longitudinal Adjusted Multivariate Models

Group	Variable Statistics					Model	
**Model / Parameter**	**Value**	**Std.Error**	**DF**	**t-value**	**P-value**	**AIC***	**LogLik***
Both Sexes							
Height	-0.0304	0.0026	606	-11.7198	0.0E+00	1238.239	-592.1197
SP	0.7947	0.0949	606	8.3772	0.0E+00		
Days:H.Dura.n	0.0003	6.9E-05	606	4.3556	0.0E+00		
CA:Height	0.0066	0.0006	606	10.9049	0.0E+00		
Days: CA:H.Dura.n	-0.0001	0.0000	606	-4.2946	0.0E+00		
H.Dura.n: Cigs.d:Height	-5.3E-05	1.3E-05	606	-4.1566	0.0E+00		
Days:H.Dose: Cigs.d:Height	2.1E-06	5.0E-07	606	4.3108	0.0E+00		
Days: CA:H.Dose: Cigs.d:Height	-6.0E-07	2.0E-07	606	-4.2968	0.0E+00		
CA:H.Dura.n: Cigs.d:Height	1.4E-05	3.4E-06	606	4.0217	0.0001		
Days: CA: Cigs.d:Height	0.0E+00	0.0E+00	606	3.7701	0.0002		
Days:H.Dura.n: Cigs.d	-1.1E-05	3.1E-06	606	-3.4709	0.0006		
Cigs.d:Height	0.0007	0.0002	606	3.3714	0.0008		
Days: CA:H.Dose:H.Dura.n: Cigs.d:Height	0.0E+00	0.0E+00	606	3.2705	0.0011		
Days: CA:H.Dura.n: Cigs.d	2.5E-06	8.0E-07	606	3.2365	0.0013		
CA: Cigs.d:Height	-0.0002	0.0001	606	-3.2045	0.0014		
Days	-0.0022	0.0007	606	-2.9194	0.0036		
H.Dose:H.Dura.n: Cigs.d	0.0102	0.0036	606	2.8304	0.0048		
CA:H.Dose:H.Dura.n: Cigs.d	-0.0026	0.0010	606	-2.6804	0.0076		
Days: CA	0.0005	0.0002	606	2.2438	0.0252		
Days: CA:H.Dose:Height	6.8E-06	3.1E-06	606	2.1984	0.0283		
Days:H.Dose:Height	-2.2E-05	1.0E-05	606	-2.1747	0.0300		
Days:H.Dose:H.Dura.n	-3.5E-05	1.7E-05	606	-2.1086	0.0354		
H.Dose: Cigs.d	-0.1067	0.0511	606	-2.0888	0.0371		
CA:H.Dose: Cigs.d	0.0290	0.0148	606	1.9645	0.0499		
Males							
Height	-0.0302	0.0035	436	-8.7423	0.0E+00	913.00	-435.50
SP	0.6376	0.1105	436	5.7717	0.0E+00		
CA:Height	0.0064	0.0008	436	8.4711	0.0E+00		
Days:H.Dura.n	0.0003	0.0001	436	3.8907	0.0001		
Days: CA:H.Dura.n	-0.0001	0.0000	436	-3.8395	0.0001		
Days:H.Dose: Cigs.d:Height	1.6E-06	4.0E-07	436	3.8181	0.0002		
Days: CA:H.Dose: Cigs.d:Height	-5.0E-07	1.0E-07	436	-3.7217	0.0002		
Days	-0.0029	0.0008	436	-3.6624	0.0003		
Days: CA	0.0007	0.0002	436	3.3251	0.0010		
H.Dose:H.Dura.n: Cigs.d:Height	2.7E-06	8.0E-07	436	3.2331	0.0013		
Days: CA: Cigs.d	5.0E-06	1.6E-06	436	3.1839	0.0016		
H.Dura.n: Cigs.d:Height	-2.8E-05	9.1E-06	436	-3.1058	0.0020		
CA:H.Dura.n: Cigs.d:Height	7.1E-06	2.4E-06	436	2.9496	0.0034		
Days:H.Dura.n: Cigs.d	-8.3E-06	3.1E-06	436	-2.6824	0.0076		
Days: CA:H.Dura.n: Cigs.d	2.0E-06	8.0E-07	436	2.5562	0.0109		
Days: CA:H.Dose:H.Dura.n: Cigs.d:Height	0.0E+00	0.0E+00	436	2.2838	0.0229		
Cigs.d:Height	0.0004	0.0002	436	2.2799	0.0231		
CA: Cigs.d:Height	-0.0001	5.0E-05	436	-2.1938	0.0288		

For peer review only

Females							
Height	-0.0279	0.0036	169	-7.6753	0.0E+00	394.69	-187.35
SP	1.2880	0.1930	169	6.6744	0.0E+00		
CA:Height	0.0064	0.0005	169	11.7493	0.0E+00		
Days: CA:H.Dose:H.Dura.n:Height	1.3E-06	5.0E-07	169	2.7371	0.0069		
Days:H.Dose:H.Dura.n:Height	-4.5E-06	1.7E-06	169	-2.7147	0.0073		
Days: CA:H.Dose:H.Dura.n: Cigs.d:Height	-1.0E-07	0.0E+00	169	-2.6052	0.0100		
Days:H.Dose:H.Dura.n: Cigs.d:Height	2.0E-07	1.0E-07	169	2.5589	0.0114		

For peer review only

Figure 1

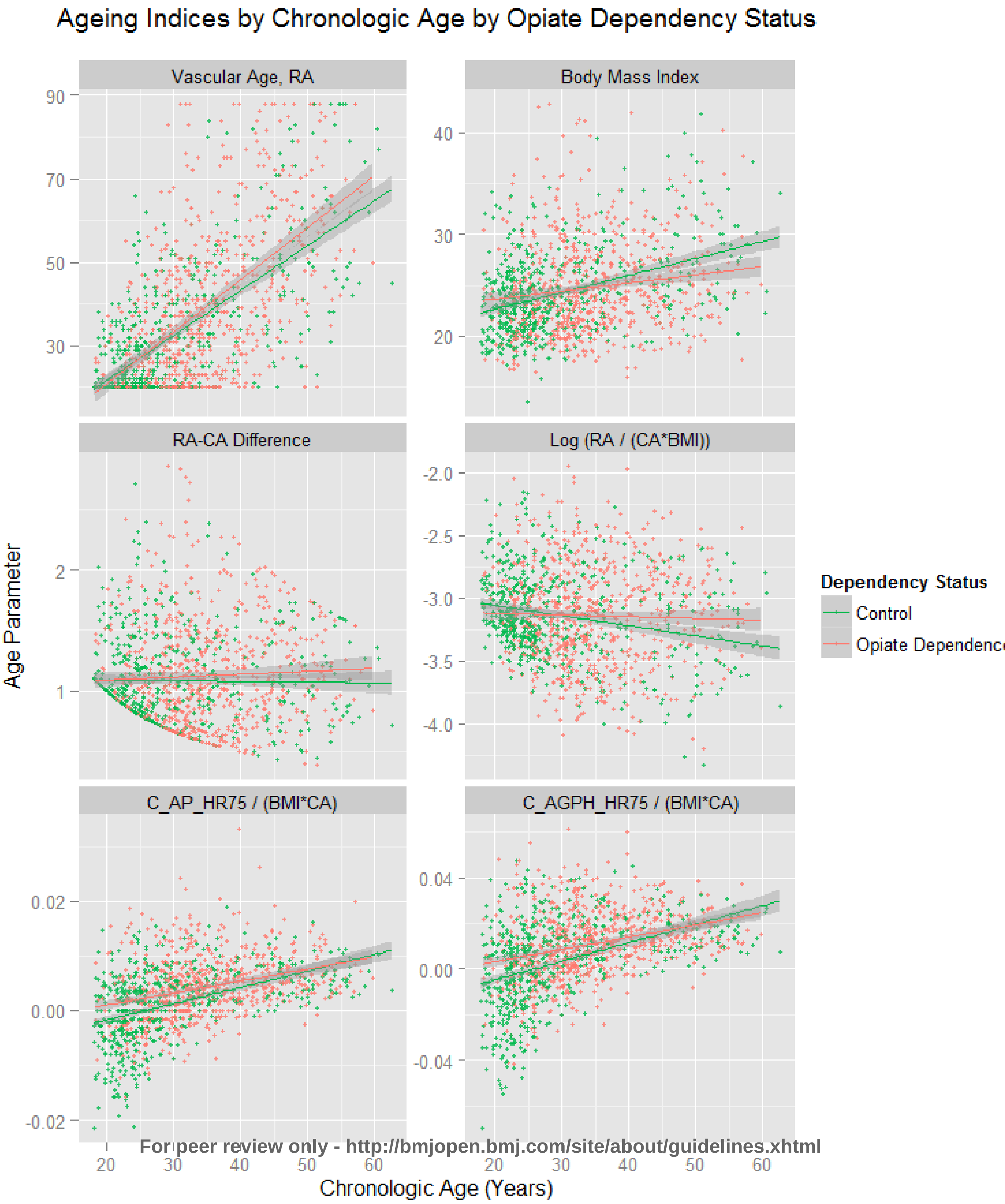
Ageing Indices by Chronologic Age by Opiate Dependency Status
Vascular Age, RA
Body Mass Index
RA-CA Difference
Log (RA / (CA*BMI))
C_AP_HR75 / (BMI*CA)
C_AGPH_HR75 / (BMI*CA)
Age Parameter
Chronologic Age (Years)
Dependency Status
Control
Opiate Dependenc

Figure 2

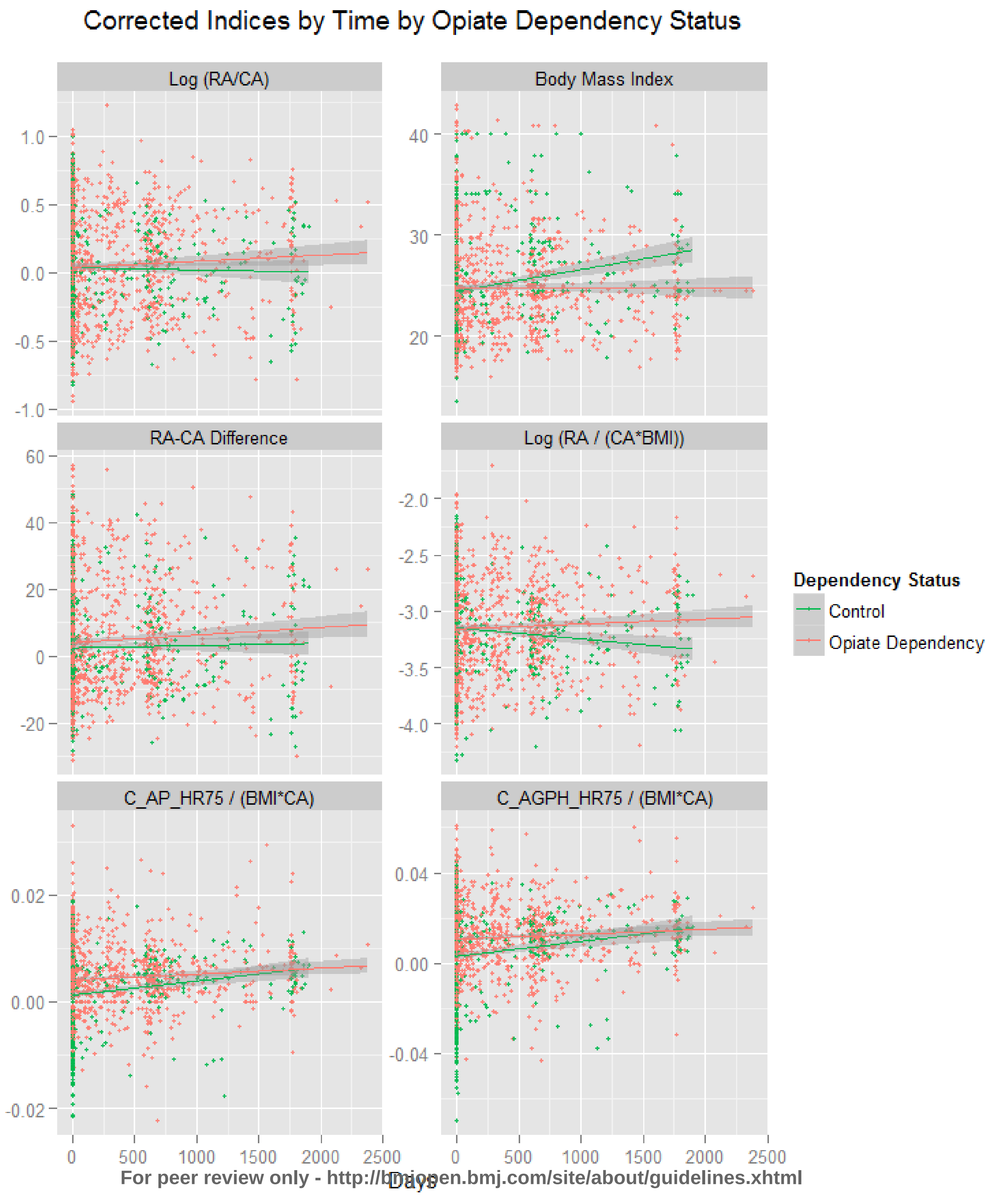
Corrected Indices by Time by Opiate Dependency Status
Log (RA/CA)
Body Mass Index
RA-CA Difference
Log (RA / (CA*BMI))
C_AP_HR75 / (BMI*CA)
C_AGPH_HR75 / (BMI*CA)
Parameter
Days
Dependency Status
Control
Opiate Dependency

Figure 3

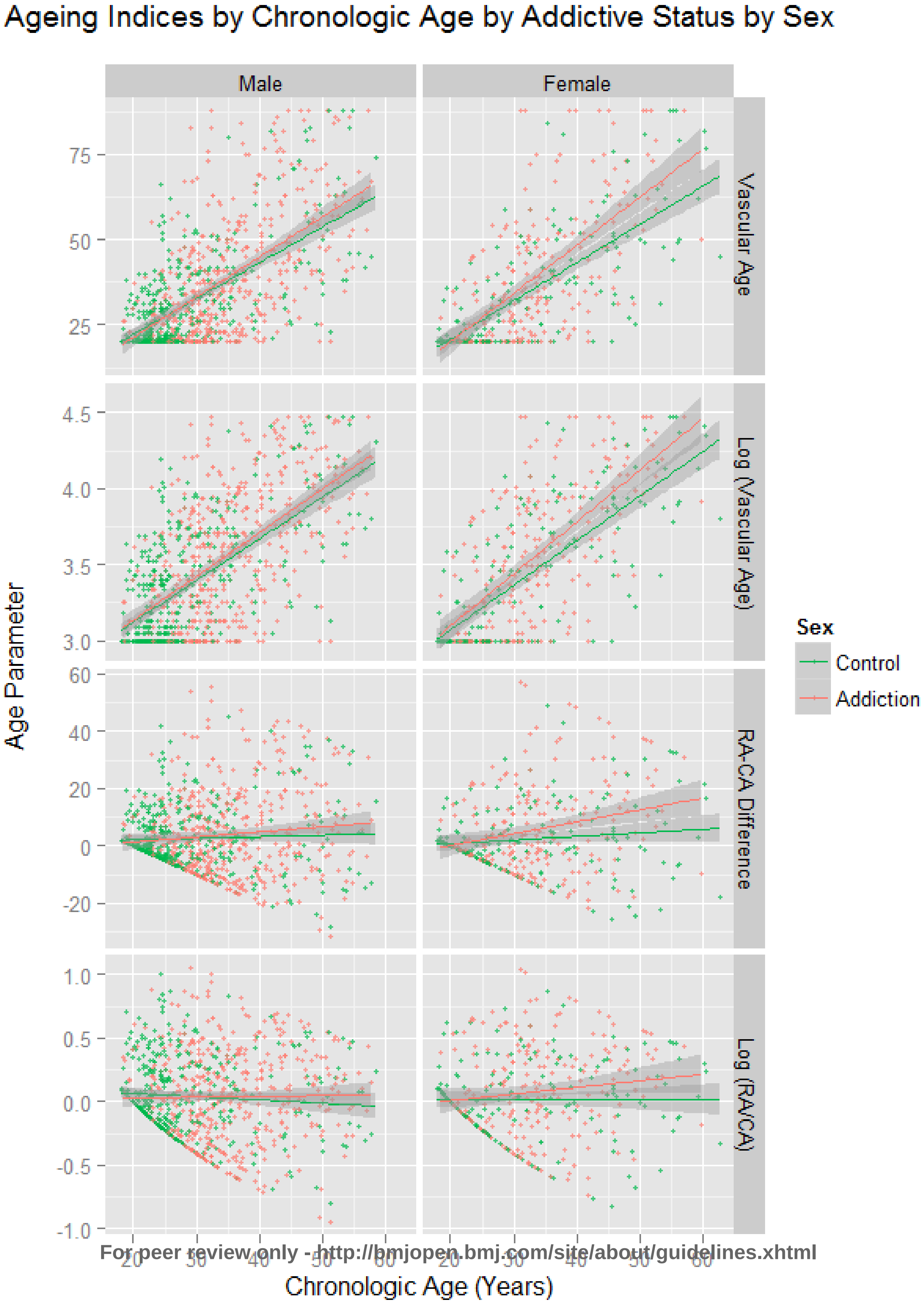
Ageing Indices by Chronologic Age by Addictive Status by Sex
Male
Female
Vascular Age
Log (Vascular Age)
RA-CA Difference
Log (RA/CA)
Age Parameter
Chronologic Age (Years)
Sex
Control
Addiction
75
50
25
4.5
4.0
3.5
3.0
60
40
20
0
-20
1.0
0.5
0.0
-0.5
-1.0
20
30
40
50
60

Figure 4

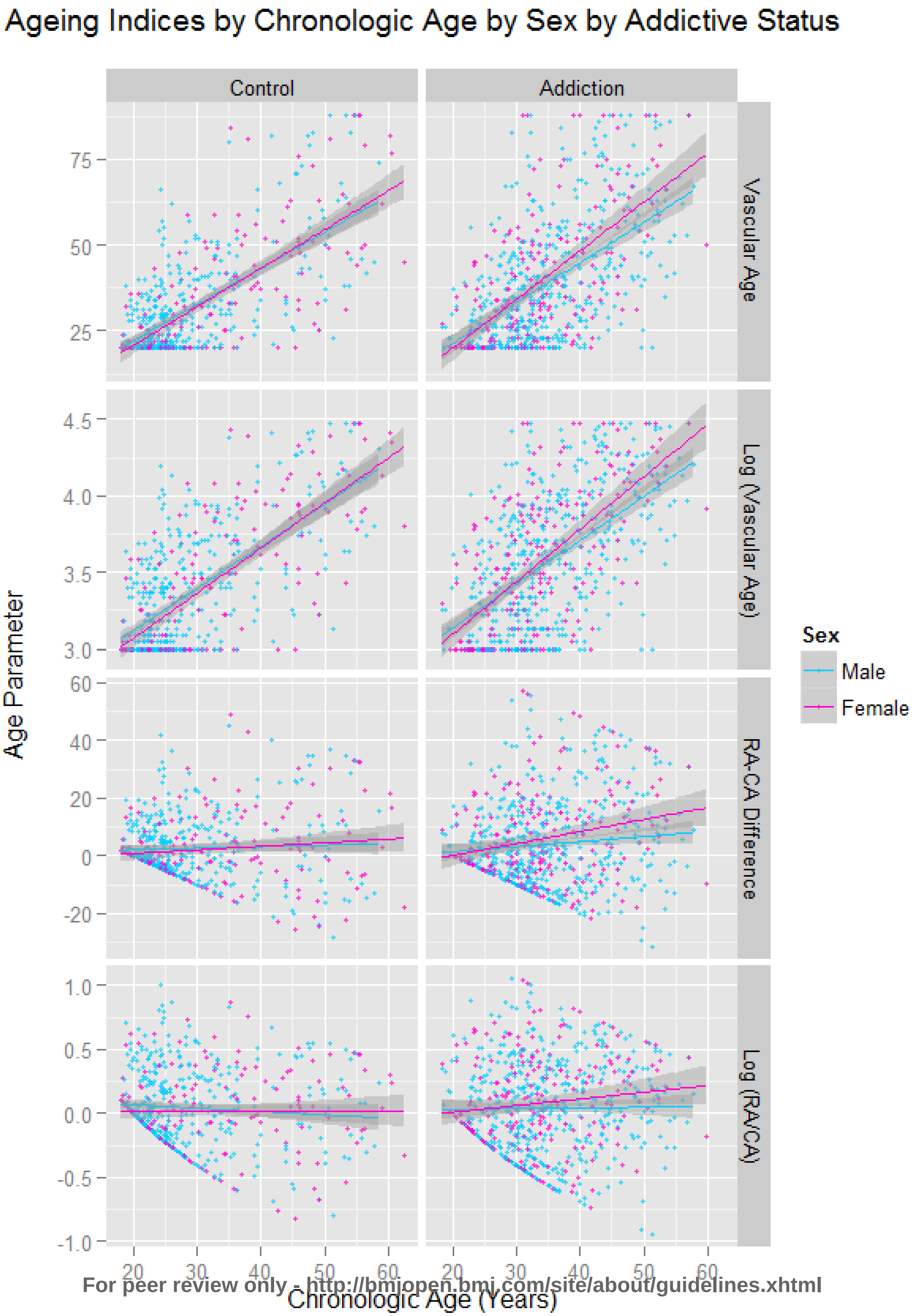

Ageing Indices by Chronologic Age by Sex by Addictive Status
Control
Addiction
Vascular Age
Log (Vascular Age)
RA-CA Difference
Log (RA/CA)
Age Parameter
Chronologic Age (Years)
Sex
Male
Female
75
50
25
4.5
4.0
3.5
3.0
60
40
20
0
-20
1.0
0.5
0.0
-0.5
-1.0
20
30
40
50
60

Figure 5

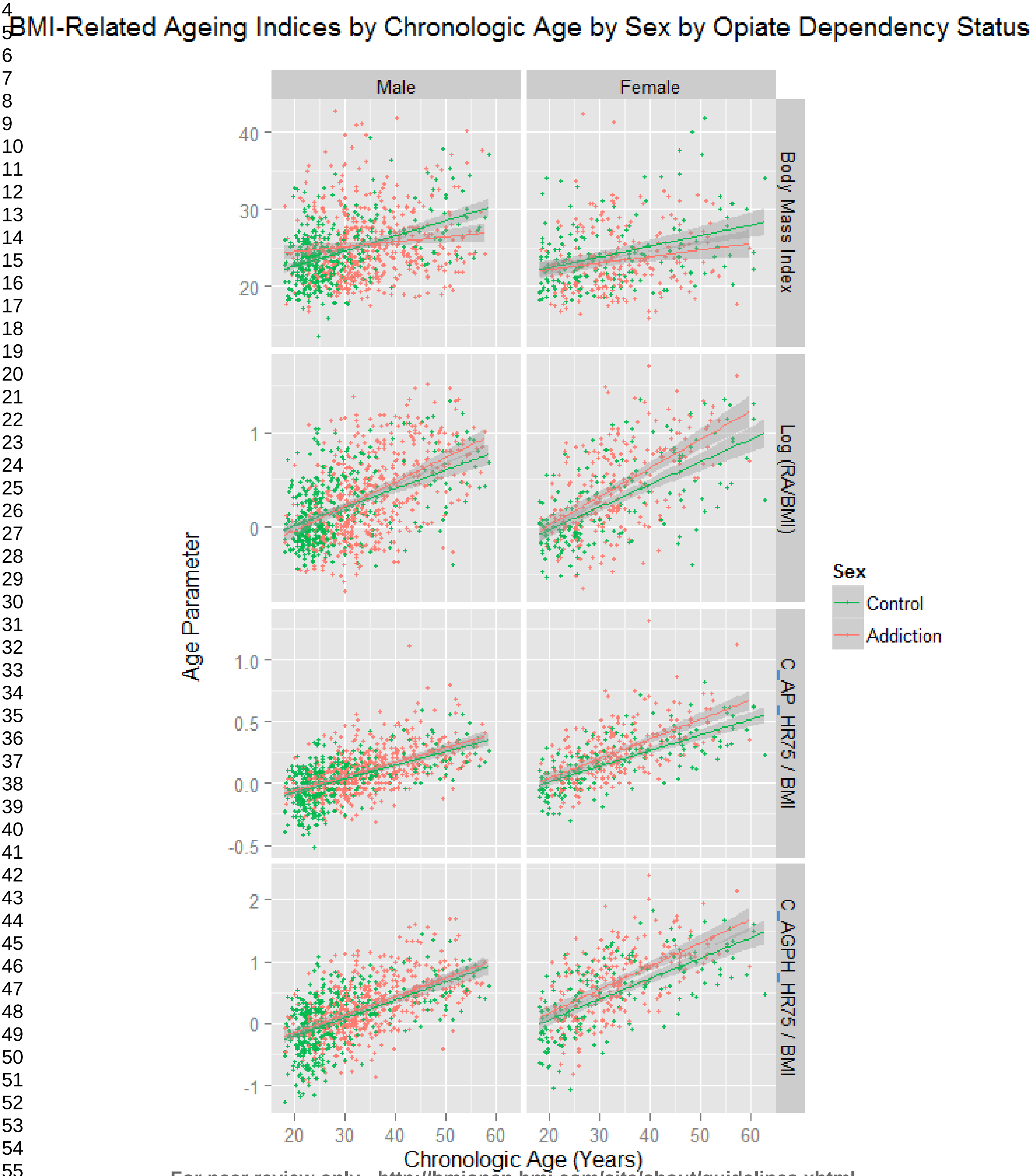
BMI-Related Ageing Indices by Chronologic Age by Sex by Opiate Dependency Status
Male
Female
Body Mass Index
Log (RA/BMI)
C_AP_HR75 / BMI
C_AGPH_HR75 / BMI
Age Parameter
Chronologic Age (Years)
Sex
Control
Addiction
40
30
20
1
0
1.0
0.5
0.0
-0.5
2
1
0
-1
20
30
40
50
60

Figure 6

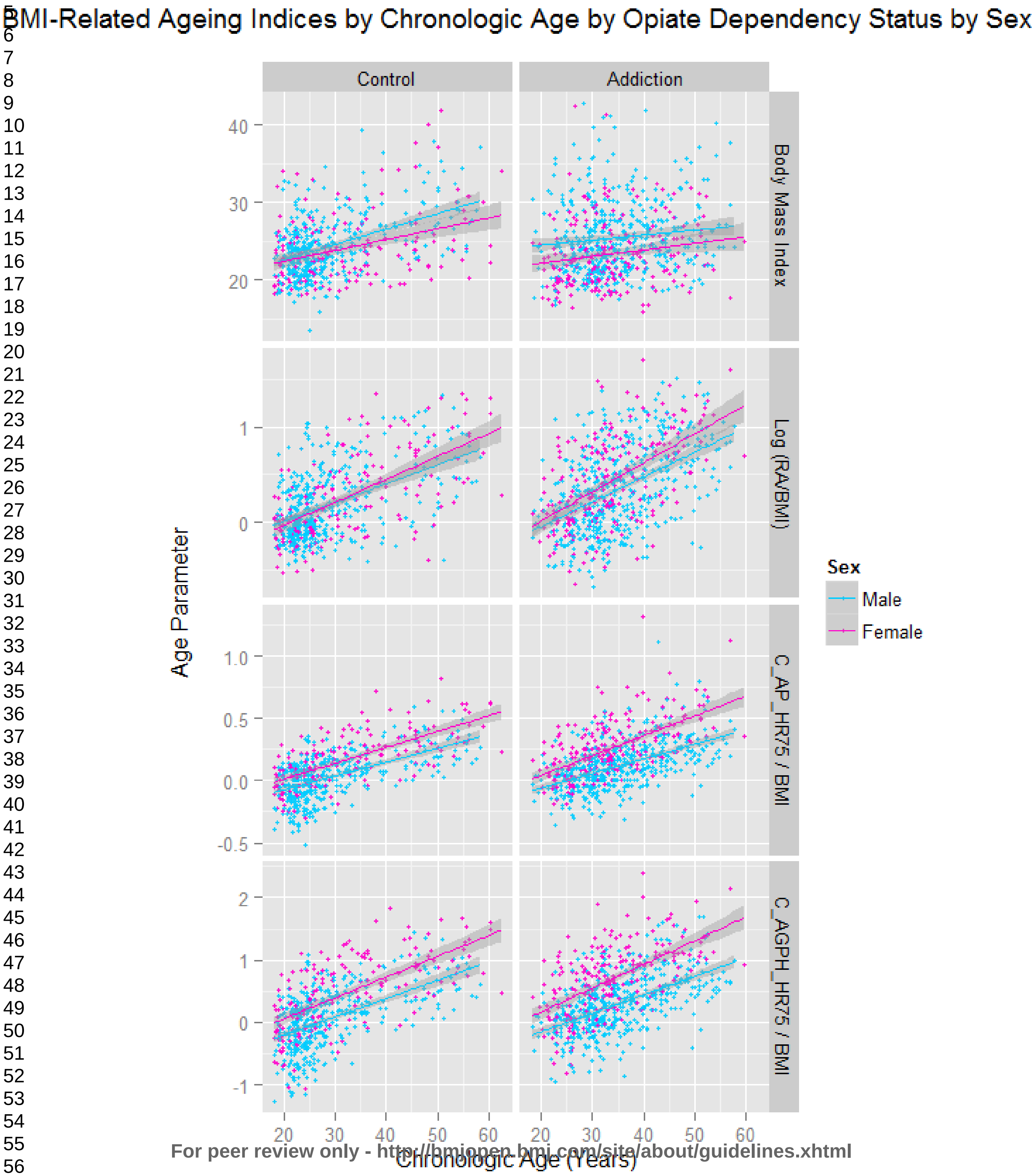
BMI-Related Ageing Indices by Chronologic Age by Opiate Dependency Status by Sex
Control
Addiction
Body Mass Index
Log (RA/BMI)
C_AP_HR75 / BMI
C_AGPH_HR75 / BMI
Age Parameter
Chronologic Age (Years)
Sex
Male
Female
40
30
20
1
0
1.0
0.5
0.0
-0.5
2
1
0
-1
20
30
40
50
60

Figure 7

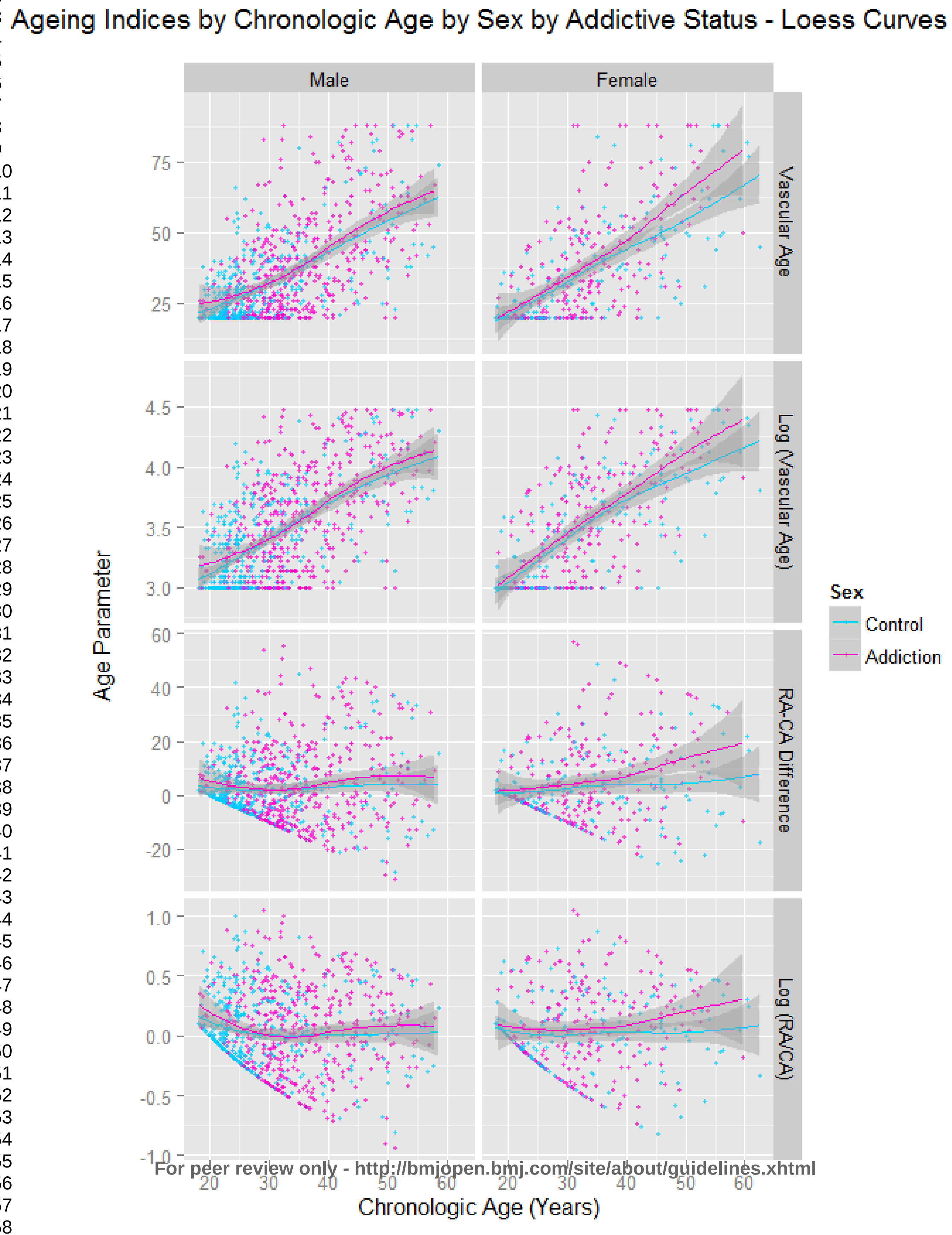
Ageing Indices by Chronologic Age by Sex by Addictive Status - Loess Curves
Male
Female
Vascular Age
Log (Vascular Age)
RA-CA Difference
Log (RA/CA)
Age Parameter
Chronologic Age (Years)
Sex
Control
Addiction

Figure 8

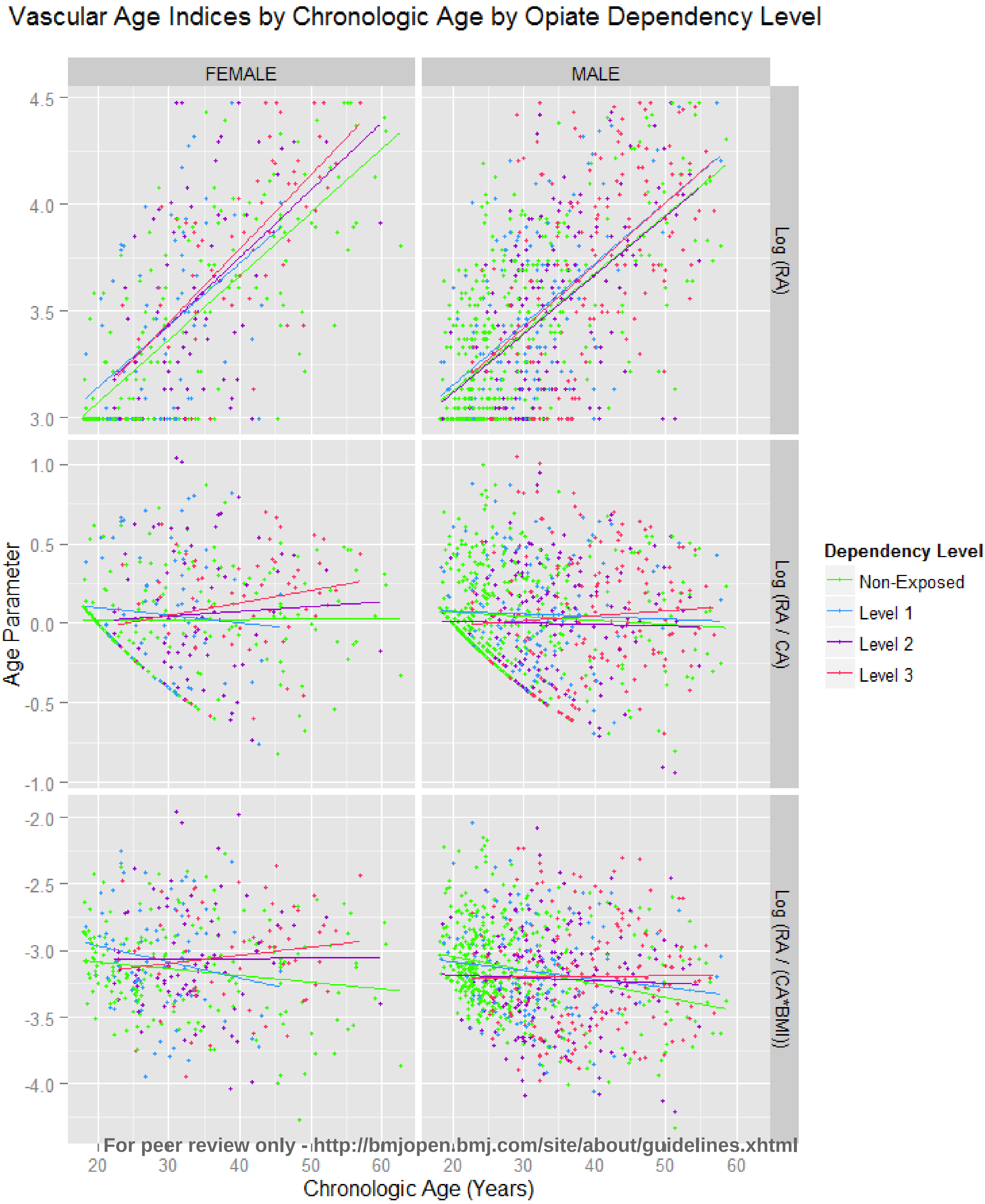
Vascular Age Indices by Chronologic Age by Opiate Dependency Level
FEMALE
MALE
Log (RA)
Log (RA / CA)
Log (RA / (CA*BMI))
Age Parameter
Chronologic Age (Years)
Dependency Level
Non-Exposed
Level 1
Level 2
Level 3

SUPPLEMENTARY TABLES

For peer review only

Supplementary Table 1.: Bivariate Comparisons of

Baseline Sociodemographic, Laboratory and Drug Use Variables

	Control	Opiate Dependence	t-value	df	P
Chronologic_Age (Yrs)	29.96_(0.45)	34.55_(0.32)	-8.262	1082.22	0.0000
Biometrics					
Height_(cm)	173.59_(0.37)	173.51_(0.33)	0.172	1261.00	0.8633
Weight_(kg)	73.21_(0.58)	74.98_(0.63)	-2.071	1260.00	0.0385
BMI_(kg/m^2)	24.25_(0.17)	24.8_(0.17)	-2.263	1256.60	0.0238
Drug Use					
Tobacco (%Smokers)	0.16_(0.02)	0.92_(0.01)	-41.558	1029.42	0.0000
Cigarettes/d	1.78_(0.22)	16.64_(0.37)	-34.982	1089.65	0.0000
Minutes After Smoking	121.98_(8.48)	96.48_(12.98)	1.645	757.57	0.1004
Heroin Dose (g/d)	0_(0)	0.53_(0.02)	-21.717	682.28	0.0000
Heroin Duration (Yr)	0.09_(0.05)	13.01_(0.33)	-38.783	695.26	0.0000
Lifetime Heroin Exposure (g-Yr)	0.03_(0.02)	7.81_(0.65)	-11.987	666.93	0.0000
Buprenorphine Dose (mg/d)	_()	6.98_(0.21)		586.00	
Methadone Dose (mg/d)	_()	63_(4.01)		66.00	
Laboratory Values					
Cholesterol (mmol/l)	4.78_(0.08)	4.45_(0.04)	3.497	781.00	0.0005
Triglyceride (mmol/l)	1.43_(0.08)	1.4_(0.04)	0.439	781.00	0.6608
HDL (mmol/l)	1.31_(0.04)	1.24_(0.02)	1.551	522.00	0.1215
LDL (mmol/l)	2.87_(0.1)	2.47_(0.04)	3.698	119.75	0.0003
ALT (IU/L)	32.8_(2.07)	65.59_(4.04)	-7.214	778.88	0.0000
AST (IU/L)	30.92_(3.9)	48.04_(2.26)	-3.793	318.80	0.0002
Glucose (mmol/l)	5.03_(0.14)	5.49_(0.2)	-1.231	785.00	0.2185
Creatinine (mcmol/l)	78.86_(1.28)	79.75_(0.68)	-0.634	785.00	0.5263
Urea (mmol/l)	5.18_(0.09)	5.12_(0.21)	0.155	783.00	0.8765
Albumin (g/L)	44.23_(0.32)	43.6_(0.2)	1.533	784.00	0.1256
Globulin (g/L)	29.3_(0.26)	31.53_(0.2)	-6.735	421.08	0.0000
CRP (mg/L)	3.43_(0.65)	6.11_(0.42)	-3.459	269.11	0.0006
ESR (mm/hr)	7.85_(0.59)	12.76_(0.5)	-6.374	365.75	0.0000
Lymphocytes (x10^9/ml)	2.32_(0.05)	2.54_(0.04)	-2.754	759.00	0.0060
Monocytes (x10^9/ml)	0.56_(0.02)	0.63_(0.01)	-2.404	629.00	0.0165
Calcium (mmol/l)	2.37_(0.01)	2.37_(0)	0.111	784.00	0.9119
Phosphate (mmol/l)	1.18_(0.02)	1.22_(0.01)	-2.491	334.08	0.0132
HBcAb Positivity Rate	0.17_(0.05)	0.25_(0.02)	-1.514	70.28	0.1344
HCV Ab Positivity Rate	0.52_(0.07)	0.82_(0.02)	-4.161	56.84	0.0001

For peer review only

Supplementary Table 2.:

Bivariate Comparisons of Central and Peripheral Cardiovascular Parameters

	Control	Opiate Dependence	t-value	df	p
**Operator Index**	88.58_(0.26)	88.34_(0.25)	0.683	1261.00	0.4946
Ages					
Chronologic_Age	29.96_(0.45)	34.55_(0.32)	-8.262	1082.22	0.0000
Vascular_Age	32.57_(0.68)	39.12_(0.7)	-6.729	1255.67	0.0000
Log(CA)	3.35_(0.01)	3.51_(0.01)	-10.375	1076.20	0.0000
Log(RA)	3.39_(0.02)	3.57_(0.02)	-7.448	1242.33	0.0000
RA-CA_Difference	2.61_(0.47)	4.57_(0.57)	-2.652	1245.27	0.0081
RA/CA	1.09_(0.01)	1.13_(0.02)	-1.821	1259.62	0.0688
Augmentation					
C_AP_HR75	1.86_(0.21)	3.94_(0.19)	-7.351	1260.00	0.0000
C_AGPH_HR75	4.78_(0.56)	10.17_(0.46)	-7.503	1260.00	0.0000
C_PH	35.29_(0.29)	37.36_(0.28)	-5.123	1261.00	0.0000
C_T1R	154.76_(0.65)	152.42_(0.54)	2.783	1233.00	0.0055
C_AL	8.25_(0.23)	9.14_(0.18)	-3.078	1037.00	0.0021
C_ATI	49.83_(1.6)	57.02_(1.32)	-3.488	1037.00	0.0005
PPAmpRatio	156.97_(0.79)	150.79_(0.74)	5.686	1260.00	0.0000
P_MAX_DPDT	844.69_(7.35)	850.89_(7.16)	-0.601	1261.00	0.5478
Timing					
HR	69.26_(0.47)	69.99_(0.45)	-1.100	1261.00	0.2714
ED	324.74_(0.84)	323.5_(0.82)	1.054	1261.00	0.2923
C_SVI	144.72_(1.29)	142.43_(1.11)	1.356	1261.00	0.1754
C_DTI	3015.42_(16.3)	3023.56_(15.82)	-0.358	1243.62	0.7201
C_TTI	2151.42_(15.84)	2186.99_(15.28)	-1.609	1261.00	0.1079
Pressure					
SP	123.29_(0.47)	123.77_(0.51)	-0.696	1260.87	0.4862
DP	68.6_(0.38)	68.18_(0.38)	0.775	1255.83	0.4383
C_SP	105.16_(0.45)	106.99_(0.46)	-2.836	1254.81	0.0046
C_DP	69.92_(0.38)	69.69_(0.39)	0.432	1256.15	0.6657
C_ESP	92.72_(0.47)	94.54_(0.46)	-2.769	1261.00	0.0057
C_MEANP	86.19_(0.39)	86.93_(0.4)	-1.305	1256.22	0.1920

For peer review only

Supplementary Table 3.:

Cross-sectional Factor Analysis

lm(log (RA/BMI) ~ log(CA)*Addictive Status)

Parameter	Variable Statistics				Model Summaries		
	Estimate	Std. Error	t Value	Pr(>|t|)	F	dF	P
All Patients							
Opiates	-0.58071	0.2615	-2.221	0.0265	184.8	3, 1258	<2.2E-16
CA: Opiates	0.1756	0.0757	2.32	0.0205			
Males							
Opiates	-0.73731	0.3270	-2.255	0.0244	109.1	3, 862	<2.2E-16
CA: Opiates	0.2128	0.0950	2.24	0.0253			
Females							
CA: Opiates	0.0300	0.0109	2.747	0.0063	116.7	2, 393	<2.2E-16

For peer review only

Supplementary Table 4.:

Longitudinal Factor Analysis

lme(log (RA/CA) ~ log(CA)*Days*Addictive Status)

Group / Parameter	Parameter Values					Model Parameters	
	Value	Std.Error	DF	t-value	Pr(>|t|)	AIC	LogLik
All Patients							
CA:Days	0.00015	0.00006	811	2.5762	0.0102	1124.528	-555.2641
Days:Opiates	0.00010	0.00003	811	2.9978	0.0028		
Males							
Days:Control	-0.00001	0.00003	551	-0.2042	0.8383	780.7702	-384.3851
Days:Opiates	0.00005	0.00002	551	2.6199	0.009		
Females							
Days:Opiates	-0.00115	0.00052	256	-2.2160	0.0276	413.4612	-198.7306
CA:Days:Opiates	0.00032	0.00014	256	2.2185	0.0274		

For peer review only

Supplementary Table 5.:

Cross-sectional Factor Analysis

Interactions of Sex with CA.

Variable	Variable Statistics					Model Summaries	
	Parameter	Estimate	Std. Error	z - Value	P, Prob.(>|z|)	LogLik / F	Wald / dF
RA	Male.Sex	0.94149	0.32377	2.908	0.00364	-787.6	813.7
RA	CA: Male.Sex	-0.26694	0.09293	-2.873	0.00407	-787.6	813.7
C_AP_HR75	CA: Male.Sex	-0.87594	0.06461	-13.56	<2E-16	580.8	2, 1259
C_AGPH_HR75	CA: Male.Sex	-2.4701	0.1626	-15.2	<2E-16	75.55	3, 1259
C_PH	Male.Sex	21.987	4.682	4.696	2.94E-06	75.55	2, 1259
C_PH	CA: Male.Sex	-5.63	1.354	-4.158	3.43E-05	75.55	2, 1259
PPAmpRatio	CA: Male.Sex	2.6901	0.2569	10.47	<2E-16	483.6	2, 1259
C_T1R	Male.Sex	9.6358	0.8174	11.79	<2E-16	130.6	2, 1232

All Interactive Models, except for C_T1R
All model P-values < 2.0E-16
Model for RA is tobit (with LogLik and Wald Statistics). All others are standard linear regression models with CA.

For peer review only

Supplementary Table 6.:

Longitudinal Factor Analysis

Interactions of Time, CA and Sex.

Group	Variable Statistics					Model	
**Parameter**	**Value**	**Std.Error**	**DF**	**t-value**	**p-value**	**AIC**	**LogLik**
RA							
CA: Days: Opiates	2.5E-05	9.0E-06	809	2.7328	0.0064	1149.41	-564.7
Days	-0.0005	0.0002	809	-2.5125	0.0122		
CA: Days	0.0001	0.0001	809	2.3973	0.0167		
C_AGPH_HR75							
CA	30.8204	1.6476	804	18.7068	0.0000	14393.8	-7181.9
Days: Male.Sex	-0.0356	0.0117	804	-3.0428	0.0024		
CA: Days: Male.Sex	0.0087	0.0030	804	2.8711	0.0042		
CA: Male.Sex	-5.1991	2.0124	804	-2.5836	0.0100		
Days: Male.Sex: Opiates	0.0335	0.0142	804	2.3631	0.0184		
CA: Days: Female.Sex: Opiates	0.0124	0.0058	804	2.1522	0.0317		
CA: Days: Male.Sex: Opiates	-0.0079	0.0037	804	-2.1274	0.0337		
Days: Female.Sex: Opiates	-0.0440	0.0218	804	-2.0213	0.0436		
C_AP_HR75							
Days: Male.Sex	-0.0198	0.0049	806	-4.0071	0.0001	10866.4	-5420.2
CA: Days: Male.Sex	0.0050	0.0013	806	3.9398	0.0001		
CA: Days: Opiates	0.0005	0.0002	806	2.8924	0.0039		
Days: Female.Sex	-0.0121	0.0045	806	-2.6830	0.0074		
CA: Days: Male.Sex: Opiates	-0.0041	0.0016	806	-2.5760	0.0102		
CA: Days: Female.Sex	0.0030	0.0012	806	2.5498	0.0110		
Days: Male.Sex: Opiates	0.0150	0.0060	806	2.5092	0.0123		
CA: Male.Sex	-2.0296	0.8114	806	-2.5013	0.0126		
C_PH							
Male.Sex	27.1005	5.0888	1198	5.3255	0.0E+00	13297.5	-6633.7
CA: Male.Sex	-7.0598	1.4690	804	-4.8058	0.0E+00		
Days	-0.0306	0.0085	804	-3.6178	0.0003		
CA: Days	0.0080	0.0022	804	3.6326	0.0003		
Days: Male.Sex: Opiates	0.0298	0.0106	804	2.8028	0.0052		
Opiates	13.5294	4.8766	804	2.7743	0.0057		
CA: Days: Male.Sex: Opiates	-0.0079	0.0029	804	-2.6945	0.0072		
CA: Opiates	-3.6162	1.4128	804	-2.5596	0.0107		

For peer review only

Supplementary Table 7.:

Significance Levels for Quartile P Values <0.1

Cross-sectional and Longitudinal Studies

Cross-sectional Studies

Group / Parameter	Parameter Values				Model Parameters		
	Estimate	Std. Error	t Value	Pr(>|t|)	F	dF	P
All Patients							
H.Exposure.Level.3	-0.7103	0.3845	-1.847	0.0650	1.126	7, 1235	0.3437
CA: H.Exposure.Level.3	0.2056	0.1069	1.924	0.0546			
Males							
Females							
CA: H.Exposure.Level.3	0.0265	0.0139	1.911	0.0568	1.193	4, 388	0.3133

Longitudinal Studies

Group / Parameter	Parameter Values					Model Parameters	
	Value	Std.Error	DF	t-value	Pr(>|t|)	AIC	LogLik
All Patients							
Days: H.Exposure.Level.1	0.00013	0.00004	810	2.9797	0.0030	1152.85	-568.42
Days: H.Exposure.Level.2	0.00016	0.00004	810	3.6254	0.0003		
Days: H.Exposure.Level.3	0.00006	0.00004	810	1.7297	0.0841		
Males							
Days: H.Exposure.Level.1	0.00011	0.00005	549	2.2010	0.0281	828.94	-406.47
Days: H.Exposure.Level.2	0.00014	0.00005	549	2.5507	0.0110		
Females							
Days: H.Exposure.Level.1	-0.00176	0.00100	253	-1.7637	0.0790	475.43	-226.71
Days: H.Exposure.Level.3	-0.00203	0.00103	253	-1.9681	0.0501		
Days: CA: H.Exposure.Level.1	0.00050	0.00028	253	1.7653	0.0787		
Days: CA: H.Exposure.Level.3	0.00054	0.00028	253	1.9298	0.0547		

For peer review only

Supplementary Table 8.:

Summary of Longitudinal Models of Quartiles of Opiate Exposure

(lme (Variable/CA)~ Time*Opiate.Exposure.Quartile)

**Parameter**		**Variable Statistics**					**Model**	
**Group**	**Variable**	**Value**	**Std.Error**	**DF**	**t-value**	**P-value**	**AIC**	**LogLik**
RA/CA								
All Patients	Days: H.Exposure.Level.2	0.00007	0.00003	811	2.26807	0.0236	1143.78	-564.89
Males	Days: H.Exposure.Level.2	0.00007	0.00004	550	2.066028	0.0393	813.62	-399.81
Females	-							
C_AGPH_HR75 / CA								
All Patients	Days	0.00004	0.00001	811	3.372907	8E-04	1176.95	-581.48
	H.Exposure.Level.1	0.15146	0.02842	811	5.329696	0E+00		
	H.Exposure.Level.2	0.17167	0.02721	811	6.308965	0E+00		
	H.Exposure.Level.3	0.20433	0.02761	811	7.400001	0E+00		
Males	Days	0.00004	0.00001	550	2.861191	0.0044	645.42	-315.71
	H.Exposure.Level.1	0.14683	0.03182	550	4.614867	0E+00		
	H.Exposure.Level.2	0.15382	0.03017	550	5.099172	0E+00		
	H.Exposure.Level.3	0.21693	0.02999	550	7.233373	0E+00		
Females	Days	0.00004	0.00003	257	1.689176	0.0924	418.64	-202.3216
	H.Exposure.Level.1	0.09204	0.04998	257	1.841536	0.0667		
	H.Exposure.Level.2	0.17788	0.04887	257	3.640107	0.0003		
	H.Exposure.Level.3	0.17765	0.05221	257	3.402395	0.0008		
C_AP_HR75 / CA								
All Patients	Days	0.00002	0.00001	811	4.778954	0E+00	-2674.138	1344.069
	H.Exposure.Level.1	0.05655	0.01070	811	5.284006	0E+00		
	H.Exposure.Level.2	0.06166	0.01024	811	6.019479	0E+00		

For peer review only - http://bmjopen.bmj.com/site/about/guidelines.xhtml

	H.Exposure.Level.3	0.08000	0.01040	811	7.696224	0E+00		
Males	Days	0.00002	0.00001	550	4.104246	0E+00	-1943.355	978.6774
	H.Exposure.Level.1	0.05617	0.01229	550	4.570086	0E+00		
	H.Exposure.Level.2	0.05482	0.01164	550	4.710096	0E+00		
	H.Exposure.Level.3	0.08586	0.01157	550	7.424323	0E+00		
Females	Days	0.00002	0.00001	257	2.369984	0.0185	-806.9698	410.4849
	H.Exposure.Level.1	0.03297	0.01826	257	1.805227	0.0722		
	H.Exposure.Level.2	0.06411	0.01786	257	3.589983	0.0004		
	H.Exposure.Level.3	0.06855	0.01909	257	3.590865	0.0004		
C_PH / CA								
All Patients	Days	-0.00004	0.00001	811	-4.989103	0E+00	-215.7032	114.8516
	H.Exposure.Level.1	-0.01120	0.02041	811	-0.548578	0.5834		
	H.Exposure.Level.2	-0.10747	0.01955	811	-5.497201	0E+00		
	H.Exposure.Level.3	-0.18195	0.01984	811	-9.171153	0E+00		
Males	Days	-0.00004	0.00001	550	-3.439231	0.0006	-151.6348	82.81741
	H.Exposure.Level.1	-0.06511	0.02450	550	-2.657892	0.0081		
	H.Exposure.Level.2	-0.13868	0.02327	550	-5.958834	0E+00		
	H.Exposure.Level.3	-0.22848	0.02315	550	-9.86833	0E+00		
Females	Days	-0.00006	0.00002	257	-3.811813	0.0002	-76.14242	45.07121
	H.Exposure.Level.1	0.12933	0.03421	257	3.78028	0.0002		
	H.Exposure.Level.2	-0.02369	0.03343	257	-0.708426	0.4793		
	H.Exposure.Level.3	-0.06000	0.03567	257	-1.681998	0.0938		

For peer review only

Supplementary Table 9.:

ANOVA Comparisons of Cross-Sectional Models Polynomial in Opiate Duration

In Table 1.

Model	RSS	
m1	84.517	
m2	83.888	
m3	84.014	
m4	84.517	
m1234	83.731	
**Comparison**	**F**	**P-value**
m1 v m2	4.9694	0.02613
m1 v m3	3.9729	0.04665
m1 v m4	-	-
m2 v m3	-	-
m2 v m4	4.9694	0.02613
m3 v m4	3.9729	0.04665
m1234 v m1	1.2372	0.2899
m1234 v m2	0.3093	0.8718
m1234 v m3	0.5559	0.6948
m1234 v m4	1.2372	0.2899

RSS – Residual Sum of Squares
m1 - Opiate Exposure Duration - Linear Model
m2 - Opiate Exposure Duration - Quadratic Model
m3 - Opiate Exposure Duration - Cubic Model
m4 - Opiate Exposure Duration - Quartic Model
m1234 - Opiate Exposure Duration – Combined Linear, Quadratic, Cubic and Quartic Model

For peer review only

Supplementary Table 10.:

Summary of Longitudinal Models Polynomial in Opiate Duration

Model	Variable Statistics					Model	
**Parameter**	**Value**	**Std.Error**	**DF**	**t-value**	**p-value**	**AIC**	**LogLik**
1 Order- Linear							
H.Dose:H.Dura.n	0.0045	0.0013	814	3.5512	0.0004	1068.74	-530.37
2 Order- Quadratic							
H.Dura.n^2	-0.0009	0.0003	809	-3.5503	0.0004	1138.29	-560.15
H.Dura.n: H.Dura.n^2	2.9E-05	0.00000838	809	3.4714	0.0005		
H.Dose:H.Dura.n: H.Dura.n^2	-0.0001	0.00002745	809	-3.2360	0.0013		
H.Dose: H.Dura.n^2	0.0041	0.0014	809	2.9052	0.0038		
H.Dose	0.1659	0.0830	809	1.9994	0.0459		
H.Dose:H.Dura.n	-0.0407	0.0204	809	-1.9982	0.0460		
3 Order - Cubic							
H.Dura.n^3	0.0000	1.2526E-05	811	-3.3596	0.0008	1156.22	-571.11
H.Dose: H.Dura.n^3	0.0001	1.9606E-05	811	4.0694	0.0001		
H.Dura.n: H.Dura.n^3	1.3E-06	3.94E-07	811	3.3670	0.0008		
H.Dose:H.Dura.n: H.Dura.n^3	-2.4E-06	6.14E-07	811	-3.8553	0.0001		
4 Order- Quartic							
H.Dose	0.0924	0.0358	810	2.5811	0.0100	1171.45	-577.73
H.Dura.n	-0.0065	0.0019	810	-3.3983	0.0007		
H.Dose: H.Dura.n^4	1.9E-06	0.0000006	810	3.0901	0.0021		
H.Dura.n: H.Dura.n^4	1.0E-08	0.0E+00	810	2.9797	0.003		
H.Dose:H.Dura.n: H.Dura.n^4	-6.0E-08	0.00000002	810	-3.0810	0.0021		
1, 2 & 4 Order							
H.Dura.n: H.Dura.n^2	-0.0023	0.0008	804	-3.0322	0.0025	1310.95	-641.48
H.Dose:H.Dura.n: H.Dura.n^2	0.0050	0.0017	804	3.0052	0.0027		
H.Dura.n^4	0.0002	0.00005279	804	3.0026	0.0028		
H.Dose: H.Dura.n^2	-0.0270	0.0092	804	-2.9399	0.0034		
H.Dose: H.Dura.n^4	-0.0003	0.0001	804	-2.9034	0.0038		
H.Dura.n: H.Dura.n^4	-4.5E-06	0.00000156	804	-2.8937	0.0039		
H.Dura.n^2	0.0110	0.0039	804	2.8136	0.0050		
H.Dura.n^2 : H.Dura.n^4	5.0E-08	0.00000002	804	2.7676	0.0058		
H.Dose:H.Dura.n: H.Dura.n^4	9.0E-06	0.00000327	804	2.7629	0.0059		
H.Dose: H.Dura.n^2 : H.Dura.n^4	-9.0E-08	0.00000003	804	-2.6261	0.0088		
H.Dose	0.1584	0.0714	804	2.2175	0.0269		

For peer review only

Supplementary Table 11.:

ANOVA Comparison of Longitudinal Models of Table 10

Model	AIC	LogLik
m1	1049.67	-520.83
m2	1040.85	-511.42
m3	1043.21	-514.60
m4	1043.11	-513.56
m124	1041.09	-506.54
**Comparison**	**L.Ratio**	**P-value**
m1 v m2	18.82	0.0021
m1 v m3	12.46	0.0060
m1 v m4	14.55	0.0057
m2 v m3	6.36	0.0416
m2 v m4	4.27	0.0388
m3 v m4	2.09	0.1482
m124 v m1	28.58	0.0015
m124 v m2	9.76	0.0824
m124 v m3	16.12	0.0241
m124 v m4	14.03	0.0294

AIC – Akiane Information Criteria
Log Lik. - Log Likelihood Ratio
L. Ratio - Log Likelihood Ratio
m1 - Opiate Exposure Duration - Linear Model
m2 - Opiate Exposure Duration - Quadratic Model
m3 - Opiate Exposure Duration - Cubic Model
m4 - Opiate Exposure Duration - Quartic Model
m124 - Opiate Exposure Duration – Combined Linear, Quadratic, and Quartic Model

For peer review only

Supplementary Table 12.:

Full Multivariate Analysis of Log (RA/CA) Including All Biochemical Variables

Parameter	Variable Statistics				Model Summaries		
	Estimate	Std. Error	t Value	Pr(>|t|)	F	dF	P
Both Sexes							
H.Dura.n: Height: SP : CRP	0.0003	0.0001	2.835	0.0048	3.030	12, 381	0.00044
H.Dura.n: Height: CRP	-0.0014	0.0005	-2.82	0.0051			
H.Dose: CRP	6.2416	2.3936	2.608	0.0095			
H.Dose: SP : CRP	-1.2903	0.4964	-2.599	0.0097			
H.Dose: Height: Cigs.d: SP	0.0004	0.0001	2.487	0.0133			
H.Dose: Height: Cigs.d	-0.0017	0.0007	-2.432	0.0155			
H.Dose: Cigs.d: SP : CRP	0.5385	0.2346	2.295	0.0223			
H.Dose: Cigs.d: CRP	-2.5285	1.1265	-2.245	0.0254			
H.Dose: Height: Cigs.d: SP : CRP	-0.0029	0.0013	-2.137	0.0332			
H.Dura.n: Cigs.d: SP : CRP	-0.0020	0.0010	-2.103	0.0361			
H.Dose: Height: Cigs.d: CRP	0.0134	0.0065	2.082	0.0380			
H.Dura.n: Cigs.d: CRP	0.0096	0.0046	2.079	0.0383			
Males							
H.Dose: Height: Cigs.d: CRP	-0.0007	0.0002	-3.534	0.0005	3.323	10, 264	0.0004
H.Dose: Cigs.d: SP : CRP	0.0214	0.0066	3.224	0.0014			
H.Dura.n: Height: CRP	-0.0008	0.0003	-2.984	0.0031			
H.Dura.n: Height: SP : CRP	0.0002	0.0001	2.969	0.0033			
H.Dose: Height: CRP	0.0231	0.0081	2.863	0.0045			
H.Dose: Height: SP : CRP	-0.0046	0.0017	-2.723	0.0069			
H.Dose: Height: Cigs.d: SP	0.0004	0.0001	2.716	0.0070			
H.Dose: Height: Cigs.d	-0.0019	0.0007	-2.583	0.0103			
Cigs.d: CRP	0.1028	0.0450	2.284	0.0232			
Cigs.d: SP : CRP	-0.0208	0.0093	-2.236	0.0262			
Females							
Cigs.d: CRP	-0.12299	0.04178	-2.944	0.00392	4.484	2, 116	0.01331
Cigs.d: SP : CRP	0.02583	0.00883	2.926	0.00413			

For peer review only

Suppl. Fig 1: Quartiles Long Logs by Days

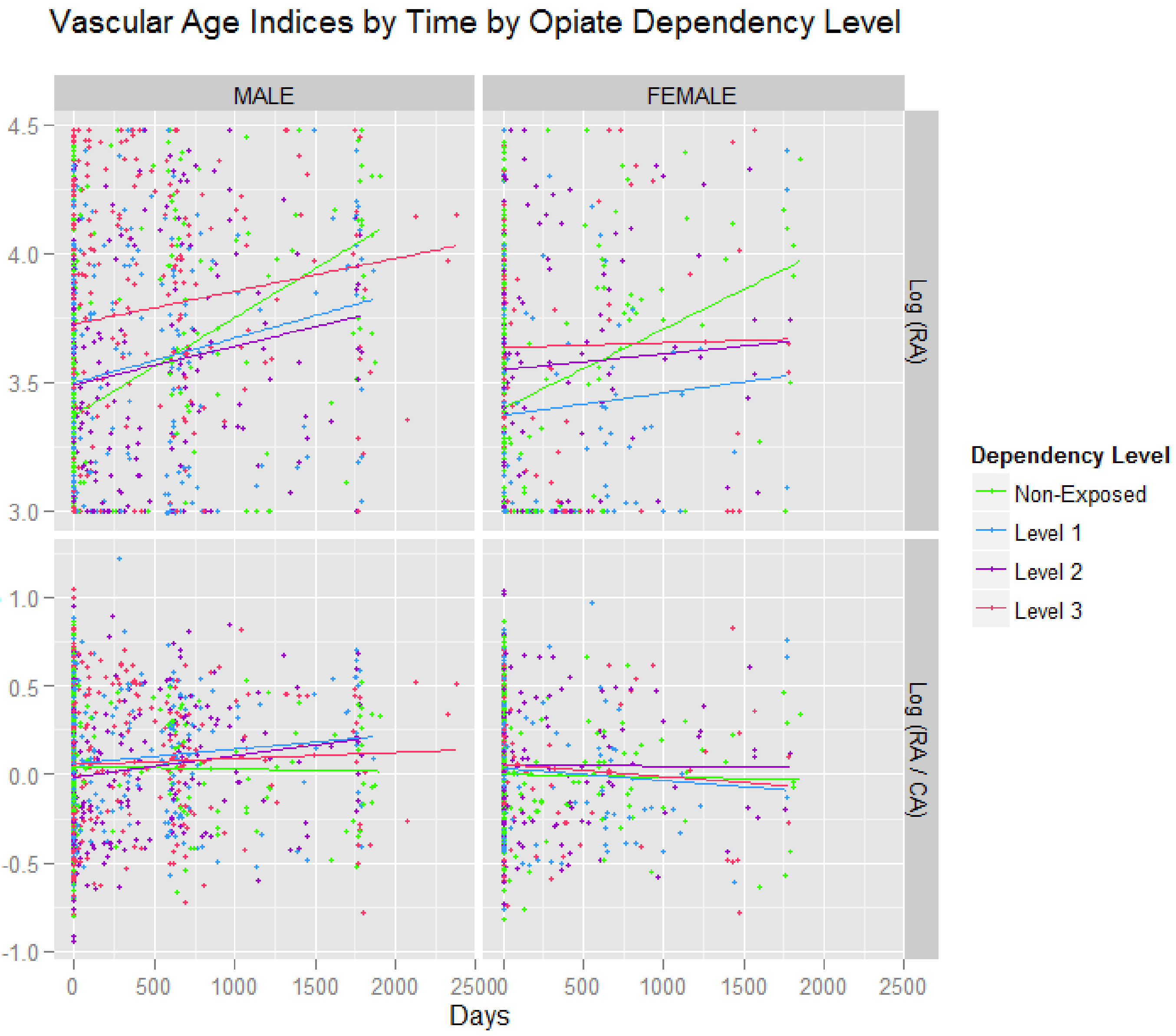

STROBE Statement—checklist of items that should be included in reports of observational studies

DEAR EDITOR,

PLEASE NOTE THAT ALKLK OF THESE HAVE BEEN MET IN OUR ACCOMPANYING ARTILCE.

THANKYOU,

DR STUART REECE.

For peer review only

	Item No	Recommendation
**Title and abstract**	1	(*a*) Indicate the study's design with a commonly used term in the title or the abstract
		(*b*) Provide in the abstract an informative and balanced summary of what was done and what was found
**Introduction**		
Background/rationale	2	Explain the scientific background and rationale for the investigation being reported
Objectives	3	State specific objectives, including any prespecified hypotheses
**Methods**		
Study design	4	Present key elements of study design early in the paper
Setting	5	Describe the setting, locations, and relevant dates, including periods of recruitment, exposure, follow-up, and data collection
Participants	6	(*a*) *Cohort study*—Give the eligibility criteria, and the sources and methods of selection of participants. Describe methods of follow-up<br>*Case-control study*—Give the eligibility criteria, and the sources and methods of case ascertainment and control selection. Give the rationale for the choice of cases and controls<br>*Cross-sectional study*—Give the eligibility criteria, and the sources and methods of selection of participants
		(*b*) *Cohort study*—For matched studies, give matching criteria and number of exposed and unexposed<br>*Case-control study*—For matched studies, give matching criteria and the number of controls per case
Variables	7	Clearly define all outcomes, exposures, predictors, potential confounders, and effect modifiers. Give diagnostic criteria, if applicable
Data sources/ measurement	8*	For each variable of interest, give sources of data and details of methods of assessment (measurement). Describe comparability of assessment methods if there is more than one group
Bias	9	Describe any efforts to address potential sources of bias
Study size	10	Explain how the study size was arrived at
Quantitative variables	11	Explain how quantitative variables were handled in the analyses. If applicable, describe which groupings were chosen and why
Statistical methods	12	(*a*) Describe all statistical methods, including those used to control for confounding
		(*b*) Describe any methods used to examine subgroups and interactions
		(*c*) Explain how missing data were addressed
		(*d*) *Cohort study*—If applicable, explain how loss to follow-up was addressed

For peer review only - http://bmjopen.bmj.com/site/about/guidelines.xhtml

Case-control study—If applicable, explain how matching of cases and controls was addressed

Cross-sectional study—If applicable, describe analytical methods taking account of sampling strategy

(e) Describe any sensitivity analyses

Continued on next page

For peer review only

**Results**		
Participants	13*	(a) Report numbers of individuals at each stage of study—eg numbers potentially eligible, examined for eligibility, confirmed eligible, included in the study, completing follow-up, and analysed
		(b) Give reasons for non-participation at each stage
		(c) Consider use of a flow diagram
Descriptive data	14*	(a) Give characteristics of study participants (eg demographic, clinical, social) and information on exposures and potential confounders
		(b) Indicate number of participants with missing data for each variable of interest
		(c) *Cohort study*—Summarise follow-up time (eg, average and total amount)
Outcome data	15*	*Cohort study*—Report numbers of outcome events or summary measures over time
		Case-control study—Report numbers in each exposure category, or summary measures of exposure
		Cross-sectional study—Report numbers of outcome events or summary measures
Main results	16	(*a*) Give unadjusted estimates and, if applicable, confounder-adjusted estimates and their precision (eg, 95% confidence interval). Make clear which confounders were adjusted for and why they were included
		(*b*) Report category boundaries when continuous variables were categorized
		(*c*) If relevant, consider translating estimates of relative risk into absolute risk for a meaningful time period
Other analyses	17	Report other analyses done—eg analyses of subgroups and interactions, and sensitivity analyses
**Discussion**		
Key results	18	Summarise key results with reference to study objectives
Limitations	19	Discuss limitations of the study, taking into account sources of potential bias or imprecision. Discuss both direction and magnitude of any potential bias
Interpretation	20	Give a cautious overall interpretation of results considering objectives, limitations, multiplicity of analyses, results from similar studies, and other relevant evidence
Generalisability	21	Discuss the generalisability (external validity) of the study results
**Other information**		
Funding	22	Give the source of funding and the role of the funders for the present study and, if applicable, for the original study on which the present article is based

*Give information separately for cases and controls in case-control studies and, if applicable, for exposed and unexposed groups in cohort and cross-sectional studies.

Note: An Explanation and Elaboration article discusses each checklist item and gives methodological background and published examples of transparent reporting. The STROBE checklist is best used in conjunction with this article (freely available on the Web sites of PLoS Medicine at http://www.plosmedicine.org/, Annals of Internal Medicine at http://www.annals.org/, and Epidemiology at http://www.epidem.com/). Information on the STROBE Initiative is available at www.strobe-statement.org.

For peer review only

For peer review only - http://bmjopen.bmj.com/site/about/guidelines.xhtml

BMJ Open

Impact of Lifetime Opioid Exposure on Arterial Stiffness and Vascular Age: Cross-sectional and Longitudinal Studies in Males and Females.

Journal:	*BMJ Open*
Manuscript ID:	bmjopen-2013-004521.R1
Article Type:	Research
Date Submitted by the Author:	19-Feb-2014
Complete List of Authors:	Reece, Albert; University of Western Australia, School of Psychiatry and Clinical Neurosciences<br>Hulse, Gary; University of Western Australia, Psychiatry
**Primary Subject Heading**:	Pharmacology and therapeutics
Secondary Subject Heading:	Cardiovascular medicine
Keywords:	arterial stiffness, opiate dependence, vascular ageing, biological age, acceleration of ageing, heroin

SCHOLARONE™
Manuscripts

Opioid

Impact of Lifetime Opioid Exposure on Arterial Stiffness and Vascular Age: Cross-sectional and Longitudinal Studies in Males and Females.

Short Title:

Opioids as Cardiovascular Risk Factors

Albert Stuart Reece

Gary Kenneth Hulse

School of Psychiatry and Clinical Neurosciences,

University of Western Australia.

Crawley WA 6009, Australia.

*** Correspondence to**:

Albert Stuart Reece

39 Gladstone Rd.,

Highgate Hill,

Brisbane, Queensland, Australia.

Ph: (617) 3844-4000

FAX: (617) 3844-4015

Email: sreece@bigpond.net.au

Word Count: 8,656.

Keywords – arterial stiffness, opioid dependence, vascular ageing, biological age, acceleration of ageing

For peer review only

Structured Abstract

Objective. To characterize and compare the potentiation of arterial stiffness and vascular ageing by opioids in males and females.

Design. Cross-sectional and longitudinal studies of 576 clinical controls and 687 opioid dependent patients (ODP) on 710 and 1,305 occasions over a total of 2,382 days (6.52 years), 2006-2011. Methodology: Radial Pulse Wave Analysis with Atcor SphygmoCor system (Sydney).

Setting. Primary care.

Participants. Controls: General practice patients with non-cardiovascular disorders, and university student controls. ODP: Patients undergoing clinical management of their opioid dependence. Controls had lower chronologic ages (CA) than ODP (30.0±0.5 vs. 34.5±0.3, mean±S.E.M., P<0.0001). Subjects were 69.6% and 67.7% male, and 16.0% and 92.3% were smokers (P<0.0001) for controls and ODP respectively. 86.3%, 10.3% and 3.4% of ODP were treated with buprenorphine (6.98±0.21mg), methadone (63.04±4.01mg) or implant naltrexone respectively. BMI was depressed in ODP.

Interventions. Nil.

Primary Outcome Measures - Vascular Reference Age (RA) and the ratio of the vascular age to the chronological age (RA/CA). Secondary outcome measures: Arterial Stiffness including Augmentation Index.

Results. After Body Mass Index -adjustment, RA in ODP was higher as a function of CA and of time (both P<0.05). Modelled mean RA in control and ODP males was 35.6 and 36.3 years (+1.97%), and in females 34.5 and 39.2 years (+13.43%). Changes in RA and major arterial stiffness indices were worse with CA in females (P<0.01). Quadratic, cubic and quartic functions of opioid exposure duration out-performed linear models with RA/CA over CA and over time. The opioid dose-response relationship persisted longitudinally after multiple adjustment from P=0.0013 in males and P=0.0073 in females.

For peer review only

Conclusion. Data show that lifetime opioid exposure is an interactive cardiovascular risk factor, particularly in females, is related to linear, quadratic, cubic and quartic functions of treatment duration and is consistent with other literature of accelerated ageing in OD patients.

For peer review only

Article Summary

Article Focus

- Despite striking consistency between several large epidemiological studies of the cardiovascular toxicity of long term opioid dependence, the subject is not well studied nor widely understood.

- There are only two (contradictory) studies of gender differences in significantly exposed populations in the published literature.

- The implications of organism-wide xenobiotic-induced inflammation have not been incorporated into the clinical practice of addiction medicine, our understanding of the inflammatory basis of atherogenesis, or the fact that vascular ageing is a major surrogate for systemic ageing across the whole organism.

Key Messages

- Opioid dependent patients (ODP) had and advance of mean vascular age from control of 35.61 to 36.31 years in males and 34.54 to 39.18 years in females which, after adjustment for BMI, were significant in both males and females cross-sectionally and longitudinally (all $P<0.05$). Both the acceleration of vascular ageing and arterial stiffness were worse in females ($P<0.01$) where it occurred 12 years earlier (29 vs 41 years).

- A dose-response relationship between lifetime opioid exposure and measures of arterial stiffness and vascular age was demonstrated in both sexes, which was robust to multiple adjustment for known cardiovascular risk factors.

- Use of quadratic, cubic and quartic functions of opioid duration exposure significantly improved the statistical fit of models of the ratio of vascular age (RA) to chronological age (CA, RA/CA ratio), thereby further highlighting the significance of the duration for which opioid agonism is administered.

For peer review only

Strengths and Limitations

- With 576 controls and 687 OPD the large size of this study was a signficant advantage, as was its combined cross-sectional and longitudinal design, its ability to control for many cardiovascular risk factors, the sex specific analysis, and the diverse output of cardiovascular parameters of the Atcor / SphygmoCor Pulse Wave Analysis technology employed.

- The use of detailed statistical modelling of relationships between factors, dose-response modelling, polynomial modelling in opioid exposure duration, inter-quartile trend effects, and adjustment for known cardiovascular risk factors added further analytical power to the study.

- As the major study limitation was its lack of other measures of organism ageing and its lack of mechanistic and ageing medicine-specific biomarkers, further studies incorporating these refinements are indicated.

For peer review only

Introduction

Opioid dependence is a condition of increasing public health importance.

Opioid Dependence is associated with an impressive array of traditional and novel cardiovascular risk factors and associations including hypertension [1], hyperlipidaemia [2-3], tobacco consumption, increased body weight [4], hyperglycaemia [3], hyperfibrinogenaemia [3], diffuse polyclonal gammopathy and immune stimulation [5-6], elevated high sensitivity CRP [7], elevated cytokines [8], relative hypercalcaemia and hyperphosphataemia [9] and reduced circulating endothelial progenitor cells [10]. Furthermore opioid dependence has been associated with atherosclerotic disease in both the coronary [11-18] and cerebral [14 18] circulations. In a 33-year review of an opioid maintenance program in California in 2004, death from cardiovascular disease was noted to account for three times as many years of life lost in the treatment group than in the general population [18]. In 2007 an Iranian surgical group identified that opium dependent patients were at increased risk of requiring open coronary revascularization with an odds ratio of 1.8 after adjustment for other risk factors [15]. Moreover in this series there was a dose-response relationship between the amount of opioids consumed and the severity of the coronary disease. It was also shown that the coronary disease occurred two years earlier in opioid dependent persons than in controls [16]. This group further identified that opium use was the strongest cardiovascular risk factor in their population of men, with effects greater than traditional risk factors [17]. The sex specific finding in this study was felt to be related only to a reduced prevalence of opium consumption amongst females.

In reviewing over 800 fatal cases of opioid toxicity in Australia in 2006 Darke et al. found that 17% of decedents over the age of 44 years had coronary stenoses in excess of 75% [12]. Similarly when comparing 1193 post mortem cases of heroin and methadone toxicity the Sydney group found 2-3 times elevated adjusted odds ratios of ventricular hypertrophy, and myocardial, interstitial and perivascular fibrosis in the methadone treated group, consistent with the effects of more consistent opioid agonism in such patients [11]. In a 21-year follow-up of 42,676 patients undergoing maintenance opioid agonist treatment in Australia, including 425,998 person-years, Degenhardt et al. identified cardiovascular death as occurring 2.2

For peer review only

times as often as in the wider community [13]. Indeed in this study elevated rates of death with severe organ dysfunction were noted which appeared to act as a backdrop to the very high rates of death by overdose generally reported in such cohorts (see Web Appendix 6 of this citation).

In 2012, a prospective population based study of 50,045 people including 234,098 person-years reported hazard ratios of death from coronary artery disease (CAD) of 1.9-fold (95%C.I. 1.57-2.29) and of cerebrovascular disease (CeVD) of 1.68-fold (C.I. 1.29-2.18) [14]. Both were worse in females (CAD: male adjusted HR (AHR) 1.58, female AHR 2.90; CeVD male AHR 1.49, female AHR 1.97). An earlier study from Iran did not confirm the cardiovascular salience of opioid exposure amongst females as compared to males, but the number of exposed females in this study was small, which likely compromised the power of the analysis to detect an efffect [17]. Furthermore recent reports of 1,263 patients found elevated rates of vascular age in patients treated with methadone compared to other agents [19] and improved vascular ages accompanying the long term drug-free condition [20].

One large case control study found a dose response relationship between opium exposure and the severity of angiographically assessed coronary artery disease in a diabetic population [21].

Moreover opioid patients dependent on short acting opioids such as morphine, oxycodone and heroin undergo the clinical syndrome of opioid withdrawal several times daily. This syndrome is a hyper-adrenergic state characterized by neurological and cardiovascular stimulation which in its more severe manifestations includes hypertension and tachycardia [22] and a reduction of subendocardial perfusion [23]. This negative aversive experience is one of the key reasons many opioid dependent patients (ODP) continue to use narcotics.

Important as the above collected findings are, other evidence suggests that the impact of elevated vascular age has implications on the health and ageing of the organism as a whole beyond those limited primarily to the cardiovascular system. It has been repeatedly proposed that vascular ageing is a principal component of biological organismal ageing, by virtue of the contribution made by cardiovascular causes to the overall death rate in developed nations, and also the importance of the vascular niche common to many stem cell beds [24-25]. This view closely accords with the finding in many of the above studies and particularly that by Degenhardt et al [13] that elevated rates of overdose occur on the background of advanced

For peer review only

disease in other organs [1 12 18]. Opioid dependent patients have many stigmata of accelerated ageing including increased rates of: hair greying, erosion of alveolar bone from chronic periodontitis [26], osteoporosis [27], chronic respiratory disease [11 13 18], chronic renal disease [11], diffuse senescent-mimetic immune stimulation [5-6 28], diabetes [3], neuropsychiatric disorders, multi-system disease [11-12], reduced circulating stem cell counts [10], and many malignancies [13 29]. Thus the call has been made for geriatricians to be appointed to the care of opioid dependent patients beyond the age of 50 years, due to the high prevalence of multi-system disease [1].

The possibility therefore arises that an improved understanding of the increased and accelerated profile of degenerative disease seen in opioid dependent patients may enhance our understanding, and in time our management, of age related disorders in the rest of the community.

As this clinic sees both opioid dependent patients and the general community, we were ideally situated to compare these two groups. The availability of the mobile SphygmoCor system from Atcor in Sydney means that a host of central cardiovascular data is available to the clinician in a non-invasive and rapid manner, providing quantitation of important subclinical endophenotypes of central vascular stiffness and cardiovascular and therefore organismal ageing. The central investigative question to be prospectively assessed was whether there were changes in major central cardiovascular parameters in the opioid dependent group, and if so are they robust to adjustment for other confounding factors. Several earlier papers from our group have considered this issue in both cross-sectional [30-31] and longitudinal [32-33] studies in each sex separately and drawn conclusions generally confirmatory of this hypothesis. It has been shown that the type of opioid pharmacotherapy affects the severity of opioid related potentiation of arterial stiffness [19], that induction of the drug free state after implantable opioid antagonists reverses this finding [20], and that opioid withdrawal is also associated with elevation of cardiovascular age and arterial stiffness parameters [23]. However a formal comparison of the effects by gender has not been possible in these earlier reports as they primarily considered single sex data. Gender comparison therefore forms the major focus of the present report which is a compilation of much of this earlier data.

Methods

For peer review only

Patient Selection. Control subjects (N=576) were recruited opportunistically from patients presenting for health examination checks for insurance or employment medical examinations or from patients presenting with minor health problems such as ear blockages, minor psychological disorders, or University students. Patients with known cardiovascular disorders such as hypertension, renal disease or diabetes or diseases known to perturb the CVS subacutely such as infections or pregnancy, were excluded. Opioid dependent patients (N=687) were also recruited opportunistically and were under treatment with either methadone (N=71), buprenorphine (N=593) maintenance, or naltrexone implant (N=23). All were sampled opportunistically at the time of their clinic visits May 2006 - December 2012.

Patient Treatment. Opioid dependent patients maintained on, methadone or buprenorphine were managed by accepted clinical algorithms by their usual health providers for their drug dependency. The naltrexone implants used for the management of heroin dependence were manufactured by “Go Medical” industries in Perth, Western Australia under Commonwealth of Australia Therapeutic Goods Administration (TGA) GMP conditions and administered under the compassionate Access arrangements of the Special Access Scheme authorized by the TGA as described elsewhere [34]. Patients were recalled for re-testing at two and five years post baseline assessment, and assessed opportunistically. Any patient with a known chronic or subacute cardiovascular condition were excluded from participation. Frequency and quantity of substance use by substance type (inclusive heroin) was assessed at the initial time and subsequently. Other opioids were converted into morphine equivalents, and then into heroin equivalents at the rate of 1g of street heroin = 0.5g morphine. The duration of opioid use was counted from the time of first use. If it became known that alcohol had been consumed in the few hours prior to the test or that stimulants had been used in the three days prior, that day’s studies were excluded.

PWA Studies. Patients were positioned supine for the performance of the test and allowed to rest for five minutes prior to the test. Consumption of food, drink, alcohol or tobacco was not restricted, but patients were not allowed either to sleep or talk during the testing procedure. Pulse wave analysis by applanation tonometry was performed on the right radial artery unless it was unavailable (due to trauma, surgery or congenital causes). The brachial blood pressure

For peer review only

was taken in the opposite arm using the oscillometric Omron HEM 907 device. The data was collected and analyzed by the SphygmoCor software. Patients were studied in quintuplicate, and adequate studies were averaged for that day. Acceptable studies had an Operator index of greater than 70%, and were not inconclusive. Details of patients' prior drug use was also taken at the time of the study performance and entered into the database. Time in days was measured from the time of the first PWA study.

The SphygmoCor software used for the PWA studies calculates a number of major indices including Vascular Reference Age (RA), Chronologic Age (CA), Central Augmentation Pressure at Heart Rate 75 (C_AP_HR75) the Central Augmentation Pressure / Pulse Height Ratio at Heart Rate 75 (C_AGPH_HR75) also known as the Augmentation Index, Central Pulse Height (C_PH), Central Augmentation Load (C_AL), Central Augmentation Time Index (C_ATI), Peripheral–Central Pulse Pressure Amplification Ratio (PPAmpRatio), Maximal Peripheral Rise of dP/dT (P_MAX_DPDT), Central Systolic Pressure (C_SP), Central Diastolic Pressure (C_DP), Central End Systolic Pressure (C_ESP), Central Mean Pressure (C_MEANP), the Central Diastolic Time Index (C_DTI), the Central Tension Time index (C_TTI), and an index of subendocardial perfusion known variously as the Subendocardial Perfusion Ratio (SEVR), the Central Stroke Volume Index (C_SVI) or the Buckberg ratio defined as the C_TTI / C_DTI.

Statistics. Data are presented as the mean ± S.E.M. Categorical data were studied using "EpiInfo" 7.0.8.3 using the Chi squared test obtained from the CDC Atlanta, Georgia or the Fisher Exact test if the numbers in a cell were less than 20. Continuous categorized data were analyzed in "Statistica" 7.1 from Statsoft, Oklahoma using Student's t-tests. T-tests with separate variances were used where Levene's test was significant. Continuous variables such as RA, CA, RA/CA, variously corrected RA, SP, Weight, HDL, BMI, and systolic pressure were log transformed in accordance with normality assumptions based on the Shapiro-Wilks test. High sensitivity CRP and the heroin dose were arcsinh transformed which is a similar transform to logarithmic, but accepts arguments of zero. Time (as days) was measured from the time of the initial PWA study. The time since the last cigarette was recorded and analyzed as a factor with cut-off points at 30, 60, 120, 180 and > 180minutes, and a non-smoking category. For some analyses lifetime heroin exposure was cut into quartile

For peer review only

categories with divisions at 0, 0.01, 2.49, 6.59 and 350 g-years respectively, yielding 583, 207, 226 and 227 patients in each group respectively. Multiple regression was performed in "R" 2.15.2 obtained from the University of Melbourne Central "R" Archive Network mirror. Mean model estimates for various groups were prepared by inserting the mean parameter values into the untransformed final models. Multiple linear regression of truncated variables such as RA (minimum value of 20, maximum value of >80) were analyzed using tobit multiple regression techniques from the Applied Econometrics library in "R" (AER). RA's of individual studies returned as >80 were assigned as 88. Time-dependent repeated measures analysis was performed using non-linear mixed effects (nlme) regression module in "R" using restricted equation maximum likelihood (REML) techniques. Random effects were assigned to unity and the patient identification code. When nlme models were compared they were converted to maximum likelihood (ML) methods to allow Anova testing. In each case model reduction was performed manually by the classical method involving removing the least significant term. The log-likelihood value was abbreviated to LogLik. Graphs were prepared with reshape2 and ggplot2. Where loess curves were fitted, the span used was 0.95. T-tests were two-sided. P<0.05 was considered significant.

Ethical Approval. All patients provided their informed consent to participate in the study and for their medical treatment. The study was approved by the Human Research Committee of the Southcity Family Medical Centre, Brisbane, Queensland, Australia, which is registered with the National Health and Medical Research Council.

For peer review only

Results

576 control patients were compared with 687 opioid dependent patients. The mean ages were 30.0±0.5 and 34.5±0.3 (mean ± S.E.M.) years (t=-8.262, dF=1082.22, P<0.0001, Supplementary Table 1), with 401 (69.62%) and 465(67.69%) males respectively (Chi^2 = 0.54, P=0.46). 15.97% and 92.29% smoked tobacco (Chi^2=746.56, P<0.0001). The mean CA of the whole group was 32.5±0.3years. The mean CA of the males was 32.1±0.3 and of females 33.2±0.5 years. 7.47% and 5.97% of the control and opiate dependent groups were aged over 50 years respectively (Chi Squ.=1.13, P = 0.29). Amongst the males these ratios were 5.74% and 6.24% (Chi Squ. =0.1), and amongst the females they were 11.43% and 5.41% (Chi Squ. = 4.79, P = 0.029, Fisher Exact Test 0.19-0.98; pooled data, Corr. Chi Squ = 3.8, P = 0.051). Racial stratification was not performed as our population is around 90% Caucasian in background. Other baseline biometric, drug use and laboratory values are given in Supplementary Table 1. Patients were studied longitudinally on 710 and 1,305 occasions. The period of repeat study, measured from the time of the first PWA study, was 624.11 ± 18.74 days (range 1-2382 days; 495 (60.36%) studies performed beyond 360 days). Details of the numbers of repeat studies are given in Supplementary Table 2.

Comparative PWA values are given in Supplementary Table 3. Most augmentation values and the C_SP and C_ESP are noted to be higher in the opioid dependent group consistent with their being an average of 4.59 years older.

Figure 1 shows different vascular age and augmentation parameters corrected for various features, against CA. This figure shows that the BMI was lower in the opioid dependent group than the controls both as a factor (factor est.=0.0719, est. S.E. = 0.0327, t=2.197, P=0.0282) and in interaction with CA (BMI: CA est.=-0.0023, est. S.E. = 0.0009, t=-2.350, P=0.0189; model F=34.94, dF=3, 1258, P<2.2E-16). In the longitudinal series BMI was lower as a factor (est.=-0.0174, dF=812, P=0.0470; model AIC= -3722.55, LogLik=1867.27). This result may be the effect of cumulative diversion of resources over time. Figure 2 shows similar indices over time. Supplementary Table 4 presents the results of the cross-sectional data where BMI corrected RA is regressed against CA and addictive status. In the whole group both opioid dependency status (P=0.0265) and the CA: addiction interaction (P=0.0205) are significant. Similar results were found in each sex. Similar results were also found in the longitudinal study when the RA/CA ratio was regressed against CA, time and

For peer review only

dependency status both for opioid dependency as a factor (P=0.0102) and in interaction with CA (P=0.0028; details in Supplementary Table 4).

Inclusion of tobacco exposure along with opioid exposure in these models did not alter these findings (data not shown).

As shown in Supplementary Figures 1 and 2, these changes were found to be more pronounced with CA in females. These data are presented corrected for the changes in BMI in Figures 3 and 4. Details for these significant differences are presented in Table 1.

Figure 5 shows these data by sex with loess curves fitted (span=0.95). This strongly suggests that an inflection point is reached about the age of 30 in females, which is not seen in males until about 40 years of age. This was quantified by dichotomizing CA from age 41 in males and 29 in females. As shown in Table 2 in males in the older age category, the addictive status and their interaction were significant (P= 0.0010, 0.0021 and 0.0008 respectively), whilst in females the older age: addiction interaction was significant (P=0.0195, analysis restricted to women <55 years).

The regression changes are quantified for some of the major indices of arterial stiffness in cross-sectional data in Supplementary Table 6 (all P<0.005). Similar significantly different sex specific data were found in the longitudinal dataset when the RA was regressed against CA, time, sex and addictive status (most P<0.02, Supplementary Table 7).

When the mean sex-specific values for CA and BMI are inserted into the equation of Table 3 the mean vascular ages of the control groups amongst all patients, males and females was 35.42, 35.61 and 34.45 years, whilst the corresponding ages of the ODP's was 37.24, 36.31 and 39.18 years, representing incremental gains of 5.14%, 1.97% and 13.43%.

When quartiles are defined for lifetime heroin exposure the results shown in Figure 8 are found. Similar effects longitudinally are shown in Supplementary Figure 3. These results are quantitated in Supplementary Table 8 for both cross-sectional and longitudinal data compared to non-exposed controls. In the cross-sectional analysis the RA/CA ratio was regressed against the CA and Opioid exposure quartile. In the longitudinal analysis the RA/CA ratio was regressed against time, CA and the opioid exposure quartile. Whilst the CA: Level 3

For peer review only

opioid exposure interaction is of borderline significance in all patients and females (P=0.0546 and 0.0568, but not in males) in the longitudinal study the age: level 2 exposure is significant in all patients and males (P=0.0003 and 0.0110) and level 3 opioid exposure approaches significance in females both as a factor and in interaction with age (P=0.0501 and 0.0547). Supplementary Table 9 presents the results of a longitudinal regression of age-corrected indices of cardiovascular stiffness against time and opioid exposure quartile by gender, and notes a highly significant effect by exposure quartile in both genders.

The data was formally interrogated for a trend between the different quartiles of lifetime opioid exposure using the contrasts polynomial function in “R”. Significant contrasts between the various quartiles for lifetime opioid exposure for the age: opioid exposure interaction was indeed demonstrated in all patients (est. = 0.1657, S.E. = 0.0787, t = 2.104, P = 0.0356; Model Adj. R^2 = 0.4265, F = 133.0, dF = 7, 1235, P < 2.2×10^{-16}), such that moving up a quartile level at age 60 increased the modelled RA by 8.42% to 65.051 years, and movement from the first (non-exposed) quartile to the highest (level 3) quartile increased the modelled RA 25.26% to 75.154 years.

Some environmental exposure – pathology relationships have been shown to be power functions of the duration of exposure and are therefore particularly sensitive to the periods for which exposure occurs [35]. Since opioid administration is often prescribed and recommended as being of indefinite duration this is particularly relevant to the present discussion. Therefore a detailed study of the power relationships of the duration of opioid administration was undertaken. Table 3 shows that simple regression models of RA/CA against the dose-duration interaction were significantly related to linear, quadratic, cubic and quartic terms in opioid exposure duration. Moreover when RA/CA was regressed against a model including interactive terms in linear, quadratic, cubic and quartic terms, all four were significant. When these models were compared by ANOVA studies the quadratic (AIC=82.888) was noted to be significantly better than the linear, cubic or quartic models (Anova tests, all P<0.05), but not significantly different from the combined model (AIC=83.731, P=0.87; Supplementary Table 10).

When a similar exercise was performed in the longitudinal dataset, similar results were obtained (Supplementary Table 11). In this study RA/CA was regressed against time and the opioid exposure quartile. As a combined linear-quadratic-cubic-quartic model failed to

For peer review only

converge, the highest order model used was linear-quadratic-quartic. Again quadratic and quartic terms in exposure duration persist in the final model. In this case the quadratic model (AIC=1040.85) was significantly superior to all the others but was not significantly better than the linear-quadratic-quartic model (AIC= 1041.09, P=0.082; Supplementary Table 12).

The data was interrogated for the presence of a dose-response relationship directly by examining continuous terms in opioid dose and duration regressed against the RA/CA ratio in both datasets. As shown in Table 4 the dose-duration interaction is significant in all patients considered together, in males and in females (P=0.000037, 0.00029 and 0.0236) in the cross-sectional dataset, whilst in the longitudinal dataset the parallel results are P = 0.0004, 0.0008 and 0.0127.

The final step of the analysis was to consider whether the significance of the opioid dose and duration might persist after adjustment for all known cardiovascular risk factors. In preliminary analyses lipidic factors were invariably eliminated during the model reduction process. Moreover, as discussed in the Introduction, opioids themselves are likely to perturb both lipid function, and also have a robust immunostimulatory action. Therefore including immune markers is likely to over-correct the model. Similarly when the time since the last cigarette consumption was included in the cross-sectional and longitudinal models it was eliminated during model reduction. For these reasons the final cross-sectional formula used regressed RA/CA against interactive terms in opioid dose and duration, height, tobacco consumption, systolic blood pressure and weight. The longitudinal model was similar but had an additive term in weight. These were the terms which remained significant in the models in preliminary exploratory analyses for log (RA). Further consideration of the clinical justification for the choice of this five way interaction is included in the Discussion section.

The results shown in Table 5 were found for the cross-sectional data. This table shows that significant interactions including opioid dose-duration terms are found in all patients (P=0.0261) and in males (P=0.0017; Adj. R^2=0.0815), whilst in females this interaction approaches significance (P=0.0516). When a similar exercise is performed on the longitudinal data, significant interactions are seen from P = 0.0011, 0.0013 and 0.0073 and above in each group respectively (Table 6).

For peer review only

Supplementary Table 13 presents the results of final models in which complete multiple regression was performed in the cross-sectional dataset for all pertinent variables. (Log) RA/CA was regressed against interactive terms in opioid dose and duration, height, tobacco use, systolic pressure and high sensitivity CRP together with additive terms in weight, cholesterol and HDL. Opioid dose and duration persist in the final model in males and in all patients, but not in females. Interestingly CRP features in most of the terms of these final models.

For peer review only

Discussion

The major findings of this study are that indices of vascular age and arterial stiffness are worse in opioid dependence compare to opioid naïve controls with mean calculated ages elevated by 1.97% in males and 13.43% in females. The effect was thus more marked in females. A significant effect was found on both vascular age and augmentation index by exposure quartile after correction for CA and BMI. The RA/CA ratio was found to be related to power functions of the opioid duration of exposure in both cross-sectional and longitudinal analyses. A dose-response relationship was demonstrated with lifetime opioid exposure. And the effect of opioid exposure was robust, and remained after multiple adjustment in cross-sectional and longitudinal studies.

These findings should be interpreted in the light of the relatively modest degree of opioid exposure to which these patients were exposed. Whilst the dose and duration of opioids used by patients in this study is typical of that seen in many published series, the treatment of these patients was mostly with the relatively mild partial agonist buprenorphine, with only modest doses employed (mean 6.98±0.21mg): this dose level being much lower than the commonly recommended (18-24mg) [36]. Moreover buprenorphine has been shown to be more mild in its cardiovascular effects than the full agonist agent methadone . For this reason the results reported herein should be seen as a lower bound on the effects which may be observed.

The findings in relation to central vascular stiffness and age being related to power functions of the treatment duration is of great importance. Chronic opioid agonist treatment is often seen as relatively benign and partly for this reason is frequently recommended for very long term or indefinite administration [37-38]. However the demonstration here that the effects likely compound with time have major clinical practice implications, and have not been previously noted. The 1.97% effect described here for males, compounded over 30 years equates cumulatively to 179.5% gain and in diminution to 44.95% loss. Similarly the 13.43% gain seen in females compounded over 30 years is a 4349.02% gain and a 98.66% loss in reduction. The effects therefore of these process maintained over the long term on stem cell diminution or immune stimulation are potentially very considerable.

The exacerbation of the effect in females was clearly demonstrated in Figures 3-7, Tables 1 and 2, and Supplementary Tables 4 and 5. This finding has, to the authors knowledge, only

For peer review only

been reported previously by an Iranian group [14]. If one looks closely at Figure 7 several fascinating findings emerge. The vascular age algorithm used by the SphygmoCor software is based upon the augmentation index C_AGPH_HR75. This index has a clear curvilinear convex upward relationship to CA as shown in Figure 7 in the lower panels, unlike the C_AP_HR75 which is directly linear [39]. Hence the reduction at the higher ages on the C_AGPH_HR75 curve may be somewhat artefactual. If one looks at the upper panels of Figure 7 the rise in RA with age in ODP may be seen to be broadly exponential in form, if the decline in slope above age 45 years is discounted. In females it appears that the rise in RA occurs some 10 years prior to that seen in males, a finding emphasized by the middle panels. This fits with a dramatic clinical transformation which is seen in opioid dependent females at around age 30, and in males at about age 40. Whilst the cause of this is unknown it may possibly be related to a more aggressive immune system in females which is in part hormonally mediated [40].

Some consideration of the comparability of the control and opiate dependent groups is appropriate. Significant differences were found between the control and opiate dependent groups in relation to chronological age, weight, BMI, smoking, cholesterol, LDL and hepatic and inflammatory laboratory markers, but these were controlled for by various the multiple regression techniques. The higher mean weight in the opiate dependent group may have been related in part to their higher age, and in part to the orexigenic effects of opiate agonism [41], whilst serial resource diversion may explain the slower rate of gain of weight in this group over time. Higher rates of tobacco consumption may be related to either the common features of the addictive lifestyle or the pro-consumptive effects of opioid aalogies [42]. Significantly more of the females in the control group (11.4% vs. 5.41%) were aged over 50 years (P=0.029). However as no data was collected relating to hormonal status with regard to the menopause it is not possible to remark on the relative hormonal status of the two groups. Opiate dependency is well known to be associated with hypothalamic hypogonadotrophic oligoamenorrhoea, so that the age-related hormonal suppression in the control group was somewhat balanced by the drug related hormonal suppression in the opiate dependent group. Clarification of this issue must await further studies.

Some consideration is appropriate of the clinical justification for the five-way interaction which was presented in the final multiple regression models of RA (Tables 5 and 6). Height is a key variable which relates to many techniques of measuring of arterial stiffness since it is

For peer review only

related to the timing of arterial wave reflections, and it is hardly surprising that this remains in the final model [43-44]. Height and weight will naturally interact and indeed the BMI is one formula which formalizes such interactions. The opioid dose-duration exposure is a standard way in which to account for lifetime opioid exposure, and has been used by WHO [35] and NIH [14] investigators amongst others. Other factors including laboratory parameters were not present in the final models. Therefore it is not surprising that this cluster of factors remains in the final model.

On the other hand CRP was prominent and present in most terms in the final model when RA/CA was interrogated by multiple regression (Supplementary Table 13) suggesting that innate immune processes may be implicated in the observed opioid-vasculopathy effect. The age-related course of high sensitivity CRP has been described in several earlier reports in our patient cohort [6 28 45]. Supplementary Table 1 shows significant elevation of CRP, ESR, serum globulins, and circulating lymphocytes and monocyte numbers in the opiate dependent group. Consideration of the very flat age-dependent trajectories of these parameters in controls in our earlier reports shows that this effect is much greater in opiate dependence than might be expected on the basis of the higher chronologic age in the opioid dependent group alone. This is in turn consistent with the important chronic inflammatory basis of many vascular diseases [46-48] and the immune theory of organismal ageing [46].[49]

As noted above, vascular age is frequently said to be a surrogate for organismal ageing. The present data are consistent with that view, and also with the literature cited of accelerated ageing in these patients. The present technique provides a mechanism to quantify these changes at the whole human level.

As no structural imaging techniques were performed it is not possible to say with certainty if the observed elevations in vascular stiffness were associated with fixed atherosclerotic lesions. However a large and weighty literature confirms the relevance of significance of elevations in vascular stiffness [43-44 50-52]. Structural correlation must await further work.

The consistency of the present results with the previously reported data from various national and international groups [11-18 53] is clear in that we have confirmed the exacerbation of cardiovascular ageing and arterial stiffness in opiate dependency . However these earlier

For peer review only

results have been extended by demonstrating a measurable major subclinical endophenotype of accelerated ageing antemortem, confirmed a dose-response relationship, demonstrated for the first time the significance of the improved model quadratic in opiate exposure duration, and shown that the opiate-related effect is robust to adjustment for known cardiovascular risk factors in both sexes. Moreover we confirmed that the effect is worse in females [14], and extended this result by showing that the differential effect commences in women over a decade earlier than in men (29 vs. 41 years). In that the PWA technique directly relates increased arterial stiffness to cardiovascular and therefore organismal ageing the present detailed studies link advanced cardiovascular age to many other indices of accelerated ageing in opioid dependency as have been shown by this [6-7 10 26 28 45 54-58] and other [59] groups. As such the present report is a meaningful and important extension of earlier reports from this project on the cardiovascular implications of opioid dependency [30-33], its treatment [19] and withdrawal [23] and the antagonist-induced reversal of these effects [20]. Hence the phenomenology of the present descriptive report is consistent with, and extends, extant reports in the published literature.

Whilst this study is not designed to be mechanistic in nature some brief comment on this issue may be appropriate, in part for its relevance to the consideration of possibly aetiologically causal pathways. Some of the most interesting questions to emerge from the present work relate to the possible mechanisms underlying the plethora of changes which have been identified in opioid dependence as cardiovascular risk factors, as potentially atherogenic processes and as accelerants of ageing processes. Opioids have been linked with blockade of the cell cycle at cyclin dependent kinases 2, 4 and 6 by $P16^{INK4A}$ and $P21^{WAF1/CIP1}$ [60-61]. P16 is the gene product of CDKN2A located at the senescence locus on Chromosome 9p21.3 which is a hotspot for many GWAS studies, and from which three senescence related proteins and one senescence associated long non-coding RNA are transcribed [62]. Furthermore opioid agonists have been shown to directly ligate the myeloid differentiation factor 2 (MD2)- toll-like receptor (TLR) 4 heterodimer leading to the downstream activation of the numerous powerful pathways linked to stimulation of the endotoxin receptor [63]. Indeed systemic endotoxaemia was recently shown to modulate the expression of 6,025 genes [64]. Some of the pathways activated downstream by TLR4 signalling include the cyclooxygenase, lipooxygenase, MAP kinase (JNK and P38), AP-1, inducible nitric oxide synthase (iNOS), phosphoinositol-3 kinase (PI3K), and TGFβ pathways [65], many of which

have been noted to be stimulated within the first few minutes of TLR4 ligation [66], to be stimulated by opioids [63 65], and to be associated with potentiating the effects of ageing [49 67]. Many of these immune products directly exacerbate the stem cell blockade [68]. This then implies that opioid dependency may be conceptualized as chronically, if largely subclinically, ill.

It is very exciting that new research has identified that these processes also act particularly in proopioimelanocortin (POMC) neurons of the paraventricular and arcuate nuclei of the mediobasal hypothalamus which are key determinants of the body's metabolic homeostasis, glucostatic and lipostatic functions, energy balance, appetitive drives, sympathoadrenal activation state, and even lifespan modulation in some organisms [69-71], and that derangements in this region cause metabolic syndrome and its numerous related disorders in model organisms including mammals and likely also humans [72]. These workers have shown that activation of the MD2/TLR4/IKKβ/NF-κB cascade induces endoplasmic reticulum, mitochondrial and autophagic stress, which along with reduced neurogenesis, increased POMC neuronal apoptosis [70], and glial activation and reactive gliosis [72-73] underlie these numerous disorders and derangements. Whilst this research focuses on TLR4 ligation of unsaturated fatty acids and oxidized low density lipoproteins, opioids have been shown to act similarly as xenobiotics through MD2-TLR4 [74], and opioid agonists have been shown to produce POMC cell hyperpolarization and relative refractoriness [42], whilst opioid antagonists have the reverse effect [75]. Small molecule TLR4 inhibitors are under development for several therapeutic applications [76-77]. The confluence then of these two important lines of recent research has the potential to produce major conceptual and therapeutic advances in this field. A clear understanding of direct links from xenobiotic-TLR4-NF-κB signalling to sympathoadrenal tone and hypothalamic lifespan control has the potential to revolutionize the direction of therapeutic endeavours in the treatment of opioid dependence.

It is fascinating that the promoters for two of the key stem cell genes, Oct4 and Nanog have response elements in their promoters for STAT-1 and -3 which are commonly produced as a result of cytokine signalling, particularly by gp-130 cytokines such as IL-6 and leukaemia inhibitory factor (LIF) [78-79]. Furthermore there is three way cross-talk between stem cell and metabolic –immune genes including STAT-3 and PPARγ [80]. Other cytokines, such as IL-1 have been noted in opioid dependency [8] and are usually indicative of inflammasome activation, and fractalkine and MCP-1/CCL2 have also been found to be elevated and are

For peer review only

relevant both to neuropathological and atherogenic processes [63]. As TLR4 shares many ligands and co-chaperones with the receptor for advanced glycation end (RAGE) products this auto-amplificatory system may also be involved [81-84].

This study had various strengths and limitations. The application of sophisticated cardiovascular measurement technology to the problem of drug addiction is unique to this group. Extended follow-up and recruitment of opioid dependent patients is also notoriously difficult. The statistical techniques employed herein are advanced and robust. Design strengths include its complimentary cross-sectional and longitudinal design. Limitations of the present work relate to difficulties quantitating frequently variable amounts of past drug use in the absence of a formal measurement instrument. Use of a formal instrument such as the addiction severity index, and spot tests for drugs and alcohol are indicated in future replications of this study. Employment of structural tests, and mechanistic studies to investigate pathophysiological pathways, would be advisable in future iterations of this work.

In summary the present study documents elevated vascular stiffness and age associated with opioid use which is robust to multiple adjustment to other known risk factors. A strong dose-response effect was noted, and the duration of treatment administration was noted to be particularly important, being related to elevations in vascular age of the first second, third and fourth order. This finding indicates limitation of treatment duration wherever clinically possible. The effect was also worse in females. Overall this study raises many further mechanistic questions which indicate further study on this complex but important model condition.

For peer review only

Acknowledgements

The authors would like to thank Dr Mervyn Thomas of Emphron for assistance with the statistics and graphical design.

Conflict of Interest

Nil declared.

Sources of Funding

Nil.

Statement of Authorship

This work is the work of the authors in its entirety.

For peer review only

References

1. Rosen D, Smith ML, Reynolds CF, 3rd. The prevalence of mental and physical health disorders among older methadone patients. *Am J Geriatr Psychiatry* 2008;16(6):488-97.
2. Cooper OB, Brown TT, Dobs AS. Opiate drug use: a potential contributor to the endocrine and metabolic complications in human immunodeficiency virus disease. *Clin Infect Dis* 2003;37 Suppl 2:S132-6.
3. Ceriello A, Quatraro A, Giugliano D. Opiate addict as diabetic patient? *Diabetes Care* 1988;11(5):443.
4. Kolarzyk E, Pach D, Wojtowicz B, Szpanowska-Wohn A, Szurkowska M. Nutritional status of the opiate dependent persons after 4 years of methadone maintenance treatment. *Przeglad lekarski* 2005;62(6):373-7.
5. Brunton LL, Lazo JS, Parker KL, editors. *Goodman and Gilman's the Pharmacologic Basis of Therapeutics*. Eleventh Edition ed. New York: McGraw Hill, 2006.
6. Reece A. S. Relative and Age Dependent Stimulation of Soluble and Cellular Immunity in Opiate Dependence. *Journal of Addiction Medicine* 2012;6(1):10-17.
7. Reece A. S. High Sensitivity CRP in Opiate Addiction: Relative and Age-Dependent Elevations. *Cardiovascular Toxicology* 2012;12(2):149-57.
8. Neri S, Bruno CM, Pulvirenti D, Malaguarnera M, Italiano C, Mauceri B, et al. Randomized clinical trial to compare the effects of methadone and buprenorphine on the immune system in drug abusers. *Psychopharmacology (Berl)* 2005;179(3):700-4.
9. Reece A. S. Absolute and Age Dependent Elevations of Serum Calcium and Phosphate and their Products in Clinical Opiate Dependence. *J. Subs. Abuse* 2013;Accepted 27th November 2012.
10. Reece AS, Davidson P. Deficit of circulating stem--progenitor cells in opiate addiction: a pilot study. *Substance abuse treatment, prevention, and policy* 2007;2:19-28.
11. Darke S, Duflou J, Torok M. The comparative toxicology and major organ pathology of fatal methadone and heroin toxicity cases. *Drug Alcohol Depend* 2010;106(1):1-6.
12. Darke S, Kaye S, Duflou J. Systemic disease among cases of fatal opioid toxicity. *Addiction* 2006;101(9):1299-305.
13. Degenhardt L., Randall D., Hall W., Law M., Butler T., Burns L. Mortality among clients of a state-wide opioid pharmacotherapy program over 20 years: Risk factors and lives saved. *Drug Alcohol Depend* 2009;105(1-2):9-15.
14. Khademi H, Malekzadeh R, Pourshams A, Jafari E, Salahi R, Semnani S, et al. Opium use and mortality in Golestan Cohort Study: prospective cohort study of 50,000 adults in Iran. *BMJ (Clinical research ed* 2012;344:e2502.
15. Sadeghian S, Darvish S, Davoodi G, Salarifar M, Mahmoodian M, Fallah N, et al. The association of opium with coronary artery disease. *Eur J Cardiovasc Prev Rehabil* 2007;14(5):715-7.
16. Sadeghian S, Dowlatshahi S, Karimi A, Tazik M. Epidemiology of opium use in 4398 patients admitted for coronary artery bypass graft in Tehran Heart Center. *J Cardiovasc Surg (Torino)* 2011;52(1):140-1.
17. Sadeghian S, Graili P, Salarifar M, Karimi AA, Darvish S, Abbasi SH. Opium consumption in men and diabetes mellitus in women are the most important risk factors of premature coronary artery disease in Iran. *International journal of cardiology* 2010;141(1):116-8.
18. Smyth B, Hoffman V, Fan J, Hser YI. Years of potential life lost among heroin addicts 33 years after treatment. *Preventive medicine* 2007;44(4):369-74.
19. Reece A.S., Hulse G.K. Impact of Opioid Pharmacotherapy on Arterial stiffness and Vascular Ageing: Cross-sectional and Longitudinal Studies. *Cardiovascular Toxicology* 2013;13(3):254-66.
20. Reece A. S., Hulse G.K. Reduction in Arterial Stiffness and Vascular Age by Naltrexone-Induced Interruption of Opiate Agonism. *British Medical Journal - Open* 2013.

For peer review only

21. Hosseini SK, Masoudkabir F, Vasheghani-Farahani A, Alipour-Parsa S, Sheikh Fathollahi M, Rahimi-Foroushani A, et al. Opium consumption and coronary atherosclerosis in diabetic patients: a propensity score-matched study. *Planta Med* 2011;77(17):1870-5.
22. Charney DS, Redmond DE, Jr., Galloway MP, Kleber HD, Heninger GR, Murberg M, et al. Naltrexone precipitated opiate withdrawal in methadone addicted human subjects: evidence for noradrenergic hyperactivity. *Life Sci* 1984;35(12):1263-72.
23. Reece AS, Hulse GK. Elevation of Central Arterial Stiffness and Vascular Ageing in Opiate Withdrawal: Cross-sectional and Longitudinal Studies. *Cardiovasc Toxicol* 2012;13(1):55-67.
24. Chien KR, Karsenty G. Longevity and lineages: toward the integrative biology of degenerative diseases in heart, muscle, and bone. *Cell* 2005;120(4):533-44.
25. Lakatta EG, Wang M, Najjar SS. Arterial aging and subclinical arterial disease are fundamentally intertwined at macroscopic and molecular levels. *Med Clin North Am* 2009;93(3):583-604, Table of Contents.
26. Reece AS. Hair graying in substance addiction. *Archives of dermatology* 2007;143(1):116-8.
27. Kim TW, Alford DP, Malabanan A, Holick MF, Samet JH. Low bone density in patients receiving methadone maintenance treatment. *Drug Alcohol Depend* 2006;85(3):258-62.
28. Reece AS. Evidence of Accelerated Ageing in Clinical Drug Addiction from Immune, Hepatic and Metabolic Biomarkers. *Immun Ageing* 2007;4(1):6-15.
29. Randall D, Degenhardt L, Vajdic CM, Burns L, Hall WD, Law M, et al. Increasing cancer mortality among opioid-dependent persons in Australia: a new public health challenge for a disadvantaged population. *Australian and New Zealand journal of public health* 2011;35(3):220-5.
30. Reece A.S., Hulse G.K. Lifetime Opiate Exposure as an Independent and Interactive Cardiovascular Risk Factor in Males: A Cross-Sectional Clinical Study. *Vascular Health and Risk Management* 2013;9(55):551-59.
31. Reece A.S., Hulse G.K. Opiate Exposure Increases Arterial Stiffness, Advances Vascular Age and is an Independent Cardiovascular Risk Factor in Females: A Cross-Sectional Clinical Study. *World J. Cardiovascular Diseases* 2013;In Press(DOI: 10.4236/wjcd.2013.35056).
32. Reece A. S., Hulse G.K. Duration of Opiate Exposure as a Determinant of Arterial Stiffness and Vascular Age in Male Opiate Dependence: A Longitudinal Study. *Journal of Clinical Pharmacy and Therapeutics* 2013;In Press(DOI: 10.1111/jcpt.12121).
33. Reece A. S., Hulse G.K. Opiate Dependence as an Independent and Interactive Risk Factor for Arterial Stiffness and Cardiovascular Ageing - A Longitudinal Study in Females. *J Clinical Medical Research* 2013;5(5):356-67.
34. Reece AS. Psychosocial and treatment correlates of opiate free success in a clinical review of a naltrexone implant program. *Subst Abuse Treat Prev Policy* 2007;2:35-49.
35. Breslow N.E., Day N.E. *Statistical Methods in Cancer Research Volume II The Design and Analysis of Cohort Studies*. 1 ed. Lyon: International Agency for Cancer Research,, 1987.
36. Kacinko SL, Jones HE, Johnson RE, Choo RE, Huestis MA. Correlations of maternal buprenorphine dose, buprenorphine, and metabolite concentrations in meconium with neonatal outcomes. *Clinical pharmacology and therapeutics* 2008;84(5):604-12.
37. Hulse G., White J., Cape G., editors. *Management of Drug and Alcohol Problems*. Sydney: Oxford University Press, 2002.
38. Oviedo-Joekes E, Brissette S, Marsh DC, Lauzon P, Guh D, Anis A, et al. Diacetylmorphine versus methadone for the treatment of opioid addiction. *N Engl J Med* 2009;361(8):777-86.
39. SphygmoCor, editor. *Software Operator's Guide, Pulse Wave Analysis System, SCOR-Px*. Sydney: AtCor Medical, 2006.
40. Klein SL. Sex influences immune responses to viruses, and efficacy of prophylaxis and treatments for viral diseases. *Bioessays* 2012;34(12):1050-9.
41. Reece AS. Hypothalamic opioid-melanocortin appetitive balance and addictive craving. *Medical hypotheses* 2011;76(1):132-7.
42. Cowley MA, Smart JL, Rubinstein M, Cerdan MG, Diano S, Horvath TL, et al. Leptin activates anorexigenic POMC neurons through a neural network in the arcuate nucleus. *Nature* 2001;411(6836):480-4.

For peer review only

43. Nichols W.W., O'Rourke M.F., editors. *McDonald's Blood Flow in Arteries: Theoretical, Experimental and Clinical Principals*. London: Hodder Arnold, 2005.
44. O'Rourke MF, Pauca AL. Augmentation of the aortic and central arterial pressure waveform. *Blood Press Monit* 2004;9(4):179-85.
45. Reece AS. High-sensitivity CRP in opiate addiction: relative and age-dependent elevations. *Cardiovasc Toxicol* 2012;12(2):149-57.
46. Fauci AS, Braunwald E, Kapser DL, Hauser SL, Longo DL, Jameson JL, editors. *Harrison's Principles of Internal Medicine, 17th Edition*. 17th Edition ed. New York: McGraw Hill, 2008.
47. Lakatta EG. Arterial aging is risky. *J Appl Physiol* 2008;105(4):1321-2.
48. Le Couteur DG, Lakatta EG. A vascular theory of aging. *J Gerontol A Biol Sci Med Sci* 2010;65(10):1025-7.
49. Hadley EC, Lakatta EG, Morrison-Bogorad M, Warner HR, Hodes RJ. The future of aging therapies. *Cell* 2005;120(4):557-67.
50. Weber T, Auer J, O'Rourke MF, Kvas E, Lassnig E, Berent R, et al. Arterial stiffness, wave reflections, and the risk of coronary artery disease. *Circulation* 2004;109(2):184-9.
51. Williams B, Lacy PS, Thom SM, Cruickshank K, Stanton A, Collier D, et al. Differential impact of blood pressure-lowering drugs on central aortic pressure and clinical outcomes: principal results of the Conduit Artery Function Evaluation (CAFE) study. *Circulation* 2006;113(9):1213-25.
52. Nichols WW. Clinical measurement of arterial stiffness obtained from noninvasive pressure waveforms. *Am J Hypertens* 2005;18(1 Pt 2):3S-10S.
53. Degenhardt L, Larney S, Randall D, Burns L, Hall W. Causes of death in a cohort treated for opioid dependence between 1985 and 2005. *Addiction* 2013.
54. Reece A. S. Manifold Implications of Forgotten Hyperglycaemia in Clinical Opiate Dependence. *Drug and Chemical Toxicology* 2012.
55. Reece AS. Relative and Age-dependent Stimulation of Soluble and Cellular Immunity in Opiate Dependence. *J Addict Med* 2012;6(1):10-7.
56. Reece AS. Differing age related trajectories of dysfunction in several organ systems in opiate dependence. *Aging Clin Exp Res* 2012;24(1):85-96.
57. Reece AS. Dentition of addiction in Queensland: poor dental status and major contributing drugs. *Australian dental journal* 2007;52(2):144-9.
58. Reece A. Lifetime prevalence of cervical neoplasia in addicted and medical patients. *Aust N Z J Obstet Gynaecol* 2007;47(5):419-23.
59. Tatia M.C. Lee, Gordon L.F. Cheng, Hung Zeng, Meikei Leung, Hui-Jun Zhang, Benson W.M. Lau, et al. Heroin abuse accelerates biological aging: A novel insight from telomerase and brain imaging interaction. *Translational Psychiatry* 2013.
60. Cheng F, McLaughlin PJ, Verderame MF, Zagon IS. The OGF-OGFr axis utilizes the p16INK4a and p21WAF1/CIP1 pathways to restrict normal cell proliferation. *Molecular biology of the cell* 2009;20(1):319-27.
61. Zagon IS, Verderame MF, McLaughlin PJ. The biology of the opioid growth factor receptor (OGFr). *Brain research* 2002;38(3):351-76.
62. Pasmant E, Sabbagh A, Vidaud M, Bieche I. ANRIL, a long, noncoding RNA, is an unexpected major hotspot in GWAS. *FASEB J* 2010;25(2):444-48.
63. Hutchinson MR, Shavit Y, Grace PM, Rice KC, Maier SF, Watkins LR. Exploring the neuroimmunopharmacology of opioids: an integrative review of mechanisms of central immune signaling and their implications for opioid analgesia. *Pharmacological reviews* 2011;63(3):772-810.
64. Starr ME, Hu Y, Stromberg AJ, Carmical JR, Wood TG, Evers BM, et al. Gene expression profile of mouse white adipose tissue during inflammatory stress: age-dependent upregulation of major pro-coagulant factors. *Aging Cell* 2012:n/a-n/a.
65. Coller JK, Hutchinson MR. Implications of central immune signaling caused by drugs of abuse: mechanisms, mediators and new therapeutic approaches for prediction and treatment of drug dependence. *Pharmacol Ther* 2012;134(2):219-45.

For peer review only

66. Grzanna R., Brown R.M. NIDA Research Monograph 125: Activation of Intermediate Early Genes by Drugs of Abuse. In: National Institutes of Drug Abuse, editor. Bethesda, Maryland: NIH, 1993:1-220.
67. Kirkwood TB. Understanding the odd science of aging. *Cell* 2005;120(4):437-47.
68. Hoing S, Rudhard Y, Reinhardt P, Glatza M, Stehling M, Wu G, et al. Discovery of inhibitors of microglial neurotoxicity acting through multiple mechanisms using a stem-cell-based phenotypic assay. *Cell Stem Cell* 2012;11(5):620-32.
69. Zhang G, Li J, Purkayastha S, Tang Y, Zhang H, Yin Y, et al. Hypothalamic programming of systemic ageing involving IKK-[bgr], NF-[kgr]B and GnRH. *Nature* 2013;497(7448):211-16.
70. Li J, Tang Y, Cai D. IKKbeta/NF-kappaB disrupts adult hypothalamic neural stem cells to mediate a neurodegenerative mechanism of dietary obesity and pre-diabetes. *Nat Cell Biol* 2012;14(10):999-1012.
71. Purkayastha S, Zhang G, Cai D. Uncoupling the mechanisms of obesity and hypertension by targeting hypothalamic IKK-beta and NF-kappaB. *Nat Med* 2011;17(7):883-7.
72. Thaler JP, Yi CX, Schur EA, Guyenet SJ, Hwang BH, Dietrich MO, et al. Obesity is associated with hypothalamic injury in rodents and humans. *J Clin Invest* 2012;122(1):153-62.
73. Czapski GA, Gajkowska B, Strosznajder JB. Systemic administration of lipopolysaccharide induces molecular and morphological alterations in the hippocampus. *Brain Res* 2010;1356:85-94.
74. Wang X, Loram LC, Ramos K, de Jesus AJ, Thomas J, Cheng K, et al. Morphine activates neuroinflammation in a manner parallel to endotoxin. *Proc Natl Acad Sci U S A* 2012;109(16):6325-30.
75. Billes SK, Greenway FL. Combination therapy with naltrexone and bupropion for obesity. *Expert opinion on pharmacotherapy* 2011;12(11):1813-26.
76. Neal MD, Jia H, Eyer B, Good M, Guerriero CJ, Sodhi CP, et al. Discovery and validation of a new class of small molecule Toll-like receptor 4 (TLR4) inhibitors. *PLoS ONE* 2013;8(6):e65779.
77. Theberge FR, Li X, Kambhampati S, Pickens CL, St Laurent R, Bossert JM, et al. Effect of chronic delivery of the Toll-like receptor 4 antagonist (+)-naltrexone on incubation of heroin craving. *Biol Psychiatry* 2013;73(8):729-37.
78. Kim CG, Chung IY, Lim Y, Lee YH, Shin SY. A Tcf/Lef element within the enhancer region of the human NANOG gene plays a role in promoter activation. *Biochem Biophys Res Commun* 2011;410(3):637-42.
79. Yang HM, Do HJ, Oh JH, Kim JH, Choi SY, Cha KY, et al. Characterization of putative cis-regulatory elements that control the transcriptional activity of the human Oct4 promoter. *J Cell Biochem* 2005;96(4):821-30.
80. Folmes CD, Dzeja PP, Nelson TJ, Terzic A. Metabolic plasticity in stem cell homeostasis and differentiation. *Cell Stem Cell* 2012;11(5):596-606.
81. Zurolo E, Iyer A, Maroso M, Carbonell C, Anink JJ, Ravizza T, et al. Activation of Toll-like receptor, RAGE and HMGB1 signalling in malformations of cortical development. *Brain* 2011;134(Pt 4):1015-32.
82. Nogueira-Machado JA, Volpe CM, Veloso CA, Chaves MM. HMGB1, TLR and RAGE: a functional tripod that leads to diabetic inflammation. *Expert opinion on therapeutic targets* 2011;15(8):1023-35.
83. Sakaguchi M, Murata H, Yamamoto K, Ono T, Sakaguchi Y, Motoyama A, et al. TIRAP, an adaptor protein for TLR2/4, transduces a signal from RAGE phosphorylated upon ligand binding. *PLoS ONE* 2011;6(8):e23132.
84. Veloso CA, Fernandes JS, Volpe CM, Fagundes-Netto FS, Reis JS, Chaves MM, et al. TLR4 and RAGE: similar routes leading to inflammation in type 2 diabetic patients. *Diabetes Metab* 2011;37(4):336-42.

For peer review only

For peer review only

Figure Captions:

Figure 1: Ageing and Stiffness Indices by Chronologic Age by Opioid Dependency Status

Figure 2: Corrected Ageing and Stiffness Indices by Time by Opioid Dependency Status

Figure 3: Ageing Indices by Chronologic Age by Addictive Status by Sex

Figure 4: Ageing Indices by Chronologic Age by Sex by Addictive Status

Figure 5: BMI-Related Ageing Indices by Chronologic Age by Addictive Status by Sex

Figure 6: BMI-Related Ageing Indices by Chronologic Age by Sex by Addictive Status

Figure 7: Ageing Indices by Chronologic Age by Sex by Addictive Status – Loess Curves

Figure 8: Vascular Age Indices by Chronologic Age by Opioid Dependency Level, Loess curves

Supplementary Figure Captions:

Suppl. Fig. 1: Vascular Age Indices by Time by Opioid Dependency Level

For peer review only

TABLES

For peer review only

Table 1.: Effects of Gender on Key Measures of Arterial Stiffness - Cross-sectional Dataset

Dependent Variable	Group	Parameter	Adj. R^2	F	dF	Model P	Estimate	Std. Error	t-Value	P
RA-CA Difference	Opioid	Chron.Age :Female.Sex	0.0189	7.596	2, 684	0.0005459	0.6324	0.3469	1.823	0.0688
BMI	Control	Chron.Age :Female.Sex	0.1523	52.58	2, 572	<2.2E-16	-0.0092	0.0040	-2.310	0.0213
BMI	Opioid	Female.Sex	0.0713	27.35	2, 684	3.75E-12	-0.0827	0.0139	-5.940	4.6E-09
RA/BMI	Opioid	Chron.Age :Female.Sex	0.2617	122.6	2, 684	<2.2E-16	0.0361	0.0091	3.948	8.7E-05
C_AP_HR75/BMI	Control	Chron.Age :Female.Sex	0.4882	274.3	2, 571	<2.2E-16	0.0312	0.0040	7.737	4.6E-14
C_AP_HR75/BMI	Control	Chron.Age :Female.Sex	0.4882	274.3	2, 571	<2.2E-16	0.0312	0.0040	7.737	4.6E-14
C_AP_HR75/BMI	Opioid	Chron.Age :Female.Sex	0.3712	203.4	2, 684	<2.2E-16	0.0467	0.0039	11.950	<2e-16
C_AGPH_HR75/BMI	Control	Chron.Age :Female.Sex	0.4603	245.4	2, 571	<2.2E-16	0.0924	0.0112	8.283	8.6E-16
C_AGPH_HR75/BMI	Opioid	Chron.Age :Female.Sex	0.3980	227.8	2, 684	<2.2E-16	0.1265	0.0095	13.370	<2e-16
C_AP_HR75/(CA*BMI)	Control	Female.Sex	0.2809	112.9	2, 571	<2.2E-16	0.0033	0.0005	6.286	6.5E-10
C_AP_HR75/(CA*BMI)	Opioid	Female.Sex	0.2489	114.7	2, 684	<2.2E-16	0.0047	0.0004	11.428	<2e-16
C_AGPH_HR75/(CA*BMI)	Control	Female.Sex	0.2567	99.96	2, 571	<2.2E-16	0.0096	0.0015	6.350	4.4E-10
C_AGPH_HR75/(CA*BMI)	Opioid	Female.Sex	0.2604	121.8	2, 684	<2.2E-16	0.0130	0.0011	12.307	<2e-16

For peer review only

Table 2.:

Age: Addictive Status Relationships with CA Discretized

About 41 Years in Males and 29 Years in Females

With Females <55 Years of Age

Parameter	Variable Statistics				Model Summaries		
	Estimate	Std. Error	t Value	P	F	dF	P
Males							
Older Age	-0.1837	0.0557	-3.296	0.0010	5.492	3, 862	0.00097
Opioids	-0.0846	0.0275	-3.082	0.0021			
Older Age: Opioids	0.2310	0.0687	3.364	0.0008			
Females							
Older Age: Opioids	0.1248	0.0532	2.346	0.0195	2.169	3, 378	0.09123

For peer review only

Table 3.: Cross-sectional Models Polynomial in Heroin Duration

	Parameter				Model		
Model / Parameter	Value	Std.Error	t-value	P-value	F	dF	P
1 Order- Linear							
H.Dura.n	-0.0059	0.0021	-2.779	0.0056	8.628	2, 664	0.0002
H.Dose: H.Dura.n	0.0091	0.0022	4.152	3.7.E-05			
2 Order- Quadratic							
H.Dura.n	-0.0171	0.0055	-3.136	0.0018	7.443	3, 663	6.60E-05
H.Dura.n^2	0.0004	0.0002	2.229	0.0261			
H.Dose: H.Dura.n	0.0084	0.0022	3.802	0.0002			
3 Order - Cubic							
H.Dura.n	-0.0116	0.0035	-3.263	0.0012	7.102	3 663	0.00011
H.Dura.n^3	6.3E-06	3.1E-06	1.993	0.0466			
H.Dose: H.Dura.n	0.0084	0.0022	3.836	0.0001			
4 Order- Quartic							
H.Dura.n	-0.0059	0.0021	-2.779	0.0056	8.628	2, 664	0.0002
H.Dose: H.Dura.n	0.0091	0.0022	4.152	3.7.E-05			
1, 2, 3 & 4 Order							
H.Dose: H.Dura.n^3	6.5E-05	2.0E-05	3.252	0.0012	3.353	7, 659	0.0016
H.Dura.n^2 : H.Dura.n^3	-1.0E-06	3.1E-07	-3.398	0.0007			
H.Dura.n^2 : H.Dura.n^4	9.2E-08	2.9E-08	3.216	0.0014			
H.Dura.n^3: H.Dura.n^4	-2.7E-09	8.8E-10	-3.029	0.0026			
H.Dose: H.Dura.n^2 : H.Dura.n^4	-4.5E-09	1.8E-09	-2.452	0.0145			
H.Dura.n: H.Dura.n^3: H.Dura.n^4	2.5E-11	9.0E-12	2.831	0.0048			
H.Dose: H.Dura.n: H.Dura.n^3: H.Dura.n^4	2.6E-12	1.2E-12	2.159	0.0312			

For peer review only

Table 4: Dose-Response Relationships

Cross-Sectional Studies							
Parameter	Variable Statistics				Model Summaries		
	Estimate	Std. Error	t Value	Pr(>|t|)	F	dF	P
Both Sexes							
H.Dura.n	-0.0059	0.0021	-2.779	0.0056	8.628	2, 664	0.0002
H.Dose: H.Dura.n	0.0091	0.0022	4.152	0.0000			
Males							
H.Dura.n	-0.0089	0.0028	-3.147	0.0018	7.105	2, 441	0.0009
H.Dose: H.Dura.n	0.0124	0.0034	3.658	0.0003			
Females							
H.Dose: H.Dura.n	0.0052	0.0023	2.281	0.0236	5.201	1, 210	0.0236

Longitudinal Studies<br>-							
**Group**	**Variable Statistics**					**Model**	
**Parameter**	**Value**	**Std.Error**	**DF**	**t-value**	**P-value**	**AIC***	**LogLik***
Both Sexes							
H.Dose:H.Dura.n	0.0045	0.0013	814	3.5512	0.0004	1068.74	-530.37
Males							
H.Dura.n	-0.0036	0.0016	552	-2.2069	0.0277	753.15	-371.58
H.Dose:H.Dura.n	0.0075	0.0022	552	3.3754	0.0008		
Females							
H.Dose:H.Dura.n	0.0052	0.0021	260	2.5091	0.0127	347.43	-169.72

For peer review only

Table 5.: Cross-Sectional Adjusted Multiple Regression Models

Group	Variable Statistics				Model		
Parameter	Estimate	Std. Error	t Value	Pr(>|t|)	F	dF	P
Both Sexes							
H.Dura.n: Cigs.d: SP : Weight	0.00023	0.00006	3.919	0.0001	7.561	9, 657	1.38E-10
H.Dura.n: Height: Cigs.d: SP	0.00030	0.00011	2.840	0.0047			
H.Dura.n: Height: Cigs.d	-0.00143	0.00051	-2.828	0.0048			
H.Dura.n: Height: Cigs.d: SP : Weight	-0.00007	0.00002	-2.808	0.0051			
H.Dura.n: Height: Cigs.d: Weight	0.00032	0.00012	2.741	0.0063			
H.Dose: H.Dura.n: Height: Cigs.d: Weight	-0.00037	0.00016	-2.229	0.0261			
H.Dose: H.Dura.n: Height: Cigs.d: SP : Weight	0.00008	0.00003	2.229	0.0262			
H.Dose: H.Dura.n: Height: Cigs.d	0.00155	0.00071	2.194	0.0286			
H.Dose: H.Dura.n: Height: Cigs.d: SP	-0.00032	0.00015	-2.188	0.0290			
Males							
H.Dura.n: Weight	-0.0024	0.0007	-3.434	0.0007	5.485	10, 438	1.07E-07
H.Dose: H.Dura.n: Weight	0.0032	0.0010	3.158	0.0017			
H.Dose: Cigs.d: SP : Weight	-4.4770	1.7470	-2.563	0.0107			
H.Dose: Height: Cigs.d: SP : Weight	0.0253	0.0099	2.557	0.0109			
H.Dose: Cigs.d: SP	19.460	7.6160	2.556	0.0109			
H.Dose: Cigs.d: Weight	21.370	8.3900	2.548	0.0112			
H.Dose: Height: Cigs.d: Weight	-0.1209	0.0476	-2.542	0.0114			
H.Dose: Height: Cigs.d: SP	-0.1098	0.0432	-2.540	0.0114			
H.Dose: Cigs.d	-92.680	36.520	-2.538	0.0115			
H.Dose: Height: Cigs.d	0.5230	0.2073	2.523	0.0120			
Females							
Height: Cigs.d: Weight	-0.00041	0.00009	-4.327	0.00002	8.25	3, 214	3.20E-05
Height: Cigs.d: SP : Weight	0.00008	0.00002	4.272	0.00003			
H.Dose: H.Dura.n: Height: Cigs.d	2.2E-06	1.1E-06	1.957	0.05160			

For peer review only

Table 6.: Longitudinal Adjusted Multiple Models

Group	Variable Statistics					Model	
**Model / Parameter**	**Value**	**Std.Error**	**DF**	**t-value**	**P-value**	**AIC***	**LogLik***
Both Sexes							
Height	-0.0304	0.0026	606	-11.7198	0.0E+00	1238.239	-592.1197
SP	0.7947	0.0949	606	8.3772	0.0E+00		
Days:H.Dura.n	0.0003	6.9E-05	606	4.3556	0.0E+00		
CA:Height	0.0066	0.0006	606	10.9049	0.0E+00		
Days: CA:H.Dura.n	-0.0001	0.0000	606	-4.2946	0.0E+00		
H.Dura.n: Cigs.d:Height	-5.3E-05	1.3E-05	606	-4.1566	0.0E+00		
Days:H.Dose: Cigs.d:Height	2.1E-06	5.0E-07	606	4.3108	0.0E+00		
Days: CA:H.Dose: Cigs.d:Height	-6.0E-07	2.0E-07	606	-4.2968	0.0E+00		
CA:H.Dura.n: Cigs.d:Height	1.4E-05	3.4E-06	606	4.0217	0.0001		
Days: CA: Cigs.d:Height	0.0E+00	0.0E+00	606	3.7701	0.0002		
Days:H.Dura.n: Cigs.d	-1.1E-05	3.1E-06	606	-3.4709	0.0006		
Cigs.d:Height	0.0007	0.0002	606	3.3714	0.0008		
Days: CA:H.Dose:H.Dura.n: Cigs.d:Height	0.0E+00	0.0E+00	606	3.2705	0.0011		
Days: CA:H.Dura.n: Cigs.d	2.5E-06	8.0E-07	606	3.2365	0.0013		
CA: Cigs.d:Height	-0.0002	0.0001	606	-3.2045	0.0014		
Days	-0.0022	0.0007	606	-2.9194	0.0036		
H.Dose:H.Dura.n: Cigs.d	0.0102	0.0036	606	2.8304	0.0048		
CA:H.Dose:H.Dura.n: Cigs.d	-0.0026	0.0010	606	-2.6804	0.0076		
Days: CA	0.0005	0.0002	606	2.2438	0.0252		
Days: CA:H.Dose:Height	6.8E-06	3.1E-06	606	2.1984	0.0283		
Days:H.Dose:Height	-2.2E-05	1.0E-05	606	-2.1747	0.0300		
Days:H.Dose:H.Dura.n	-3.5E-05	1.7E-05	606	-2.1086	0.0354		
H.Dose: Cigs.d	-0.1067	0.0511	606	-2.0888	0.0371		
CA:H.Dose: Cigs.d	0.0290	0.0148	606	1.9645	0.0499		
Males							
Height	-0.0302	0.0035	436	-8.7423	0.0E+00	913.00	-435.50
SP	0.6376	0.1105	436	5.7717	0.0E+00		
CA:Height	0.0064	0.0008	436	8.4711	0.0E+00		
Days:H.Dura.n	0.0003	0.0001	436	3.8907	0.0001		
Days: CA:H.Dura.n	-0.0001	0.0000	436	-3.8395	0.0001		
Days:H.Dose: Cigs.d:Height	1.6E-06	4.0E-07	436	3.8181	0.0002		
Days: CA:H.Dose: Cigs.d:Height	-5.0E-07	1.0E-07	436	-3.7217	0.0002		
Days	-0.0029	0.0008	436	-3.6624	0.0003		
Days: CA	0.0007	0.0002	436	3.3251	0.0010		
H.Dose:H.Dura.n: Cigs.d:Height	2.7E-06	8.0E-07	436	3.2331	0.0013		
Days: CA: Cigs.d	5.0E-06	1.6E-06	436	3.1839	0.0016		
H.Dura.n: Cigs.d:Height	-2.8E-05	9.1E-06	436	-3.1058	0.0020		
CA:H.Dura.n: Cigs.d:Height	7.1E-06	2.4E-06	436	2.9496	0.0034		
Days:H.Dura.n: Cigs.d	-8.3E-06	3.1E-06	436	-2.6824	0.0076		
Days: CA:H.Dura.n: Cigs.d	2.0E-06	8.0E-07	436	2.5562	0.0109		
Days: CA:H.Dose:H.Dura.n: Cigs.d:Height	0.0E+00	0.0E+00	436	2.2838	0.0229		
Cigs.d:Height	0.0004	0.0002	436	2.2799	0.0231		
CA: Cigs.d:Height	-0.0001	5.0E-05	436	-2.1938	0.0288		

For peer review only

Females							
Height	-0.0279	0.0036	169	-7.6753	0.0E+00	394.69	-187.35
SP	1.2880	0.1930	169	6.6744	0.0E+00		
CA:Height	0.0064	0.0005	169	11.7493	0.0E+00		
Days: CA:H.Dose:H.Dura.n:Height	1.3E-06	5.0E-07	169	2.7371	0.0069		
Days:H.Dose:H.Dura.n:Height	-4.5E-06	1.7E-06	169	-2.7147	0.0073		
Days: CA:H.Dose:H.Dura.n: Cigs.d:Height	-1.0E-07	0.0E+00	169	-2.6052	0.0100		
Days:H.Dose:H.Dura.n: Cigs.d:Height	2.0E-07	1.0E-07	169	2.5589	0.0114		

For peer review only

~~Lifetime Opiate~~Opioid ~~Exposure is an Interactive Cardiovascular Risk Factor for Vascular Ageing Related to Power Functions of Exposure Duration Particularly Marked in Females: Cross-sectional and Longitudinal Studies~~

Impact of Lifetime Opioid Exposure on Arterial Stiffness and Vascular Age: Cross-sectional and Longitudinal Studies in Males and Females.

Formatted: Font: Bold, Italic

Formatted: Font: Bold, Italic

Formatted: Font: Bold, Italic

Short Title:

~~Opiate~~Opioids as ~~a~~ Cardiovascular Risk Factors

Albert Stuart Reece

Gary Kenneth Hulse

School of Psychiatry and Clinical Neurosciences,

University of Western Australia.

Crawley WA 6009, Australia.

* **Correspondence to**:

Albert Stuart Reece

39 Gladstone Rd.,

Highgate Hill,

Brisbane, Queensland, Australia.

Ph: (617) 3844-4000

FAX: (617) 3844-4015

Email: sreece@bigpond.net.au

Word Count: 8,656.

For peer review only

Keywords – arterial stiffness, ~~opiate~~opioid dependence, vascular ageing, biological age, acceleration of ageing

For peer review only

Structured Abstract

Objective. To characterize and compare the potentiation of arterial stiffness and vascular ageing by opioids in males and females.

~~**Objective**. To compare the potentiation of opiate exposure on vascular / biological ageing and arterial stiffness between males and females.~~

Design. Cross-sectional and longitudinal studies of 576 clinical controls and 687 ~~opiate~~opioid dependent patients (ODP) on 710 and 1,305 occasions over a total of 2,382 days (6.52 years), 2006-2011. Methodology: Radial Pulse Wave Analysis with Atcor SphygmoCor system (Sydney).

Setting. Primary care.

Participants. Controls: General practice patients with non-cardiovascular disorders, and university student controls. ODP: Patients undergoing clinical management of their ~~opiate~~opioid dependence. Controls had lower chronologic ages (CA) than ODP (30.0~~29.96~~±0.45 vs. 34.5~~5~~±0.3~~2~~, mean±S.E.M., P<0.0001). Subjects were 69.6~~2~~% and 67.7~~69~~% male, and 16~~5~~.09~~7~~% and 92.3~~29~~% were smokers (P<0.0001) for controls and ODP respectively. 86.3~~2~~%, 10.3~~3~~% and 3.4~~35~~% of ODP were treated with buprenorphine (6.98±0.21mg), methadone (63.04±4.01mg) or implant naltrexone respectively. BMI was depressed in ODP.

Interventions. ~~Radial Pulse Wave Analysis was undertaken with Atcor SphygmoCor software (Sydney) and Miller microtonometer.~~ Nil.

Primary Outcome Measures - Vascular Reference Age (RA) and the ratio of the vascular age to the chronological age (RA/CA). Secondary outcome measures: Arterial Stiffness including Augmentation Index. ~~Radial Pulse Wave Analysis was undertaken with Atcor SphygmoCor software (Sydney) and Miller microtonometer.~~

For peer review only - http://bmjopen.bmj.com/site/about/guidelines.xhtml

Results. After Body Mass Index -adjustment, RA in ODP was higher as a function of CA and of time (both P<0.05). Modelled mean ~~ages~~ RA in control and ODP males was 35.6~~1~~ and 36.3~~1~~ years (+1.97%), and in females 34.54 and 39.~~18~~2 years (+13.43%). Changes in RA and major arterial stiffness indices were worse with CA in females (P<0.01). Quadratic, cubic and quartic functions of ~~opiate~~opioid exposure duration out-performed linear models with RA/CA over CA and over time. The ~~opiate~~opioid dose-response relationship persisted longitudinally after ~~multivariate~~multiple adjustment from P=0.0013 in males and P=0.0073 in females.

Conclusion. Data show that lifetime ~~opiate~~opioid exposure is an interactive cardiovascular risk factor, particularly in females, is related to linear, quadratic, cubic and quartic functions of treatment duration and is consistent with other literature of accelerated ageing in OD patients.

For peer review only

Article Summary

Article Focus

- Despite striking consistency between several large epidemiological studies of the cardiovascular toxicity of long term ~~opiate~~opioid dependence, the subject is not well studied nor widely understood.

- There are ~~is~~ only ~~a single~~ two (contradictory) studies~~y~~ of gender differences in significantly exposed populations ~~known~~ in the published literature.

- The implications of organism-wide xenobiotic-induced inflammation have not been incorporated into the clinical practice of addiction medicine, our understanding of the inflammatory basis of atherogenesis, or the fact that vascular ageing is a major surrogate for systemic ageing across the whole organism.

Key Messages

- ~~Opiate~~Opioid dependent patients (ODP) had and advance of mean vascular age from control of 35.61 to 36.31 years in males and 34.54 to 39.18 years in females which, after adjustment for BMI, were significant in both males and females cross-sectionally and longitudinally (all P<0.05). Both the acceleration of vascular ageing and arterial stiffness were worse in females (P<0.01) where it occurred 12 years earlier (29 vs 41 years).

- A dose-response relationship between lifetime ~~opiate~~opioid exposure and measures of arterial stiffness and vascular age was demonstrated in both sexes, which was robust to ~~multivariate~~multiple adjustment for known cardiovascular risk factors.

- Use of quadratic, cubic and quartic functions of ~~opiate~~opioid duration exposure significantly improved the statistical fit of models of the ratio of vascular age (RA) to chronological age (CA, RA/CA ratio), thereby further highlighting the significance of the duration for which ~~opiate~~opioid agonism is administered.

For peer review only

Strengths and Limitations

- With 576 controls and 687 OPD the large size of this study was a signficant advantage, as was its combined cross-sectional and longitudinal design, its ability to control for many cardiovascular risk factors, the sex specific analysis, and the diverse output of cardiovascular parameters of the Atcor / SphygmoCor Pulse Wave Analysis technology employed.

- The use of ~~advanced~~ detailed statistical modelling of relationships between factors, dose-response modelling, polynomial modelling in ~~opiate~~opioid exposure duration, inter-quartile trend effects, and ~~multivariate~~ adjustment for known cardiovascular risk factors added further analytical power to the study.

- As the major study limitation was its lack of other measures of organism ageing and its lack of mechanistic and ageing medicine-specific biomarkers, further studies incorporating these refinements are indicated.

For peer review only

Introduction

~~Opiate~~Opioid dependence ~~(OD)~~ is a condition of increasing public health importance.

~~OD~~ Opioid Dependence is associated with an impressive array of traditional and novel cardiovascular risk factors and associations including hypertension [1], hyperlipidaemia [2-3], tobacco consumption, increased body weight [4], hyperglycaemia [3] , hyperfibrinogenaemia [3], diffuse polyclonal gammopathy and immune stimulation [5-6], elevated high sensitivity CRP [7], elevated cytokines [8], relative hypercalcaemia and hyperphosphataemia [9] and reduced circulating endothelial progenitor cells [10]. Furthermore ~~OD~~ opioid dependence has been associated with atherosclerotic disease in both the coronary [11-18] and cerebral [14 18] circulations. In a 33-year review of an opioid maintenance program in California in 2004, death from cardiovascular disease was noted to account for three times as many years of life lost in the treatment group than in the general population [18]. In 2007 an Iranian surgical group identified that opium dependent patients were at increased risk of requiring open coronary revascularization with an odds ratio of 1.8 after adjustment for other risk factors [15]. Moreover in this series there was a dose-response relationship between the amount of ~~opiate~~opioids consumed and the severity of the coronary disease. It was also shown that the coronary disease occurred two years earlier in ~~OD~~ opioid dependent persons than in controls [16]. This group further identified that opium use was the strongest cardiovascular risk factor in their population of men, with effects greater than traditional risk factors [17]. The sex specific finding in this study was felt to be related only to a reduced prevalence of opium consumption amongst females.

In reviewing over 800 fatal cases of ~~opiate~~opioid toxicity in Australia in 2006 Darke et al. found that 17% of decedents over the age of 44 years had coronary stenoses in excess of 75% [12]. Similarly when comparing 1193 post mortem cases of heroin and methadone toxicity the Sydney group found 2-3 times elevated adjusted odds ratios of ventricular hypertrophy, and myocardial, interstitial and perivascular fibrosis in the methadone treated group, consistent with the effects of more consistent ~~opiate~~opioid agonism in such patients [11]. In a 21-year follow-up of 42,676 patients undergoing maintenance opioid agonist treatment in Australia, including 425,998 person-years ~~in 2009~~, Degenhardt et al. identified cardiovascular death as

For peer review only

occurring 2.2 times as often as in the wider community [13]. Indeed in this study elevated rates of death with severe organ dysfunction were noted which appeared to act as a backdrop to the very high rates of death by overdose generally reported in such cohorts (see Web Appendix 6 of this citation).

Field Code Changed

In 2012, a prospective population based study of 50,045 people including 234,098 person-years reported hazard ratios of death from coronary artery disease (CAD) of 1.9-fold (95%C.I. 1.57-2.29) and of cerebrovascular disease (CeVD) of 1.68-fold (C.I. 1.29-2.18) [14]. Both were worse in females (CAD: male adjusted HR (AHR) 1.58, female AHR 2.90; CeVD male AHR 1.49, female AHR 1.97). An earlier study from Iran did not confirm the cardiovascular salience of opioid exposure amongst females as compared to males, but the number of exposed females in this study was small, which likely compromised the power of the analysis to detect an efffect [17]. Furthermore recent reports of 1,263 patients found elevated rates of vascular age in patients treated with methadone compared to other agents [19] and improved vascular ages accompanying the long term drug-free condition [20].

Field Code Changed

Formatted: Do not check spelling or grammar, Superscript

Field Code Changed

Field Code Changed

One large case control study found a dose response relationship between opium exposure and the severity of angiographically assessed coronary artery disease in a diabetic population [21].

Field Code Changed

Moreover ~~opiate~~opioid patients dependent on short acting ~~opiate~~opioids such as morphine, oxycodone and heroin undergo the clinical syndrome of ~~opiate~~opioid withdrawal several times daily. This syndrome is a hyper-adrenergic state characterized by neurological and cardiovascular stimulation which in its more severe manifestations includes hypertension and tachycardia [22] and a reduction of subendocardial perfusion [23]. This negative aversive experience is one of the key reasons many ~~opiate~~opioid dependent patients (ODP) continue to use narcotics.

Field Code Changed

Field Code Changed

Important as the above collected findings are, other evidence suggests that the impact of elevated vascular age has implications on the health and ageing of the organism as a whole beyond those limited primarily to the cardiovascular system. It has been repeatedly proposed that vascular ageing is a principal component of biological organismal ageing, by virtue of the contribution made by cardiovascular causes to the overall death rate in developed nations, and also the importance of the vascular niche common to many stem cell beds [24-25]. This view closely accords with the finding in many of the above studies and particularly that by

Field Code Changed

For peer review only

Degenhardt et al [13] that elevated rates of overdose occur on the background of advanced disease in other organs [1 12 18]. OpiateOpioid dependent patients have many stigmata of accelerated ageing including increased rates of: hair greying, erosion of alveolar bone from chronic periodontitis [26], osteoporosis [27], chronic respiratory disease [11 13 18], chronic renal disease [11], diffuse senescent-mimetic immune stimulation [5-6 28], diabetes [3], neuropsychiatric disorders, multi-system disease [11-12], reduced circulating stem cell counts [10], and many malignancies [13 29]. Thus the call has been made for geriatricians to be appointed to the care of opiateopioid dependent patients beyond the age of 50 years, due to the high prevalence of multi-system disease [1].

Field Code Changed
Field Code Changed
Field Code Changed
Field Code Changed
Field Code Changed
Field Code Changed
Field Code Changed
Field Code Changed
Field Code Changed
Field Code Changed
Field Code Changed
Field Code Changed

The exciting possibility therefore arises that an improved understanding of the increased and accelerated profile of degenerative disease seen in opiateopioid dependent patients may enhance our understanding, and in time our management, of age related disorders in the rest of the community.

As this clinic sees both opiateopioid ODPdependent patients and the general community, we were ideally situated to compare these two groups. The availability of the mobile SphygmoCor system from Atcor in Sydney implies means that a host of central cardiovascular data is available to the clinician in a non-invasive and rapid manner, providing quantitation of important subclinical endophenotypes of central vascular stiffness and cardiovascular and therefore organismal ageing. The central investigative question to be prospectively assessed was whether there were changes in major central cardiovascular parameters in the opiateopioid dependent group, and if so are they robust to multivariate adjustment for other confounding factors. Several earlier papers from our group have considered this issue in both cross-sectional [30-31] and longitudinal [32-33] studies in each sex separately and drawn conclusions generally confirmatory of this hypothesis. It has been shown that the type of opioid pharmacotherapy affects the severity of opiateopioid related potentiation of arterial stiffness [19], and that induction of the drug free state after implantable opiateopioid antagonists reverses this finding [20], and that opioid withdrawal is also associated with elevation of cardiovascular age and arterial stiffness parameters [23]. However a formal comparison of the effects by gender has not been possible in these earlier reports as they primarily considered single sex data. Gender comparison therefore forms the major focus of the present report which is a compilation of much of this earlier data.

Field Code Changed
Field Code Changed
Field Code Changed
Field Code Changed

Formatted: Do not check spelling or grammar, Superscript

For peer review only

For peer review only

Methods

Patient Selection. Control subjects (N=576) were recruited opportunistically from patients presenting for health examination checks for insurance or employment medical examinations or from patients presenting with minor health problems such as ear blockages, minor psychological disorders, or University students. Patients with known cardiovascular disorders such as hypertension, renal disease or diabetes or diseases known to perturb the CVS subacutely such as infections or pregnancy, were excluded. OpiateOpioid dependent patients (N=687) were also recruited opportunistically and were under treatment with either methadone (N=71), buprenorphine (N=593) maintenance, or naltrexone implant (N=23). All were sampled opportunistically at the time of their clinic visits May 2006 - December 2012.

Patient Treatment. OpiateOpioid dependent patients maintained on, methadone or buprenorphine were managed by accepted clinical algorithms by their usual health providers for their drug dependency. The naltrexone implants used for the management of heroin dependence were manufactured by "Go Medical" industries in Perth, Western Australia under Commonwealth of Australia Therapeutic Goods Administration (TGA) GMP conditions and administered under the compassionate Access arrangements of the Special Access Scheme authorized by the TGA as described elsewhere [34]. Patients were recalled for re-testing at two and five years post baseline assessment, and assessed opportunistically. Any patient with a known chronic or subacute cardiovascular condition were excluded from participation. Frequency and quantity of substance use by substance type (inclusive heroin) was assessed at the initial time and subsequently. Other opiateopioids were converted into morphine equivalents, and then into heroin equivalents at the rate of 1g of street heroin = 0.5g morphine. The duration of opiateopioid use was counted from the time of first use. If it became known that alcohol had been consumed in the few hours prior to the test or that stimulants had been used in the three days prior, that day's studies were excluded.

PWA Studies. Patients were positioned supine for the performance of the test and allowed to rest for five minutes prior to the test. Consumption of food, drink, alcohol or tobacco was not restricted, but patients were not allowed either to sleep or talk during the testing procedure. Pulse wave analysis by applanation tonometry was performed on the right radial artery unless

For peer review only

it was unavailable (due to trauma, surgery or congenital causes). The brachial blood pressure was taken in the opposite arm using the oscillometric Omron HEM 907 device. The data was collected and analyzed by the SphygmoCor software. Patients were studied in quintuplicate, and adequate studies were averaged for that day. Acceptable studies had an Operator index of greater than 70%, and were not inconclusive. Details of patients' prior drug use was also taken at the time of the study performance and entered into the database. Time in days was measured from the time of the first PWA study.

The SphygmoCor software used for the PWA studies calculates a number of major indices including Vascular Reference Age (RA), Chronologic Age (CA), Central Augmentation Pressure at Heart Rate 75 (C_AP_HR75) the Central Augmentation Pressure / Pulse Height Ratio at Heart Rate 75 (C_AGPH_HR75) also known as the Augmentation Index, Central Pulse Height (C_PH), Central Augmentation Load (C_AL), Central Augmentation Time Index (C_ATI), Peripheral–Central Pulse Pressure Amplification Ratio (PPAmpRatio), Maximal Peripheral Rise of dP/dT (P_MAX_DPDT), Central Systolic Pressure (C_SP), Central Diastolic Pressure (C_DP), Central End Systolic Pressure (C_ESP), Central Mean Pressure (C_MEANP), the Central Diastolic Time Index (C_DTI), the Central Tension Time index (C_TTI), and an index of subendocardial perfusion known variously as the Subendocardial Perfusion Ratio (SEVR), the Central Stroke Volume Index (C_SVI) or the Buckberg ratio defined as the C_TTI / C_DTI.

Statistics. Data are presented as the mean ± S.E.M. Categorical data were studied using "EpiInfo" 7.0.8.3 using the Chi squared test obtained from the CDC Atlanta, Georgia or the Fisher Exact test if the numbers in a cell were less than 20. Continuous categorized data were analyzed in "Statistica" 7.1 from Statsoft, Oklahoma using Student's t-tests. T-tests with separate variances were used where Levene's test was significant. Continuous variables such as RA, CA, RA/CA, variously corrected RA, SP, Weight, HDL, BMI, and systolic pressure were log transformed in accordance with normality assumptions based on the Shapiro-Wilks test. High sensitivity CRP and the heroin dose were arcsinh transformed which is a similar transform to logarithmic, but accepts arguments of zero. Time (as days) was measured from the time of the initial PWA study. The time since the last cigarette was recorded and analyzed as a factor with cut-off points at 30, 60, 120, 180 and > 180minutes, and a non-

For peer review only - http://bmjopen.bmj.com/site/about/guidelines.xhtml

smoking category. For some analyses lifetime heroin exposure was cut into quartile categories with divisions at 0, 0.01, 2.49, 6.59 and 350 g-years respectively, yielding 583, 207, 226 and 227 patients in each group respectively. Multiple regression was performed in "R" 2.15.2 obtained from the University of Melbourne Central "R" Archive Network mirror. Mean model estimates for various groups were prepared by inserting the mean parameter values into the untransformed final models. Multiple linear regression of truncated variables such as RA (minimum value of 20, maximum value of >80) were analyzed using tobit multiple regression techniques from the Applied Econometrics library in "R" (AER). RA's of individual studies returned as >80 were assigned as 88. ~~Random effects were assigned to unity and the patient identification code.~~ Time-dependent repeated measures analysis was performed using non-linear mixed effects (nlme) regression module in "R" using restricted equation maximum likelihood (REML) techniques. Random effects were assigned to unity and the patient identification code. When nlme models were compared they were converted to maximum likelihood (ML) methods to allow Anova testing. In each case model reduction was performed manually by the classical method involving removing the least significant term. The log-likelihood value was abbreviated to LogLik. Graphs were prepared with reshape2 and ggplot2. Where loess curves were fitted, the span used was 0.95. T-tests were two-sided. P<0.05 was considered significant.

Ethical Approval. All patients provided their informed consent to participate in the study and for their medical treatment. The study was approved by the Human Research Committee of the Southcity Family Medical Centre, Brisbane, Queensland, Australia, which is registered with the National Health and Medical Research Council.

For peer review only

Results

576 control patients were compared with 687 ~~opiate~~opioid dependent patients. The mean ages were ~~29.96~~30.0+0.45 and 34.5~~5~~+0.3~~2~~ (mean + S.E.M.) years (t=-8.262, dF=1082.22, P<0.0001, Supplementary Table 1~~.~~), with 401 (69.62%) and 465(67.69%) males respectively (Chi^2 = 0.54, P=0.46). 15.97% and 92.29% smoked tobacco (Chi^2=746.56, P<0.0001). The mean CA of the whole group was 32.45+0.~~27~~3years. The mean CA of the males was 32.14+0.3~~3~~ and of females 33.2~~16~~+0.5~~3~~ years. 7.47% and 5.97% of the control and opiate dependent groups were aged over 50 years respectively (Chi Squ.=1.13, P = 0.29). Amongst the males these ratios were 5.74% and 6.24% (Chi Squ. =0.1), and amongst the females they were 11.43% and 5.41% (Chi Squ. = 4.79, P = 0.029, Fisher Exact Test 0.19-0.98; pooled data, Corr. Chi Squ = 3.8, P = 0.051). Racial stratification was not performed as our population is around 90% Caucasian in background. Other baseline biometric, drug use and laboratory values are given in Supplementary Table 1. Patients were studied longitudinally on 710 and 1,305 occasions. The period of repeat study, measured from the time of the first PWA study, was 624.11 + 18.74 days (range 1-2382 days; 495 (60.36%) studies performed beyond 360 days). Details of the numbers of repeat studies are given in Supplementary Table 2.

Comparative PWA values are given in Supplementary Table 3~~2~~. Most augmentation values and the C_SP and C_ESP are noted to be higher in the ~~OD~~ opioid dependent group consistent with their being an average of 4.59 years older.

Figure 1 shows different vascular age and augmentation parameters corrected for various features, against CA. This figure shows that the BMI was lower in the ~~OD~~ opioid dependent group than the controls both as a factor (factor est.=0.0719, est. S.E. = 0.0327, t=2.197, P=0.0282) and in interaction with CA (BMI: CA est.=-0.0023, est. S.E. = 0.0009, t=-2.350, P=0.0189; model F=34.94, dF=3, 1258, P<2.2E-16). In the longitudinal series BMI was lower as a factor (est.=-0.0174, dF=812, P=0.0470; model AIC= -3722.55, LogLik=1867.27). This result may be the effect of cumulative diversion of resources over time. Figure 2 shows similar indices over time. Supplementary Table 4~~3~~ presents the results of the cross-sectional data where BMI corrected RA is regressed against CA and addictive status. In the whole group both ~~OD~~ opioid dependency status (P=0.0265) and the CA: addiction interaction (P=0.0205) are significant. Similar results were found in each sex. Similar results were also

For peer review only

found in the longitudinal study when the RA/CA ratio was regressed against CA, time and dependency status both for ~~OD~~ opioid dependency as a factor (P=0.0102) and in interaction with CA (P=0.0028; details in Supplementary Table 4).

Inclusion of tobacco exposure along with ~~opiate~~opioid exposure in these models did not alter these findings (data not shown).

As shown in Supplementary Figures 1~~3~~ and 2~~4~~, these changes were found to be more pronounced with CA in females. These data are presented corrected for the changes in BMI in Figures 3~~5~~ and 4~~6~~. Details for these significant differences are presented in Table 1.

Figure 5~~7~~ shows these data by sex with loess curves fitted (span=0.95). This strongly suggests that an inflection point is reached about the age of 30 in females, which is not seen in males until about 40 years of age. This was quantified by dichotomizing CA from age 41 in males and 29 in females. As shown in Table 2 in males in the older age category, the addictive status and their interaction were significant (P= 0.0010, 0.0021 and 0.0008 respectively), whilst in females ~~<55 years~~ the older age: addiction interaction was significant (P=0.0195, analysis restricted to women <55 years).

The regression changes are quantified for some of the major indices of arterial stiffness in cross-sectional data in Supplementary Table 6~~5~~ (all P<0.005). Similar significantly different sex specific data were found in the longitudinal dataset when the RA was regressed against CA, time, sex and addictive status (most P<0.02, Supplementary Table 7~~6~~).

When the mean sex-specific values for CA and BMI are inserted into the equation of Table 3 the mean vascular ages of the control groups amongst all patients, males and females was 35.42, 35.61 and 34.45 years, whilst the corresponding ages of the ODP's was 37.24, 36.31 and 39.18 years, representing incremental gains of 5.14%, 1.97% and 13.43%.

When quartiles are defined for lifetime heroin exposure the results shown in Figure 8 are found. Similar effects longitudinally are shown in Supplementary Figure 3~~1~~. These results are quantitated in Supplementary Table 8~~7~~ for both cross-sectional and longitudinal data compared to non-exposed controls. In the cross-sectional analysis the RA/CA ratio was regressed against the CA and ~~Opiate~~Opioid exposure quartile. In the longitudinal analysis the

For peer review only

RA/CA ratio was regressed against time, CA and the ~~opiate~~opioid exposure quartile. Whilst the CA: Level 3 ~~opiate~~opioid exposure interaction is of borderline significance in all patients and females (P=0.0546 and 0.0568, but not in males) in the longitudinal study the age: level 2 exposure is significant in all patients and males (P=0.0003 and 0.0110) and level 3 ~~opiate~~opioid exposure approaches significance in females both as a factor and in interaction with age (P=0.0501 and 0.0547). Supplementary Table ~~8~~9 presents the results of a longitudinal regression of age-corrected indices of cardiovascular stiffness against time and ~~opiate~~opioid exposure quartile by gender, and notes a highly significant effect by exposure quartile in both genders.

The data was formally interrogated for a trend between the different quartiles of lifetime ~~opiate~~opioid exposure using the contrasts polynomial function in “R”. Significant contrasts between the various quartiles for lifetime ~~opiate~~opioid exposure for the age: ~~opiate~~opioid exposure interaction was indeed demonstrated in all patients (est. = 0.1657, S.E. = 0.0787, t = 2.104, P = 0.0356; Model Adj. $R^2 = 0.4265$, F = 133.0, dF = 7, 1235, $P < 2.2\times10^{-16}$), such that moving up a quartile level at age 60 increased the modelled RA by 8.42% to 65.051 years, and movement from the first (non-exposed) quartile to the highest (level 3) quartile increased the modelled RA 25.26% to 75.154 years.

Some environmental exposure – pathology relationships have been shown to be power functions of the duration of exposure and are therefore particularly sensitive to the periods for which exposure occurs [35]. Since ~~opiate~~opioid administration is often prescribed and recommended as being of indefinite duration this is particularly relevant to the present discussion. Therefore a detailed study of the power relationships of the duration of ~~opiate~~opioid administration was undertaken. Table 3 shows that simple regression models of RA/CA against the dose-duration interaction were significantly related to linear, quadratic, cubic and quartic terms in ~~opiate~~opioid exposure duration. Moreover when RA/CA was regressed against a model including interactive terms in linear, quadratic, cubic and quartic terms, all four were significant. When these models were compared by ANOVA studies the quadratic (AIC=82.888) was noted to be significantly better than the linear, cubic or quartic models (Anova tests, all P<0.05), but not significantly different from the combined model (AIC=83.731, P=0.87; Supplementary Table ~~9~~10).

Field Code Changed

For peer review only

When a similar exercise was performed in the longitudinal dataset, similar results were obtained (Supplementary Table 110). In this study RA/CA was regressed against time and the opiateopioid exposure quartile. As a combined linear-quadratic-cubic-quartic model failed to converge, the highest order model used was linear-quadratic-quartic. Again quadratic and quartic terms in exposure duration persist in the final model. In this case the quadratic model (AIC=1040.85) was significantly superior to all the others but was not significantly better than the linear-quadratic-quartic model (AIC= 1041.09, P=0.082; Supplementary Table 121).

The data was interrogated for the presence of a dose-response relationship directly by examining continuous terms in opiateopioid dose and duration regressed against the RA/CA ratio in both datasets. As shown in Table 4 the dose-duration interaction is significant in all patients considered together, in males and in females (P=0.0.000037, 0.00029 and 0.0236) in the cross-sectional dataset, whilst in the longitudinal dataset the parallel results are P = 0.0004, 0.0008 and 0.0127.

The final step of the analysis was to consider whether the significance of the opiateopioid dose and duration might persist after multivariate adjustment for all known cardiovascular risk factors. In preliminary analyses lipidic factors were invariably eliminated during the model reduction process. Moreover, as discussed in the Introduction, opiateopioids themselves are likely to perturb both lipid function, and also have a robust immunostimulatory action. Therefore including immune markers is likely to over-correct the model. Similarly when the time since the last cigarette consumption was included in the cross-sectional and longitudinal models it was eliminated during model reduction. For these reasons the final cross-sectional formula used regressed RA/CA against interactive terms in opiateopioid dose and duration, height, tobacco consumption, systolic blood pressure and weight. The longitudinal model was similar but had an additive term in weight. These were the terms which remained significant in the models in preliminary exploratory analyses for log (RA). Further consideration of the clinical justification for the choice of this five way interaction is included in the Discussion section.

The results shown in Table 5 were found for the cross-sectional data. This table shows that significant interactions including opiateopioid dose-duration terms are found in all patients

(P=0.0261) and in males (P=0.0017; Adj. R^2=0.0815), whilst in females this interaction approaches significance (P=0.0516). When a similar exercise is performed on the longitudinal data, significant interactions are seen from P = 0.0011, 0.0013 and 0.0073 and above in each group respectively (Table 6).

Supplementary Table 13~~2~~ presents the results of final models in which complete multiple regression was performed in the cross-sectional dataset for all pertinent variables. (Log) RA/CA was regressed against interactive terms in ~~opiate~~opioid dose and duration, height, tobacco use, systolic pressure and high sensitivity CRP together with additive terms in weight, cholesterol and HDL. ~~Opiate~~Opioid dose and duration persist in the final model in males and in all patients, but not in females. Interestingly CRP features in most of the terms of these final models.

For peer review only

Discussion

The major findings of this study are that indices of vascular age and arterial stiffness are worse in ~~opiate~~opioid dependence compare to ~~opiate~~opioid naïve controls with mean calculated ages elevated by 1.97% in males and 13.43% in females. The effect was thus more marked in females. A significant effect was found on both vascular age and augmentation index by exposure quartile after correction for CA and BMI. The RA/CA ratio was found to be related to power functions of the ~~opiate~~opioid duration of exposure in both cross-sectional and longitudinal analyses. A dose-response relationship was demonstrated with lifetime ~~opiate~~opioid exposure. And the effect of ~~opiate~~opioid exposure was robust, and remained after ~~multivariate~~multiple adjustment in cross-sectional and longitudinal studies.

These findings should be interpreted in the light of the relatively modest degree of ~~opiate~~opioid exposure to which these patients were exposed. Whilst the dose and duration of ~~opiate~~opioids used by patients in this study is typical of that seen in many published series, the treatment of these patients was mostly with the relatively mild partial agonist buprenorphine, with only modest doses employed (mean 6.98$\pm$0.21mg): this dose level being much lower than the commonly recommended (18-24mg) [36]. Moreover buprenorphine has been shown to be more mild in its cardiovascular effects than the full agonist agent methadone . For this reason the results reported herein should be seen as a lower bound on the effects which may be observed.

Field Code Changed

The findings in relation to central vascular stiffness and age being related to power functions of the treatment duration is of great importance. Chronic ~~opiate~~opioid agonist treatment is often seen as relatively benign and partly for this reason is frequently recommended for very long term or indefinite administration [37-38]. However the demonstration here that the effects likely compound with time have major clinical practice implications, and have not been previously noted. The 1.97% effect described here for males, compounded over 30 years equates cumulatively to 179.5% gain and in diminution to 44.95% loss. Similarly the 13.43% gain seen in females compounded over 30 years is a 4349.02% gain and a 98.66% loss in reduction. The effects therefore of these process maintained over the long term on stem cell diminution or immune stimulation are potentially very considerable.

Field Code Changed

For peer review only

The exacerbation of the effect in females was clearly demonstrated in Figures 3-7, Tables 1 and 2, and Supplementary Tables 4 and 5. This finding has, to the authors knowledge, only been reported previously by an Iranian group[14]. If one looks closely at Figure 7 several fascinating findings emerge. The vascular age algorithm used by the SphygmoCor software is based upon the augmentation index C_AGPH_HR75. This index has a clear curvilinear convex upward relationship to CA as shown in Figure 7 in the lower panels, unlike the C_AP_HR75 which is directly linear[39]. Hence the reduction at the higher ages on the C_AGPH_HR75 curve may be somewhat artefactual. If one looks at the upper panels of Figure 7 the rise in RA with age in ODP may be seen to be broadly exponential in form, if the decline in slope above age 45 years is discounted. In females it appears that the rise in RA occurs some 10 years prior to that seen in males, a finding emphasized by the middle panels. This fits with a dramatic clinical transformation which is seen in ~~OD~~ opioid dependent females at around age 30, and in males at about age 40. Whilst the cause of this is unknown it may possibly be related to a more aggressive immune system in females which is in part hormonally mediated[40].

Field Code Changed

Field Code Changed

Field Code Changed

Some consideration of the comparability of the control and opiate dependent groups is appropriate. Significant differences were found between the control and opiate dependent groups in relation to chronological age, weight, BMI, smoking, cholesterol, LDL and hepatic and inflammatory laboratory markers, but these were controlled for by various the multiple regression techniques. The higher mean weight in the opiate dependent group may have been related in part to their higher age, and in part to the orexigenic effects of opiate agonism[41], whilst serial resource diversion may explain the slower rate of gain of weight in this group over time. Higher rates of tobacco consumption may be related to either the common features of the addictive lifestyle or the pro-consumptive effects of opioid aalogies[42]. Significantly more of the females in the control group (11.4% vs. 5.41%) were aged over 50 years (P=0.029). However as no data was collected relating to hormonal status with regard to the menopause it is not possible to remark on the relative hormonal status of the two groups. Opiate dependency is well known to be associated with hypothalamic hypogonadotrophic oligoamenorrhoea, so that the age-related hormonal suppression in the control group was somewhat balanced by the drug related hormonal suppression in the opiate dependent group. Clarification of this issue must await further studies.

Formatted: Do not check spelling or grammar, Superscript

Formatted: Do not check spelling or grammar, Superscript

For peer review only

Some consideration is appropriate of the clinical justification for the five-way interaction which was presented in the final multiple regression models of RA (Tables 5 and 6). Height is a key variable which relates to many techniques of measuring of arterial stiffness since it is related to the timing of arterial wave reflections, and it is hardly surprising that this remains in the final model[43-44]. Height and weight will naturally interact and indeed the BMI is one formula which formalizes such interactions. The opioid dose-duration exposure is a standard way in which to account for lifetime opioid exposure, and has been used by WHO[35] and NIH[14] investigators amongst others. Other factors including laboratory parameters were not present in the final models. Therefore it is not surprising that this cluster of factors remains in the final model.

On the other hand CRP was prominent and present in most terms in the final model when RA/CA was interrogated by multiple regression (Supplementary Table 13) suggesting that innate immune processes may be implicated in the observed opioid-vasculopathy effect. The age-related course of high sensitivity CRP has been described in several earlier reports in our patient cohort[6 28 45]. Supplementary Table 1 shows significant elevation of CRP, ESR, serum globulins, and circulating lymphocytes and monocyte numbers in the opiate dependent group. Consideration of the very flat age-dependent trajectories of these parameters in controls in our earlier reports shows that this effect is much greater in opiate dependence than might be expected on the basis of the higher chronologic age in the opioid dependent group alone. This is in turn consistent with the important chronic inflammatory basis of many vascular diseases[46-48] and the immune theory of organismal ageing[46 49].

As noted above, vascular age is frequently said to be a surrogate for organismal ageing. The present data are consistent with that view, and also with the literature cited of accelerated ageing in these patients. The present technique provides a mechanism to quantify these changes at the whole human level.

As no structural im~~aging~~aging techniques were performed it is not possible to say with certainty if the observed elevations in vascular stiffness were associated with fixed atherosclerotic lesions. However a large and weighty literature confirms the relevance of significance of elevations in vascular stiffness[43-44 50-52~~41-45~~]. Structural correlation must await further work.

Formatted: Font: 12 pt
Formatted: Font: 12 pt
Formatted: Font: 12 pt
Formatted: Font: 12 pt
Formatted: Font: 12 pt
Formatted: Font: 12 pt
Formatted: Font: 12 pt
Formatted: Font: 12 pt
Formatted: Font: 12 pt
Formatted: Do not check spelling or grammar, Superscript
Formatted: Do not check spelling or grammar, Superscript
Formatted: Do not check spelling or grammar, Superscript
Formatted: Do not check spelling or grammar, Superscript
Formatted: Subscript
Field Code Changed

For peer review only

The consistency of the present results with the previously reported data from various national and international groups [11-18 53] is clear in that we have confirmed the exacerbation of cardiovascular ageing and arterial stiffness in opiate dependency . However these earlier results have been extended by demonstrating a measurable major subclinical endophenotype of accelerated ageing antemortem, confirmed a dose-response relationship, demonstrated for the first time the significance of the improved model quadratic in opiate exposure duration, and shown that the opiate-related effect is robust to adjustment for known cardiovascular risk factors in both sexes. Moreover we confirmed that the effect is worse in females [14], and extended this result by showing that the differential effect commences in women over a decade earlier than in men (29 vs. 41 years). In that the PWA technique directly relates increased arterial stiffness to cardiovascular and therefore organismal ageing the present detailed studies link advanced cardiovascular age to many other indices of accelerated ageing in opioid dependency as have been shown by this [6-7 10 26 28 45 54-58] and other [59] groups. As such the present report is a meaningful and important extension of earlier reports from this project on the cardiovascular implications of opioid dependency [30-33], its treatment [19] and withdrawal [23] and the antagonist-induced reversal of these effects [20]. Hence the phenomenology of the present descriptive report is consistent with, and extends, extant reports in the published literature.

Formatted: Do not check spelling or grammar, Superscript

Formatted: Do not check spelling or grammar, Superscript

Formatted: Do not check spelling or grammar, Superscript

Formatted: Do not check spelling or grammar, Superscript

Formatted: Do not check spelling or grammar, Superscript

Formatted: Do not check spelling or grammar, Superscript

Formatted: Do not check spelling or grammar, Superscript

Formatted: Do not check spelling or grammar, Superscript

Whilst this study is not designed to be mechanistic in nature some brief comment on this issue may be appropriate, in part for its relevance to the consideration of possibly aetiologically causal pathways. S~~S~~ome of the most interesting questions to emerge from the present work relate to the possible mechanisms underlying the plethora of changes which have been identified in ~~OD~~ opioid dependence as cardiovascular risk factors, as potentially atherogenic processes and as accelerants of ageing processes. ~~Opiate~~Opioids have been linked with blockade of the cell cycle at cyclin dependent kinases 2, 4 and 6 by P16[INK4A] and P21[WAF1/CIP1] [60-61~~46-47~~]. P16 is the gene product of CDKN2A located at the senescence locus on Chromosome 9p21.3 which is a hotspot for many GWAS studies, and from which three senescence related proteins and one senescence associated long non-coding RNA are transcribed [62~~48~~]. Furthermore ~~opiate~~opioid agonists have been shown to directly ligate the myeloid differentiation factor 2 (MD2)- toll-like receptor (TLR) 4 heterodimer leading to the downstream activation of the numerous powerful pathways linked to stimulation of the

Field Code Changed

Field Code Changed

endotoxin receptor [63~~49~~]. Indeed systemic endotoxaemia was recently shown to modulate the expression of 6,025 genes [64~~50~~]. Some of the pathways activated downstream by TLR4 signalling include the cyclooxygenase, lipooxygenase, MAP kinase (JNK and P38), AP-1, inducible nitric oxide synthase (iNOS), phosphoinositol-3 kinase (PI3K), and TGFβ pathways [65~~51~~], many of which have been noted to be stimulated within the first few minutes of TLR4 ligation [66~~52~~], to be stimulated by ~~opiate~~opioids [63 65~~49-51~~], and to be associated with potentiating the effects of ageing [49 67~~53-54~~]. Many of these immune products directly exacerbate the stem cell blockade [68~~55~~]. This then implies that ~~OD~~opioid dependency ~~-~~may be conceptualized as chronically, if largely subclinically, ill.

It is very exciting that new research has identified that these processes also act particularly in proopioimelanocortin (POMC) neurons of the paraventricular and arcuate nuclei of the mediobasal hypothalamus which are key determinants of the body's metabolic homeostasis, glucostatic and lipostatic functions, energy balance, appetitive drives, sympathoadrenal activation state, and even lifespan modulation in some organisms [69-71~~56-58~~], and that derangements in this region cause metabolic syndrome and its numerous related disorders in model organisms including mammals and likely also humans [72~~59~~]. These workers have shown that activation of the MD2/TLR4/IKKβ/NF-κB cascade induces endoplasmic reticulum, mitochondrial and autophagic stress, which along with reduced neurogenesis, increased POMC neuronal apoptosis [70~~57~~], and glial activation and reactive gliosis [72-73~~59-60~~] underlie these numerous disorders and derangements. Whilst this research focuses on TLR4 ligation of unsaturated fatty acids and oxidized low density lipoproteins, ~~opiate~~opioids have been shown to act similarly as xenobiotics through MD2-TLR4 [74~~61~~], and ~~opiate~~opioid agonists have been shown to produce POMC cell hyperpolarization and relative refractoriness [42~~62~~], whilst ~~opiate~~opioid antagonists have the reverse effect [75~~63~~]. Small molecule TLR4 inhibitors are under development for several therapeutic applications [76-77~~64-65~~]. The confluence then of these two important lines of recent research has the potential to produce major conceptual and therapeutic advances in this field. A clear understanding of direct links from xenobiotic-TLR4-NF-κB signalling to sympathoadrenal tone and hypothalamic lifespan control has the potential to revolutionize the direction of therapeutic endeavours in the treatment of ~~opiate~~opioid dependence.

It is fascinating that the promoters for two of the key stem cell genes, Oct4 and Nanog have response elements in their promoters for STAT-1 and -3 which are commonly produced as a

Field Code Changed
Field Code Changed
Field Code Changed
Field Code Changed
Field Code Changed
Field Code Changed
Field Code Changed
Field Code Changed
Field Code Changed
Field Code Changed
Field Code Changed
Field Code Changed
Field Code Changed
Field Code Changed
Field Code Changed

result of cytokine signalling, particularly by gp-130 cytokines such as IL-6 and leukaemia inhibitory factor (LIF)[78-79][~~66-67~~]. Furthermore there is three way cross-talk between stem cell and metabolic –immune genes including STAT-3 and PPARγ[80][~~68~~]. Other cytokines, such as IL-1 have been noted in ~~OD~~ opioid dependency[8] and are usually indicative of inflammasome activation, and fractalkine and MCP-1/CCL2 have also been found to be elevated and are relevant both to neuropathological and atherogenic processes[63][~~49~~]. As TLR4 shares many ligands and co-chaperones with the receptor for advanced glycation end (RAGE) products this auto-amplificatory system may also be involved[81-84][~~69-72~~].

Field Code Changed

Field Code Changed

Field Code Changed

Field Code Changed

Field Code Changed

This study had various strengths and limitations. The application of sophisticated cardiovascular measurement technology to the problem of drug addiction is unique to this group. Extended follow-up and recruitment of ~~opiate~~opioid dependent patients is also notoriously difficult. The statistical techniques employed herein are advanced and robust. Design strengths include its complimentary cross-sectional and longitudinal design. Limitations of the present work relate to difficulties quantitating frequently variable amounts of past drug use in the absence of a formal measurement instrument. Use of a formal instrument such as the addiction severity index, and spot tests for drugs and alcohol are indicated in future replications of this study. Employment of structural tests, and mechanistic studies to investigate pathophysiological pathways, would be advisable in future iterations of this work.

In summary the present study documents elevated vascular stiffness and age associated with ~~opiate~~opioid use which is robust to ~~multivariate~~multiple adjustment to other known risk factors. A strong dose-response effect was noted, and the duration of treatment administration was noted to be particularly important, being related to elevations in vascular age of the first second, third and fourth order. This finding indicates limitation of treatment duration wherever clinically possible. The effect was also worse in females. Overall this study raises many further mechanistic questions which indicate further study on this complex but important model condition.

For peer review only

Acknowledgements

The authors would like to thank Dr Mervyn Thomas of Emphron for assistance with the statistics and graphical design.

Conflict of Interest

Nil declared.

Sources of Funding

Nil.

Statement of Authorship

This work is the work of the authors in its entirety.

For peer review only

References

1. Rosen D, Smith ML, Reynolds CF, 3rd. The prevalence of mental and physical health disorders among older methadone patients. *Am J Geriatr Psychiatry* 2008;16(6):488-97.
2. Cooper OB, Brown TT, Dobs AS. Opiate drug use: a potential contributor to the endocrine and metabolic complications in human immunodeficiency virus disease. *Clin Infect Dis* 2003;37 Suppl 2:S132-6.
3. Ceriello A, Quatraro A, Giugliano D. Opiate addict as diabetic patient? *Diabetes Care* 1988;11(5):443.
4. Kolarzyk E, Pach D, Wojtowicz B, Szpanowska-Wohn A, Szurkowska M. Nutritional status of the opiate dependent persons after 4 years of methadone maintenance treatment. *Przeglad lekarski* 2005;62(6):373-7.
5. Brunton LL, Lazo JS, Parker KL, editors. *Goodman and Gilman's the Pharmacologic Basis of Therapeutics*. Eleventh Edition ed. New York: McGraw Hill, 2006.
6. Reece A. S. Relative and Age Dependent Stimulation of Soluble and Cellular Immunity in Opiate Dependence. *Journal of Addiction Medicine* 2012;6(1):10-17.
7. Reece A. S. High Sensitivity CRP in Opiate Addiction: Relative and Age-Dependent Elevations. *Cardiovascular Toxicology* 2012;12(2):149-57.
8. Neri S, Bruno CM, Pulvirenti D, Malaguarnera M, Italiano C, Mauceri B, et al. Randomized clinical trial to compare the effects of methadone and buprenorphine on the immune system in drug abusers. *Psychopharmacology (Berl)* 2005;179(3):700-4.
9. Reece A. S. Absolute and Age Dependent Elevations of Serum Calcium and Phosphate and their Products in Clinical Opiate Dependence. *J. Subs. Abuse* 2013;Accepted 27th November 2012.
10. Reece AS, Davidson P. Deficit of circulating stem--progenitor cells in opiate addiction: a pilot study. *Substance abuse treatment, prevention, and policy* 2007;2:19-28.
11. Darke S, Duflou J, Torok M. The comparative toxicology and major organ pathology of fatal methadone and heroin toxicity cases. *Drug Alcohol Depend* 2010;106(1):1-6.
12. Darke S, Kaye S, Duflou J. Systemic disease among cases of fatal opioid toxicity. *Addiction* 2006;101(9):1299-305.
13. Degenhardt L., Randall D., Hall W., Law M., Butler T., Burns L. Mortality among clients of a state-wide opioid pharmacotherapy program over 20 years: Risk factors and lives saved. *Drug Alcohol Depend* 2009;105(1-2):9-15.
14. Khademi H, Malekzadeh R, Pourshams A, Jafari E, Salahi R, Semnani S, et al. Opium use and mortality in Golestan Cohort Study: prospective cohort study of 50,000 adults in Iran. *BMJ (Clinical research ed* 2012;344:e2502.
15. Sadeghian S, Darvish S, Davoodi G, Salarifar M, Mahmoodian M, Fallah N, et al. The association of opium with coronary artery disease. *Eur J Cardiovasc Prev Rehabil* 2007;14(5):715-7.
16. Sadeghian S, Dowlatshahi S, Karimi A, Tazik M. Epidemiology of opium use in 4398 patients admitted for coronary artery bypass graft in Tehran Heart Center. *J Cardiovasc Surg (Torino)* 2011;52(1):140-1.
17. Sadeghian S, Graili P, Salarifar M, Karimi AA, Darvish S, Abbasi SH. Opium consumption in men and diabetes mellitus in women are the most important risk factors of premature coronary artery disease in Iran. *International journal of cardiology* 2010;141(1):116-8.
18. Smyth B, Hoffman V, Fan J, Hser YI. Years of potential life lost among heroin addicts 33 years after treatment. *Preventive medicine* 2007;44(4):369-74.
19. Reece A.S., Hulse G.K. Impact of Opioid Pharmacotherapy on Arterial stiffness and Vascular Ageing: Cross-sectional and Longitudinal Studies. *Cardiovascular Toxicology* 2013;13(3):254-66.
20. Reece A. S., Hulse G.K. Reduction in Arterial Stiffness and Vascular Age by Naltrexone-Induced Interruption of Opiate Agonism. *British Medical Journal - Open* 2013.

Formatted: Font: Bold, Underline, Do not check spelling or grammar

Formatted: Font: Not Bold, No underline
Formatted: Font: Not Bold, Italic, No
Formatted: Font: Not Bold, No underline
Formatted: Font: Not Bold, Italic, No
Formatted: Font: Not Bold, No underline
Formatted: Font: Not Bold, Italic, No
Formatted: Font: Not Bold, No underline
Formatted: Font: Not Bold, Italic, No
Formatted: Font: Not Bold, No underline
Formatted: Font: Not Bold, Italic, No
Formatted: Font: Not Bold, No underline
Formatted: Font: Not Bold, Italic, No
Formatted: Font: Not Bold, No underline
Formatted: Font: Not Bold, Italic, No
Formatted: Font: Not Bold, No underline
Formatted: Font: Not Bold, Italic, No
Formatted: Font: Not Bold, No underline
Formatted: Font: Not Bold, Italic, No
Formatted: Font: Not Bold, No underline
Formatted: Font: Not Bold, Italic, No
Formatted: Font: Not Bold, No underline
Formatted: Font: Not Bold, Italic, No
Formatted: Font: Not Bold, No underline
Formatted: Font: Not Bold, Italic, No
Formatted: Font: Not Bold, No underline
Formatted: Font: Not Bold, Italic, No
Formatted: Font: Not Bold, No underline
Formatted: Font: Not Bold, Italic, No
Formatted: Font: Not Bold, No underline
Formatted: Font: Not Bold, Italic, No
Formatted: Font: Not Bold, No underline
Formatted: Font: Not Bold, Italic, No
Formatted: Font: Not Bold, No underline
Formatted: Font: Not Bold, Italic, No
Formatted: Font: Not Bold, No underline
Formatted: Font: Not Bold, Italic, No
Formatted: Font: Not Bold, No underline
Formatted: Font: Not Bold, Italic, No
Formatted: Font: Not Bold, No underline
Formatted: Font: Not Bold, Italic, No
Formatted: Font: Not Bold, No underline

21. Hosseini SK, Masoudkabir F, Vasheghani-Farahani A, Alipour-Parsa S, Sheikh Fathollahi M, Rahimi-Foroushani A, et al. Opium consumption and coronary atherosclerosis in diabetic patients: a propensity score-matched study. *Planta Med* 2011;77(17):1870-5.
22. Charney DS, Redmond DE, Jr., Galloway MP, Kleber HD, Heninger GR, Murberg M, et al. Naltrexone precipitated opiate withdrawal in methadone addicted human subjects: evidence for noradrenergic hyperactivity. *Life Sci* 1984;35(12):1263-72.
23. Reece AS, Hulse GK. Elevation of Central Arterial Stiffness and Vascular Ageing in Opiate Withdrawal: Cross-sectional and Longitudinal Studies. *Cardiovasc Toxicol* 2012;13(1):55-67.
24. Chien KR, Karsenty G. Longevity and lineages: toward the integrative biology of degenerative diseases in heart, muscle, and bone. *Cell* 2005;120(4):533-44.
25. Lakatta EG, Wang M, Najjar SS. Arterial aging and subclinical arterial disease are fundamentally intertwined at macroscopic and molecular levels. *Med Clin North Am* 2009;93(3):583-604, Table of Contents.
26. Reece AS. Hair graying in substance addiction. *Archives of dermatology* 2007;143(1):116-8.
27. Kim TW, Alford DP, Malabanan A, Holick MF, Samet JH. Low bone density in patients receiving methadone maintenance treatment. *Drug Alcohol Depend* 2006;85(3):258-62.
28. Reece AS. Evidence of Accelerated Ageing in Clinical Drug Addiction from Immune, Hepatic and Metabolic Biomarkers. *Immun Ageing* 2007;4(1):6-15.
29. Randall D, Degenhardt L, Vajdic CM, Burns L, Hall WD, Law M, et al. Increasing cancer mortality among opioid-dependent persons in Australia: a new public health challenge for a disadvantaged population. *Australian and New Zealand journal of public health* 2011;35(3):220-5.
30. Reece A.S., Hulse G.K. Lifetime Opiate Exposure as an Independent and Interactive Cardiovascular Risk Factor in Males: A Cross-Sectional Clinical Study. *Vascular Health and Risk Management* 2013;9(55):551-59.
31. Reece A.S., Hulse G.K. Opiate Exposure Increases Arterial Stiffness, Advances Vascular Age and is an Independent Cardiovascular Risk Factor in Females: A Cross-Sectional Clinical Study. *World J. Cardiovascular Diseases* 2013;In Press(DOI: 10.4236/wjcd.2013.35056).
32. Reece A. S., Hulse G.K. Duration of Opiate Exposure as a Determinant of Arterial Stiffness and Vascular Age in Male Opiate Dependence: A Longitudinal Study. *Journal of Clinical Pharmacy and Therapeutics* 2013;In Press(DOI: 10.1111/jcpt.12121).
33. Reece A. S., Hulse G.K. Opiate Dependence as an Independent and Interactive Risk Factor for Arterial Stiffness and Cardiovascular Ageing - A Longitudinal Study in Females. *J Clinical Medical Research* 2013;5(5):356-67.
34. Reece AS. Psychosocial and treatment correlates of opiate free success in a clinical review of a naltrexone implant program. *Subst Abuse Treat Prev Policy* 2007;2:35-49.
35. Breslow N.E., Day N.E. *Statistical Methods in Cancer Research Volume II The Design and Analysis of Cohort Studies*. 1 ed. Lyon: International Agency for Cancer Research,, 1987.
36. Kacinko SL, Jones HE, Johnson RE, Choo RE, Huestis MA. Correlations of maternal buprenorphine dose, buprenorphine, and metabolite concentrations in meconium with neonatal outcomes. *Clinical pharmacology and therapeutics* 2008;84(5):604-12.
37. Hulse G., White J., Cape G., editors. *Management of Drug and Alcohol Problems*. Sydney: Oxford University Press, 2002.
38. Oviedo-Joekes E, Brissette S, Marsh DC, Lauzon P, Guh D, Anis A, et al. Diacetylmorphine versus methadone for the treatment of opioid addiction. *N Engl J Med* 2009;361(8):777-86.
39. SphygmoCor, editor. *Software Operator's Guide, Pulse Wave Analysis System, SCOR-Px*. Sydney: AtCor Medical, 2006.
40. Klein SL. Sex influences immune responses to viruses, and efficacy of prophylaxis and treatments for viral diseases. *Bioessays* 2012;34(12):1050-9.
41. Reece AS. Hypothalamic opioid-melanocortin appetitive balance and addictive craving. *Medical hypotheses* 2011;76(1):132-7.
42. Cowley MA, Smart JL, Rubinstein M, Cerdan MG, Diano S, Horvath TL, et al. Leptin activates anorexigenic POMC neurons through a neural network in the arcuate nucleus. *Nature* 2001;411(6836):480-4.

Formatted: Font: Not Bold, Italic, No
Formatted: Font: Not Bold, No underline
Formatted: Font: Not Bold, Italic, No
Formatted: Font: Not Bold, No underline
Formatted: Font: Not Bold, Italic, No
Formatted: Font: Not Bold, No underline
Formatted: Font: Not Bold, Italic, No
Formatted: Font: Not Bold, No underline
Formatted: Font: Not Bold, Italic, No
Formatted: Font: Not Bold, No underline
Formatted: Font: Not Bold, Italic, No
Formatted: Font: Not Bold, No underline
Formatted: Font: Not Bold, Italic, No
Formatted: Font: Not Bold, No underline
Formatted: Font: Not Bold, Italic, No
Formatted: Font: Not Bold, No underline
Formatted: Font: Not Bold, Italic, No
Formatted: Font: Not Bold, No underline
Formatted: Font: Not Bold, Italic, No
Formatted: Font: Not Bold, No underline
Formatted: Font: Not Bold, Italic, No
Formatted: Font: Not Bold, No underline
Formatted: Font: Not Bold, Italic, No
Formatted: Font: Not Bold, No underline
Formatted: Font: Not Bold, Italic, No
Formatted: Font: Not Bold, No underline
Formatted: Font: Not Bold, Italic, No
Formatted: Font: Not Bold, No underline
Formatted: Font: Not Bold, Italic, No
Formatted: Font: Not Bold, No underline
Formatted: Font: Not Bold, Italic, No
Formatted: Font: Not Bold, No underline
Formatted: Font: Not Bold, Italic, No
Formatted: Font: Not Bold, No underline
Formatted: Font: Not Bold, Italic, No
Formatted: Font: Not Bold, No underline
Formatted: Font: Not Bold, Italic, No
Formatted: Font: Not Bold, No underline
Formatted: Font: Not Bold, Italic, No
Formatted: Font: Not Bold, No underline
Formatted: Font: Not Bold, Italic, No
Formatted: Font: Not Bold, No underline
Formatted: Font: Not Bold, Italic, No
Formatted: Font: Not Bold, No underline

For peer review only

43. Nichols W.W., O'Rourke M.F., editors. *McDonald's Blood Flow in Arteries: Theoretical, Experimental and Clinical Principals*. London: Hodder Arnold, 2005.
44. O'Rourke MF, Pauca AL. Augmentation of the aortic and central arterial pressure waveform. *Blood Press Monit* 2004;9(4):179-85.
45. Reece AS. High-sensitivity CRP in opiate addiction: relative and age-dependent elevations. *Cardiovasc Toxicol* 2012;12(2):149-57.
46. Fauci AS, Braunwald E, Kapser DL, Hauser SL, Longo DL, Jameson JL, editors. *Harrison's Principles of Internal Medicine, 17th Edition*. 17th Edition ed. New York: McGraw Hill, 2008.
47. Lakatta EG. Arterial aging is risky. *J Appl Physiol* 2008;105(4):1321-2.
48. Le Couteur DG, Lakatta EG. A vascular theory of aging. *J Gerontol A Biol Sci Med Sci* 2010;65(10):1025-7.
49. Hadley EC, Lakatta EG, Morrison-Bogorad M, Warner HR, Hodes RJ. The future of aging therapies. *Cell* 2005;120(4):557-67.
50. Weber T, Auer J, O'Rourke MF, Kvas E, Lassnig E, Berent R, et al. Arterial stiffness, wave reflections, and the risk of coronary artery disease. *Circulation* 2004;109(2):184-9.
51. Williams B, Lacy PS, Thom SM, Cruickshank K, Stanton A, Collier D, et al. Differential impact of blood pressure-lowering drugs on central aortic pressure and clinical outcomes: principal results of the Conduit Artery Function Evaluation (CAFE) study. *Circulation* 2006;113(9):1213-25.
52. Nichols WW. Clinical measurement of arterial stiffness obtained from noninvasive pressure waveforms. *Am J Hypertens* 2005;18(1 Pt 2):3S-10S.
53. Degenhardt L, Larney S, Randall D, Burns L, Hall W. Causes of death in a cohort treated for opioid dependence between 1985 and 2005. *Addiction* 2013.
54. Reece A. S. Manifold Implications of Forgotten Hyperglycaemia in Clinical Opiate Dependence. *Drug and Chemical Toxicology* 2012.
55. Reece AS. Relative and Age-dependent Stimulation of Soluble and Cellular Immunity in Opiate Dependence. *J Addict Med* 2012;6(1):10-7.
56. Reece AS. Differing age related trajectories of dysfunction in several organ systems in opiate dependence. *Aging Clin Exp Res* 2012;24(1):85-96.
57. Reece AS. Dentition of addiction in Queensland: poor dental status and major contributing drugs. *Australian dental journal* 2007;52(2):144-9.
58. Reece A. Lifetime prevalence of cervical neoplasia in addicted and medical patients. *Aust N Z J Obstet Gynaecol* 2007;47(5):419-23.
59. Tatia M.C. Lee, Gordon L.F. Cheng, Hung Zeng, Meikei Leung, Hui-Jun Zhang, Benson W.M. Lau, et al. Heroin abuse accelerates biological aging: A novel insight from telomerase and brain imaging interaction. *Translational Psychiatry* 2013.
60. Cheng F, McLaughlin PJ, Verderame MF, Zagon IS. The OGF-OGFr axis utilizes the p16INK4a and p21WAF1/CIP1 pathways to restrict normal cell proliferation. *Molecular biology of the cell* 2009;20(1):319-27.
61. Zagon IS, Verderame MF, McLaughlin PJ. The biology of the opioid growth factor receptor (OGFr). *Brain research* 2002;38(3):351-76.
62. Pasmant E, Sabbagh A, Vidaud M, Bieche I. ANRIL, a long, noncoding RNA, is an unexpected major hotspot in GWAS. *FASEB J* 2010;25(2):444-48.
63. Hutchinson MR, Shavit Y, Grace PM, Rice KC, Maier SF, Watkins LR. Exploring the neuroimmunopharmacology of opioids: an integrative review of mechanisms of central immune signaling and their implications for opioid analgesia. *Pharmacological reviews* 2011;63(3):772-810.
64. Starr ME, Hu Y, Stromberg AJ, Carmical JR, Wood TG, Evers BM, et al. Gene expression profile of mouse white adipose tissue during inflammatory stress: age-dependent upregulation of major pro-coagulant factors. *Aging Cell* 2012:n/a-n/a.
65. Coller JK, Hutchinson MR. Implications of central immune signaling caused by drugs of abuse: mechanisms, mediators and new therapeutic approaches for prediction and treatment of drug dependence. *Pharmacol Ther* 2012;134(2):219-45.

Formatted: Font: Not Bold, Italic, No
Formatted: Font: Not Bold, No underline
Formatted: Font: Not Bold, Italic, No
Formatted: Font: Not Bold, No underline
Formatted: Font: Not Bold, Italic, No
Formatted: Font: Not Bold, No underline
Formatted: Font: Not Bold, Italic, No
Formatted: Font: Not Bold, No underline
Formatted: Font: Not Bold, Italic, No
Formatted: Font: Not Bold, No underline
Formatted: Font: Not Bold, Italic, No
Formatted: Font: Not Bold, No underline
Formatted: Font: Not Bold, Italic, No
Formatted: Font: Not Bold, No underline
Formatted: Font: Not Bold, Italic, No
Formatted: Font: Not Bold, No underline
Formatted: Font: Not Bold, Italic, No
Formatted: Font: Not Bold, No underline
Formatted: Font: Not Bold, Italic, No
Formatted: Font: Not Bold, No underline
Formatted: Font: Not Bold, Italic, No
Formatted: Font: Not Bold, No underline
Formatted: Font: Not Bold, Italic, No
Formatted: Font: Not Bold, No underline
Formatted: Font: Not Bold, Italic, No
Formatted: Font: Not Bold, No underline
Formatted: Font: Not Bold, Italic, No
Formatted: Font: Not Bold, No underline
Formatted: Font: Not Bold, Italic, No
Formatted: Font: Not Bold, No underline
Formatted: Font: Not Bold, Italic, No
Formatted: Font: Not Bold, No underline
Formatted: Font: Not Bold, Italic, No
Formatted: Font: Not Bold, No underline
Formatted: Font: Not Bold, Italic, No
Formatted: Font: Not Bold, No underline
Formatted: Font: Not Bold, Italic, No
Formatted: Font: Not Bold, No underline
Formatted: Font: Not Bold, Italic, No
Formatted: Font: Not Bold, No underline
Formatted: Font: Not Bold, Italic, No
Formatted: Font: Not Bold, No underline
Formatted: Font: Not Bold, Italic, No
Formatted: Font: Not Bold, No underline
Formatted: Font: Not Bold, Italic, No
Formatted: Font: Not Bold, No underline

For peer review only

66. Grzanna R., Brown R.M. NIDA Research Monograph 125: Activation of Intermediate Early Genes by Drugs of Abuse. In: National Institutes of Drug Abuse, editor. Bethesda, Maryland: NIH, 1993:1-220.

67. Kirkwood TB. Understanding the odd science of aging. *Cell* 2005;120(4):437-47.

68. Hoing S, Rudhard Y, Reinhardt P, Glatza M, Stehling M, Wu G, et al. Discovery of inhibitors of microglial neurotoxicity acting through multiple mechanisms using a stem-cell-based phenotypic assay. *Cell Stem Cell* 2012;11(5):620-32.

69. Zhang G, Li J, Purkayastha S, Tang Y, Zhang H, Yin Y, et al. Hypothalamic programming of systemic ageing involving IKK-[bgr], NF-[kgr]B and GnRH. *Nature* 2013;497(7448):211-16.

70. Li J, Tang Y, Cai D. IKKbeta/NF-kappaB disrupts adult hypothalamic neural stem cells to mediate a neurodegenerative mechanism of dietary obesity and pre-diabetes. *Nat Cell Biol* 2012;14(10):999-1012.

71. Purkayastha S, Zhang G, Cai D. Uncoupling the mechanisms of obesity and hypertension by targeting hypothalamic IKK-beta and NF-kappaB. *Nat Med* 2011;17(7):883-7.

72. Thaler JP, Yi CX, Schur EA, Guyenet SJ, Hwang BH, Dietrich MO, et al. Obesity is associated with hypothalamic injury in rodents and humans. *J Clin Invest* 2012;122(1):153-62.

73. Czapski GA, Gajkowska B, Strosznajder JB. Systemic administration of lipopolysaccharide induces molecular and morphological alterations in the hippocampus. *Brain Res* 2010;1356:85-94.

74. Wang X, Loram LC, Ramos K, de Jesus AJ, Thomas J, Cheng K, et al. Morphine activates neuroinflammation in a manner parallel to endotoxin. *Proc Natl Acad Sci U S A* 2012;109(16):6325-30.

75. Billes SK, Greenway FL. Combination therapy with naltrexone and bupropion for obesity. *Expert opinion on pharmacotherapy* 2011;12(11):1813-26.

76. Neal MD, Jia H, Eyer B, Good M, Guerriero CJ, Sodhi CP, et al. Discovery and validation of a new class of small molecule Toll-like receptor 4 (TLR4) inhibitors. *PLoS ONE* 2013;8(6):e65779.

77. Theberge FR, Li X, Kambhampati S, Pickens CL, St Laurent R, Bossert JM, et al. Effect of chronic delivery of the Toll-like receptor 4 antagonist (+)-naltrexone on incubation of heroin craving. *Biol Psychiatry* 2013;73(8):729-37.

78. Kim CG, Chung IY, Lim Y, Lee YH, Shin SY. A Tcf/Lef element within the enhancer region of the human NANOG gene plays a role in promoter activation. *Biochem Biophys Res Commun* 2011;410(3):637-42.

79. Yang HM, Do HJ, Oh JH, Kim JH, Choi SY, Cha KY, et al. Characterization of putative cis-regulatory elements that control the transcriptional activity of the human Oct4 promoter. *J Cell Biochem* 2005;96(4):821-30.

80. Folmes CD, Dzeja PP, Nelson TJ, Terzic A. Metabolic plasticity in stem cell homeostasis and differentiation. *Cell Stem Cell* 2012;11(5):596-606.

81. Zurolo E, Iyer A, Maroso M, Carbonell C, Anink JJ, Ravizza T, et al. Activation of Toll-like receptor, RAGE and HMGB1 signalling in malformations of cortical development. *Brain* 2011;134(Pt 4):1015-32.

82. Nogueira-Machado JA, Volpe CM, Veloso CA, Chaves MM. HMGB1, TLR and RAGE: a functional tripod that leads to diabetic inflammation. *Expert opinion on therapeutic targets* 2011;15(8):1023-35.

83. Sakaguchi M, Murata H, Yamamoto K, Ono T, Sakaguchi Y, Motoyama A, et al. TIRAP, an adaptor protein for TLR2/4, transduces a signal from RAGE phosphorylated upon ligand binding. *PLoS ONE* 2011;6(8):e23132.

84. Veloso CA, Fernandes JS, Volpe CM, Fagundes-Netto FS, Reis JS, Chaves MM, et al. TLR4 and RAGE: similar routes leading to inflammation in type 2 diabetic patients. *Diabetes Metab* 2011;37(4):336-42.

~~**References**~~

Formatted: Font: Not Bold, Italic, No
Formatted: Font: Not Bold, No underline
Formatted: Font: Not Bold, Italic, No
Formatted: Font: Not Bold, No underline
Formatted: Font: Not Bold, Italic, No
Formatted: Font: Not Bold, No underline
Formatted: Font: Not Bold, Italic, No
Formatted: Font: Not Bold, No underline
Formatted: Font: Not Bold, Italic, No
Formatted: Font: Not Bold, No underline
Formatted: Font: Not Bold, Italic, No
Formatted: Font: Not Bold, No underline
Formatted: Font: Not Bold, Italic, No
Formatted: Font: Not Bold, No underline
Formatted: Font: Not Bold, Italic, No
Formatted: Font: Not Bold, No underline
Formatted: Font: Not Bold, Italic, No
Formatted: Font: Not Bold, No underline
Formatted: Font: Not Bold, Italic, No
Formatted: Font: Not Bold, No underline
Formatted: Font: Not Bold, Italic, No
Formatted: Font: Not Bold, No underline
Formatted: Font: Not Bold, Italic, No
Formatted: Font: Not Bold, No underline
Formatted: Font: Not Bold, Italic, No
Formatted: Font: Not Bold, No underline
Formatted: Font: Not Bold, Italic, No
Formatted: Font: Not Bold, No underline
Formatted: Font: Not Bold, Italic, No
Formatted: Font: Not Bold, No underline
Formatted: Font: Not Bold, Italic, No
Formatted: Font: Not Bold, No underline
Formatted: Font: Not Bold, Italic, No
Formatted: Font: Not Bold, No underline
Formatted: Font: Not Bold, Italic, No
Formatted: Font: Not Bold, No underline
Formatted: Left, Indent: Left: 0", Hanging: 0.5"

For peer review only

1. Rosen D, Smith ML, Reynolds CF, 3rd. The prevalence of mental and physical health disorders among older methadone patients. *Am J Geriatr Psychiatry* 2008;16(6):488-97.
2. Cooper OB, Brown TT, Dobs AS. Opiate drug use: a potential contributor to the endocrine and metabolic complications in human immunodeficiency virus disease. *Clin Infect Dis* 2003;37 Suppl 2:S132-6.
3. Ceriello A, Quatraro A, Giugliano D. Opiate addict as diabetic patient? *Diabetes Care* 1988;11(5):443.
4. Kolarzyk E, Pach D, Wojtowicz B, Szpanowska-Wohn A, Szurkowska M. Nutritional status of the opiate dependent persons after 4 years of methadone maintenance treatment. *Przeglad lekarski* 2005;62(6):373-7.
5. Brunton LL, Lazo JS, Parker KL, editors. *Goodman and Gilman's the Pharmacologic Basis of Therapeutics*. Eleventh Edition ed. New York: McGraw Hill, 2006.
6. Reece A. S. Relative and Age Dependent Stimulation of Soluble and Cellular Immunity in Opiate Dependence. *Journal of Addiction Medicine* 2012;6(1):10-17.
7. Reece A. S. High Sensitivity CRP in Opiate Addiction: Relative and Age-Dependent Elevations. *Cardiovascular Toxicology* 2012;12(2):149-57.
8. Neri S, Bruno CM, Pulvirenti D, Malaguarnera M, Italiano C, Mauceri B, et al. Randomized clinical trial to compare the effects of methadone and buprenorphine on the immune system in drug abusers. *Psychopharmacology (Berl)* 2005;179(3):700-4.
9. Reece A. S. Absolute and Age Dependent Elevations of Serum Calcium and Phosphate and their Products in Clinical Opiate Dependence. *J. Subs. Abuse* 2013;Accepted 27th November 2012.
10. Reece AS, Davidson P. Deficit of circulating stem--progenitor cells in opiate addiction: a pilot study. *Substance abuse treatment, prevention, and policy* 2007;2:19-28.
11. Darke S, Duflou J, Torok M. The comparative toxicology and major organ pathology of fatal methadone and heroin toxicity cases. *Drug Alcohol Depend* 2010;106(1):1-6.
12. Darke S, Kaye S, Duflou J. Systemic disease among cases of fatal opioid toxicity. *Addiction* 2006;101(9):1299-305.
13. Degenhardt L., Randall D., Hall W., Law M., Butler T., Burns L. Mortality among clients of a state-wide opioid pharmacotherapy program over 20 years: Risk factors and lives saved. *Drug Alcohol Depend* 2009;105(1-2):9-15.
14. Khademi H, Malekzadeh R, Pourshams A, Jafari E, Salahi R, Semnani S, et al. Opium use and mortality in Golestan Cohort Study: prospective cohort study of 50,000 adults in Iran. *BMJ (Clinical research ed* 2012;344:e2502.
15. Sadeghian S, Darvish S, Davoodi G, Salarifar M, Mahmoodian M, Fallah N, et al. The association of opium with coronary artery disease. *Eur J Cardiovasc Prev Rehabil* 2007;14(5):715-7.
16. Sadeghian S, Dowlatshahi S, Karimi A, Tazik M. Epidemiology of opium use in 4398 patients admitted for coronary artery bypass graft in Tehran Heart Center. *J Cardiovasc Surg (Torino)* 2011;52(1):140-1.
17. Sadeghian S, Graili P, Salarifar M, Karimi AA, Darvish S, Abbasi SH. Opium consumption in men and diabetes mellitus in women are the most important risk factors of premature coronary artery disease in Iran. *International journal of cardiology* 2010;141(1):116-8.
18. Smyth B, Hoffman V, Fan J, Hser YI. Years of potential life lost among heroin addicts 33 years after treatment. *Preventive medicine* 2007;44(4):369-74.
19. Reece A.S., Hulse G.K. Impact of Opioid Pharmacotherapy on Arterial stiffness and Vascular Ageing: Cross-sectional and Longitudinal Studies. *Cardiovascular Toxicology* 2013;13(3):254-66.
20. Reece A. S., Hulse G.K. Reduction in Arterial Stiffness and Vascular Age by Naltrexone-Induced Interruption of Opiate Agonism. *British Medical Journal - Open* 2013.
21. Hosseini SK, Masoudkabir F, Vasheghani-Farahani A, Alipour-Parsa S, Sheikh Fathollahi M, Rahimi-Foroushani A, et al. Opium consumption and coronary atherosclerosis in diabetic patients: a propensity score-matched study. *Planta Med* 2011;77(17):1870-5.
22. Charney DS, Redmond DE, Jr., Galloway MP, Kleber HD, Heninger GR, Murberg M, et al. Naltrexone precipitated opiate withdrawal in methadone addicted human subjects: evidence for noradrenergic hyperactivity. *Life Sci* 1984;35(12):1263-72.

For peer review only

23. Reece AS, Hulse GK. Elevation of Central Arterial Stiffness and Vascular Ageing in Opiate Withdrawal: Cross-sectional and Longitudinal Studies. *Cardiovasc Toxicol* 2012.
24. Chien KR, Karsenty G. Longevity and lineages: toward the integrative biology of degenerative diseases in heart, muscle, and bone. *Cell* 2005;120(4):533-44.
25. Lakatta EG, Wang M, Najjar SS. Arterial aging and subclinical arterial disease are fundamentally intertwined at macroscopic and molecular levels. *Med Clin North Am* 2009;93(3):583-604, Table of Contents.
26. Reece AS. Hair graying in substance addiction. *Archives of dermatology* 2007;143(1):116-8.
27. Kim TW, Alford DP, Malabanan A, Holick MF, Samet JH. Low bone density in patients receiving methadone maintenance treatment. *Drug Alcohol Depend* 2006;85(3):258-62.
28. Reece AS. Evidence of Accelerated Ageing in Clinical Drug Addiction from Immune, Hepatic and Metabolic Biomarkers. *Immun Ageing* 2007;4(1):6-15.
29. Randall D, Degenhardt L, Vajdic CM, Burns L, Hall WD, Law M, et al. Increasing cancer mortality among opioid-dependent persons in Australia: a new public health challenge for a disadvantaged population. *Australian and New Zealand journal of public health* 2011;35(3):220-5.
30. Reece A.S., Hulse G.K. Lifetime Opiate Exposure as an Independent and Interactive Cardiovascular Risk Factor in Males: A Cross-Sectional Clinical Study. *Vascular Health and Risk Management* 2013;9(55):551-59.
31. Reece A.S., Hulse G.K. Opiate Exposure Increases Arterial Stiffness, Advances Vascular Age and is an Independent Cardiovascular Risk Factor in Females: A Cross-Sectional Clinical Study. *World J. Cardiovascular Diseases* 2013;In Press.
32. Reece A. S., Hulse G.K. Duration of Opiate Exposure as a Determinant of Arterial Stiffness and Vascular Age in Male Opiate Dependence: A Longitudinal Study. *Journal of Clinical Pharmacy and Therapeutics* 2013.
33. Reece A. S., Hulse G.K. Opiate Dependence as an Independent and Interactive Risk Factor for Arterial Stiffness and Cardiovascular Ageing - A Longitudinal Study in Females. *J Clinical Medical Research* 2013;5(5):356-67.
34. Reece AS. Psychosocial and treatment correlates of opiate free success in a clinical review of a naltrexone implant program. *Subst Abuse Treat Prev Policy* 2007;2:35-49.
35. Breslow N.E., Day N.E. *Statistical Methods in Cancer Research Volume II–The Design and Analysis of Cohort Studies*. 1 ed. Lyon: International Agency for Cancer Research,, 1987.
36. Kacinko SL, Jones HE, Johnson RE, Choo RE, Huestis MA. Correlations of maternal buprenorphine dose, buprenorphine, and metabolite concentrations in meconium with neonatal outcomes. *Clinical pharmacology and therapeutics* 2008;84(5):604-12.
37. Hulse G., White J., Cape G., editors. *Management of Drug and Alcohol Problems*. Sydney: Oxford University Press, 2002.
38. Oviedo-Joekes E, Brissette S, Marsh DC, Lauzon P, Guh D, Anis A, et al. Diacetylmorphine versus methadone for the treatment of opioid addiction. *N Engl J Med* 2009;361(8):777-86.
39. SphygmoCor, editor. *Software Operator's Guide, Pulse Wave Analysis System, SCOR-Px*. Sydney: AtCor Medical, 2006.
40. Klein SL. Sex influences immune responses to viruses, and efficacy of prophylaxis and treatments for viral diseases. *Bioessays* 2012;34(12):1050-9.
41. Nichols W.W., O'Rourke M.F., editors. *McDonald's Blood Flow in Arteries: Theoretical, Experimental and Clinical Principals*. London: Hodder Arnold, 2005.
42. O'Rourke MF, Pauca AL. Augmentation of the aortic and central arterial pressure waveform. *Blood Press Monit* 2004;9(4):179-85.
43. Weber T, Auer J, O'Rourke MF, Kvas E, Lassnig E, Berent R, et al. Arterial stiffness, wave reflections, and the risk of coronary artery disease. *Circulation* 2004;109(2):184-9.
44. Williams B, Lacy PS, Thom SM, Cruickshank K, Stanton A, Collier D, et al. Differential impact of blood pressure-lowering drugs on central aortic pressure and clinical outcomes: principal results of the Conduit Artery Function Evaluation (CAFE) study. *Circulation* 2006;113(9):1213-25.
45. Nichols WW. Clinical measurement of arterial stiffness obtained from noninvasive pressure waveforms. *Am J Hypertens* 2005;18(1 Pt 2):3S-10S.

For peer review only

46. Cheng F, McLaughlin PJ, Verderame MF, Zagon IS. The OGF-OGFr axis utilizes the p16INK4a and p21WAF1/CIP1 pathways to restrict normal cell proliferation. *Molecular biology of the cell* 2009;20(1):319-27.
47. Zagon IS, Verderame MF, McLaughlin PJ. The biology of the opioid growth factor receptor (OGFr). *Brain research* 2002;38(3):351-76.
48. Pasmant E, Sabbagh A, Vidaud M, Bieche I. ANRIL, a long, noncoding RNA, is an unexpected major hotspot in GWAS. *FASEB J* 2010;25(2):444-48.
49. Hutchinson MR, Shavit Y, Grace PM, Rice KC, Maier SF, Watkins LR. Exploring the neuroimmunopharmacology of opioids: an integrative review of mechanisms of central immune signaling and their implications for opioid analgesia. *Pharmacological reviews* 2011;63(3):772-810.
50. Starr ME, Hu Y, Stromberg AJ, Carmical JR, Wood TG, Evers BM, et al. Gene expression profile of mouse white adipose tissue during inflammatory stress: age-dependent upregulation of major pro-coagulant factors. *Aging Cell* 2012:n/a-n/a.
51. Coller JK, Hutchinson MR. Implications of central immune signaling caused by drugs of abuse: mechanisms, mediators and new therapeutic approaches for prediction and treatment of drug dependence. *Pharmacol Ther* 2012;134(2):219-45.
52. Grzanna R., Brown R.M. NIDA Research Monograph 125: Activation of Intermediate Early Genes by Drugs of Abuse. In: National Institutes of Drug Abuse, editor. Bethesda, Maryland: NIH, 1993:1-220.
53. Hadley EC, Lakatta EG, Morrison-Bogorad M, Warner HR, Hodes RJ. The future of aging therapies. *Cell* 2005;120(4):557-67.
54. Kirkwood TB. Understanding the odd science of aging. *Cell* 2005;120(4):437-47.
55. Hoing S, Rudhard Y, Reinhardt P, Glatza M, Stehling M, Wu G, et al. Discovery of inhibitors of microglial neurotoxicity acting through multiple mechanisms using a stem-cell-based phenotypic assay. *Cell Stem Cell* 2012;11(5):620-32.
56. Zhang G, Li J, Purkayastha S, Tang Y, Zhang H, Yin Y, et al. Hypothalamic programming of systemic ageing involving IKK-[bgr], NF-[kgr]B and GnRH. *Nature* 2013;497(7448):211-16.
57. Li J, Tang Y, Cai D. IKKbeta/NF-kappaB disrupts adult hypothalamic neural stem cells to mediate a neurodegenerative mechanism of dietary obesity and pre-diabetes. *Nat Cell Biol* 2012;14(10):999-1012.
58. Purkayastha S, Zhang G, Cai D. Uncoupling the mechanisms of obesity and hypertension by targeting hypothalamic IKK-beta and NF-kappaB. *Nat Med* 2011;17(7):883-7.
59. Thaler JP, Yi CX, Schur EA, Guyenet SJ, Hwang BH, Dietrich MO, et al. Obesity is associated with hypothalamic injury in rodents and humans. *J Clin Invest* 2012;122(1):153-62.
60. Czapski GA, Gajkowska B, Strosznajder JB. Systemic administration of lipopolysaccharide induces molecular and morphological alterations in the hippocampus. *Brain Res* 2010;1356:85-94.
61. Wang X, Loram LC, Ramos K, de Jesus AJ, Thomas J, Cheng K, et al. Morphine activates neuroinflammation in a manner parallel to endotoxin. *Proc Natl Acad Sci U S A* 2012;109(16):6325-30.
62. Cowley MA, Smart JL, Rubinstein M, Cerdan MG, Diano S, Horvath TL, et al. Leptin activates anorexigenic POMC neurons through a neural network in the arcuate nucleus. *Nature* 2001;411(6836):480-4.
63. Billes SK, Greenway FL. Combination therapy with naltrexone and bupropion for obesity. *Expert opinion on pharmacotherapy* 2011;12(11):1813-26.
64. Neal MD, Jia H, Eyer B, Good M, Guerriero CJ, Sodhi CP, et al. Discovery and validation of a new class of small molecule Toll-like receptor 4 (TLR4) inhibitors. *PLoS ONE* 2013;8(6):e65779.
65. Theberge FR, Li X, Kambhampati S, Pickens CL, St Laurent R, Bossert JM, et al. Effect of chronic delivery of the Toll-like receptor 4 antagonist (+)-naltrexone on incubation of heroin craving. *Biol Psychiatry* 2013;73(8):729-37.
66. Kim CG, Chung IY, Lim Y, Lee YH, Shin SY. A Tcf/Lef element within the enhancer region of the human NANOG gene plays a role in promoter activation. *Biochem Biophys Res Commun* 2011;410(3):637-42.

For peer review only

67. Yang HM, Do HJ, Oh JH, Kim JH, Choi SY, Cha KY, et al. Characterization of putative cis-regulatory elements that control the transcriptional activity of the human Oct4 promoter. *J Cell Biochem* 2005;96(4):821-30.
68. Folmes CD, Dzeja PP, Nelson TJ, Terzic A. Metabolic plasticity in stem cell homeostasis and differentiation. *Cell Stem Cell* 2012;11(5):596-606.
69. Zurolo E, Iyer A, Maroso M, Carbonell C, Anink JJ, Ravizza T, et al. Activation of Toll-like receptor, RAGE and HMGB1 signalling in malformations of cortical development. *Brain* 2011;134(Pt 4):1015-32.
70. Nogueira-Machado JA, Volpe CM, Veloso CA, Chaves MM. HMGB1, TLR and RAGE: a functional tripod that leads to diabetic inflammation. *Expert opinion on therapeutic targets* 2011;15(8):1023-35.
71. Sakaguchi M, Murata H, Yamamoto K, Ono T, Sakaguchi Y, Motoyama A, et al. TIRAP, an adaptor protein for TLR2/4, transduces a signal from RAGE phosphorylated upon ligand binding. *PLoS ONE* 2011;6(8):e23132.
72. Veloso CA, Fernandes JS, Volpe CM, Fagundes-Netto FS, Reis JS, Chaves MM, et al. TLR4 and RAGE: similar routes leading to inflammation in type 2 diabetic patients. *Diabetes Metab* 2011;37(4):336-42.

For peer review only

Figure Captions:

Figure 1: Ageing and Stiffness Indices by Chronologic Age by ~~Opiate~~Opioid Dependency Status

Figure 2: Corrected Ageing and Stiffness Indices by Time by ~~Opiate~~Opioid Dependency Status

Figure 3: Ageing Indices by Chronologic Age by Addictive Status by Sex

Figure 4: Ageing Indices by Chronologic Age by Sex by Addictive Status

Figure 5: BMI-Related Ageing Indices by Chronologic Age by Addictive Status by Sex

Figure 6: BMI-Related Ageing Indices by Chronologic Age by Sex by Addictive Status

Figure 7: Ageing Indices by Chronologic Age by Sex by Addictive Status – Loess Curves

Figure 8: Vascular Age Indices by Chronologic Age by ~~Opiate~~Opioid Dependency Level, Loess curves

Supplementary Figure Captions:

Suppl. Fig. 1: Vascular Age Indices by Time by ~~Opiate~~Opioid Dependency Level

For peer review only

TABLES

For peer review only

Table 1.: Effects of Gender on Key Measures of Arterial Stiffness - Cross-sectional Dataset

Dependent Variable	Group	Parameter	Adj. R^2	F	dF	Model P	Estimate	Std. Error	t-Value	P
RA-CA Difference	~~Opiate~~Opioid	Chron.Age :Female.Sex	0.0189	7.596	2, 684	0.0005459	0.6324	0.3469	1.823	0.0688
BMI	Control	Chron.Age :Female.Sex	0.1523	52.58	2, 572	<2.2E-16	-0.0092	0.0040	-2.310	0.0213
BMI	~~Opiate~~Opioid	Female.Sex	0.0713	27.35	2, 684	3.75E-12	-0.0827	0.0139	-5.940	4.6E-09
RA/BMI	~~Opiate~~Opioid	Chron.Age :Female.Sex	0.2617	122.6	2, 684	<2.2E-16	0.0361	0.0091	3.948	8.7E-05
C_AP_HR75/BMI	Control	Chron.Age :Female.Sex	0.4882	274.3	2, 571	<2.2E-16	0.0312	0.0040	7.737	4.6E-14
C_AP_HR75/BMI	Control	Chron.Age :Female.Sex	0.4882	274.3	2, 571	<2.2E-16	0.0312	0.0040	7.737	4.6E-14
C_AP_HR75/BMI	~~Opiate~~Opioid	Chron.Age :Female.Sex	0.3712	203.4	2, 684	<2.2E-16	0.0467	0.0039	11.950	<2e-16
C_AGPH_HR75/BMI	Control	Chron.Age :Female.Sex	0.4603	245.4	2, 571	<2.2E-16	0.0924	0.0112	8.283	8.6E-16
C_AGPH_HR75/BMI	~~Opiate~~Opioid	Chron.Age :Female.Sex	0.3980	227.8	2, 684	<2.2E-16	0.1265	0.0095	13.370	<2e-16
C_AP_HR75/(CA*BMI)	Control	Female.Sex	0.2809	112.9	2, 571	<2.2E-16	0.0033	0.0005	6.286	6.5E-10
C_AP_HR75/(CA*BMI)	~~Opiate~~Opioid	Female.Sex	0.2489	114.7	2, 684	<2.2E-16	0.0047	0.0004	11.428	<2e-16
C_AGPH_HR75/(CA*BMI)	Control	Female.Sex	0.2567	99.96	2, 571	<2.2E-16	0.0096	0.0015	6.350	4.4E-10
C_AGPH_HR75/(CA*BMI)	~~Opiate~~Opioid	Female.Sex	0.2604	121.8	2, 684	<2.2E-16	0.0130	0.0011	12.307	<2e-16

For peer review only

Table 2.:

Age: Addictive Status Relationships with CA Discretized

About 41 Years in Males and 29 Years in Females

With Females <55 Years of Age

Parameter	Variable Statistics				Model Summaries		
	Estimate	Std. Error	t Value	~~Pr(>|t|)~~P	F	dF	P
Males							
Older Age	-0.1837	0.0557	-3.296	0.0010	5.492	3, 862	0.00097
~~Opiate~~Opioids	-0.0846	0.0275	-3.082	0.0021			
Older Age: ~~Opiate~~Opioids	0.2310	0.0687	3.364	0.0008			
Females							
Older Age: ~~Opiate~~Opioids	0.1248	0.0532	2.346	0.0195	2.169	3, 378	0.09123

For peer review only

Table 3.: Cross-sectional Models Polynomial in Heroin Duration

	Parameter				Model		
Model / Parameter	Value	Std.Error	t-value	P-value	F	dF	P
1 Order- Linear							
H.Dura.n	-0.0059	0.0021	-2.779	0.0056	8.628	2, 664	0.0002
H.Dose: H.Dura.n	0.0091	0.0022	4.152	3.7.E-05			
2 Order- Quadratic							
H.Dura.n	-0.0171	0.0055	-3.136	0.0018	7.443	3, 663	6.60E-05
H.Dura.n^2	0.0004	0.0002	2.229	0.0261			
H.Dose: H.Dura.n	0.0084	0.0022	3.802	0.0002			
3 Order - Cubic							
H.Dura.n	-0.0116	0.0035	-3.263	0.0012	7.102	3 663	0.00011
H.Dura.n^3	6.3E-06	3.1E-06	1.993	0.0466			
H.Dose: H.Dura.n	0.0084	0.0022	3.836	0.0001			
4 Order- Quartic							
H.Dura.n	-0.0059	0.0021	-2.779	0.0056	8.628	2, 664	0.0002
H.Dose: H.Dura.n	0.0091	0.0022	4.152	3.7.E-05			
1, 2, 3 & 4 Order							
H.Dose: H.Dura.n^3	6.5E-05	2.0E-05	3.252	0.0012	3.353	7, 659	0.0016
H.Dura.n^2 : H.Dura.n^3	-1.0E-06	3.1E-07	-3.398	0.0007			
H.Dura.n^2 : H.Dura.n^4	9.2E-08	2.9E-08	3.216	0.0014			
H.Dura.n^3: H.Dura.n^4	-2.7E-09	8.8E-10	-3.029	0.0026			
H.Dose: H.Dura.n^2 : H.Dura.n^4	-4.5E-09	1.8E-09	-2.452	0.0145			
H.Dura.n: H.Dura.n^3: H.Dura.n^4	2.5E-11	9.0E-12	2.831	0.0048			
H.Dose: H.Dura.n: H.Dura.n^3: H.Dura.n^4	2.6E-12	1.2E-12	2.159	0.0312			

For peer review only

Table 4: Dose-Response Relationships

Cross-Sectional Studies							
Parameter	Variable Statistics				Model Summaries		
	Estimate	Std. Error	t Value	Pr(>|t|)	F	dF	P
Both Sexes							
H.Dura.n	-0.0059	0.0021	-2.779	0.0056	8.628	2, 664	0.0002
H.Dose: H.Dura.n	0.0091	0.0022	4.152	0.0000			
Males							
H.Dura.n	-0.0089	0.0028	-3.147	0.0018	7.105	2, 441	0.0009
H.Dose: H.Dura.n	0.0124	0.0034	3.658	0.0003			
Females							
H.Dose: H.Dura.n	0.0052	0.0023	2.281	0.0236	5.201	1, 210	0.0236

Longitudinal Studies							
Group	Variable Statistics					Model	
Parameter	Value	Std.Error	DF	t-value	P-value	AIC*	LogLik*
Both Sexes							
H.Dose:H.Dura.n	0.0045	0.0013	814	3.5512	0.0004	1068.74	-530.37
Males							
H.Dura.n	-0.0036	0.0016	552	-2.2069	0.0277	753.15	-371.58
H.Dose:H.Dura.n	0.0075	0.0022	552	3.3754	0.0008		
Females							
H.Dose:H.Dura.n	0.0052	0.0021	260	2.5091	0.0127	347.43	-169.72

For peer review only

Table 5.: Cross-Sectional Adjusted ~~Multivariate~~Multiple Regression Models

Group	Variable Statistics				Model		
**Parameter**	**Estimate**	**Std. Error**	**t Value**	**Pr(>|t|)**	**F**	**dF**	**P**
Both Sexes							
H.Dura.n: Cigs.d: SP : Weight	0.00023	0.00006	3.919	0.0001	7.561	9, 657	1.38E-10
H.Dura.n: Height: Cigs.d: SP	0.00030	0.00011	2.840	0.0047			
H.Dura.n: Height: Cigs.d	-0.00143	0.00051	-2.828	0.0048			
H.Dura.n: Height: Cigs.d: SP : Weight	-0.00007	0.00002	-2.808	0.0051			
H.Dura.n: Height: Cigs.d: Weight	0.00032	0.00012	2.741	0.0063			
H.Dose: H.Dura.n: Height: Cigs.d: Weight	-0.00037	0.00016	-2.229	0.0261			
H.Dose: H.Dura.n: Height: Cigs.d: SP : Weight	0.00008	0.00003	2.229	0.0262			
H.Dose: H.Dura.n: Height: Cigs.d	0.00155	0.00071	2.194	0.0286			
H.Dose: H.Dura.n: Height: Cigs.d: SP	-0.00032	0.00015	-2.188	0.0290			
Males							
H.Dura.n: Weight	-0.0024	0.0007	-3.434	0.0007	5.485	10, 438	1.07E-07
H.Dose: H.Dura.n: Weight	0.0032	0.0010	3.158	0.0017			
H.Dose: Cigs.d: SP : Weight	-4.4770	1.7470	-2.563	0.0107			
H.Dose: Height: Cigs.d: SP : Weight	0.0253	0.0099	2.557	0.0109			
H.Dose: Cigs.d: SP	19.460	7.6160	2.556	0.0109			
H.Dose: Cigs.d: Weight	21.370	8.3900	2.548	0.0112			
H.Dose: Height: Cigs.d: Weight	-0.1209	0.0476	-2.542	0.0114			
H.Dose: Height: Cigs.d: SP	-0.1098	0.0432	-2.540	0.0114			
H.Dose: Cigs.d	-92.680	36.520	-2.538	0.0115			
H.Dose: Height: Cigs.d	0.5230	0.2073	2.523	0.0120			
Females							
Height: Cigs.d: Weight	-0.00041	0.00009	-4.327	0.00002	8.25	3, 214	3.20E-05
Height: Cigs.d: SP : Weight	0.00008	0.00002	4.272	0.00003			
H.Dose: H.Dura.n: Height: Cigs.d	2.2E-06	1.1E-06	1.957	0.05160			

For peer review only

Table 6.: Longitudinal Adjusted ~~Multivariate~~Multiple Models

Group						Model	
	Variable Statistics						
**Model / Parameter**	**Value**	**Std.Error**	**DF**	**t-value**	**P-value**	**AIC***	**LogLik***
Both Sexes							
Height	-0.0304	0.0026	606	-11.7198	0.0E+00	1238.239	- 592.1197
SP	0.7947	0.0949	606	8.3772	0.0E+00		
Days:H.Dura.n	0.0003	6.9E-05	606	4.3556	0.0E+00		
CA:Height	0.0066	0.0006	606	10.9049	0.0E+00		
Days: CA:H.Dura.n	-0.0001	0.0000	606	-4.2946	0.0E+00		
H.Dura.n: Cigs.d:Height	-5.3E-05	1.3E-05	606	-4.1566	0.0E+00		
Days:H.Dose: Cigs.d:Height	2.1E-06	5.0E-07	606	4.3108	0.0E+00		
Days: CA:H.Dose: Cigs.d:Height	-6.0E-07	2.0E-07	606	-4.2968	0.0E+00		
CA:H.Dura.n: Cigs.d:Height	1.4E-05	3.4E-06	606	4.0217	0.0001		
Days: CA: Cigs.d:Height	0.0E+00	0.0E+00	606	3.7701	0.0002		
Days:H.Dura.n: Cigs.d	-1.1E-05	3.1E-06	606	-3.4709	0.0006		
Cigs.d:Height	0.0007	0.0002	606	3.3714	0.0008		
Days: CA:H.Dose:H.Dura.n: Cigs.d:Height	0.0E+00	0.0E+00	606	3.2705	0.0011		
Days: CA:H.Dura.n: Cigs.d	2.5E-06	8.0E-07	606	3.2365	0.0013		
CA: Cigs.d:Height	-0.0002	0.0001	606	-3.2045	0.0014		
Days	-0.0022	0.0007	606	-2.9194	0.0036		
H.Dose:H.Dura.n: Cigs.d	0.0102	0.0036	606	2.8304	0.0048		
CA:H.Dose:H.Dura.n: Cigs.d	-0.0026	0.0010	606	-2.6804	0.0076		
Days: CA	0.0005	0.0002	606	2.2438	0.0252		
Days: CA:H.Dose:Height	6.8E-06	3.1E-06	606	2.1984	0.0283		
Days:H.Dose:Height	-2.2E-05	1.0E-05	606	-2.1747	0.0300		
Days:H.Dose:H.Dura.n	-3.5E-05	1.7E-05	606	-2.1086	0.0354		
H.Dose: Cigs.d	-0.1067	0.0511	606	-2.0888	0.0371		
CA:H.Dose: Cigs.d	0.0290	0.0148	606	1.9645	0.0499		
Males							
Height	-0.0302	0.0035	436	-8.7423	0.0E+00	913.00	-435.50
SP	0.6376	0.1105	436	5.7717	0.0E+00		
CA:Height	0.0064	0.0008	436	8.4711	0.0E+00		
Days:H.Dura.n	0.0003	0.0001	436	3.8907	0.0001		
Days: CA:H.Dura.n	-0.0001	0.0000	436	-3.8395	0.0001		
Days:H.Dose: Cigs.d:Height	1.6E-06	4.0E-07	436	3.8181	0.0002		
Days: CA:H.Dose: Cigs.d:Height	-5.0E-07	1.0E-07	436	-3.7217	0.0002		
Days	-0.0029	0.0008	436	-3.6624	0.0003		
Days: CA	0.0007	0.0002	436	3.3251	0.0010		
H.Dose:H.Dura.n: Cigs.d:Height	2.7E-06	8.0E-07	436	3.2331	0.0013		
Days: CA: Cigs.d	5.0E-06	1.6E-06	436	3.1839	0.0016		
H.Dura.n: Cigs.d:Height	-2.8E-05	9.1E-06	436	-3.1058	0.0020		
CA:H.Dura.n: Cigs.d:Height	7.1E-06	2.4E-06	436	2.9496	0.0034		
Days:H.Dura.n: Cigs.d	-8.3E-06	3.1E-06	436	-2.6824	0.0076		
Days: CA:H.Dura.n: Cigs.d	2.0E-06	8.0E-07	436	2.5562	0.0109		
Days: CA:H.Dose:H.Dura.n: Cigs.d:Height	0.0E+00	0.0E+00	436	2.2838	0.0229		
Cigs.d:Height	0.0004	0.0002	436	2.2799	0.0231		
CA: Cigs.d:Height	-0.0001	5.0E-05	436	-2.1938	0.0288		

For peer review only

Females							
Height	-0.0279	0.0036	169	-7.6753	0.0E+00	394.69	-187.35
SP	1.2880	0.1930	169	6.6744	0.0E+00		
CA:Height	0.0064	0.0005	169	11.7493	0.0E+00		
Days: CA:H.Dose:H.Dura.n:Height	1.3E-06	5.0E-07	169	2.7371	0.0069		
Days:H.Dose:H.Dura.n:Height	-4.5E-06	1.7E-06	169	-2.7147	0.0073		
Days: CA:H.Dose:H.Dura.n: Cigs.d:Height	-1.0E-07	0.0E+00	169	-2.6052	0.0100		
Days:H.Dose:H.Dura.n: Cigs.d:Height	2.0E-07	1.0E-07	169	2.5589	0.0114		

For peer review only

Figure 1

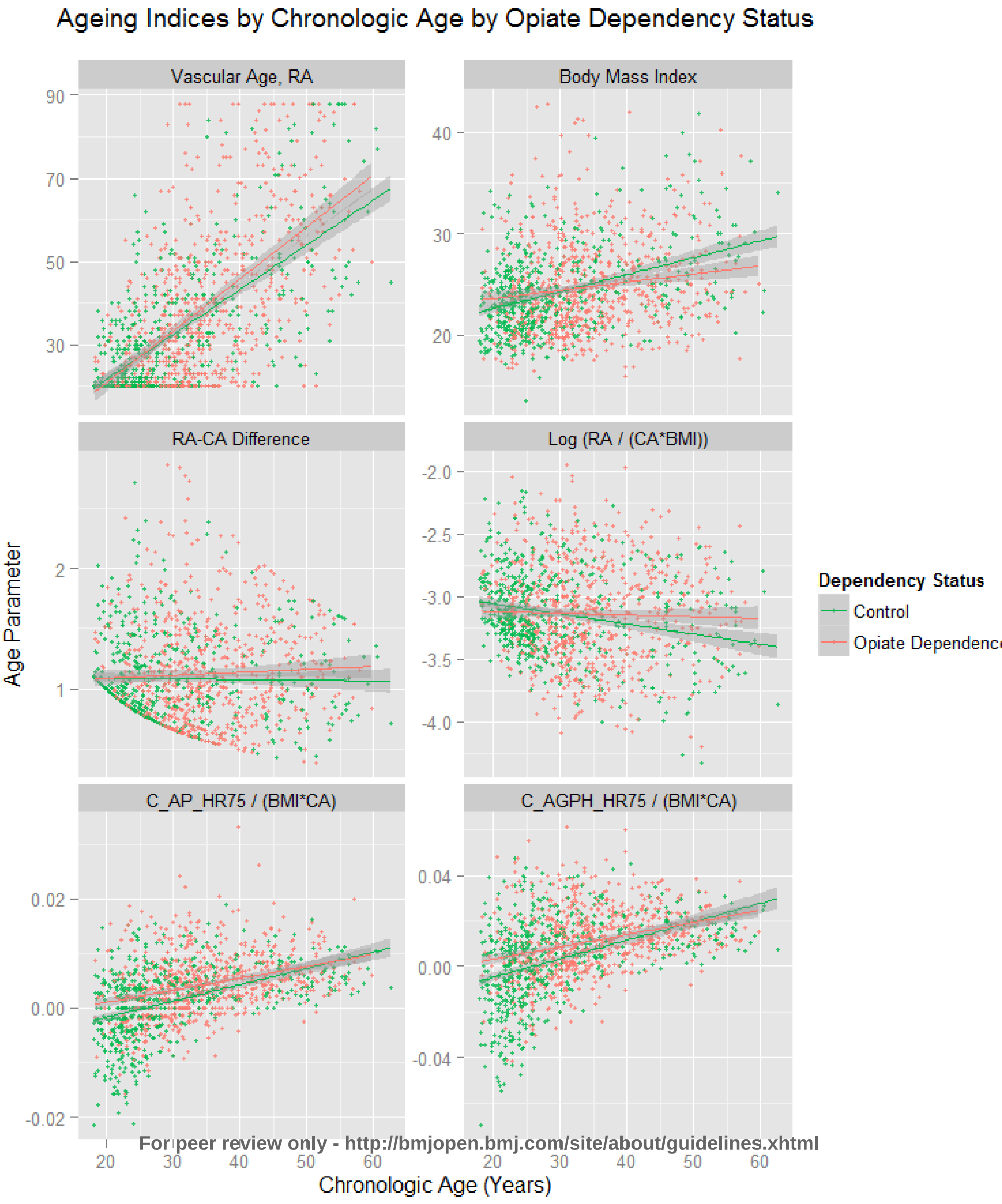
Ageing Indices by Chronologic Age by Opiate Dependency Status
Vascular Age, RA
Body Mass Index
RA-CA Difference
Log (RA / (CA*BMI))
C_AP_HR75 / (BMI*CA)
C_AGPH_HR75 / (BMI*CA)
Age Parameter
Chronologic Age (Years)
Dependency Status
Control
Opiate Dependenc

Figure 2

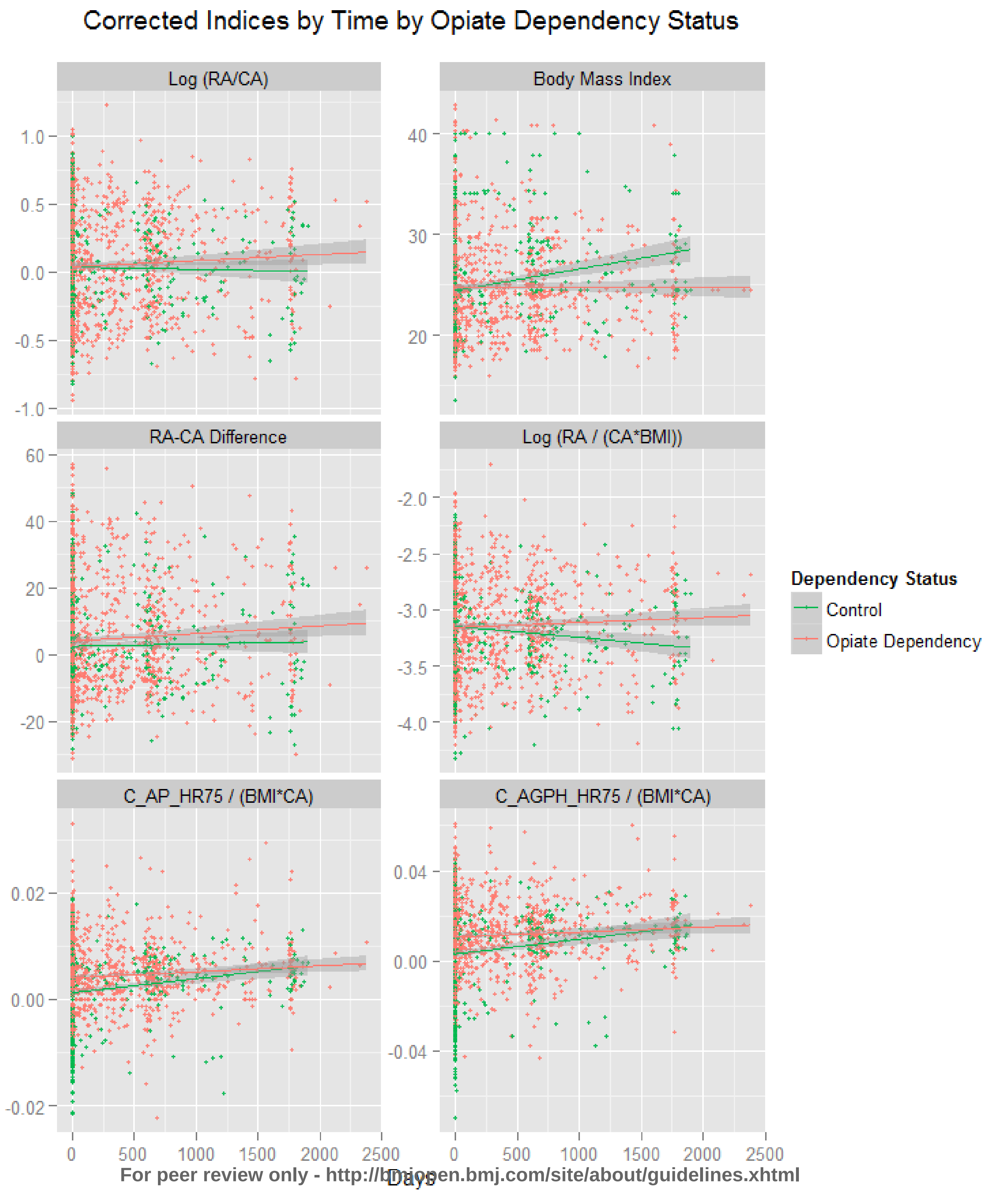

Corrected Indices by Time by Opiate Dependency Status
Log (RA/CA)
Body Mass Index
RA-CA Difference
Log (RA / (CA*BMI))
C_AP_HR75 / (BMI*CA)
C_AGPH_HR75 / (BMI*CA)
Parameter
Days
Dependency Status
Control
Opiate Dependency

Figure 3

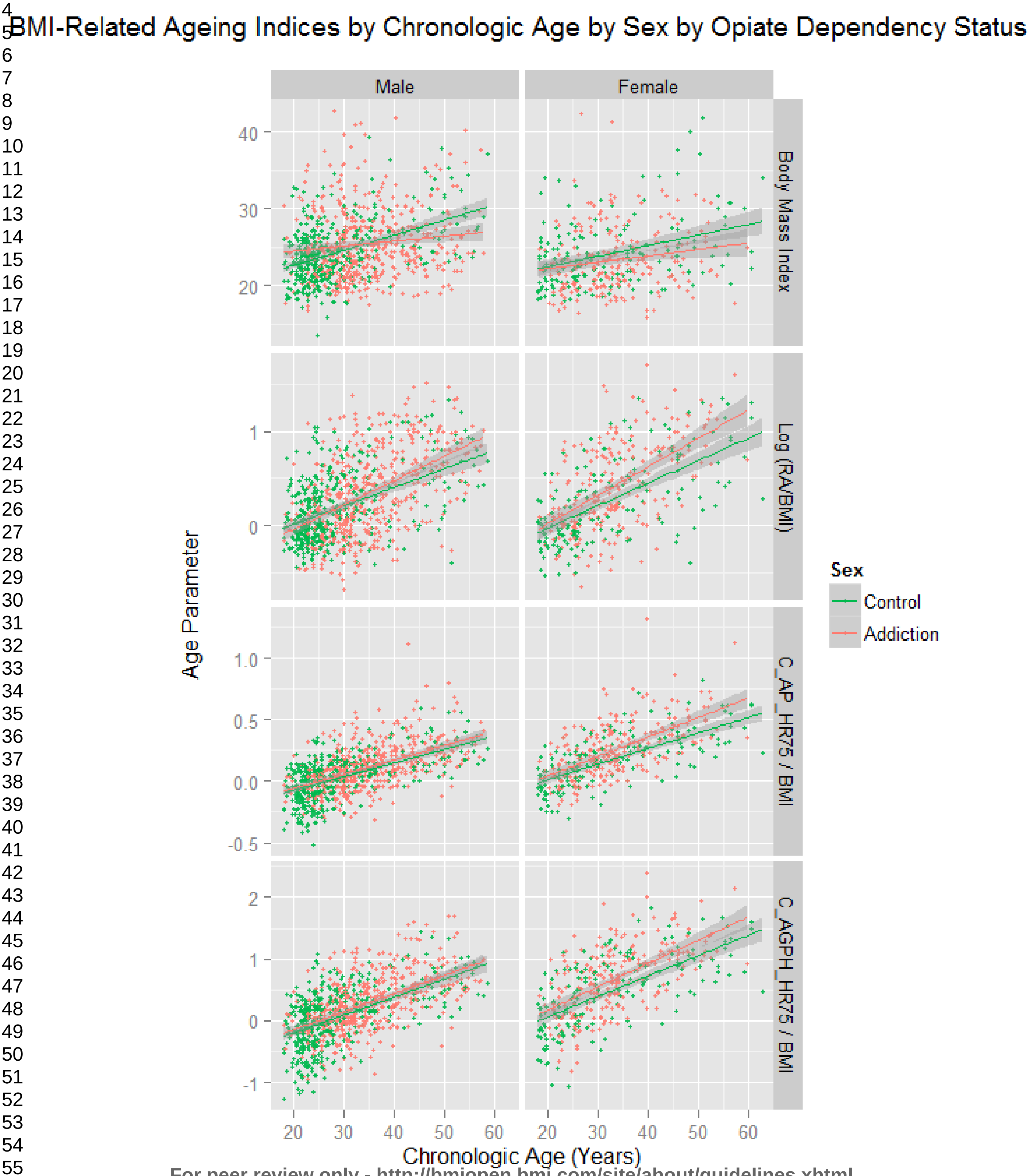
BMI-Related Ageing Indices by Chronologic Age by Sex by Opiate Dependency Status
Male
Female
Body Mass Index
Log (RA/BMI)
C_AP_HR75 / BMI
C_AGPH_HR75 / BMI
Age Parameter
Chronologic Age (Years)
Sex
Control
Addiction
20
30
40
50
60

Figure 4

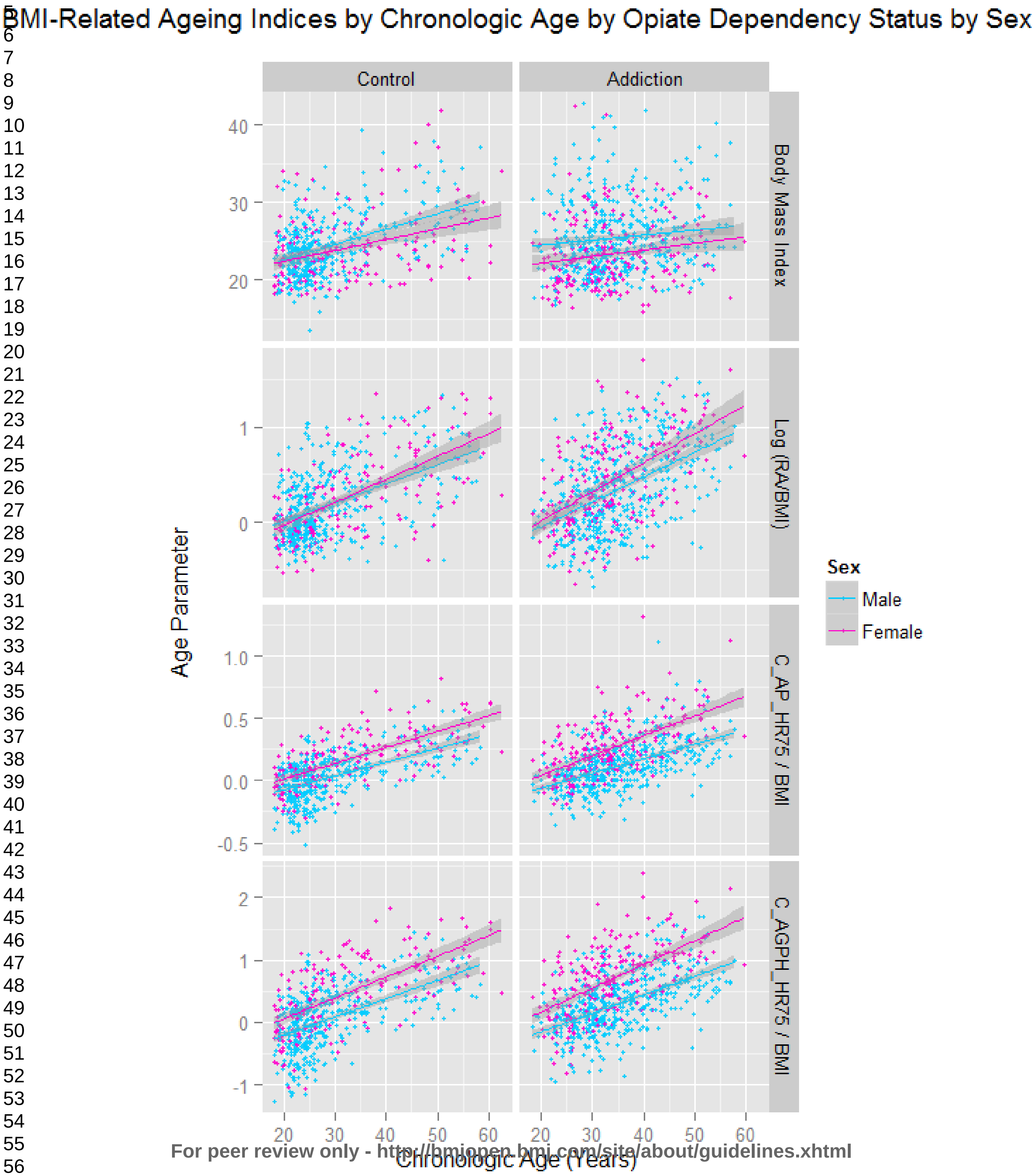
BMI-Related Ageing Indices by Chronologic Age by Opiate Dependency Status by Sex
Control
Addiction
Body Mass Index
Log (RA/BMI)
C_AP_HR75 / BMI
C_AGPH_HR75 / BMI
Age Parameter
Chronologic Age (Years)
Sex
Male
Female

Figure 5

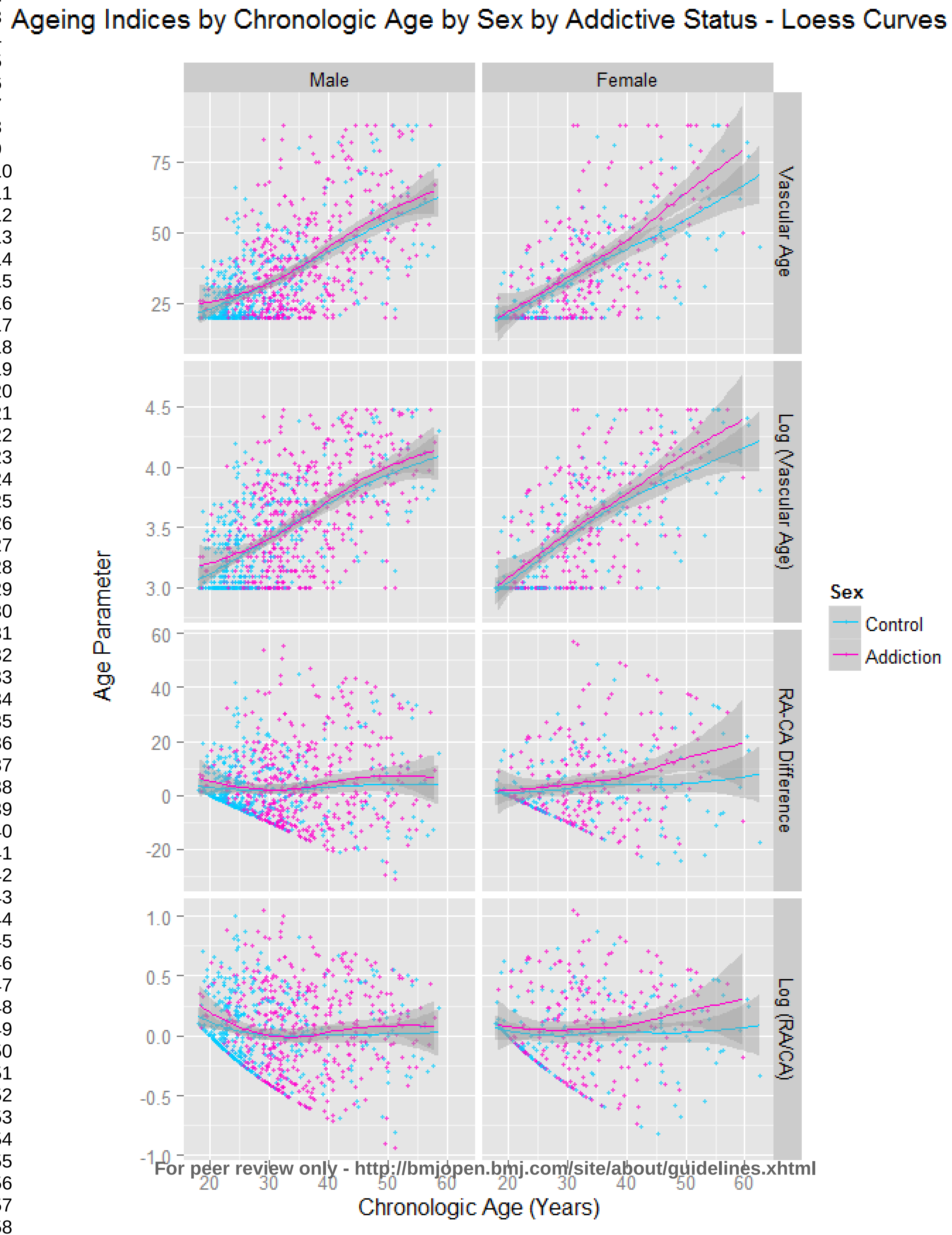

Ageing Indices by Chronologic Age by Sex by Addictive Status - Loess Curves
Male
Female
Vascular Age
Log (Vascular Age)
RA-CA Difference
Log (RA/CA)
Age Parameter
Chronologic Age (Years)
Sex
Control
Addiction

Figure 6

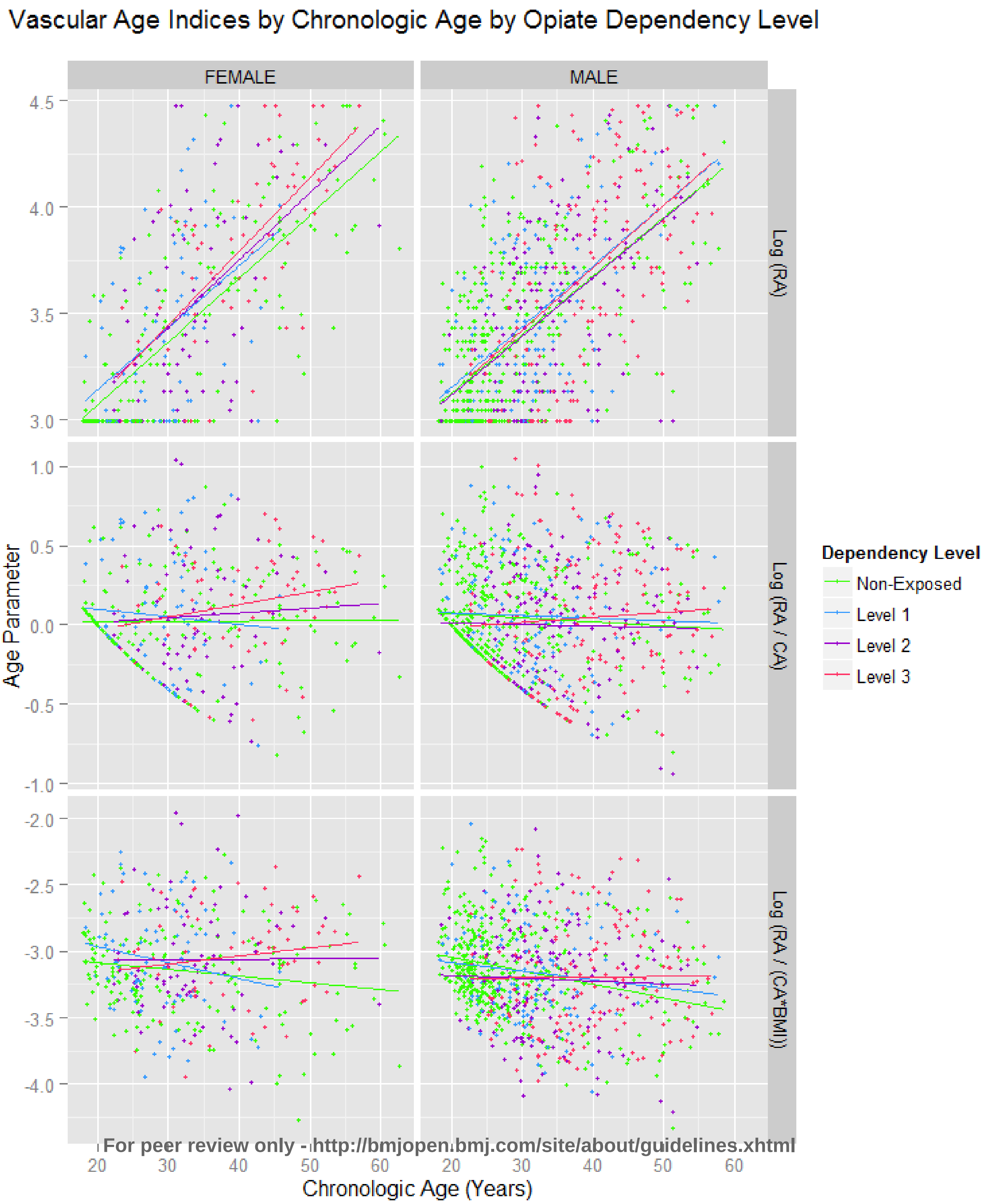
Vascular Age Indices by Chronologic Age by Opiate Dependency Level
FEMALE
MALE
Log (RA)
Log (RA / CA)
Log (RA / (CA*BMI))
Age Parameter
Chronologic Age (Years)
Dependency Level
Non-Exposed
Level 1
Level 2
Level 3

SUPPLEMENTARY TABLES

For peer review only

Supplementary Table 1.: Bivariate Comparisons of

Baseline Sociodemographic, Laboratory and Drug Use Variables

	Control	Opiate Dependence	t-value	df	P
Chronologic_Age (Yrs)	29.96_(0.45)	34.55_(0.32)	-8.262	1082.22	0.0000
Biometrics					
Height_(cm)	173.59_(0.37)	173.51_(0.33)	0.172	1261.00	0.8633
Weight_(kg)	73.21_(0.58)	74.98_(0.63)	-2.071	1260.00	0.0385
BMI_(kg/m^2)	24.25_(0.17)	24.8_(0.17)	-2.263	1256.60	0.0238
Drug Use					
Tobacco (%Smokers)	0.16_(0.02)	0.92_(0.01)	-41.558	1029.42	0.0000
Cigarettes/d	1.78_(0.22)	16.64_(0.37)	-34.982	1089.65	0.0000
Minutes After Smoking	121.98_(8.48)	96.48_(12.98)	1.645	757.57	0.1004
Heroin Dose (g/d)	0_(0)	0.53_(0.02)	-21.717	682.28	0.0000
Heroin Duration (Yr)	0.09_(0.05)	13.01_(0.33)	-38.783	695.26	0.0000
Lifetime Heroin Exposure (g-Yr)	0.03_(0.02)	7.81_(0.65)	-11.987	666.93	0.0000
Buprenorphine Dose (mg/d)	_()	6.98_(0.21)		586.00	
Methadone Dose (mg/d)	_()	63_(4.01)		66.00	
Laboratory Values					
Cholesterol (mmol/l)	4.78_(0.08)	4.45_(0.04)	3.497	781.00	0.0005
Triglyceride (mmol/l)	1.43_(0.08)	1.4_(0.04)	0.439	781.00	0.6608
HDL (mmol/l)	1.31_(0.04)	1.24_(0.02)	1.551	522.00	0.1215
LDL (mmol/l)	2.87_(0.1)	2.47_(0.04)	3.698	119.75	0.0003
ALT (IU/L)	32.8_(2.07)	65.59_(4.04)	-7.214	778.88	0.0000
AST (IU/L)	30.92_(3.9)	48.04_(2.26)	-3.793	318.80	0.0002
Glucose (mmol/l)	5.03_(0.14)	5.49_(0.2)	-1.231	785.00	0.2185
Creatinine (mcmol/l)	78.86_(1.28)	79.75_(0.68)	-0.634	785.00	0.5263
Urea (mmol/l)	5.18_(0.09)	5.12_(0.21)	0.155	783.00	0.8765
Albumin (g/L)	44.23_(0.32)	43.6_(0.2)	1.533	784.00	0.1256
Globulin (g/L)	29.3_(0.26)	31.53_(0.2)	-6.735	421.08	0.0000
CRP (mg/L)	3.43_(0.65)	6.11_(0.42)	-3.459	269.11	0.0006
ESR (mm/hr)	7.85_(0.59)	12.76_(0.5)	-6.374	365.75	0.0000
Lymphocytes (x10^9/ml)	2.32_(0.05)	2.54_(0.04)	-2.754	759.00	0.0060
Monocytes (x10^9/ml)	0.56_(0.02)	0.63_(0.01)	-2.404	629.00	0.0165
Calcium (mmol/l)	2.37_(0.01)	2.37_(0)	0.111	784.00	0.9119
Phosphate (mmol/l)	1.18_(0.02)	1.22_(0.01)	-2.491	334.08	0.0132
HBcAb Positivity Rate	0.17_(0.05)	0.25_(0.02)	-1.514	70.28	0.1344
HCV Ab Positivity Rate	0.52_(0.07)	0.82_(0.02)	-4.161	56.84	0.0001

Data presented as mean $\pm$ S.E.M.

For peer review only - http://bmjopen.bmj.com/site/about/guidelines.xhtml

Supplementary Table 2.:

Numbers of Longitudinal Studies

No. Studies	Groups		No. Studies		
	Control	Opiate Dependent	Control	Opiate Dependent	Total
1	456	388	456	388	844
2	45	144	90	288	378
3	12	68	36	204	240
4	6	36	24	144	168
5	3	15	15	75	90
6	4	10	24	60	84
7	2	5	14	35	49
8	2	5	16	40	56
9	0	1	0	9	9
10	1	1	10	10	20
11	0	1	0	11	11
12	0	1	0	12	12
13	0	1	0	13	13
16	0	1	0	16	16
25	1	0	25	0	25
Totals			710	1305	2015

Numbers of stuidies performed relate to the ease of follow-up of patients.

For peer review only

Supplementary Table 3.:

Bivariate Comparisons of Central and Peripheral Cardiovascular Parameters

	Control	Opiate Dependence	t-value	df	p
**Operator Index**	88.58_(0.26)	88.34_(0.25)	0.683	1261.00	0.4946
Ages					
Chronologic_Age	29.96_(0.45)	34.55_(0.32)	-8.262	1082.22	0.0000
Vascular_Age	32.57_(0.68)	39.12_(0.7)	-6.729	1255.67	0.0000
Log(CA)	3.35_(0.01)	3.51_(0.01)	-10.375	1076.20	0.0000
Log(RA)	3.39_(0.02)	3.57_(0.02)	-7.448	1242.33	0.0000
RA-CA_Difference	2.61_(0.47)	4.57_(0.57)	-2.652	1245.27	0.0081
RA/CA	1.09_(0.01)	1.13_(0.02)	-1.821	1259.62	0.0688
Augmentation					
C_AP_HR75	1.86_(0.21)	3.94_(0.19)	-7.351	1260.00	0.0000
C_AGPH_HR75	4.78_(0.56)	10.17_(0.46)	-7.503	1260.00	0.0000
C_PH	35.29_(0.29)	37.36_(0.28)	-5.123	1261.00	0.0000
C_T1R	154.76_(0.65)	152.42_(0.54)	2.783	1233.00	0.0055
C_AL	8.25_(0.23)	9.14_(0.18)	-3.078	1037.00	0.0021
C_ATI	49.83_(1.6)	57.02_(1.32)	-3.488	1037.00	0.0005
PPAmpRatio	156.97_(0.79)	150.79_(0.74)	5.686	1260.00	0.0000
P_MAX_DPDT	844.69_(7.35)	850.89_(7.16)	-0.601	1261.00	0.5478
Timing					
HR	69.26_(0.47)	69.99_(0.45)	-1.100	1261.00	0.2714
ED	324.74_(0.84)	323.5_(0.82)	1.054	1261.00	0.2923
C_SVI	144.72_(1.29)	142.43_(1.11)	1.356	1261.00	0.1754
C_DTI	3015.42_(16.3)	3023.56_(15.82)	-0.358	1243.62	0.7201
C_TTI	2151.42_(15.84)	2186.99_(15.28)	-1.609	1261.00	0.1079
Pressure					
SP	123.29_(0.47)	123.77_(0.51)	-0.696	1260.87	0.4862
DP	68.6_(0.38)	68.18_(0.38)	0.775	1255.83	0.4383
C_SP	105.16_(0.45)	106.99_(0.46)	-2.836	1254.81	0.0046
C_DP	69.92_(0.38)	69.69_(0.39)	0.432	1256.15	0.6657
C_ESP	92.72_(0.47)	94.54_(0.46)	-2.769	1261.00	0.0057
C_MEANP	86.19_(0.39)	86.93_(0.4)	-1.305	1256.22	0.1920

Data presented as mean ± S.E.M.

For peer review only

Supplementary Table 4:

Cross-sectional Factor Analysis

lm(log (RA/BMI) ~ log(CA)*Addictive Status)

Parameter	Variable Statistics				Model Summaries		
	Estimate	Std. Error	t Value	Pr(>|t|)	F	dF	P
All Patients							
Opiates	-0.58071	0.2615	-2.221	0.0265	184.8	3, 1258	<2.2E-16
CA: Opiates	0.1756	0.0757	2.32	0.0205			
Males							
Opiates	-0.73731	0.3270	-2.255	0.0244	109.1	3, 862	<2.2E-16
CA: Opiates	0.2128	0.0950	2.24	0.0253			
Females							
CA: Opiates	0.0300	0.0109	2.747	0.0063	116.7	2, 393	<2.2E-16

For peer review only

Supplementary Table 5.:

Longitudinal Factor Analysis

lme(log (RA/CA) ~ log(CA)*Days*Addictive Status)

Group / Parameter	Parameter Values					Model Parameters	
	Value	Std.Error	DF	t-value	Pr(>|t|)	AIC	LogLik
All Patients							
CA:Days	0.00015	0.00006	811	2.5762	0.0102	1124.528	-555.2641
Days:Opiates	0.00010	0.00003	811	2.9978	0.0028		
Males							
Days:Control	-0.00001	0.00003	551	-0.2042	0.8383	780.7702	-384.3851
Days:Opiates	0.00005	0.00002	551	2.6199	0.009		
Females							
Days:Opiates	-0.00115	0.00052	256	-2.2160	0.0276	413.4612	-198.7306
CA:Days:Opiates	0.00032	0.00014	256	2.2185	0.0274		

For peer review only

Supplementary Table 6.:

Cross-sectional Factor Analysis

Interactions of Sex with CA.

Variable	Variable Statistics					Model Summaries	
	Parameter	Estimate	Std. Error	z - Value	P, Prob.(>|z|)	LogLik / F	Wald / dF
RA	Male.Sex	0.94149	0.32377	2.908	0.00364	-787.6	813.7
RA	CA: Male.Sex	-0.26694	0.09293	-2.873	0.00407	-787.6	813.7
C_AP_HR75	CA: Male.Sex	-0.87594	0.06461	-13.56	<2E-16	580.8	2, 1259
C_AGPH_HR75	CA: Male.Sex	-2.4701	0.1626	-15.2	<2E-16	75.55	3, 1259
C_PH	Male.Sex	21.987	4.682	4.696	2.94E-06	75.55	2, 1259
C_PH	CA: Male.Sex	-5.63	1.354	-4.158	3.43E-05	75.55	2, 1259
PPAmpRatio	CA: Male.Sex	2.6901	0.2569	10.47	<2E-16	483.6	2, 1259
C_T1R	Male.Sex	9.6358	0.8174	11.79	<2E-16	130.6	2, 1232

All Interactive Models, except for C_T1R
All model P-values < 2.0E-16
Model for RA is tobit (with LogLik and Wald Statistics). All others are standard linear regression models with CA.

For peer review only

Supplementary Table 7.:

Longitudinal Factor Analysis

Interactions of Time, CA and Sex.

Group	Variable Statistics					Model	
Parameter	Value	Std.Error	DF	t-value	p-value	AIC	LogLik
RA							
CA: Days: Opiates	2.5E-05	9.0E-06	809	2.7328	0.0064	1149.41	-564.7
Days	-0.0005	0.0002	809	-2.5125	0.0122		
CA: Days	0.0001	0.0001	809	2.3973	0.0167		
C_AGPH_HR75							
CA	30.8204	1.6476	804	18.7068	0.0000	14393.8	-7181.9
Days: Male.Sex	-0.0356	0.0117	804	-3.0428	0.0024		
CA: Days: Male.Sex	0.0087	0.0030	804	2.8711	0.0042		
CA: Male.Sex	-5.1991	2.0124	804	-2.5836	0.0100		
Days: Male.Sex: Opiates	0.0335	0.0142	804	2.3631	0.0184		
CA: Days: Female.Sex: Opiates	0.0124	0.0058	804	2.1522	0.0317		
CA: Days: Male.Sex: Opiates	-0.0079	0.0037	804	-2.1274	0.0337		
Days: Female.Sex: Opiates	-0.0440	0.0218	804	-2.0213	0.0436		
C_AP_HR75							
Days: Male.Sex	-0.0198	0.0049	806	-4.0071	0.0001	10866.4	-5420.2
CA: Days: Male.Sex	0.0050	0.0013	806	3.9398	0.0001		
CA: Days: Opiates	0.0005	0.0002	806	2.8924	0.0039		
Days: Female.Sex	-0.0121	0.0045	806	-2.6830	0.0074		
CA: Days: Male.Sex: Opiates	-0.0041	0.0016	806	-2.5760	0.0102		
CA: Days: Female.Sex	0.0030	0.0012	806	2.5498	0.0110		
Days: Male.Sex: Opiates	0.0150	0.0060	806	2.5092	0.0123		
CA: Male.Sex	-2.0296	0.8114	806	-2.5013	0.0126		
C_PH							
Male.Sex	27.1005	5.0888	1198	5.3255	0.0E+00	13297.5	-6633.7
CA: Male.Sex	-7.0598	1.4690	804	-4.8058	0.0E+00		
Days	-0.0306	0.0085	804	-3.6178	0.0003		
CA: Days	0.0080	0.0022	804	3.6326	0.0003		
Days: Male.Sex: Opiates	0.0298	0.0106	804	2.8028	0.0052		
Opiates	13.5294	4.8766	804	2.7743	0.0057		
CA: Days: Male.Sex: Opiates	-0.0079	0.0029	804	-2.6945	0.0072		
CA: Opiates	-3.6162	1.4128	804	-2.5596	0.0107		

For peer review only

Supplementary Table 8.:

Significance Levels for Quartile P Values <0.1

Cross-sectional and Longitudinal Studies

Cross-sectional Studies

**Group / Parameter**	**Parameter Values**				**Model Parameters**		
	**Estimate**	**Std. Error**	**t Value**	**Pr(>|t|)**	**F**	**dF**	**P**
All Patients							
H.Exposure.Level.3	-0.7103	0.3845	-1.847	0.0650	1.126	7, 1235	0.3437
CA: H.Exposure.Level.3	0.2056	0.1069	1.924	0.0546			
Males							
Females							
CA: H.Exposure.Level.3	0.0265	0.0139	1.911	0.0568	1.193	4, 388	0.3133

Longitudinal Studies

**Group / Parameter**	**Parameter Values**					**Model Parameters**	
	**Value**	**Std.Error**	**DF**	**t-value**	**Pr(>|t|)**	**AIC**	**LogLik**
All Patients							
Days: H.Exposure.Level.1	0.00013	0.00004	810	2.9797	0.0030	1152.85	-568.42
Days: H.Exposure.Level.2	0.00016	0.00004	810	3.6254	0.0003		
Days: H.Exposure.Level.3	0.00006	0.00004	810	1.7297	0.0841		
Males							
Days: H.Exposure.Level.1	0.00011	0.00005	549	2.2010	0.0281	828.94	-406.47
Days: H.Exposure.Level.2	0.00014	0.00005	549	2.5507	0.0110		
Females							
Days: H.Exposure.Level.1	-0.00176	0.00100	253	-1.7637	0.0790	475.43	-226.71
Days: H.Exposure.Level.3	-0.00203	0.00103	253	-1.9681	0.0501		
Days: CA: H.Exposure.Level.1	0.00050	0.00028	253	1.7653	0.0787		
Days: CA: H.Exposure.Level.3	0.00054	0.00028	253	1.9298	0.0547		

For peer review only

Supplementary Table 9.:

Summary of Longitudinal Models of Quartiles of Opiate Exposure

(lme (Variable/CA)~ Time*Opiate.Exposure.Quartile)

Parameter		Variable Statistics					Model	
**Group**	**Variable**	**Value**	**Std.Error**	**DF**	**t-value**	**P-value**	**AIC**	**LogLik**
RA/CA								
All Patients	Days: H.Exposure.Level.2	0.00007	0.00003	811	2.26807	0.0236	1143.78	-564.89
Males	Days: H.Exposure.Level.2	0.00007	0.00004	550	2.066028	0.0393	813.62	-399.81
Females	-							
C_AGPH_HR75 / CA								
All Patients	Days	0.00004	0.00001	811	3.372907	8E-04	1176.95	-581.48
	H.Exposure.Level.1	0.15146	0.02842	811	5.329696	0E+00		
	H.Exposure.Level.2	0.17167	0.02721	811	6.308965	0E+00		
	H.Exposure.Level.3	0.20433	0.02761	811	7.400001	0E+00		
Males	Days	0.00004	0.00001	550	2.861191	0.0044	645.42	-315.71
	H.Exposure.Level.1	0.14683	0.03182	550	4.614867	0E+00		
	H.Exposure.Level.2	0.15382	0.03017	550	5.099172	0E+00		
	H.Exposure.Level.3	0.21693	0.02999	550	7.233373	0E+00		
Females	Days	0.00004	0.00003	257	1.689176	0.0924	418.64	-202.3216
	H.Exposure.Level.1	0.09204	0.04998	257	1.841536	0.0667		
	H.Exposure.Level.2	0.17788	0.04887	257	3.640107	0.0003		
	H.Exposure.Level.3	0.17765	0.05221	257	3.402395	0.0008		
C_AP_HR75 / CA								
All Patients	Days	0.00002	0.00001	811	4.778954	0E+00	-2674.138	1344.069
	H.Exposure.Level.1	0.05655	0.01070	811	5.284006	0E+00		
	H.Exposure.Level.2	0.06166	0.01024	811	6.019479	0E+00		

	H.Exposure.Level.3	0.08000	0.01040	811	7.696224	0E+00		
Males	Days	0.00002	0.00001	550	4.104246	0E+00	-1943.355	978.6774
	H.Exposure.Level.1	0.05617	0.01229	550	4.570086	0E+00		
	H.Exposure.Level.2	0.05482	0.01164	550	4.710096	0E+00		
	H.Exposure.Level.3	0.08586	0.01157	550	7.424323	0E+00		
Females	Days	0.00002	0.00001	257	2.369984	0.0185	-806.9698	410.4849
	H.Exposure.Level.1	0.03297	0.01826	257	1.805227	0.0722		
	H.Exposure.Level.2	0.06411	0.01786	257	3.589983	0.0004		
	H.Exposure.Level.3	0.06855	0.01909	257	3.590865	0.0004		
C_PH / CA								
All Patients	Days	-0.00004	0.00001	811	-4.989103	0E+00	-215.7032	114.8516
	H.Exposure.Level.1	-0.01120	0.02041	811	-0.548578	0.5834		
	H.Exposure.Level.2	-0.10747	0.01955	811	-5.497201	0E+00		
	H.Exposure.Level.3	-0.18195	0.01984	811	-9.171153	0E+00		
Males	Days	-0.00004	0.00001	550	-3.439231	0.0006	-151.6348	82.81741
	H.Exposure.Level.1	-0.06511	0.02450	550	-2.657892	0.0081		
	H.Exposure.Level.2	-0.13868	0.02327	550	-5.958834	0E+00		
	H.Exposure.Level.3	-0.22848	0.02315	550	-9.86833	0E+00		
Females	Days	-0.00006	0.00002	257	-3.811813	0.0002	-76.14242	45.07121
	H.Exposure.Level.1	0.12933	0.03421	257	3.78028	0.0002		
	H.Exposure.Level.2	-0.02369	0.03343	257	-0.708426	0.4793		
	H.Exposure.Level.3	-0.06000	0.03567	257	-1.681998	0.0938		

For peer review only

Supplementary Table 10.:

ANOVA Comparisons of Cross-Sectional Models Polynomial in Opiate Duration

In Table 1.

Model	RSS	
m1	84.517	
m2	83.888	
m3	84.014	
m4	84.517	
m1234	83.731	
**Comparison**	**F**	**P-value**
m1 v m2	4.9694	0.02613
m1 v m3	3.9729	0.04665
m1 v m4	-	-
m2 v m3	-	-
m2 v m4	4.9694	0.02613
m3 v m4	3.9729	0.04665
m1234 v m1	1.2372	0.2899
m1234 v m2	0.3093	0.8718
m1234 v m3	0.5559	0.6948
m1234 v m4	1.2372	0.2899

RSS – Residual Sum of Squares
m1 - Opiate Exposure Duration - Linear Model
m2 - Opiate Exposure Duration - Quadratic Model
m3 - Opiate Exposure Duration - Cubic Model
m4 - Opiate Exposure Duration - Quartic Model
m1234 - Opiate Exposure Duration – Combined Linear, Quadratic, Cubic and Quartic Model

For peer review only

Supplementary Table 11.:

Summary of Longitudinal Models Polynomial in Opiate Duration

Model	Variable Statistics					Model	
**Parameter**	**Value**	**Std.Error**	**DF**	**t-value**	**p-value**	**AIC**	**LogLik**
1 Order- Linear							
H.Dose:H.Dura.n	0.0045	0.0013	814	3.5512	0.0004	1068.74	-530.37
2 Order- Quadratic							
H.Dura.n^2	-0.0009	0.0003	809	-3.5503	0.0004	1138.29	-560.15
H.Dura.n: H.Dura.n^2	2.9E-05	0.00000838	809	3.4714	0.0005		
H.Dose:H.Dura.n: H.Dura.n^2	-0.0001	0.00002745	809	-3.2360	0.0013		
H.Dose: H.Dura.n^2	0.0041	0.0014	809	2.9052	0.0038		
H.Dose	0.1659	0.0830	809	1.9994	0.0459		
H.Dose:H.Dura.n	-0.0407	0.0204	809	-1.9982	0.0460		
3 Order - Cubic							
H.Dura.n^3	0.0000	1.2526E-05	811	-3.3596	0.0008	1156.22	-571.11
H.Dose: H.Dura.n^3	0.0001	1.9606E-05	811	4.0694	0.0001		
H.Dura.n: H.Dura.n^3	1.3E-06	3.94E-07	811	3.3670	0.0008		
H.Dose:H.Dura.n: H.Dura.n^3	-2.4E-06	6.14E-07	811	-3.8553	0.0001		
4 Order- Quartic							
H.Dose	0.0924	0.0358	810	2.5811	0.0100	1171.45	-577.73
H.Dura.n	-0.0065	0.0019	810	-3.3983	0.0007		
H.Dose: H.Dura.n^4	1.9E-06	0.0000006	810	3.0901	0.0021		
H.Dura.n: H.Dura.n^4	1.0E-08	0.0E+00	810	2.9797	0.003		
H.Dose:H.Dura.n: H.Dura.n^4	-6.0E-08	0.00000002	810	-3.0810	0.0021		
1, 2 & 4 Order							
H.Dura.n: H.Dura.n^2	-0.0023	0.0008	804	-3.0322	0.0025	1310.95	-641.48
H.Dose:H.Dura.n: H.Dura.n^2	0.0050	0.0017	804	3.0052	0.0027		
H.Dura.n^4	0.0002	0.00005279	804	3.0026	0.0028		
H.Dose: H.Dura.n^2	-0.0270	0.0092	804	-2.9399	0.0034		
H.Dose: H.Dura.n^4	-0.0003	0.0001	804	-2.9034	0.0038		
H.Dura.n: H.Dura.n^4	-4.5E-06	0.00000156	804	-2.8937	0.0039		
H.Dura.n^2	0.0110	0.0039	804	2.8136	0.0050		
H.Dura.n^2 : H.Dura.n^4	5.0E-08	0.00000002	804	2.7676	0.0058		
H.Dose:H.Dura.n: H.Dura.n^4	9.0E-06	0.00000327	804	2.7629	0.0059		
H.Dose: H.Dura.n^2 : H.Dura.n^4	-9.0E-08	0.00000003	804	-2.6261	0.0088		
H.Dose	0.1584	0.0714	804	2.2175	0.0269		

For peer review only

Supplementary Table 12.:

ANOVA Comparison of Longitudinal Models of Table 10

Model	AIC	LogLik
m1	1049.67	-520.83
m2	1040.85	-511.42
m3	1043.21	-514.60
m4	1043.11	-513.56
m124	1041.09	-506.54
**Comparison**	**L.Ratio**	**P-value**
m1 v m2	18.82	0.0021
m1 v m3	12.46	0.0060
m1 v m4	14.55	0.0057
m2 v m3	6.36	0.0416
m2 v m4	4.27	0.0388
m3 v m4	2.09	0.1482
m124 v m1	28.58	0.0015
m124 v m2	9.76	0.0824
m124 v m3	16.12	0.0241
m124 v m4	14.03	0.0294

AIC – Akiane Information Criteria
Log Lik. - Log Likelihood Ratio
L. Ratio - Log Likelihood Ratio
m1 - Opiate Exposure Duration - Linear Model
m2 - Opiate Exposure Duration - Quadratic Model
m3 - Opiate Exposure Duration - Cubic Model
m4 - Opiate Exposure Duration - Quartic Model
m124 - Opiate Exposure Duration – Combined Linear, Quadratic, and Quartic Model

For peer review only

Supplementary Table 13.:

Full Multivariate Analysis of Log (RA/CA) Including All Biochemical Variables

Parameter	Variable Statistics				Model Summaries		
	Estimate	Std. Error	t Value	Pr(>|t|)	F	dF	P
Both Sexes							
H.Dura.n: Height: SP : CRP	0.0003	0.0001	2.835	0.0048	3.030	12, 381	0.00044
H.Dura.n: Height: CRP	-0.0014	0.0005	-2.82	0.0051			
H.Dose: CRP	6.2416	2.3936	2.608	0.0095			
H.Dose: SP : CRP	-1.2903	0.4964	-2.599	0.0097			
H.Dose: Height: Cigs.d: SP	0.0004	0.0001	2.487	0.0133			
H.Dose: Height: Cigs.d	-0.0017	0.0007	-2.432	0.0155			
H.Dose: Cigs.d: SP : CRP	0.5385	0.2346	2.295	0.0223			
H.Dose: Cigs.d: CRP	-2.5285	1.1265	-2.245	0.0254			
H.Dose: Height: Cigs.d: SP : CRP	-0.0029	0.0013	-2.137	0.0332			
H.Dura.n: Cigs.d: SP : CRP	-0.0020	0.0010	-2.103	0.0361			
H.Dose: Height: Cigs.d: CRP	0.0134	0.0065	2.082	0.0380			
H.Dura.n: Cigs.d: CRP	0.0096	0.0046	2.079	0.0383			
Males							
H.Dose: Height: Cigs.d: CRP	-0.0007	0.0002	-3.534	0.0005	3.323	10, 264	0.0004
H.Dose: Cigs.d: SP : CRP	0.0214	0.0066	3.224	0.0014			
H.Dura.n: Height: CRP	-0.0008	0.0003	-2.984	0.0031			
H.Dura.n: Height: SP : CRP	0.0002	0.0001	2.969	0.0033			
H.Dose: Height: CRP	0.0231	0.0081	2.863	0.0045			
H.Dose: Height: SP : CRP	-0.0046	0.0017	-2.723	0.0069			
H.Dose: Height: Cigs.d: SP	0.0004	0.0001	2.716	0.0070			
H.Dose: Height: Cigs.d	-0.0019	0.0007	-2.583	0.0103			
Cigs.d: CRP	0.1028	0.0450	2.284	0.0232			
Cigs.d: SP : CRP	-0.0208	0.0093	-2.236	0.0262			
Females							
Cigs.d: CRP	-0.12299	0.04178	-2.944	0.00392	4.484	2, 116	0.01331
Cigs.d: SP : CRP	0.02583	0.00883	2.926	0.00413			

For peer review only

Suppl. Figure 1

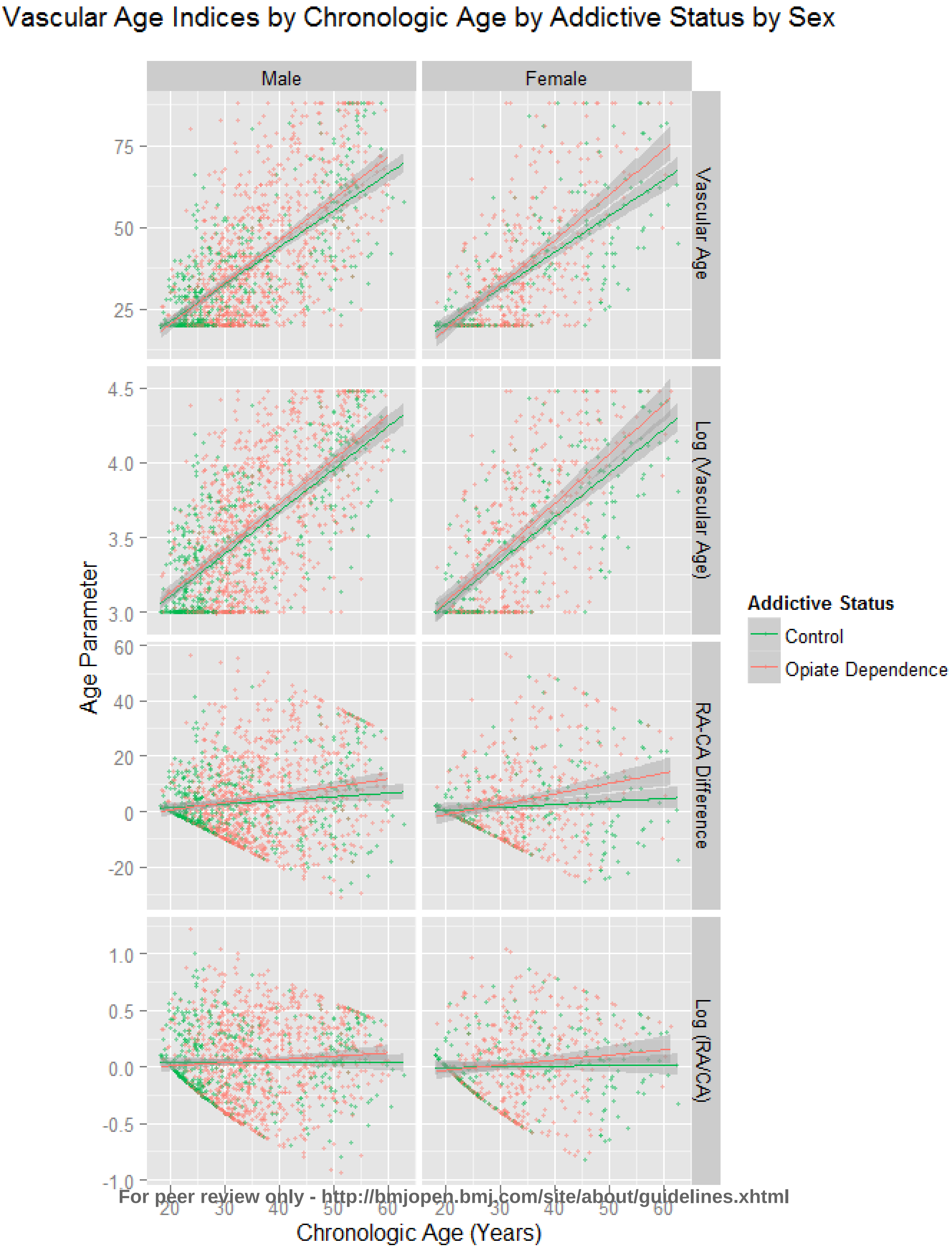

Suppl. Figure 2

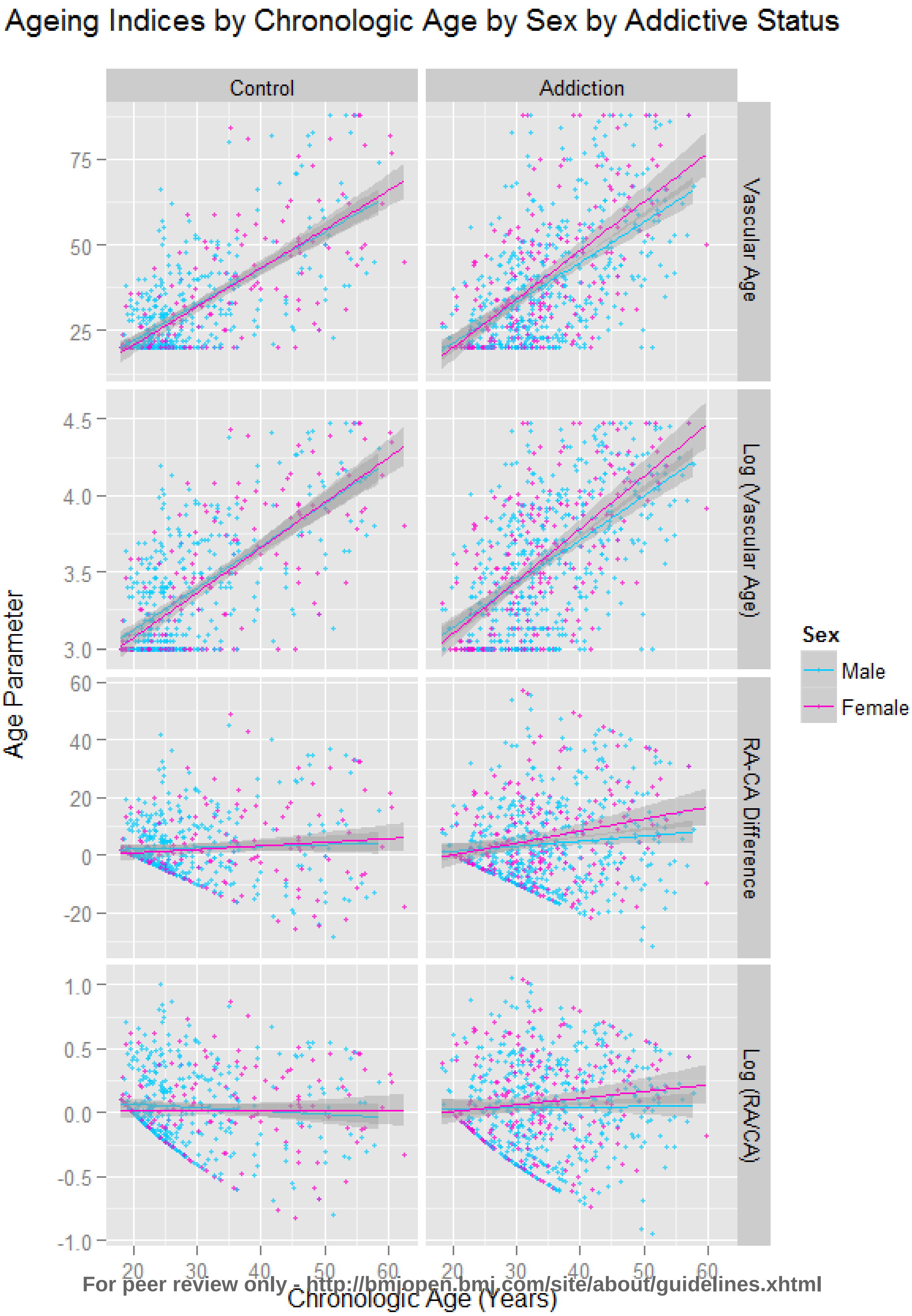

Suppl. Fig 3: Quartiles Long Logs by Days

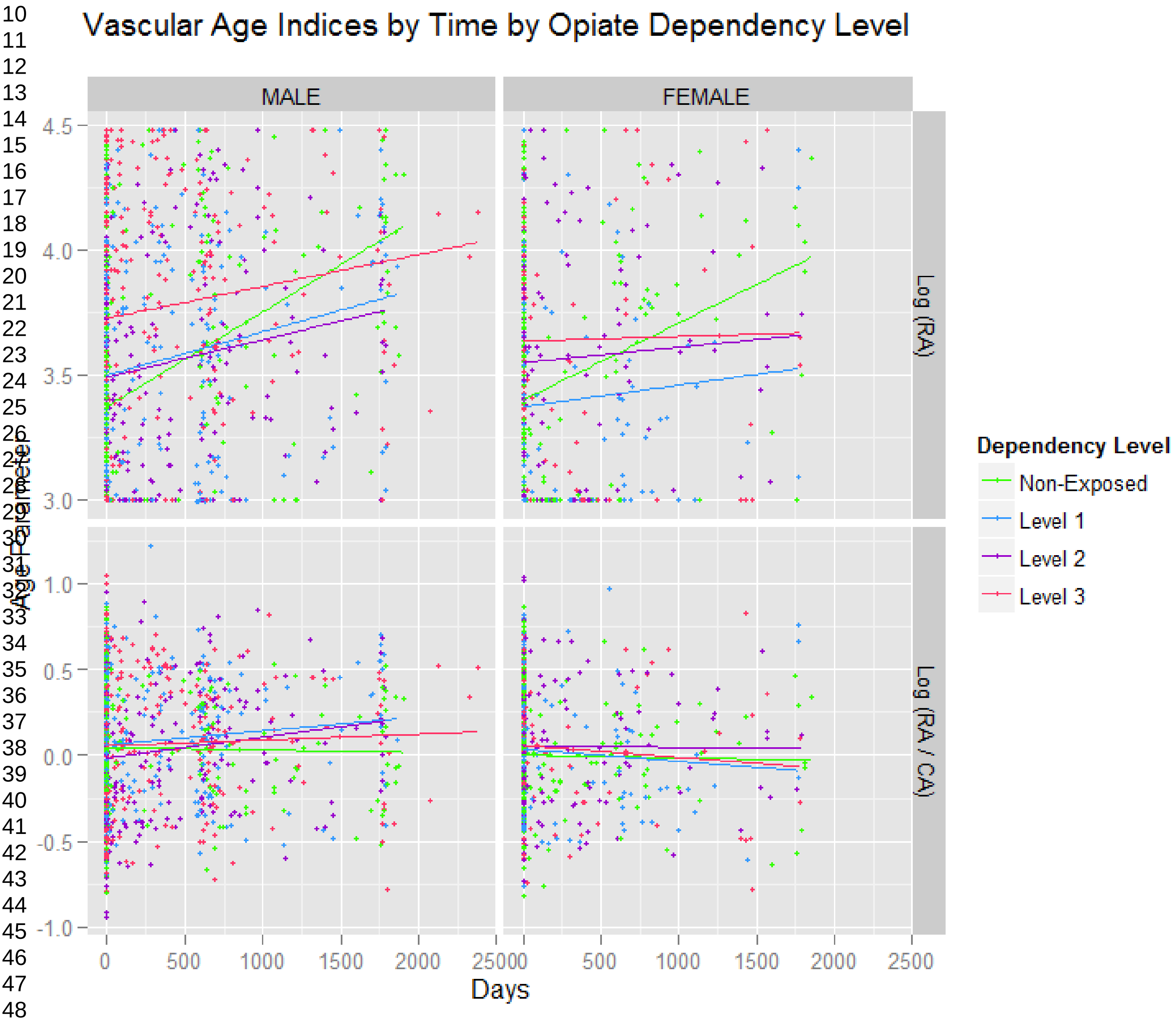

STROBE Statement—checklist of items that should be included in reports of observational studies

DEAR EDITOR,

PLEASE NOTE THAT ALKLK OF THESE HAVE BEEN MET IN OUR ACCOMPANYING ARTILCE.

THANKYOU,

DR STUART REECE.

For peer review only

	Item No	Recommendation
**Title and abstract**	1	(*a*) Indicate the study's design with a commonly used term in the title or the abstract
		(*b*) Provide in the abstract an informative and balanced summary of what was done and what was found
**Introduction**		
Background/rationale	2	Explain the scientific background and rationale for the investigation being reported
Objectives	3	State specific objectives, including any prespecified hypotheses
**Methods**		
Study design	4	Present key elements of study design early in the paper
Setting	5	Describe the setting, locations, and relevant dates, including periods of recruitment, exposure, follow-up, and data collection
Participants	6	(*a*) *Cohort study*—Give the eligibility criteria, and the sources and methods of selection of participants. Describe methods of follow-up<br>*Case-control study*—Give the eligibility criteria, and the sources and methods of case ascertainment and control selection. Give the rationale for the choice of cases and controls<br>*Cross-sectional study*—Give the eligibility criteria, and the sources and methods of selection of participants
		(*b*) *Cohort study*—For matched studies, give matching criteria and number of exposed and unexposed<br>*Case-control study*—For matched studies, give matching criteria and the number of controls per case
Variables	7	Clearly define all outcomes, exposures, predictors, potential confounders, and effect modifiers. Give diagnostic criteria, if applicable
Data sources/ measurement	8*	For each variable of interest, give sources of data and details of methods of assessment (measurement). Describe comparability of assessment methods if there is more than one group
Bias	9	Describe any efforts to address potential sources of bias
Study size	10	Explain how the study size was arrived at
Quantitative variables	11	Explain how quantitative variables were handled in the analyses. If applicable, describe which groupings were chosen and why
Statistical methods	12	(*a*) Describe all statistical methods, including those used to control for confounding
		(*b*) Describe any methods used to examine subgroups and interactions
		(*c*) Explain how missing data were addressed
		(*d*) *Cohort study*—If applicable, explain how loss to follow-up was addressed

Case-control study—If applicable, explain how matching of cases and controls was addressed

Cross-sectional study—If applicable, describe analytical methods taking account of sampling strategy

(e) Describe any sensitivity analyses

Continued on next page

For peer review only

For peer review only - http://bmjopen.bmj.com/site/about/guidelines.xhtml

**Results**		
Participants	13*	(a) Report numbers of individuals at each stage of study—eg numbers potentially eligible, examined for eligibility, confirmed eligible, included in the study, completing follow-up, and analysed
		(b) Give reasons for non-participation at each stage
		(c) Consider use of a flow diagram
Descriptive data	14*	(a) Give characteristics of study participants (eg demographic, clinical, social) and information on exposures and potential confounders
		(b) Indicate number of participants with missing data for each variable of interest
		(c) *Cohort study*—Summarise follow-up time (eg, average and total amount)
Outcome data	15*	*Cohort study*—Report numbers of outcome events or summary measures over time
		Case-control study—Report numbers in each exposure category, or summary measures of exposure
		Cross-sectional study—Report numbers of outcome events or summary measures
Main results	16	(*a*) Give unadjusted estimates and, if applicable, confounder-adjusted estimates and their precision (eg, 95% confidence interval). Make clear which confounders were adjusted for and why they were included
		(*b*) Report category boundaries when continuous variables were categorized
		(*c*) If relevant, consider translating estimates of relative risk into absolute risk for a meaningful time period
Other analyses	17	Report other analyses done—eg analyses of subgroups and interactions, and sensitivity analyses
**Discussion**		
Key results	18	Summarise key results with reference to study objectives
Limitations	19	Discuss limitations of the study, taking into account sources of potential bias or imprecision. Discuss both direction and magnitude of any potential bias
Interpretation	20	Give a cautious overall interpretation of results considering objectives, limitations, multiplicity of analyses, results from similar studies, and other relevant evidence
Generalisability	21	Discuss the generalisability (external validity) of the study results
**Other information**		
Funding	22	Give the source of funding and the role of the funders for the present study and, if applicable, for the original study on which the present article is based

*Give information separately for cases and controls in case-control studies and, if applicable, for exposed and unexposed groups in cohort and cross-sectional studies.

Note: An Explanation and Elaboration article discusses each checklist item and gives methodological background and published examples of transparent reporting. The STROBE checklist is best used in conjunction with this article (freely available on the Web sites of PLoS Medicine at http://www.plosmedicine.org/, Annals of Internal Medicine at http://www.annals.org/, and Epidemiology at http://www.epidem.com/). Information on the STROBE Initiative is available at www.strobe-statement.org.

For peer review only

BMJ Open

Impact of Lifetime Opioid Exposure on Arterial Stiffness and Vascular Age: Cross-sectional and Longitudinal Studies in Males and Females.

Journal:	*BMJ Open*
Manuscript ID:	bmjopen-2013-004521.R2
Article Type:	Research
Date Submitted by the Author:	05-May-2014
Complete List of Authors:	Reece, Albert; University of Western Australia, School of Psychiatry and Clinical Neurosciences<br>Hulse, Gary; University of Western Australia, Psychiatry
**Primary Subject Heading**:	Pharmacology and therapeutics
Secondary Subject Heading:	Cardiovascular medicine, Addiction, Neurology, Public health, Immunology (including allergy)
Keywords:	arterial stiffness, opiate dependence, vascular ageing, biological age, acceleration of ageing, heroin

SCHOLARONE™ Manuscripts

For peer review only

Impact of Lifetime Opioid Exposure on Arterial Stiffness and Vascular Age: Cross-sectional and Longitudinal Studies in Males and Females.

Short Title:

Opioids as Cardiovascular Risk Factors

Albert Stuart Reece

Gary Kenneth Hulse

School of Psychiatry and Clinical Neurosciences,

University of Western Australia.

Crawley WA 6009, Australia.

*** Correspondence to**:

Albert Stuart Reece

39 Gladstone Rd.,

Highgate Hill,

Brisbane, Queensland, Australia.

Ph: (617) 3844-4000

FAX: (617) 3844-4015

Email: sreece@bigpond.net.au

Word Count: 6,118.

Keywords – arterial stiffness, opioid dependence, vascular ageing, biological age, acceleration of ageing

For peer review only

Structured Abstract

Objective. To characterize and compare the potentiation of arterial stiffness and vascular ageing by opioids in males and females.

Design. Cross-sectional and longitudinal studies of 576 clinical controls and 687 opioid dependent patients (ODP) on 710 and 1,305 occasions over a total of 2,382 days (6.52 years), 2006-2011. Methodology: Radial Pulse Wave Analysis with Atcor SphygmoCor system (Sydney).

Setting. Primary care.

Participants. Controls: General practice patients with non-cardiovascular disorders, and university student controls. ODP: Patients undergoing clinical management of their opioid dependence. Controls had lower chronologic ages (CA) than ODP (30.0±0.5 vs. 34.5±0.3, mean±S.E.M., P<0.0001). Subjects were 69.6% and 67.7% male, and 16.0% and 92.3% were smokers (P<0.0001) for controls and ODP respectively. 86.3%, 10.3% and 3.4% of ODP were treated with buprenorphine (6.98±0.21mg), methadone (63.04±4.01mg) or implant naltrexone respectively. BMI was depressed in ODP.

Interventions. Nil.

Primary Outcome Measures - Vascular Reference Age (RA) and the ratio of the vascular age to the chronological age (RA/CA). Secondary outcome measures: Arterial Stiffness including Augmentation Index.

Results. After Body Mass Index -adjustment, RA in ODP was higher as a function of CA and of time (both P<0.05). Modelled mean RA in control and ODP males was 35.6 and 36.3 years (+1.97%), and in females 34.5 and 39.2 years (+13.43%). Changes in RA and major arterial stiffness indices were worse with CA in females (P<0.01). Quadratic, cubic and quartic functions of opioid exposure duration out-performed linear models with RA/CA over CA and over time. The opioid dose-response relationship persisted longitudinally after multiple adjustment from P=0.0013 in males and P=0.0073 in females.

For peer review only

Conclusion. Data show that lifetime opioid exposure is an interactive cardiovascular risk factor, particularly in females, is related to linear, quadratic, cubic and quartic functions of treatment duration and is consistent with other literature of accelerated ageing in OD patients.

For peer review only

Article Summary

Article Focus

- Despite striking consistency between several large epidemiological studies of the cardiovascular toxicity of long term opioid dependence, the subject is not well studied nor widely understood.

- There are only two (contradictory) studies of gender differences in significantly exposed populations in the published literature.

- The implications of organism-wide xenobiotic-induced inflammation have not been incorporated into the clinical practice of addiction medicine, our understanding of the inflammatory basis of atherogenesis, or the fact that vascular ageing is a major surrogate for systemic ageing across the whole organism.

Key Messages

- Opioid dependent patients (ODP) had and advance of mean vascular age from control of 35.61 to 36.31 years in males and 34.54 to 39.18 years in females which, after adjustment for BMI, were significant in both males and females cross-sectionally and longitudinally (all $P<0.05$). Both the acceleration of vascular ageing and arterial stiffness were worse in females ($P<0.01$) where it occurred 12 years earlier (29 vs. 41 years).

- A dose-response relationship between lifetime opioid exposure and measures of arterial stiffness and vascular age was demonstrated in both sexes, which was robust to multiple adjustment for known cardiovascular risk factors.

- Use of quadratic, cubic and quartic functions of opioid duration exposure significantly improved the statistical fit of models of the ratio of vascular age (RA) to chronological age (CA, RA/CA ratio), thereby further highlighting the significance of the duration for which opioid agonism is administered.

For peer review only

Strengths and Limitations

- With 576 controls and 687 OPD the large size of this study was a signficant advantage, as was its combined cross-sectional and longitudinal design, its ability to control for many cardiovascular risk factors, the sex specific analysis, and the diverse output of cardiovascular parameters of the Atcor / SphygmoCor Pulse Wave Analysis technology employed.

- The use of detailed statistical modelling of relationships between factors, dose-response modelling, polynomial modelling in opioid exposure duration, inter-quartile trend effects, and adjustment for known cardiovascular risk factors added further analytical power to the study.

- As the major study limitation was its lack of other measures of organism ageing and its lack of mechanistic and ageing medicine-specific biomarkers, further studies incorporating these refinements are indicated.

For peer review only

Introduction

Opioid dependence is a condition of increasing public health importance.

Opioid Dependence is associated with an impressive array of traditional and novel cardiovascular risk factors and associations including hypertension [1], hyperlipidaemia [2-3], tobacco consumption, increased body weight [4], hyperglycaemia [3], hyperfibrinogenaemia [3], diffuse polyclonal gammopathy and immune stimulation [5-6], elevated high sensitivity CRP [7], elevated cytokines [8], relative hypercalcaemia and hyperphosphataemia [9] and reduced circulating endothelial progenitor cells [10]. Furthermore opioid dependence has been associated with atherosclerotic disease in both the coronary [11-18] and cerebral [14 18] circulations. In a 33-year review of an opioid maintenance program in California in 2004, death from cardiovascular disease was noted to account for three times as many years of life lost in the treatment group than in the general population [18]. In 2007 an Iranian surgical group identified that opium dependent patients were at increased risk of requiring open coronary revascularization with an odds ratio of 1.8 after adjustment for other risk factors [15]. Moreover in this series there was a dose-response relationship between the amount of opioids consumed and the severity of the coronary disease. It was also shown that the coronary disease occurred two years earlier in opioid dependent persons than in controls [16]. This group further identified that opium use was the strongest cardiovascular risk factor in their population of men, with effects greater than traditional risk factors [17]. The sex specific finding in this study was felt to be related only to a reduced prevalence of opium consumption amongst females.

In reviewing over 800 fatal cases of opioid toxicity in Australia in 2006 Darke et al. found that 17% of decedents over the age of 44 years had coronary stenoses in excess of 75% [12]. Similarly when comparing 1193 post mortem cases of heroin and methadone toxicity the Sydney group found 2-3 times elevated adjusted odds ratios of ventricular hypertrophy, and myocardial, interstitial and perivascular fibrosis in the methadone treated group, consistent with the effects of more consistent opioid agonism in such patients [11]. In a 21-year follow-up of 42,676 patients undergoing maintenance opioid agonist treatment in Australia, including 425,998 person-years, Degenhardt et al. identified cardiovascular death as occurring 2.2

For peer review only

times as often as in the wider community [13]. Indeed in this study elevated rates of death with severe organ dysfunction were noted which appeared to act as a backdrop to the very high rates of death by overdose generally reported in such cohorts (see Web Appendix 6 of this citation).

In 2012, a prospective population based study of 50,045 people including 234,098 person-years reported hazard ratios of death from coronary artery disease (CAD) of 1.9-fold (95%C.I. 1.57-2.29) and of cerebrovascular disease (CeVD) of 1.68-fold (C.I. 1.29-2.18) [14]. Both were worse in females (CAD: male adjusted HR (AHR) 1.58, female AHR 2.90; CeVD male AHR 1.49, female AHR 1.97). An earlier study from Iran did not confirm the cardiovascular salience of opioid exposure amongst females as compared to males, but the number of exposed females in this study was small, which likely compromised the power of the analysis to detect an effect [17]. Furthermore recent reports of 1,263 patients found elevated rates of vascular age in patients treated with methadone compared to other agents [19] and improved vascular ages accompanying the long term drug-free condition [20].

One large case control study found a dose response relationship between opium exposure and the severity of angiographically assessed coronary artery disease in a diabetic population [21].

Moreover opioid patients dependent on short acting opioids such as morphine, oxycodone and heroin undergo the clinical syndrome of opioid withdrawal several times daily. This syndrome is a hyper-adrenergic state characterized by neurological and cardiovascular stimulation which in its more severe manifestations includes hypertension and tachycardia [22] and a reduction of subendocardial perfusion [23]. This negative aversive experience is one of the key reasons many opioid dependent patients (ODP) continue to use narcotics.

Important as the above collected findings are, other evidence suggests that the impact of elevated vascular age has implications on the health and ageing of the organism as a whole beyond those limited primarily to the cardiovascular system. It has been repeatedly proposed that vascular ageing is a principal component of biological organismal ageing, by virtue of the contribution made by cardiovascular causes to the overall death rate in developed nations, and also the importance of the vascular niche common to many stem cell beds [24-25]. This view closely accords with the finding in many of the above studies and particularly that by Degenhardt et al [13] that elevated rates of overdose occur on the background of advanced

For peer review only

disease in other organs [1 12 18]. Opioid dependent patients have many stigmata of accelerated ageing including increased rates of: hair greying, erosion of alveolar bone from chronic periodontitis [26], osteoporosis [27], chronic respiratory disease [11 13 18], chronic renal disease [11], diffuse senescent-mimetic immune stimulation [5-6 28], diabetes [3], neuropsychiatric disorders, multi-system disease [11-12], reduced circulating stem cell counts [10], and many malignancies [13 29]. Thus the call has been made for geriatricians to be appointed to the care of opioid dependent patients beyond the age of 50 years, due to the high prevalence of multi-system disease [1].

The possibility therefore arises that an improved understanding of the increased and accelerated profile of degenerative disease seen in opioid dependent patients may enhance our understanding, and in time our management, of age related disorders in the rest of the community.

As this clinic sees both opioid dependent patients and the general community, we were ideally situated to compare these two groups. The availability of the mobile SphygmoCor system from Atcor in Sydney means that a host of central cardiovascular data is available to the clinician in a non-invasive and rapid manner, providing quantitation of important subclinical endophenotypes of central vascular stiffness and cardiovascular and therefore organismal ageing. The central investigative question to be prospectively assessed was whether there were changes in major central cardiovascular parameters in the opioid dependent group, and if so are they robust to adjustment for other confounding factors. Several earlier papers from our group have considered this issue in both cross-sectional [30-31] and longitudinal [32-33] studies in each sex separately and drawn conclusions generally confirmatory of this hypothesis. It has been shown that the type of opioid pharmacotherapy affects the severity of opioid related potentiation of arterial stiffness [19], that induction of the drug free state after implantable opioid antagonists reverses this finding [20], and that opioid withdrawal is also associated with elevation of cardiovascular age and arterial stiffness parameters [23]. However a formal comparison of the effects by gender has not been possible in these earlier reports as they primarily considered single sex data. Gender comparison therefore forms the major focus of the present report which is a compilation of much of this earlier data.

Methods

For peer review only

Patient Selection. Control subjects (N=576) were recruited opportunistically from patients presenting for health examination checks for insurance or employment medical examinations or from patients presenting with minor health problems such as ear blockages, minor psychological disorders, or University students. Patients with known cardiovascular disorders such as hypertension, renal disease or diabetes or diseases known to perturb the CVS subacutely such as infections or pregnancy, were excluded. Opioid dependent patients (N=687) were also recruited opportunistically and were under treatment with either methadone (N=71), buprenorphine (N=593) maintenance, or naltrexone implant (N=23). All were sampled opportunistically at the time of their clinic visits May 2006 - December 2012.

Patient Treatment. Opioid dependent patients maintained on, methadone or buprenorphine were managed by accepted clinical algorithms by their usual health providers for their drug dependency. The naltrexone implants used for the management of heroin dependence were manufactured by "Go Medical" industries in Perth, Western Australia under Commonwealth of Australia Therapeutic Goods Administration (TGA) GMP conditions and administered under the compassionate Access arrangements of the Special Access Scheme authorized by the TGA as described elsewhere [34]. Patients were recalled for re-testing at two and five years post baseline assessment, and assessed opportunistically. Any patient with a known chronic or subacute cardiovascular condition were excluded from participation. Frequency and quantity of substance use by substance type (inclusive heroin) was assessed at the initial time and subsequently. Other opioids were converted into morphine equivalents, and then into heroin equivalents at the rate of 1g of street heroin = 0.5g morphine. The duration of opioid use was counted from the time of first use. If it became known that alcohol had been consumed in the few hours prior to the test or that stimulants had been used in the three days prior, or that patients were acutely affected by short acting opioids, that day's studies were excluded.

PWA Studies. Patients were positioned supine for the performance of the test and allowed to rest for five minutes prior to the test. Consumption of food, drink, alcohol or tobacco was not restricted, but patients were not allowed either to sleep or talk during the testing procedure. Pulse wave analysis by applanation tonometry was performed on the right radial artery unless

it was unavailable (due to trauma, surgery or congenital causes). The brachial blood pressure was taken in the opposite arm using the oscillometric Omron HEM 907 device. The data was collected and analyzed by the SphygmoCor software. Patients were studied in quintuplicate, and adequate studies were averaged for that day. Acceptable studies had an Operator index of greater than 70%, and were not inconclusive. Details of patients' prior drug use was also taken at the time of the study performance and entered into the database. Time in days was measured from the time of the first PWA study.

The SphygmoCor software used for the PWA studies calculates a number of major indices including Vascular Reference Age (RA), Chronologic Age (CA), Central Augmentation Pressure at Heart Rate 75 (C_AP_HR75) the Central Augmentation Pressure / Pulse Height Ratio at Heart Rate 75 (C_AGPH_HR75) also known as the Augmentation Index, Central Pulse Height (C_PH), Central Augmentation Load (C_AL), Central Augmentation Time Index (C_ATI), Peripheral–Central Pulse Pressure Amplification Ratio (PPAmpRatio), Maximal Peripheral Rise of dP/dT (P_MAX_DPDT), Central Systolic Pressure (C_SP), Central Diastolic Pressure (C_DP), Central End Systolic Pressure (C_ESP), Central Mean Pressure (C_MEANP), the Central Diastolic Time Index (C_DTI), the Central Tension Time index (C_TTI), and an index of subendocardial perfusion known variously as the Subendocardial Perfusion Ratio (SEVR), the Central Stroke Volume Index (C_SVI) or the Buckberg ratio defined as the C_TTI / C_DTI.

Statistics. Data are presented as the mean ± S.E.M. Categorical data were studied using "EpiInfo" 7.0.8.3 using the Chi squared test obtained from the CDC Atlanta, Georgia or the Fisher Exact test if the numbers in a cell were less than 20. Continuous categorized data were analyzed in "Statistica" 7.1 from Statsoft, Oklahoma using Student's t-tests. T-tests with separate variances were used where Levene's test was significant. Continuous variables such as RA, CA, RA/CA, variously corrected RA, SP, Weight, HDL, BMI, and systolic pressure were log transformed in accordance with normality assumptions based on the Shapiro-Wilks test. High sensitivity CRP and the heroin dose were arcsinh transformed which is a similar transform to logarithmic, but accepts arguments of zero. Time (as days) was measured from the time of the initial PWA study. The time since the last cigarette was recorded and analyzed as a factor with cut-off points at 30, 60, 120, 180 and > 180minutes, and a non-

For peer review only

smoking category. For some analyses lifetime heroin exposure was cut into quartile categories with divisions at 0, 0.01, 2.49, 6.59 and 350 g-years respectively, yielding 583, 207, 226 and 227 patients in each group respectively. Multiple regression was performed in "R" 2.15.2 obtained from the University of Melbourne Central "R" Archive Network mirror. Mean model estimates for various groups were prepared by inserting the mean parameter values into the untransformed final models. Multiple linear regression of truncated variables such as RA (minimum value of 20, maximum value of >80) were analyzed using tobit multiple regression techniques from the Applied Econometrics library in "R" (AER). RA's of individual studies returned as >80 were assigned as 88. Time-dependent repeated measures analysis was performed using non-linear mixed effects (nlme) regression module in "R" using restricted equation maximum likelihood (REML) techniques. Random effects were assigned to unity and the patient identification code. When nlme models were compared they were converted to maximum likelihood (ML) methods to allow Anova testing. In each case model reduction was performed manually by the classical method involving removing the least significant term. The log-likelihood value was abbreviated to LogLik. Graphs were prepared with reshape2 and ggplot2. Where loess curves were fitted, the span used was 0.95. T-tests were two-sided. $P<0.05$ was considered significant.

Ethical Approval. All patients provided their informed consent to participate in the study and for their medical treatment. The study was approved by the Human Research Committee of the Southcity Family Medical Centre, Brisbane, Queensland, Australia, which is registered with the National Health and Medical Research Council.

For peer review only

Results

576 control patients were compared with 687 opioid dependent patients. The mean ages were 30.0±0.5 and 34.5±0.3 (mean ± S.E.M.) years (t=-8.262, dF=1082.22, P<0.0001, Supplementary Table 1), with 401 (69.62%) and 465(67.69%) males respectively (Chi^2 = 0.54, P=0.46). 15.97% and 92.29% smoked tobacco (Chi^2=746.56, P<0.0001). The mean CA of the whole group was 32.5±0.3years. The mean CA of the males was 32.1±0.3 and of females 33.2±0.5 years. 7.47% and 5.97% of the control and opioid dependent groups were aged over 50 years respectively (Chi^2 .=1.13, P = 0.29). Amongst the males these ratios were 5.74% and 6.24% (Chi^2. =0.1), and amongst the females they were 11.43% and 5.41% (Chi^2. = 4.79, P = 0.029, Fisher Exact Test 0.19-0.98; pooled data, Corr. Chi^2= 3.8, P = 0.051). Racial stratification was not performed as our population is around 90% Caucasian in background. Other baseline biometric, drug use and laboratory values are given in Supplementary Table 1. Patients were studied longitudinally on 710 and 1,305 occasions. The period of repeat study, measured from the time of the first PWA study, was 624.11 ± 18.74 days (range 1-2,382 days; 495 (60.36%) studies performed beyond 360 days). Details of the numbers of repeat studies are given in Supplementary Table 2.

Comparative PWA values are given in Supplementary Table 3. Most augmentation values and the C_SP and C_ESP are noted to be higher in the opioid dependent group consistent with their being an average of 4.59 years older.

Figure 1 shows different vascular age and augmentation parameters corrected for various features, against CA. This figure shows that the BMI was lower in the opioid dependent group than the controls both as a factor (factor est.=0.0719, est. S.E. = 0.0327, t=2.197, P=0.0282) and in interaction with CA (BMI: CA est.=-0.0023, est. S.E. = 0.0009, t=-2.350, P=0.0189; model F=34.94, dF=3, 1258, P<0.0001). In the longitudinal series BMI was lower as a factor (est.=-0.0174, dF=812, P=0.0470; model AIC= -3722.55, LogLik=1867.27). This result may be the effect of cumulative diversion of resources over time. Figure 2 shows similar indices over time. Supplementary Table 4 presents the results of the cross-sectional data where BMI corrected RA is regressed against CA and addictive status. In the whole group both opioid dependency status (P=0.0265) and the CA: addiction interaction (P=0.0205) are significant. Similar results were found in each sex. Similar results were also found in the longitudinal study when the RA/CA ratio was regressed against CA, time and

For peer review only

dependency status both for opioid dependency as a factor (P=0.0102) and in interaction with CA (P=0.0028; details in Supplementary Table 4).

Inclusion of tobacco exposure along with opioid exposure in these models did not alter these findings (data not shown).

As shown in Supplementary Figures 1 and 2, these changes were found to be more pronounced with CA in females. These data are presented corrected for the changes in BMI in Figures 3 and 4. Details for these significant differences are presented in Table 1.

Figure 5 shows these data by sex with loess curves fitted (span=0.95). This strongly suggests that an inflection point is reached about the age of 30 in females, which is not seen in males until about 40 years of age. This was quantified by dichotomizing CA from age 41 in males and 29 in females. As shown in Table 2 in males in the older age category, the addictive status and their interaction were significant (P= 0.0010, 0.0021 and 0.0008 respectively), whilst in females the older age: addiction interaction was significant (P=0.0195, analysis restricted to women <55 years).

The regression changes are quantified for some of the major indices of arterial stiffness in cross-sectional data in Supplementary Table 6 (all P<0.005). Similar significantly different sex specific data were found in the longitudinal dataset when the RA was regressed against CA, time, sex and addictive status (most P<0.02, Supplementary Table 7).

When the mean sex-specific values for CA and BMI are inserted into the equation of Table 3 the mean vascular ages of the control groups amongst all patients, males and females was 35.42, 35.61 and 34.45 years, whilst the corresponding ages of the ODP's was 37.24, 36.31 and 39.18 years, representing incremental gains of 5.14%, 1.97% and 13.43%.

When quartiles are defined for lifetime heroin exposure the results shown in Figure 6 are found. Similar effects longitudinally are shown in Supplementary Figure 3. These results are quantitated in Supplementary Table 8 for both cross-sectional and longitudinal data compared to non-exposed controls. In the cross-sectional analysis the RA/CA ratio was regressed against the CA and Opioid exposure quartile. In the longitudinal analysis the RA/CA ratio was regressed against time, CA and the opioid exposure quartile. Whilst the CA: Level 3

For peer review only

opioid exposure interaction is of borderline significance in all patients and females (P=0.0546 and 0.0568, but not in males) in the longitudinal study the age: level 2 exposure is significant in all patients and males (P=0.0003 and 0.0110) and level 3 opioid exposure approaches significance in females both as a factor and in interaction with age (P=0.0501 and 0.0547). Supplementary Table 9 presents the results of a longitudinal regression of age-corrected indices of cardiovascular stiffness against time and opioid exposure quartile by gender, and notes a highly significant effect by exposure quartile in both genders.

The data was formally interrogated for a trend between the different quartiles of lifetime opioid exposure using the contrasts polynomial function in "R". Significant contrasts between the various quartiles for lifetime opioid exposure for the age: opioid exposure interaction was indeed demonstrated in all patients (est. = 0.1657, S.E. = 0.0787, t = 2.104, P = 0.0356; Model Adj. R^2 = 0.4265, F = 133.0, dF = 7, 1235, P < <0.0001), such that moving up a quartile level at age 60 increased the modelled RA by 8.42% to 65.051 years, and movement from the first (non-exposed) quartile to the highest (level 3) quartile increased the modelled RA 25.26% to 75.154 years.

Some environmental exposure – pathology relationships have been shown to be power functions of the duration of exposure and are therefore particularly sensitive to the periods for which exposure occurs [35]. Since opioid administration is often prescribed and recommended as being of indefinite duration this is particularly relevant to the present discussion. Therefore a detailed study of the power relationships of the duration of opioid administration was undertaken. Table 3 shows that simple regression models of RA/CA against the dose-duration interaction were significantly related to linear, quadratic, cubic and quartic terms in opioid exposure duration. Moreover when RA/CA was regressed against a model including interactive terms in linear, quadratic, cubic and quartic terms, all four were significant. When these models were compared by ANOVA studies the quadratic (AIC=82.888) was noted to be significantly better than the linear, cubic or quartic models (Anova tests, all P<0.05), but not significantly different from the combined model (AIC=83.731, P=0.87; Supplementary Table 10).

When a similar exercise was performed in the longitudinal dataset, similar results were obtained (Supplementary Table 11). In this study RA/CA was regressed against time and the opioid exposure quartile. As a combined linear-quadratic-cubic-quartic model failed to

For peer review only

converge, the highest order model used was linear-quadratic-quartic. Again quadratic and quartic terms in exposure duration persist in the final model. In this case the quadratic model (AIC=1040.85) was significantly superior to all the others but was not significantly better than the linear-quadratic-quartic model (AIC= 1041.09, P=0.082; Supplementary Table 12).

The data was interrogated for the presence of a dose-response relationship directly by examining continuous terms in opioid dose and duration regressed against the RA/CA ratio in both datasets. As shown in Table 4 the dose-duration interaction is significant in all patients considered together, in males and in females (P <0.0001, 0.0003 and 0.0236) in the cross-sectional dataset, whilst in the longitudinal dataset the parallel results are P = 0.0004, 0.0008 and 0.0127.

The final step of the analysis was to consider whether the significance of the opioid dose and duration might persist after adjustment for all known cardiovascular risk factors. In preliminary analyses lipidic factors were invariably eliminated during the model reduction process. Moreover, as discussed in the Introduction, opioids themselves are likely to perturb both lipid function, and also have a robust immunostimulatory action. Therefore including immune markers is likely to over-correct the model. Similarly when the time since the last cigarette consumption was included in the cross-sectional and longitudinal models it was eliminated during model reduction. For these reasons the final cross-sectional formula used regressed RA/CA against interactive terms in opioid dose and duration, height, tobacco consumption, systolic blood pressure and weight. The longitudinal model was similar but had an additive term in weight. These were the terms which remained significant in the models in preliminary exploratory analyses for log (RA). Further consideration of the clinical justification for the choice of this five way interaction is included in the Discussion section.

The results shown in Table 5 were found for the cross-sectional data. This table shows that significant interactions including opioid dose-duration terms are found in all patients (P=0.0261) and in males (P=0.0017; Adj. R^2=0.0815), whilst in females this interaction approaches significance (P=0.0516). When a similar exercise is performed on the longitudinal data, significant interactions are seen from P = 0.0011, 0.0013 and 0.0073 and above in each group respectively (Table 6).

For peer review only

Supplementary Table 13 presents the results of final models in which complete multiple regression was performed in the cross-sectional dataset for all pertinent variables. (Log) RA/CA was regressed against interactive terms in opioid dose and duration, height, tobacco use, systolic pressure and high sensitivity CRP together with additive terms in weight, cholesterol and HDL. Opioid dose and duration persist in the final model in males and in all patients, but not in females. Interestingly CRP features in most of the terms of these final models.

These overall results were broadly confirmed by the running of further analyses including by the use of by additivity and variance stabilization (AVAS) in R [36]. In particular we analysed a range of models including different polynomial terms in known predictors, and matching models augmenting the known risk factors with heroin use. Models with and without heroin use were compared. These analyses confirmed that the model fit for the relationship between log (RA/CA) and the predictive variables was significantly improved by the inclusion of terms for opiate exposure (dose and duration) amongst the male opiate dependents, in the opioid dependent groups in both sexes and in all patients; that the relationship between the various model parameters was (unsurprisingly) unlikely to be of a strictly linear form; and that superior model fits for log (RA/CA) were likely to be accompanied by various higher order interactions between the most powerful predictive independent variables namely (log) SP, weight, height, tobacco consumption and opioid exposure dose and duration.

For peer review only

Discussion

The major findings of this study are that indices of vascular age and arterial stiffness are worse in opioid dependence compare to opioid naïve controls with mean calculated ages elevated by 1.97% in males and 13.43% in females. The effect was thus more marked in females. A significant effect was found on both vascular age and augmentation index by exposure quartile after correction for CA and BMI. The RA/CA ratio was found to be related to power functions of the opioid duration of exposure in both cross-sectional and longitudinal analyses. A dose-response relationship was demonstrated with lifetime opioid exposure. In particular the effect of opioid exposure was robust, and remained after multiple adjustment in cross-sectional and longitudinal studies.

These findings should be interpreted in the light of the relatively modest degree of opioid exposure to which these patients were exposed. Whilst the dose and duration of opioids used by patients in this study is typical of that seen in many published series, the treatment of these patients was mostly with the relatively mild partial agonist buprenorphine, with only modest doses employed (mean 6.98$\pm$0.21mg): this dose level being much lower than the commonly recommended (18-24mg) [37]. Moreover buprenorphine has been shown to be more mild in its cardiovascular effects than the full agonist agent methadone . For this reason the results reported herein should be seen as a lower bound on the effects which may be observed.

The findings in relation to central vascular stiffness and age being related to power functions of the treatment duration is of great importance. Chronic opioid agonist treatment is often seen as relatively benign and partly for this reason is frequently recommended for very long term or indefinite administration [38-39]. However the demonstration here that the effects likely compound with time have major clinical practice implications, and have not been previously noted. The 1.97% effect described here for males, compounded over 30 years equates cumulatively to 179.5% gain and in diminution to 44.95% loss. Similarly the 13.43% gain seen in females compounded over 30 years is a 4349.02% gain and a 98.66% loss in reduction. The effects therefore of these process maintained over the long term on stem cell diminution or immune stimulation are potentially very considerable.

The exacerbation of the effect in females was clearly demonstrated in Figures 3-5, Tables 1 and 2, and Supplementary Tables 4 and 5 and Supplementary Figures 1 and 2. This finding

For peer review only

has, to the authors knowledge, only been reported previously by an Iranian group [14]. If one looks closely at Figure 5 several fascinating findings emerge. The vascular age algorithm used by the SphygmoCor software is based upon the augmentation index C_AGPH_HR75. This index has a clear curvilinear convex upward relationship to CA as shown in Figure 5 in the lower panels, unlike the C_AP_HR75 which is directly linear [40]. Hence the reduction at the higher ages on the C_AGPH_HR75 curve may be somewhat artefactual. If one looks at the upper panels of Figure 5 the rise in RA with age in ODP may be seen to be broadly exponential in form, if the decline in slope above age 45 years is discounted. In females it appears that the rise in RA occurs some 10 years prior to that seen in males, a finding emphasized by the middle panels. This fits with a dramatic clinical transformation which is seen in opioid dependent females at around age 30, and in males at about age 40. Whilst the cause of this is unknown it may possibly be related to a more aggressive immune system in females which is in part hormonally mediated [41]. In this context the demonstration that oestradiol directly stimulates telomerase was particularly provocative [42].

Some consideration of the comparability of the control and opioid dependent groups is appropriate. Significant differences were found between the control and opioid dependent groups in relation to chronological age, weight, BMI, smoking, cholesterol, LDL and hepatic and inflammatory laboratory markers, but these were controlled for by various the multiple regression techniques. The higher mean weight in the opioid dependent group may have been related in part to their higher age, and in part to the orexigenic effects of opioid agonism [43], whilst serial resource diversion may explain the slower rate of gain of weight in this group over time. Higher rates of tobacco consumption may be related to either the common features of the addictive lifestyle or the pro-consumptive effects of opioid analogues [44]. Significantly more of the females in the control group (11.4% vs. 5.41%) were aged over 50 years (P=0.029). However as no data was collected relating to hormonal status with regard to the menopause it is not possible to remark on the relative hormonal status of the two groups. Opioid dependency is well known to be associated with hypothalamic hypogonadotrophic oligoamenorrhoea, so that the age-related hormonal suppression in the control group was somewhat balanced by the drug related hormonal suppression in the opioid dependent group. Clarification of this issue must await further studies.

Some consideration is appropriate of the clinical justification for the five-way interaction which was presented in the final multiple regression models of RA (Tables 5 and 6). Height

For peer review only

is a key variable which relates to many techniques of measuring of arterial stiffness since it is related to the timing of arterial wave reflections, and it is hardly surprising that this remains in the final model [45-46]. Height and weight will naturally interact and indeed the BMI is one formula which formalizes such interactions. The opioid dose-duration exposure is a standard way in which to account for lifetime opioid exposure, and has been used by WHO [35] and NIH [14] investigators amongst others. Other factors including laboratory parameters were not present in the final models. Therefore it is not surprising that this cluster of factors remains in the final model.

On the other hand CRP was prominent and present in most terms in the final model when RA/CA was interrogated by multiple regression (Supplementary Table 13) suggesting that innate immune processes may be implicated in the observed opioid-vasculopathy effect. The age-related course of high sensitivity CRP has been described in several earlier reports in our patient cohort [6 28 47]. Supplementary Table 1 shows significant elevation of CRP, ESR, serum globulins, and circulating lymphocytes and monocyte numbers in the opioid dependent group. Consideration of the very flat age-dependent trajectories of these parameters in controls in our earlier reports shows that this effect is much greater in opioid dependence than might be expected on the basis of the higher chronologic age in the opioid dependent group alone. This is in turn consistent with the important chronic inflammatory basis of many vascular diseases [48-50] and the immune theory of organismal ageing [48].[51]

As noted above, vascular age is frequently said to be a surrogate for organismal ageing. The present data are consistent with that view, and also with the literature cited of accelerated ageing in these patients. The present technique provides a mechanism to quantify these changes at the whole human level. Interestingly, while it is well known that telomere length can function as a "mitotic clock" of the biological aging process, it has been shown that oxidative stress can stochastically increase this rate, in part by inducing single strand telomeric breaks related to the high guanosine content of telomeric repeats (TTAGGG)n [52-54]. The heightened inflammatory activity associated with opioid dependence has been previously noted [5-7 28 55], and the involvement of this pathway mechanistically unifies the inflammatory, oxidative, chromosomal, telomeric and stem cell hypotheses of aging, and accounts for the uniformity of the progeroid findings across organ systems [56] and the high prevalence of multisystem disease seen in opioid dependence [11-12 28].

For peer review only

As no structural imaging techniques were performed it is not possible to say with certainty if the observed elevations in vascular stiffness were associated with fixed atherosclerotic lesions. However a large and weighty literature confirms the relevance of significance of elevations in vascular stiffness [45-46 57-59]. Structural correlation must await further work.

The consistency of the present results with the previously reported data from various national and international groups [11-18 60] is clear in that we have confirmed the exacerbation of cardiovascular ageing and arterial stiffness in opioid dependency . However these earlier results have been extended by demonstrating a measurable major subclinical endophenotype of accelerated ageing antemortem, confirmed a dose-response relationship, demonstrated for the first time the significance of the improved model quadratic in opioid exposure duration, and shown that the opioid-related effect is robust to adjustment for known cardiovascular risk factors in both sexes. Moreover we confirmed that the effect is worse in females [14], and extended this result by showing that the differential effect commences in women over a decade earlier than in men (29 vs. 41 years). In that the PWA technique directly relates increased arterial stiffness to cardiovascular and therefore organismal ageing the present detailed studies link advanced cardiovascular age to many other indices of accelerated ageing in opioid dependency as have been shown by this [6-7 10 26 28 47 61-65] and other [66] groups. As such the present report is a meaningful and important extension of earlier reports from this project on the cardiovascular implications of opioid dependency [30-33], its treatment [19] and withdrawal [23] and the antagonist-induced reversal of these effects [20]. Hence the phenomenology of the present descriptive report is consistent with, and extends, extant reports in the published literature.

Whilst this study is not designed to be mechanistic in nature some brief comment on this issue may be appropriate, in part for its relevance to the consideration of possibly aetiologically causal pathways. Some of the most interesting questions to emerge from the present work relate to the possible mechanisms underlying the plethora of changes which have been identified in opioid dependence as cardiovascular risk factors, as potentially atherogenic processes and as accelerants of ageing processes. Opioids have been linked with blockade of the cell cycle at cyclin dependent kinases 2, 4 and 6 by $P16^{INK4A}$ and $P21^{WAF1/CIP1}$ [67-68]. P16 is the gene product of CDKN2A located at the senescence locus on Chromosome

For peer review only - http://bmjopen.bmj.com/site/about/guidelines.xhtml

9p21.3 which is a hotspot for many GWAS studies, and from which three senescence related proteins and one senescence associated long non-coding RNA are transcribed [69]. Furthermore opioid agonists have been shown to directly ligate the myeloid differentiation factor 2 (MD2)- toll-like receptor (TLR) 4 heterodimer leading to the downstream activation of the numerous powerful pathways linked to stimulation of the endotoxin receptor [70]. Indeed systemic endotoxaemia was recently shown to modulate the expression of 6,025 genes [71]. Some of the pathways activated downstream by TLR4 signalling include the cyclooxygenase, lipooxygenase, MAP kinase (JNK and P38), AP-1, inducible nitric oxide synthase (iNOS), phosphoinositol-3 kinase (PI3K), and TGFβ pathways [72], many of which have been noted to be stimulated within the first few minutes of TLR4 ligation [73], to be stimulated by opioids [70 72], and to be associated with potentiating the effects of ageing [51 74]. Many of these immune products directly exacerbate the stem cell blockade [75]. This then implies that opioid dependency may be conceptualized as chronically, if largely subclinically, ill.

It is very exciting that new research has identified that these processes also act particularly in proopioimelanocortin (POMC) neurons of the paraventricular and arcuate nuclei of the mediobasal hypothalamus which are key determinants of the body's metabolic homeostasis, glucostatic and lipostatic functions, energy balance, appetitive drives, sympathoadrenal activation state, and even lifespan modulation in some organisms [76-78], and that derangements in this region cause metabolic syndrome and its numerous related disorders in model organisms including mammals and likely also humans [79]. These workers have shown that activation of the MD2/TLR4/IKKβ/NF-κB cascade induces endoplasmic reticulum, mitochondrial and autophagic stress, which along with reduced neurogenesis, increased POMC neuronal apoptosis [77], and glial activation and reactive gliosis [79-80] underlie these numerous disorders and derangements. Whilst this research focuses on TLR4 ligation of unsaturated fatty acids and oxidized low density lipoproteins, opioids have been shown to act similarly as xenobiotics through MD2-TLR4 [81], and opioid agonists have been shown to produce POMC cell hyperpolarization and relative refractoriness [44], whilst opioid antagonists have the reverse effect [82]. Small molecule TLR4 inhibitors are under development for several therapeutic applications [83-84]. The confluence then of these two important lines of recent research has the potential to produce major conceptual and therapeutic advances in this field. A clear understanding of direct links from xenobiotic-TLR4-NF-κB signalling to

For peer review only

sympathoadrenal tone and hypothalamic lifespan control has the potential to revolutionize the direction of therapeutic endeavours in the treatment of opioid dependence.

It is fascinating that the promoters for two of the key stem cell genes, Oct4 and Nanog have response elements in their promoters for STAT-1 and -3 which are commonly produced as a result of cytokine signalling, particularly by gp-130 cytokines such as IL-6 and leukaemia inhibitory factor (LIF) [85-86]. Furthermore there is three way cross-talk between stem cell and metabolic –immune genes including STAT-3 and PPARγ [87]. Other cytokines, such as IL-1 have been noted in opioid dependency [8] and are usually indicative of inflammasome activation, and fractalkine and MCP-1/CCL2 have also been found to be elevated and are relevant both to neuropathological and atherogenic processes [70]. As TLR4 shares many ligands and co-chaperones with the receptor for advanced glycation end (RAGE) products this auto-amplificatory system may also be involved [88-91].

This study had various strengths and limitations. The application of sophisticated cardiovascular measurement technology to the problem of drug addiction is unique to this group. Extended follow-up and recruitment of opioid dependent patients is also notoriously difficult. The statistical techniques employed herein are advanced and robust. Design strengths include its complimentary cross-sectional and longitudinal design. Limitations of the present work relate to difficulties quantitating frequently variable amounts of past drug use in the absence of a formal measurement instrument. Use of a formal instrument such as the addiction severity index, and spot tests for drugs and alcohol are indicated in future replications of this study. Employment of structural tests, and mechanistic studies to investigate pathophysiological pathways, would be advisable in future iterations of this work.

It is important to remember that the models and results described in this work are not represented as reliable and generalizable calibrations of the cardiovascular effects of opioid exposure, in combination with other well established risk factors. These models are the result of an empirical investigation of many correlated risk factors from a particular clinical cohort. It is most unlikely that similar model parameter estimates would be reproduced in a study of a different clinical cohort. The results serve a much more limited objective: is there evidence of an opioid effect cardiovascular health, taking account of other known risk factors. We suggest that there is evidence for such an effect; though we must accept that this evidence is in part contingent of the adequacy of the model for these known risk factors. This is a general problem facing all studies of correlated risk factors, in the absence of a well

For peer review only

established mechanistic model. We would consider the impact of opioids to be established only if similar results were obtained in analysis of data from an independent case series. In this context the confirmatory nature of the present data to essentially identical results from the cross-sectional (only) data from Iran amongst males [16-17], and the various other findings noted in the literature review in the Introductory section of the present report are particularly pertinent.

In summary the present study documents elevated vascular stiffness and age associated with opioid use which is robust to multiple adjustment to other known risk factors. A strong dose-response effect was noted, and the duration of treatment administration was noted to be particularly important, being related to elevations in vascular age of the first second, third and fourth order. This finding indicates limitation of treatment duration wherever clinically possible. The effect was also worse in females. Overall this study raises many further mechanistic questions which indicate further study on this complex but important model condition.

For peer review only

Acknowledgements

The authors would like to thank Dr Mervyn Thomas of Emphron for assistance with the statistics and graphical design.

Conflict of Interest

Nil declared.

Sources of Funding

Nil.

Statement of Authorship

The study was designed, the data collected, the analyses performed, the literature reviewed and the first draft written by ASR. GKH provided important intellectual input, assisted with study design, and revised and reviewed the manuscript. Both authors confirm the accuracy and integrity of the work and data collection and analysis.

Data sharing

No additional data available.

For peer review only

References

1. Rosen D, Smith ML, Reynolds CF, 3rd. The prevalence of mental and physical health disorders among older methadone patients. *Am J Geriatr Psychiatry* 2008;16(6):488-97.
2. Cooper OB, Brown TT, Dobs AS. Opiate drug use: a potential contributor to the endocrine and metabolic complications in human immunodeficiency virus disease. *Clin Infect Dis* 2003;37 Suppl 2:S132-6.
3. Ceriello A, Quatraro A, Giugliano D. Opiate addict as diabetic patient? *Diabetes Care* 1988;11(5):443.
4. Kolarzyk E, Pach D, Wojtowicz B, et al. Nutritional status of the opiate dependent persons after 4 years of methadone maintenance treatment. *Przeglad lekarski* 2005;62(6):373-7.
5. Brunton LL, Lazo JS, Parker KL, editors. *Goodman and Gilman's the Pharmacologic Basis of Therapeutics*. Eleventh Edition ed. New York: McGraw Hill, 2006.
6. Reece A. S. Relative and Age Dependent Stimulation of Soluble and Cellular Immunity in Opiate Dependence. *Journal of Addiction Medicine* 2012;6(1):10-17.
7. Reece A. S. High Sensitivity CRP in Opiate Addiction: Relative and Age-Dependent Elevations. *Cardiovascular Toxicology* 2012;12(2):149-57.
8. Neri S, Bruno CM, Pulvirenti D, et al. Randomized clinical trial to compare the effects of methadone and buprenorphine on the immune system in drug abusers. *Psychopharmacology (Berl)* 2005;179(3):700-4.
9. Reece A. S. Absolute and Age Dependent Elevations of Serum Calcium and Phosphate and their Products in Clinical Opiate Dependence. *J. Subs. Abuse* 2013;Accepted 27th November 2012.
10. Reece AS, Davidson P. Deficit of circulating stem--progenitor cells in opiate addiction: a pilot study. *Substance abuse treatment, prevention, and policy* 2007;2:19-28.
11. Darke S, Duflou J, Torok M. The comparative toxicology and major organ pathology of fatal methadone and heroin toxicity cases. *Drug Alcohol Depend* 2010;106(1):1-6.
12. Darke S, Kaye S, Duflou J. Systemic disease among cases of fatal opioid toxicity. *Addiction* 2006;101(9):1299-305.
13. Degenhardt L., Randall D., Hall W., et al. Mortality among clients of a state-wide opioid pharmacotherapy program over 20 years: Risk factors and lives saved. *Drug Alcohol Depend* 2009;105(1-2):9-15.
14. Khademi H, Malekzadeh R, Pourshams A, Jafari E, Salahi R, Semnani S, et al. Opium use and mortality in Golestan Cohort Study: prospective cohort study of 50,000 adults in Iran. *BMJ (Clinical research ed* 2012;344:e2502.
15. Sadeghian S, Darvish S, Davoodi G, et al. The association of opium with coronary artery disease. *Eur J Cardiovasc Prev Rehabil* 2007;14(5):715-7.
16. Sadeghian S, Dowlatshahi S, Karimi A, et al. Epidemiology of opium use in 4398 patients admitted for coronary artery bypass graft in Tehran Heart Center. *J Cardiovasc Surg (Torino)* 2011;52(1):140-1.
17. Sadeghian S, Graili P, Salarifar M, et al. Opium consumption in men and diabetes mellitus in women are the most important risk factors of premature coronary artery disease in Iran. *International journal of cardiology* 2010;141(1):116-8.
18. Smyth B, Hoffman V, Fan J, et al. Years of potential life lost among heroin addicts 33 years after treatment. *Preventive medicine* 2007;44(4):369-74.
19. Reece A.S., Hulse G.K. Impact of Opioid Pharmacotherapy on Arterial stiffness and Vascular Ageing: Cross-sectional and Longitudinal Studies. *Cardiovascular Toxicology* 2013;13(3):254-66.
20. Reece A. S., Hulse G.K. Reduction in Arterial Stiffness and Vascular Age by Naltrexone-Induced Interruption of Opiate Agonism. *British Medical Journal - Open* 2013.

For peer review only

21. Hosseini SK, Masoudkabir F, Vasheghani-Farahani A, et al. Opium consumption and coronary atherosclerosis in diabetic patients: a propensity score-matched study. *Planta Med* 2011;77(17):1870-5.
22. Charney DS, Redmond DE, Jr., Galloway MP, et al. Naltrexone precipitated opiate withdrawal in methadone addicted human subjects: evidence for noradrenergic hyperactivity. *Life Sci* 1984;35(12):1263-72.
23. Reece AS, Hulse GK. Elevation of Central Arterial Stiffness and Vascular Ageing in Opiate Withdrawal: Cross-sectional and Longitudinal Studies. *Cardiovasc Toxicol* 2012;13(1):55-67.
24. Chien KR, Karsenty G. Longevity and lineages: toward the integrative biology of degenerative diseases in heart, muscle, and bone. *Cell* 2005;120(4):533-44.
25. Lakatta EG, Wang M, Najjar SS. Arterial aging and subclinical arterial disease are fundamentally intertwined at macroscopic and molecular levels. *Med Clin North Am* 2009;93(3):583-604, Table of Contents.
26. Reece AS. Hair graying in substance addiction. *Archives of dermatology* 2007;143(1):116-8.
27. Kim TW, Alford DP, Malabanan A, et al. Low bone density in patients receiving methadone maintenance treatment. *Drug Alcohol Depend* 2006;85(3):258-62.
28. Reece AS. Evidence of Accelerated Ageing in Clinical Drug Addiction from Immune, Hepatic and Metabolic Biomarkers. *Immun Ageing* 2007;4(1):6-15.
29. Randall D, Degenhardt L, Vajdic CM, et al. Increasing cancer mortality among opioid-dependent persons in Australia: a new public health challenge for a disadvantaged population. *Australian and New Zealand journal of public health* 2011;35(3):220-5.
30. Reece A.S., Hulse G.K. Lifetime Opiate Exposure as an Independent and Interactive Cardiovascular Risk Factor in Males: A Cross-Sectional Clinical Study. *Vascular Health and Risk Management* 2013;9(55):551-59.
31. Reece A.S., Hulse G.K. Opiate Exposure Increases Arterial Stiffness, Advances Vascular Age and is an Independent Cardiovascular Risk Factor in Females: A Cross-Sectional Clinical Study. *World J. Cardiovascular Diseases* 2013;In Press(DOI: 10.4236/wjcd.2013.35056).
32. Reece A. S., Hulse G.K. Duration of Opiate Exposure as a Determinant of Arterial Stiffness and Vascular Age in Male Opiate Dependence: A Longitudinal Study. *Journal of Clinical Pharmacy and Therapeutics* 2013;39(2):158-67.
33. Reece A. S., Hulse G.K. Opiate Dependence as an Independent and Interactive Risk Factor for Arterial Stiffness and Cardiovascular Ageing - A Longitudinal Study in Females. *J Clinical Medical Research* 2013;5(5):356-67.
34. Reece AS. Psychosocial and treatment correlates of opiate free success in a clinical review of a naltrexone implant program. *Subst Abuse Treat Prev Policy* 2007;2:35-49.
35. Breslow N.E., Day N.E. *Statistical Methods in Cancer Research Volume II The Design and Analysis of Cohort Studies*. 1 ed. Lyon: International Agency for Cancer Research,, 1987.
36. Tibshirani R. Estimating transformations for regression via additivity and variance stabilization. *Journal of the American Statistical Association* 1988;83(402):394-405.
37. Kacinko SL, Jones HE, Johnson RE, et al. Correlations of maternal buprenorphine dose, buprenorphine, and metabolite concentrations in meconium with neonatal outcomes. *Clinical pharmacology and therapeutics* 2008;84(5):604-12.
38. Hulse G., White J., Cape G., editors. *Management of Drug and Alcohol Problems*. Sydney: Oxford University Press, 2002.
39. Oviedo-Joekes E, Brissette S, Marsh DC, et al. Diacetylmorphine versus methadone for the treatment of opioid addiction. *N Engl J Med* 2009;361(8):777-86.
40. SphygmoCor, editor. *Software Operator's Guide, Pulse Wave Analysis System, SCOR-Px*. Sydney: AtCor Medical, 2006.
41. Klein SL. Sex influences immune responses to viruses, and efficacy of prophylaxis and treatments for viral diseases. *Bioessays* 2012;34(12):1050-9.
42. Sato R, Maesawa C, Fujisawa K, ured cells and carbon tetrachloride induced rat liver fibrosis. *Gut* 2004;53(7):1001-9.
43. Reece AS. Hypothalamic opioid-melanocortin appetitive balance and addictive craving. *Medical hypotheses* 2011;76(1):132-7.

For peer review only

44. Cowley MA, Smart JL, Rubinstein M, et al. Leptin activates anorexigenic POMC neurons through a neural network in the arcuate nucleus. *Nature* 2001;411(6836):480-4.
45. Nichols W.W., O'Rourke M.F., editors. *McDonald's Blood Flow in Arteries: Theoretical, Experimental and Clinical Principals*. London: Hodder Arnold, 2005.
46. O'Rourke MF, Pauca AL. Augmentation of the aortic and central arterial pressure waveform. *Blood Press Monit* 2004;9(4):179-85.
47. Reece AS. High-sensitivity CRP in opiate addiction: relative and age-dependent elevations. *Cardiovasc Toxicol* 2012;12(2):149-57.
48. Fauci AS, Braunwald E, Kapser DL, et al. *Harrison's Principles of Internal Medicine, 17th Edition*. 17th Edition ed. New York: McGraw Hill, 2008.
49. Lakatta EG. Arterial aging is risky. *J Appl Physiol* 2008;105(4):1321-2.
50. Le Couteur DG, Lakatta EG. A vascular theory of aging. *J Gerontol A Biol Sci Med Sci* 2010;65(10):1025-7.
51. Hadley EC, Lakatta EG, Morrison-Bogorad M, et al. The future of aging therapies. *Cell* 2005;120(4):557-67.
52. Furumoto K, Inoue E, Nagao N, et al. Age-dependent telomere shortening is slowed down by enrichment of intracellular vitamin C via suppression of oxidative stress. *Life Sci* 1998;63(11):935-48.
53. von Zglinicki T. Oxidative stress shortens telomeres. *Trends Biochem Sci* 2002;27(7):339-44.
54. von Zglinicki T, Pilger R, Sitte N. Accumulation of single-strand breaks is the major cause of telomere shortening in human fibroblasts. *Free Radic Biol Med* 2000;28(1):64-74.
55. Reece A. S. Chronic Immune Stimulation as a Contributing Cause of Chronic Disease in Opiate Addiction Including Multi-System Ageing *Medical hypotheses* 2010;In Press.
56. Reece AS. Differing age related trajectories of dysfunction in several organ systems in opiate dependence. *Aging Clin Exp Res* 2011;24(1):85-96.
57. Weber T, Auer J, O'Rourke MF, et al. Arterial stiffness, wave reflections, and the risk of coronary artery disease. *Circulation* 2004;109(2):184-9.
58. Williams B, Lacy PS, Thom SM, et al. Differential impact of blood pressure-lowering drugs on central aortic pressure and clinical outcomes: principal results of the Conduit Artery Function Evaluation (CAFE) study. *Circulation* 2006;113(9):1213-25.
59. Nichols WW. Clinical measurement of arterial stiffness obtained from noninvasive pressure waveforms. *Am J Hypertens* 2005;18(1 Pt 2):3S-10S.
60. Degenhardt L, Larney S, Randall D, et al. Causes of death in a cohort treated for opioid dependence between 1985 and 2005. *Addiction* 2013.
61. Reece A. S. Manifold Implications of Forgotten Hyperglycaemia in Clinical Opiate Dependence. *Drug and Chemical Toxicology* 2012.
62. Reece AS. Relative and Age-dependent Stimulation of Soluble and Cellular Immunity in Opiate Dependence. *J Addict Med* 2012;6(1):10-7.
63. Reece AS. Differing age related trajectories of dysfunction in several organ systems in opiate dependence. *Aging Clin Exp Res* 2012;24(1):85-96.
64. Reece AS. Dentition of addiction in Queensland: poor dental status and major contributing drugs. *Australian dental journal* 2007;52(2):144-9.
65. Reece A. Lifetime prevalence of cervical neoplasia in addicted and medical patients. *Aust N Z J Obstet Gynaecol* 2007;47(5):419-23.
66. Tatia M.C. Lee, Gordon L.F. Cheng, Hung Zeng, et al. Heroin abuse accelerates biological aging: A novel insight from telomerase and brain imaging interaction. *Translational Psychiatry* 2013.
67. Cheng F, McLaughlin PJ, Verderame MF, et al.The OGF-OGFr axis utilizes the p16INK4a and p21WAF1/CIP1 pathways to restrict normal cell proliferation. *Molecular biology of the cell* 2009;20(1):319-27.
68. Zagon IS, Verderame MF, McLaughlin PJ. The biology of the opioid growth factor receptor (OGFr). *Brain research* 2002;38(3):351-76.
69. Pasmant E, Sabbagh A, Vidaud M, et al. ANRIL, a long, noncoding RNA, is an unexpected major hotspot in GWAS. *FASEB J* 2010;25(2):444-48.

For peer review only

70. Hutchinson MR, Shavit Y, Grace PM, et al. Exploring the neuroimmunopharmacology of opioids: an integrative review of mechanisms of central immune signaling and their implications for opioid analgesia. *Pharmacological reviews* 2011;63(3):772-810.
71. Starr ME, Hu Y, Stromberg AJ, et al. Gene expression profile of mouse white adipose tissue during inflammatory stress: age-dependent upregulation of major pro-coagulant factors. *Aging Cell* 2012:n/a-n/a.
72. Coller JK, Hutchinson MR. Implications of central immune signaling caused by drugs of abuse: mechanisms, mediators and new therapeutic approaches for prediction and treatment of drug dependence. *Pharmacol Ther* 2012;134(2):219-45.
73. Grzanna R., Brown R.M. NIDA Research Monograph 125: Activation of Intermediate Early Genes by Drugs of Abuse. In: National Institutes of Drug Abuse, editor. Bethesda, Maryland: NIH, 1993:1-220.
74. Kirkwood TB. Understanding the odd science of aging. *Cell* 2005;120(4):437-47.
75. Hoing S, Rudhard Y, Reinhardt P, et al. Discovery of inhibitors of microglial neurotoxicity acting through multiple mechanisms using a stem-cell-based phenotypic assay. *Cell Stem Cell* 2012;11(5):620-32.
76. Zhang G, Li J, Purkayastha S, et al. Hypothalamic programming of systemic ageing involving IKK-[bgr], NF-[kgr]B and GnRH. *Nature* 2013;497(7448):211-16.
77. Li J, Tang Y, Cai D. IKKbeta/NF-kappaB disrupts adult hypothalamic neural stem cells to mediate a neurodegenerative mechanism of dietary obesity and pre-diabetes. *Nat Cell Biol* 2012;14(10):999-1012.
78. Purkayastha S, Zhang G, Cai D. Uncoupling the mechanisms of obesity and hypertension by targeting hypothalamic IKK-beta and NF-kappaB. *Nat Med* 2011;17(7):883-7.
79. Thaler JP, Yi CX, Schur EA, et al. Obesity is associated with hypothalamic injury in rodents and humans. *J Clin Invest* 2012;122(1):153-62.
80. Czapski GA, Gajkowska B, Strosznajder JB. Systemic administration of lipopolysaccharide induces molecular and morphological alterations in the hippocampus. *Brain Res* 2010;1356:85-94.
81. Wang X, Loram LC, Ramos K, et al. Morphine activates neuroinflammation in a manner parallel to endotoxin. *Proc Natl Acad Sci U S A* 2012;109(16):6325-30.
82. Billes SK, Greenway FL. Combination therapy with naltrexone and bupropion for obesity. *Expert opinion on pharmacotherapy* 2011;12(11):1813-26.
83. Neal MD, Jia H, Eyer B, et al. Discovery and validation of a new class of small molecule Toll-like receptor 4 (TLR4) inhibitors. *PLoS ONE* 2013;8(6):e65779.
84. Theberge FR, Li X, Kambhampati S, et al. Effect of chronic delivery of the Toll-like receptor 4 antagonist (+)-naltrexone on incubation of heroin craving. *Biol Psychiatry* 2013;73(8):729-37.
85. Kim CG, Chung IY, Lim Y, et al. A Tcf/Lef element within the enhancer region of the human NANOG gene plays a role in promoter activation. *Biochem Biophys Res Commun* 2011;410(3):637-42.
86. Yang HM, Do HJ, Oh JH, et al. Characterization of putative cis-regulatory elements that control the transcriptional activity of the human Oct4 promoter. *J Cell Biochem* 2005;96(4):821-30.
87. Folmes CD, Dzeja PP, Nelson TJ, et al. Metabolic plasticity in stem cell homeostasis and differentiation. *Cell Stem Cell* 2012;11(5):596-606.
88. Zurolo E, Iyer A, Maroso M, et al. Activation of Toll-like receptor, RAGE and HMGB1 signalling in malformations of cortical development. *Brain* 2011;134(Pt 4):1015-32.
89. Nogueira-Machado JA, Volpe CM, Veloso CA, et al. HMGB1, TLR and RAGE: a functional tripod that leads to diabetic inflammation. *Expert opinion on therapeutic targets* 2011;15(8):1023-35.
90. Sakaguchi M, Murata H, Yamamoto K, et al. TIRAP, an adaptor protein for TLR2/4, transduces a signal from RAGE phosphorylated upon ligand binding. *PLoS ONE* 2011;6(8):e23132.
91. Veloso CA, Fernandes JS, Volpe CM, et al. TLR4 and RAGE: similar routes leading to inflammation in type 2 diabetic patients. *Diabetes Metab* 2011;37(4):336-42.

For peer review only

For peer review only

Figure Captions:

Figure 1: Ageing and Stiffness Indices by Chronologic Age by Opioid Dependency Status
RA – Vascular Reference Age, CA – Chronologic Age, C_AP_HR75 – central augmentation pressure at Heart Rate 75 bpm, C_AGPH_HR75 – Central Augmentation Pressure / Pulse Height Ratio at Heart Rate 75 bpm.

Figure 2: Corrected Ageing and Stiffness Indices by Time by Opioid Dependency Status
RA – Vascular Reference Age, CA – Chronologic Age, C_AP_HR75 – central augmentation pressure at Heart Rate 75 bpm, C_AGPH_HR75 – Central Augmentation Pressure / Pulse Height Ratio at Heart Rate 75 bpm.

Figure 3: BMI-Related Ageing Indices by Chronologic Age by Addictive Status by Sex
RA – Vascular Reference Age, CA – Chronologic Age, C_AP_HR75 – central augmentation pressure at Heart Rate 75 bpm, C_AGPH_HR75 – Central Augmentation Pressure / Pulse Height Ratio at Heart Rate 75 bpm.

Figure 4: BMI-Related Ageing Indices by Chronologic Age by Sex by Addictive Status
RA – Vascular Reference Age, CA – Chronologic Age, C_AP_HR75 – central augmentation pressure at Heart Rate 75 bpm, C_AGPH_HR75 – Central Augmentation Pressure / Pulse Height Ratio at Heart Rate 75 bpm.

Figure 5: Ageing Indices by Chronologic Age by Sex by Addictive Status – Loess Curves
RA – Vascular Reference Age, CA – Chronologic Age.

Figure 6: Vascular Age Indices by Chronologic Age by Opioid Dependency Quartile
RA – Vascular Reference Age, CA – Chronologic Age. Opioid Exposure Quartiles as Defined in Methods section.

Supplementary Figure Captions:

Suppl. Figure 1: Ageing Indices by Chronologic Age by Addictive Status by Sex
RA – Vascular Reference Age, CA – Chronologic Age, C_AP_HR75 – central augmentation pressure at Heart Rate 75 bpm, C_AGPH_HR75 – Central Augmentation Pressure / Pulse Height Ratio at Heart Rate 75 bpm.

Suppl. Figure 2: Ageing Indices by Chronologic Age by Sex by Addictive Status
RA – Vascular Reference Age, CA – Chronologic Age.

For peer review only

Suppl. Fig. 3: Vascular Age Indices by Time by Opioid Dependency Level
RA – Vascular Reference Age, CA – Chronologic Age.

For peer review only

TABLES

For peer review only

For peer review only

TABLES

For peer review only

Table 1.: Effects of Gender on Key Measures of Arterial Stiffness - Cross-Sectional Dataset

Dependent Variable	Group	Parameter	Adj. R^2	F	dF	Model P	Estimate	Upper Limit	Lower Limit	P
RA-CA Difference	Opioid	Chron.Age :Female.Sex	0.0189	7.596	2, 684	0.0005	0.6324	1.3123	-0.0475	0.0688
BMI	Control	Chron.Age :Female.Sex	0.1523	52.58	2, 572	<0.0001	-0.0092	-0.0014	-0.0170	0.0213
BMI	Opioid	Female.Sex	0.0713	27.35	2, 684	<0.0001	-0.0827	-0.0555	-0.1099	<0.0001
RA/BMI	Opioid	Chron.Age :Female.Sex	0.2617	122.6	2, 684	<0.0001	0.0361	0.0539	0.0183	<0.0001
C_AP_HR75/BMI	Control	Chron.Age :Female.Sex	0.4882	274.3	2, 571	<0.0001	0.0312	0.0390	0.0234	<0.0001
C_AP_HR75/BMI	Control	Chron.Age :Female.Sex	0.4882	274.3	2, 571	<0.0001	0.0312	0.0390	0.0234	<0.0001
C_AP_HR75/BMI	Opioid	Chron.Age :Female.Sex	0.3712	203.4	2, 684	<0.0001	0.0467	0.0543	0.0391	<0.0001
C_AGPH_HR75/BMI	Control	Chron.Age :Female.Sex	0.4603	245.4	2, 571	<0.0001	0.0924	0.1144	0.0704	<0.0001
C_AGPH_HR75/BMI	Opioid	Chron.Age :Female.Sex	0.3980	227.8	2, 684	<0.0001	0.1265	0.1451	0.1079	<0.0001
C_AP_HR75/(CA*BMI)	Control	Female.Sex	0.2809	112.9	2, 571	<0.0001	0.0033	0.0043	0.0023	<0.0001
C_AP_HR75/(CA*BMI)	Opioid	Female.Sex	0.2489	114.7	2, 684	<0.0001	0.0047	0.0055	0.0039	<0.0001
C_AGPH_HR75/(CA*BMI)	Control	Female.Sex	0.2567	99.96	2, 571	<0.0001	0.0096	0.0125	0.0067	<0.0001
C_AGPH_HR75/(CA*BMI)	Opioid	Female.Sex	0.2604	121.8	2, 684	<0.0001	0.0130	0.0152	0.0108	<0.0001

For peer review only

Table 2.:

Age: Addictive Status Relationships with CA Discretized

About 41 Years in Males and 29 Years in Females

With Females <55 Years of Age

Parameter	Variable Statistics				Model Summaries		
	Estimate	Upper Limit	Lower Limit	P	F	dF	P
Males							
Older Age	-0.1837	-0.0745	-0.2929	0.0010	5.492	3, 862	0.0010
Opioids	-0.0846	-0.0307	-0.1385	0.0021			
Older Age: Opioids	0.2310	0.3657	0.0963	0.0008			
Females							
Older Age: Opioids	0.1248	0.2291	0.0205	0.0195	2.169	3, 378	0.0912

For peer review only

Table 3.: Cross-sectional Models Polynomial in Heroin Duration

	Parameter				Model		
**Model / Parameter**	**Value**	**Upper Limit**	**Lower Limit**	**P-value**	**F**	**dF**	**P**
1 Order- Linear							
H.Dura.n	-0.0059	-0.0018	-0.0100	0.0056	8.628	2, 664	0.0002
H.Dose: H.Dura.n	0.0091	0.0134	0.0048	3.7.E-05			
2 Order- Quadratic							
H.Dura.n	-0.0171	-0.0063	-0.0279	0.0018	7.443	3, 663	<0.0001
H.Dura.n^2	0.0004	0.0008	0.0000	0.0261			
H.Dose: H.Dura.n	0.0084	0.0127	0.0041	0.0002			
3 Order - Cubic							
H.Dura.n	-0.0116	-0.0047	-0.0185	0.0012	7.102	3 663	0.0001
H.Dura.n^3	6.3E-06	0.0000	0.0000	0.0466			
H.Dose: H.Dura.n	0.0084	0.0127	0.0041	0.0001			
4 Order- Quartic							
H.Dura.n	-0.0059	-0.0018	-0.0100	0.0056	8.628	2, 664	0.0002
H.Dose: H.Dura.n	0.0091	0.0134	0.0048	3.7.E-05			
1, 2, 3 & 4 Order							
H.Dose: H.Dura.n^3	6.5E-05	1.0E-04	2.6E-05	0.0012	3.353	7, 659	0.0016
H.Dura.n^2 : H.Dura.n^3	-1.0E-06	-3.9E-07	-1.6E-06	0.0007			
H.Dura.n^2 : H.Dura.n^4	9.2E-08	1.5E-07	3.5E-08	0.0014			
H.Dura.n^3: H.Dura.n^4	-2.7E-09	-9.8E-10	-4.4E-09	0.0026			
H.Dose: H.Dura.n^2 : H.Dura.n^4	-4.5E-09	-9.7E-10	-8.0E-09	0.0145			
H.Dura.n: H.Dura.n^3: H.Dura.n^4	2.5E-11	4.3E-11	7.4E-12	0.0048			
H.Dose: H.Dura.n: H.Dura.n^3: H.Dura.n^4	2.6E-12	5.0E-12	2.5E-13	0.0312			

For peer review only

Table 4: Dose-Response Relationships

**Cross-Sectional Studies**							
**Parameter**	**Variable Statistics**				**Model Summaries**		
	**Estimate**	**Upper Limit**	**Lower Limit**	**Pr(>|t|)**	**F**	**dF**	**P**
Both Sexes							
H.Dura.n	-0.0059	-0.0018	-0.0100	0.0056	8.628	2, 664	0.0002
H.Dose: H.Dura.n	0.0091	0.0134	0.0048	0.0000			
Males							
H.Dura.n	-0.0089	-0.0034	-0.0144	0.0018	7.105	2, 441	0.0009
H.Dose: H.Dura.n	0.0124	0.0191	0.0057	0.0003			
Females							
H.Dose: H.Dura.n	0.0052	0.0097	0.0007	0.0236	5.201	1, 210	0.0236

**Longitudinal Studies** -							
**Group**	**Variable Statistics**					**Model**	
**Parameter**	**Value**	**Upper Limit**	**Lower Limit**	**t-value**	**P-value**	**AIC***	**LogLik***
Both Sexes (DF=814)							
H.Dose:H.Dura.n	0.0045	0.0070	0.0020	3.5512	0.0004	1068.74	-530.37
Males (DF=552)							
H.Dura.n	-0.0036	-0.0005	-0.0067	-2.2069	0.0277	753.15	-371.58
H.Dose:H.Dura.n	0.0075	0.0118	0.0032	3.3754	0.0008		
Females (DF=260)							
H.Dose:H.Dura.n	0.0052	0.0093	0.0011	2.5091	0.0127	347.43	-169.72

For peer review only

Table 5.: Cross-Sectional Adjusted Regression Models

Group	Variable Statistics				Model		
Parameter	Estimate	Upper Limit	Lower Limit	Pr(>|t|)	F	dF	P
Both Sexes							
H.Dura.n: Cigs.d: SP : Weight	0.00023	0.0003	0.0001	0.0001	7.561	9, 657	<0.0001
H.Dura.n: Height: Cigs.d: SP	0.00030	0.0005	0.0001	0.0047			
H.Dura.n: Height: Cigs.d	-0.00143	-0.0004	-0.0024	0.0048			
H.Dura.n: Height: Cigs.d: SP : Weight	-0.00007	0.0000	-0.0001	0.0051			
H.Dura.n: Height: Cigs.d: Weight	0.00032	0.0006	0.0001	0.0063			
H.Dose: H.Dura.n: Height: Cigs.d: Weight	-0.00037	-0.0001	-0.0007	0.0261			
H.Dose: H.Dura.n: Height: Cigs.d: SP : Weight	0.00008	0.0001	0.0000	0.0262			
H.Dose: H.Dura.n: Height: Cigs.d	0.00155	0.0029	0.0002	0.0286			
H.Dose: H.Dura.n: Height: Cigs.d: SP	-0.00032	0.0000	-0.0006	0.0290			
Males							
H.Dura.n: Weight	-0.0024	-0.0010	-0.0038	0.0007	5.485	10, 438	<0.0001
H.Dose: H.Dura.n: Weight	0.0032	0.0052	0.0012	0.0017			
H.Dose: Cigs.d: SP : Weight	-4.4770	-1.0529	-7.9011	0.0107			
H.Dose: Height: Cigs.d: SP : Weight	0.0253	0.0447	0.0059	0.0109			
H.Dose: Cigs.d: SP	19.460	34.3874	4.5326	0.0109			
H.Dose: Cigs.d: Weight	21.370	37.8144	4.9256	0.0112			
H.Dose: Height: Cigs.d: Weight	-0.1209	-0.0276	-0.2142	0.0114			
H.Dose: Height: Cigs.d: SP	-0.1098	-0.0251	-0.1945	0.0114			
H.Dose: Cigs.d	-92.680	-21.100	-164.25	0.0115			
H.Dose: Height: Cigs.d	0.5230	0.9293	0.1167	0.0120			
Females							
Height: Cigs.d: Weight	-0.00041	-0.0002	-0.0006	0.00002	8.25	3, 214	<0.0001
Height: Cigs.d: SP : Weight	0.00008	0.0001	0.0000	0.00003			
H.Dose: H.Dura.n: Height: Cigs.d	2.2E-06	0.0000	0.0000	0.05160			

For peer review only

Table 6.: Longitudinal Adjusted Regression Models

Group	Variable Statistics					Model	
Model / Parameter	Value	Upper Limit	Lower Limit	t-value	P-value	AIC*	LogLik*
Both Sexes (DF=606)							
Height	-0.0304	-0.0253	-0.0355	-11.7198	0.0E+00	1238.239	-592.1197
SP	0.7947	0.9807	0.6087	8.3772	0.0E+00		
Days:H.Dura.n	0.0003	0.0004	0.0002	4.3556	0.0E+00		
CA:Height	0.0066	0.0078	0.0054	10.9049	0.0E+00		
Days: CA:H.Dura.n	-0.0001	-0.0001	-0.0001	-4.2946	0.0E+00		
H.Dura.n: Cigs.d:Height	-5.3E-05	-2.8E-05	-7.8E-05	-4.1566	0.0E+00		
Days:H.Dose: Cigs.d:Height	2.1E-06	3.1E-06	1.1E-06	4.3108	0.0E+00		
Days: CA:H.Dose: Cigs.d:Height	-6.0E-07	-2.1E-07	-9.9E-07	-4.2968	0.0E+00		
CA:H.Dura.n: Cigs.d:Height	1.4E-05	2.1E-05	7.3E-06	4.0217	0.0001		
Days: CA: Cigs.d:Height	0.0E+00	0.0E+00	0.0E+00	3.7701	0.0002		
Days:H.Dura.n: Cigs.d	-1.1E-05	-4.9E-06	-1.7E-05	-3.4709	0.0006		
Cigs.d:Height	0.0007	0.0011	0.0003	3.3714	0.0008		
Days: CA:H.Dose:H.Dura.n: Cigs.d:Height	0.0E+00	0.0E+00	0.0E+00	3.2705	0.0011		
Days: CA:H.Dura.n: Cigs.d	2.5E-06	4.1E-06	9.3E-07	3.2365	0.0013		
CA: Cigs.d:Height	-0.0002	0.0000	-0.0004	-3.2045	0.0014		
Days	-0.0022	-0.0008	-0.0036	-2.9194	0.0036		
H.Dose:H.Dura.n: Cigs.d	0.0102	0.0173	0.0031	2.8304	0.0048		
CA:H.Dose:H.Dura.n: Cigs.d	-0.0026	-0.0006	-0.0046	-2.6804	0.0076		
Days: CA	0.0005	0.0009	0.0001	2.2438	0.0252		
Days: CA:H.Dose:Height	6.8E-06	1.3E-05	7.2E-07	2.1984	0.0283		
Days:H.Dose:Height	-2.2E-05	-2.4E-06	-4.2E-05	-2.1747	0.0300		
Days:H.Dose:H.Dura.n	-3.5E-05	-1.7E-06	-6.8E-05	-2.1086	0.0354		
H.Dose: Cigs.d	-0.1067	-0.0065	-0.2069	-2.0888	0.0371		
CA:H.Dose: Cigs.d	0.0290	0.0580	0.0000	1.9645	0.0499		
Males (DF=436)							
Height	-0.0302	-0.0233	-0.0371	-8.7423	0.0E+00	913.00	-435.50
SP	0.6376	0.8542	0.4210	5.7717	0.0E+00		
CA:Height	0.0064	0.0080	0.0048	8.4711	0.0E+00		
Days:H.Dura.n	0.0003	0.0005	0.0001	3.8907	0.0001		
Days: CA:H.Dura.n	-0.0001	-0.0001	-0.0001	-3.8395	0.0001		
Days:H.Dose: Cigs.d:Height	1.6E-06	2.4E-06	8.2E-07	3.8181	0.0002		
Days: CA:H.Dose: Cigs.d:Height	-5.0E-07	-3.0E-07	-7.0E-07	-3.7217	0.0002		
Days	-0.0029	-0.0013	-0.0045	-3.6624	0.0003		
Days: CA	0.0007	0.0011	0.0003	3.3251	0.0010		
H.Dose:H.Dura.n: Cigs.d:Height	2.7E-06	4.3E-06	1.1E-06	3.2331	0.0013		
Days: CA: Cigs.d	5.0E-06	8.1E-06	1.9E-06	3.1839	0.0016		
H.Dura.n: Cigs.d:Height	-2.8E-05	-1.0E-05	-4.6E-05	-3.1058	0.0020		
CA:H.Dura.n: Cigs.d:Height	7.1E-06	1.2E-05	2.4E-06	2.9496	0.0034		
Days:H.Dura.n: Cigs.d	-8.3E-06	-2.2E-06	-1.4E-05	-2.6824	0.0076		
Days: CA:H.Dura.n: Cigs.d	2.0E-06	3.6E-06	4.3E-07	2.5562	0.0109		
Days: CA:H.Dose:H.Dura.n: Cigs.d:Height	0.0E+00	0.0E+00	0.0E+00	2.2838	0.0229		
Cigs.d:Height	0.0004	0.0008	0.0000	2.2799	0.0231		
CA: Cigs.d:Height	-0.0001	-2.0E-06	-2.0E-04	-2.1938	0.0288		

For peer review only

Females (DF=169)							
Height	-0.0279	-0.0208	-0.0350	-7.6753	0.0E+00	394.69	-187.35
SP	1.2880	1.6663	0.9097	6.6744	0.0E+00		
CA:Height	0.0064	0.0074	0.0054	11.7493	0.0E+00		
Days: CA:H.Dose:H.Dura.n:Height	1.3E-06	2.3E-06	3.2E-07	2.7371	0.0069		
Days:H.Dose:H.Dura.n:Height	-4.5E-06	-1.2E-06	-7.8E-06	-2.7147	0.0073		
Days: CA:H.Dose:H.Dura.n: Cigs.d:Height	-1.0E-07	-1.0E-07	-1.0E-07	-2.6052	0.0100		
Days:H.Dose:H.Dura.n: Cigs.d:Height	2.0E-07	4.0E-07	4.0E-09	2.5589	0.0114		

For peer review only

~~*Opioid*~~

Impact of Lifetime Opioid Exposure on Arterial Stiffness and Vascular Age: Cross-sectional and Longitudinal Studies in Males and Females.

Short Title:

Opioids as Cardiovascular Risk Factors

Albert Stuart Reece

Gary Kenneth Hulse

School of Psychiatry and Clinical Neurosciences,

University of Western Australia.

Crawley WA 6009, Australia.

*** Correspondence to**:

Albert Stuart Reece

39 Gladstone Rd.,

Highgate Hill,

Brisbane, Queensland, Australia.

Ph: (617) 3844-4000

FAX: (617) 3844-4015

Email: sreece@bigpond.net.au

Word Count: ~~8,656~~ 6,118.

Keywords – arterial stiffness, opioid dependence, vascular ageing, biological age, acceleration of ageing

For peer review only

Structured Abstract

Objective. To characterize and compare the potentiation of arterial stiffness and vascular ageing by opioids in males and females.

Design. Cross-sectional and longitudinal studies of 576 clinical controls and 687 opioid dependent patients (ODP) on 710 and 1,305 occasions over a total of 2,382 days (6.52 years), 2006-2011. Methodology: Radial Pulse Wave Analysis with Atcor SphygmoCor system (Sydney).

Setting. Primary care.

Participants. Controls: General practice patients with non-cardiovascular disorders, and university student controls. ODP: Patients undergoing clinical management of their opioid dependence. Controls had lower chronologic ages (CA) than ODP (30.0±0.5 vs. 34.5±0.3, mean±S.E.M., P<0.0001). Subjects were 69.6% and 67.7% male, and 16.0% and 92.3% were smokers (P<0.0001) for controls and ODP respectively. 86.3%, 10.3% and 3.4% of ODP were treated with buprenorphine (6.98±0.21mg), methadone (63.04±4.01mg) or implant naltrexone respectively. BMI was depressed in ODP.

Interventions. Nil.

Primary Outcome Measures - Vascular Reference Age (RA) and the ratio of the vascular age to the chronological age (RA/CA). Secondary outcome measures: Arterial Stiffness including Augmentation Index.

Results. After Body Mass Index -adjustment, RA in ODP was higher as a function of CA and of time (both P<0.05). Modelled mean RA in control and ODP males was 35.6 and 36.3 years (+1.97%), and in females 34.5 and 39.2 years (+13.43%). Changes in RA and major arterial stiffness indices were worse with CA in females (P<0.01). Quadratic, cubic and quartic functions of opioid exposure duration out-performed linear models with RA/CA over CA and over time. The opioid dose-response relationship persisted longitudinally after multiple adjustment from P=0.0013 in males and P=0.0073 in females.

Conclusion. Data show that lifetime opioid exposure is an interactive cardiovascular risk factor, particularly in females, is related to linear, quadratic, cubic and quartic functions of treatment duration and is consistent with other literature of accelerated ageing in OD patients.

For peer review only

Article Summary

Article Focus

- Despite striking consistency between several large epidemiological studies of the cardiovascular toxicity of long term opioid dependence, the subject is not well studied nor widely understood.
- There are only two (contradictory) studies of gender differences in significantly exposed populations in the published literature.
- The implications of organism-wide xenobiotic-induced inflammation have not been incorporated into the clinical practice of addiction medicine, our understanding of the inflammatory basis of atherogenesis, or the fact that vascular ageing is a major surrogate for systemic ageing across the whole organism.

Key Messages

- Opioid dependent patients (ODP) had and advance of mean vascular age from control of 35.61 to 36.31 years in males and 34.54 to 39.18 years in females which, after adjustment for BMI, were significant in both males and females cross-sectionally and longitudinally (all P<0.05). Both the acceleration of vascular ageing and arterial stiffness were worse in females (P<0.01) where it occurred 12 years earlier (29 ~~vs~~vs. 41 years).
- A dose-response relationship between lifetime opioid exposure and measures of arterial stiffness and vascular age was demonstrated in both sexes, which was robust to multiple adjustment for known cardiovascular risk factors.
- Use of quadratic, cubic and quartic functions of opioid duration exposure significantly improved the statistical fit of models of the ratio of vascular age (RA) to chronological age (CA, RA/CA ratio), thereby further highlighting the significance of the duration for which opioid agonism is administered.

For peer review only

Strengths and Limitations

- With 576 controls and 687 OPD the large size of this study was a signficant advantage, as was its combined cross-sectional and longitudinal design, its ability to control for many cardiovascular risk factors, the sex specific analysis, and the diverse output of cardiovascular parameters of the Atcor / SphygmoCor Pulse Wave Analysis technology employed.

- The use of detailed statistical modelling of relationships between factors, dose-response modelling, polynomial modelling in opioid exposure duration, inter-quartile trend effects, and adjustment for known cardiovascular risk factors added further analytical power to the study.

- As the major study limitation was its lack of other measures of organism ageing and its lack of mechanistic and ageing medicine-specific biomarkers, further studies incorporating these refinements are indicated.

For peer review only

Introduction

Opioid dependence is a condition of increasing public health importance.

Opioid Dependence is associated with an impressive array of traditional and novel cardiovascular risk factors and associations including hypertension [1], hyperlipidaemia [2-3], tobacco consumption, increased body weight [4], hyperglycaemia [3], hyperfibrinogenaemia [3], diffuse polyclonal gammopathy and immune stimulation [5-6], elevated high sensitivity CRP [7], elevated cytokines [8], relative hypercalcaemia and hyperphosphataemia [9] and reduced circulating endothelial progenitor cells [10]. Furthermore opioid dependence has been associated with atherosclerotic disease in both the coronary [11-18] and cerebral [14 18] circulations. In a 33-year review of an opioid maintenance program in California in 2004, death from cardiovascular disease was noted to account for three times as many years of life lost in the treatment group than in the general population [18]. In 2007 an Iranian surgical group identified that opium dependent patients were at increased risk of requiring open coronary revascularization with an odds ratio of 1.8 after adjustment for other risk factors [15]. Moreover in this series there was a dose-response relationship between the amount of opioids consumed and the severity of the coronary disease. It was also shown that the coronary disease occurred two years earlier in opioid dependent persons than in controls [16]. This group further identified that opium use was the strongest cardiovascular risk factor in their population of men, with effects greater than traditional risk factors [17]. The sex specific finding in this study was felt to be related only to a reduced prevalence of opium consumption amongst females.

In reviewing over 800 fatal cases of opioid toxicity in Australia in 2006 Darke et al. found that 17% of decedents over the age of 44 years had coronary stenoses in excess of 75% [12]. Similarly when comparing 1193 post mortem cases of heroin and methadone toxicity the Sydney group found 2-3 times elevated adjusted odds ratios of ventricular hypertrophy, and myocardial, interstitial and perivascular fibrosis in the methadone treated group, consistent with the effects of more consistent opioid agonism in such patients [11]. In a 21-year follow-up of 42,676 patients undergoing maintenance opioid agonist treatment in Australia, including 425,998 person-years, Degenhardt et al. identified cardiovascular death as occurring 2.2

For peer review only

times as often as in the wider community[13]. Indeed in this study elevated rates of death with severe organ dysfunction were noted which appeared to act as a backdrop to the very high rates of death by overdose generally reported in such cohorts (see Web Appendix 6 of this citation).

Field Code Changed

In 2012, a prospective population based study of 50,045 people including 234,098 person-years reported hazard ratios of death from coronary artery disease (CAD) of 1.9-fold (95%C.I. 1.57-2.29) and of cerebrovascular disease (CeVD) of 1.68-fold (C.I. 1.29-2.18)[14]. Both were worse in females (CAD: male adjusted HR (AHR) 1.58, female AHR 2.90; CeVD male AHR 1.49, female AHR 1.97). An earlier study from Iran did not confirm the cardiovascular salience of opioid exposure amongst females as compared to males, but the number of exposed females in this study was small, which likely compromised the power of the analysis to detect an efffecteffect[17]. Furthermore recent reports of 1,263 patients found elevated rates of vascular age in patients treated with methadone compared to other agents[19] and improved vascular ages accompanying the long term drug-free condition[20].

Field Code Changed
Field Code Changed
Field Code Changed
Field Code Changed

One large case control study found a dose response relationship between opium exposure and the severity of angiographically assessed coronary artery disease in a diabetic population[21].

Field Code Changed

Moreover opioid patients dependent on short acting opioids such as morphine, oxycodone and heroin undergo the clinical syndrome of opioid withdrawal several times daily. This syndrome is a hyper-adrenergic state characterized by neurological and cardiovascular stimulation which in its more severe manifestations includes hypertension and tachycardia[22] and a reduction of subendocardial perfusion[23]. This negative aversive experience is one of the key reasons many opioid dependent patients (ODP) continue to use narcotics.

Field Code Changed
Field Code Changed

Important as the above collected findings are, other evidence suggests that the impact of elevated vascular age has implications on the health and ageing of the organism as a whole beyond those limited primarily to the cardiovascular system. It has been repeatedly proposed that vascular ageing is a principal component of biological organismal ageing, by virtue of the contribution made by cardiovascular causes to the overall death rate in developed nations, and also the importance of the vascular niche common to many stem cell beds[24-25]. This view closely accords with the finding in many of the above studies and particularly that by Degenhardt et al[13] that elevated rates of overdose occur on the background of advanced

Field Code Changed
Field Code Changed

For peer review only

disease in other organs [1 12 18]. Opioid dependent patients have many stigmata of accelerated ageing including increased rates of: hair greying, erosion of alveolar bone from chronic periodontitis [26], osteoporosis [27], chronic respiratory disease [11 13 18], chronic renal disease [11], diffuse senescent-mimetic immune stimulation [5-6 28], diabetes [3], neuropsychiatric disorders, multi-system disease [11-12], reduced circulating stem cell counts [10], and many malignancies [13 29]. Thus the call has been made for geriatricians to be appointed to the care of opioid dependent patients beyond the age of 50 years, due to the high prevalence of multi-system disease [1].

The possibility therefore arises that an improved understanding of the increased and accelerated profile of degenerative disease seen in opioid dependent patients may enhance our understanding, and in time our management, of age related disorders in the rest of the community.

As this clinic sees both opioid dependent patients and the general community, we were ideally situated to compare these two groups. The availability of the mobile SphygmoCor system from Atcor in Sydney means that a host of central cardiovascular data is available to the clinician in a non-invasive and rapid manner, providing quantitation of important subclinical endophenotypes of central vascular stiffness and cardiovascular and therefore organismal ageing. The central investigative question to be prospectively assessed was whether there were changes in major central cardiovascular parameters in the opioid dependent group, and if so are they robust to adjustment for other confounding factors. Several earlier papers from our group have considered this issue in both cross-sectional [30-31] and longitudinal [32-33] studies in each sex separately and drawn conclusions generally confirmatory of this hypothesis. It has been shown that the type of opioid pharmacotherapy affects the severity of opioid related potentiation of arterial stiffness [19], that induction of the drug free state after implantable opioid antagonists reverses this finding [20], and that opioid withdrawal is also associated with elevation of cardiovascular age and arterial stiffness parameters [23]. However a formal comparison of the effects by gender has not been possible in these earlier reports as they primarily considered single sex data. Gender comparison therefore forms the major focus of the present report which is a compilation of much of this earlier data.

Methods

For peer review only

Patient Selection. Control subjects (N=576) were recruited opportunistically from patients presenting for health examination checks for insurance or employment medical examinations or from patients presenting with minor health problems such as ear blockages, minor psychological disorders, or University students. Patients with known cardiovascular disorders such as hypertension, renal disease or diabetes or diseases known to perturb the CVS subacutely such as infections or pregnancy, were excluded. Opioid dependent patients (N=687) were also recruited opportunistically and were under treatment with either methadone (N=71), buprenorphine (N=593) maintenance, or naltrexone implant (N=23). All were sampled opportunistically at the time of their clinic visits May 2006 - December 2012.

Patient Treatment. Opioid dependent patients maintained on, methadone or buprenorphine were managed by accepted clinical algorithms by their usual health providers for their drug dependency. The naltrexone implants used for the management of heroin dependence were manufactured by “Go Medical” industries in Perth, Western Australia under Commonwealth of Australia Therapeutic Goods Administration (TGA) GMP conditions and administered under the compassionate Access arrangements of the Special Access Scheme authorized by the TGA as described elsewhere [34]. Patients were recalled for re-testing at two and five years post baseline assessment, and assessed opportunistically. Any patient with a known chronic or subacute cardiovascular condition were excluded from participation. Frequency and quantity of substance use by substance type (inclusive heroin) was assessed at the initial time and subsequently. Other opioids were converted into morphine equivalents, and then into heroin equivalents at the rate of 1g of street heroin = 0.5g morphine. The duration of opioid use was counted from the time of first use. If it became known that alcohol had been consumed in the few hours prior to the test or that stimulants had been used in the three days prior, or that patients were acutely affected by short acting ~~opiate~~opioids, that day’s studies were excluded.

PWA Studies. Patients were positioned supine for the performance of the test and allowed to rest for five minutes prior to the test. Consumption of food, drink, alcohol or tobacco was not restricted, but patients were not allowed either to sleep or talk during the testing procedure. Pulse wave analysis by applanation tonometry was performed on the right radial artery unless

For peer review only

it was unavailable (due to trauma, surgery or congenital causes). The brachial blood pressure was taken in the opposite arm using the oscillometric Omron HEM 907 device. The data was collected and analyzed by the SphygmoCor software. Patients were studied in quintuplicate, and adequate studies were averaged for that day. Acceptable studies had an Operator index of greater than 70%, and were not inconclusive. Details of patients' prior drug use was also taken at the time of the study performance and entered into the database. Time in days was measured from the time of the first PWA study.

The SphygmoCor software used for the PWA studies calculates a number of major indices including Vascular Reference Age (RA), Chronologic Age (CA), Central Augmentation Pressure at Heart Rate 75 (C_AP_HR75) the Central Augmentation Pressure / Pulse Height Ratio at Heart Rate 75 (C_AGPH_HR75) also known as the Augmentation Index, Central Pulse Height (C_PH), Central Augmentation Load (C_AL), Central Augmentation Time Index (C_ATI), Peripheral–Central Pulse Pressure Amplification Ratio (PPAmpRatio), Maximal Peripheral Rise of dP/dT (P_MAX_DPDT), Central Systolic Pressure (C_SP), Central Diastolic Pressure (C_DP), Central End Systolic Pressure (C_ESP), Central Mean Pressure (C_MEANP), the Central Diastolic Time Index (C_DTI), the Central Tension Time index (C_TTI), and an index of subendocardial perfusion known variously as the Subendocardial Perfusion Ratio (SEVR), the Central Stroke Volume Index (C_SVI) or the Buckberg ratio defined as the C_TTI / C_DTI.

Statistics. Data are presented as the mean $\pm$ S.E.M. Categorical data were studied using "EpiInfo" 7.0.8.3 using the Chi squared test obtained from the CDC Atlanta, Georgia or the Fisher Exact test if the numbers in a cell were less than 20. Continuous categorized data were analyzed in "Statistica" 7.1 from Statsoft, Oklahoma using Student's t-tests. T-tests with separate variances were used where Levene's test was significant. Continuous variables such as RA, CA, RA/CA, variously corrected RA, SP, Weight, HDL, BMI, and systolic pressure were log transformed in accordance with normality assumptions based on the Shapiro-Wilks test. High sensitivity CRP and the heroin dose were arcsinh transformed which is a similar transform to logarithmic, but accepts arguments of zero. Time (as days) was measured from the time of the initial PWA study. The time since the last cigarette was recorded and analyzed as a factor with cut-off points at 30, 60, 120, 180 and > 180minutes, and a non-

For peer review only

smoking category. For some analyses lifetime heroin exposure was cut into quartile categories with divisions at 0, 0.01, 2.49, 6.59 and 350 g-years respectively, yielding 583, 207, 226 and 227 patients in each group respectively. Multiple regression was performed in "R" 2.15.2 obtained from the University of Melbourne Central "R" Archive Network mirror. Mean model estimates for various groups were prepared by inserting the mean parameter values into the untransformed final models. Multiple linear regression of truncated variables such as RA (minimum value of 20, maximum value of >80) were analyzed using tobit multiple regression techniques from the Applied Econometrics library in "R" (AER). RA's of individual studies returned as >80 were assigned as 88. Time-dependent repeated measures analysis was performed using non-linear mixed effects (nlme) regression module in "R" using restricted equation maximum likelihood (REML) techniques. Random effects were assigned to unity and the patient identification code. When nlme models were compared they were converted to maximum likelihood (ML) methods to allow Anova testing. In each case model reduction was performed manually by the classical method involving removing the least significant term. The log-likelihood value was abbreviated to LogLik. Graphs were prepared with reshape2 and ggplot2. Where loess curves were fitted, the span used was 0.95. T-tests were two-sided. P<0.05 was considered significant.

Ethical Approval. All patients provided their informed consent to participate in the study and for their medical treatment. The study was approved by the Human Research Committee of the Southcity Family Medical Centre, Brisbane, Queensland, Australia, which is registered with the National Health and Medical Research Council.

For peer review only

Results

576 control patients were compared with 687 opioid dependent patients. The mean ages were 30.0+0.5 and 34.5+0.3 (mean + S.E.M.) years (t=-8.262, dF=1082.22, P<0.0001, Supplementary Table 1), with 401 (69.62%) and 465(67.69%) males respectively (Chi^2 = 0.54, P=0.46). 15.97% and 92.29% smoked tobacco (Chi^2=746.56, P<0.0001). The mean CA of the whole group was 32.5+0.3years. The mean CA of the males was 32.1+0.3 and of females 33.2+0.5 years. 7.47% and 5.97% of the control and ~~opiate~~opioid dependent groups were aged over 50 years respectively (Chi^2 ~~Squ.~~=1.13, P = 0.29). Amongst the males these ratios were 5.74% and 6.24% (Chi^2 ~~Squ.~~ =0.1), and amongst the females they were 11.43% and 5.41% (Chi^2 ~~Squ.~~ = 4.79, P = 0.029, Fisher Exact Test 0.19-0.98; pooled data, Corr. Chi^2 ~~Squ~~ = 3.8, P = 0.051). Racial stratification was not performed as our population is around 90% Caucasian in background. Other baseline biometric, drug use and laboratory values are given in Supplementary Table 1. Patients were studied longitudinally on 710 and 1,305 occasions. The period of repeat study, measured from the time of the first PWA study, was 624.11 + 18.74 days (range 1-2,382 days; 495 (60.36%) studies performed beyond 360 days). Details of the numbers of repeat studies are given in Supplementary Table 2.

Formatted: Superscript

Formatted: Superscript

Formatted: Superscript

Formatted: Superscript

Comparative PWA values are given in Supplementary Table 3. Most augmentation values and the C_SP and C_ESP are noted to be higher in the opioid dependent group consistent with their being an average of 4.59 years older.

Figure 1 shows different vascular age and augmentation parameters corrected for various features, against CA. This figure shows that the BMI was lower in the opioid dependent group than the controls both as a factor (factor est.=0.0719, est. S.E. = 0.0327, t=2.197, P=0.0282) and in interaction with CA (BMI: CA est.=-0.0023, est. S.E. = 0.0009, t=-2.350, P=0.0189; model F=34.94, dF=3, 1258, P~~<2.2E-16~~<0.0001). In the longitudinal series BMI was lower as a factor (est.=-0.0174, dF=812, P=0.0470; model AIC= -3722.55, LogLik=1867.27). This result may be the effect of cumulative diversion of resources over time. Figure 2 shows similar indices over time. Supplementary Table 4 presents the results of the cross-sectional data where BMI corrected RA is regressed against CA and addictive status. In the whole group both opioid dependency status (P=0.0265) and the CA: addiction interaction (P=0.0205) are significant. Similar results were found in each sex. Similar results were also found in the longitudinal study when the RA/CA ratio was regressed against CA,

For peer review only

time and dependency status both for opioid dependency as a factor (P=0.0102) and in interaction with CA (P=0.0028; details in Supplementary Table 4).

Inclusion of tobacco exposure along with opioid exposure in these models did not alter these findings (data not shown).

As shown in Supplementary Figures 1 and 2, these changes were found to be more pronounced with CA in females. These data are presented corrected for the changes in BMI in Figures 3 and 4. Details for these significant differences are presented in Table 1.

Figure 5 shows these data by sex with loess curves fitted (span=0.95). This strongly suggests that an inflection point is reached about the age of 30 in females, which is not seen in males until about 40 years of age. This was quantified by dichotomizing CA from age 41 in males and 29 in females. As shown in Table 2 in males in the older age category, the addictive status and their interaction were significant (P= 0.0010, 0.0021 and 0.0008 respectively), whilst in females the older age: addiction interaction was significant (P=0.0195, analysis restricted to women <55 years).

The regression changes are quantified for some of the major indices of arterial stiffness in cross-sectional data in Supplementary Table 6 (all P<0.005). Similar significantly different sex specific data were found in the longitudinal dataset when the RA was regressed against CA, time, sex and addictive status (most P<0.02, Supplementary Table 7).

When the mean sex-specific values for CA and BMI are inserted into the equation of Table 3 the mean vascular ages of the control groups amongst all patients, males and females was 35.42, 35.61 and 34.45 years, whilst the corresponding ages of the ODP's was 37.24, 36.31 and 39.18 years, representing incremental gains of 5.14%, 1.97% and 13.43%.

When quartiles are defined for lifetime heroin exposure the results shown in Figure ~~8~~6 are found. Similar effects longitudinally are shown in Supplementary Figure 3. These results are quantitated in Supplementary Table 8 for both cross-sectional and longitudinal data compared to non-exposed controls. In the cross-sectional analysis the RA/CA ratio was regressed against the CA and Opioid exposure quartile. In the longitudinal analysis the RA/CA ratio was regressed against time, CA and the opioid exposure quartile. Whilst the CA: Level 3

For peer review only

opioid exposure interaction is of borderline significance in all patients and females (P=0.0546 and 0.0568, but not in males) in the longitudinal study the age: level 2 exposure is significant in all patients and males (P=0.0003 and 0.0110) and level 3 opioid exposure approaches significance in females both as a factor and in interaction with age (P=0.0501 and 0.0547). Supplementary Table 9 presents the results of a longitudinal regression of age-corrected indices of cardiovascular stiffness against time and opioid exposure quartile by gender, and notes a highly significant effect by exposure quartile in both genders.

The data was formally interrogated for a trend between the different quartiles of lifetime opioid exposure using the contrasts polynomial function in "R". Significant contrasts between the various quartiles for lifetime opioid exposure for the age: opioid exposure interaction was indeed demonstrated in all patients (est. = 0.1657, S.E. = 0.0787, t = 2.104, P = 0.0356; Model Adj. R^2 = 0.4265, F = 133.0, dF = 7, 1235, P < <0.0001~~2.2x10^{-16}~~), such that moving up a quartile level at age 60 increased the modelled RA by 8.42% to 65.051 years, and movement from the first (non-exposed) quartile to the highest (level 3) quartile increased the modelled RA 25.26% to 75.154 years.

Some environmental exposure – pathology relationships have been shown to be power functions of the duration of exposure and are therefore particularly sensitive to the periods for which exposure occurs[35]. Since opioid administration is often prescribed and recommended as being of indefinite duration this is particularly relevant to the present discussion. Therefore a detailed study of the power relationships of the duration of opioid administration was undertaken. Table 3 shows that simple regression models of RA/CA against the dose-duration interaction were significantly related to linear, quadratic, cubic and quartic terms in opioid exposure duration. Moreover when RA/CA was regressed against a model including interactive terms in linear, quadratic, cubic and quartic terms, all four were significant. When these models were compared by ANOVA studies the quadratic (AIC=82.888) was noted to be significantly better than the linear, cubic or quartic models (Anova tests, all P<0.05), but not significantly different from the combined model (AIC=83.731, P=0.87; Supplementary Table 10).

Field Code Changed

When a similar exercise was performed in the longitudinal dataset, similar results were obtained (Supplementary Table 11). In this study RA/CA was regressed against time and the opioid exposure quartile. As a combined linear-quadratic-cubic-quartic model failed to

For peer review only

converge, the highest order model used was linear-quadratic-quartic. Again quadratic and quartic terms in exposure duration persist in the final model. In this case the quadratic model (AIC=1040.85) was significantly superior to all the others but was not significantly better than the linear-quadratic-quartic model (AIC= 1041.09, P=0.082; Supplementary Table 12).

The data was interrogated for the presence of a dose-response relationship directly by examining continuous terms in opioid dose and duration regressed against the RA/CA ratio in both datasets. As shown in Table 4 the dose-duration interaction is significant in all patients considered together, in males and in females (P <0.0001~~=0.000037~~, 0.000~~29~~3 and 0.0236) in the cross-sectional dataset, whilst in the longitudinal dataset the parallel results are P = 0.0004, 0.0008 and 0.0127.

The final step of the analysis was to consider whether the significance of the opioid dose and duration might persist after adjustment for all known cardiovascular risk factors. In preliminary analyses lipidic factors were invariably eliminated during the model reduction process. Moreover, as discussed in the Introduction, opioids themselves are likely to perturb both lipid function, and also have a robust immunostimulatory action. Therefore including immune markers is likely to over-correct the model. Similarly when the time since the last cigarette consumption was included in the cross-sectional and longitudinal models it was eliminated during model reduction. For these reasons the final cross-sectional formula used regressed RA/CA against interactive terms in opioid dose and duration, height, tobacco consumption, systolic blood pressure and weight. The longitudinal model was similar but had an additive term in weight. These were the terms which remained significant in the models in preliminary exploratory analyses for log (RA). Further consideration of the clinical justification for the choice of this five way interaction is included in the Discussion section.

The results shown in Table 5 were found for the cross-sectional data. This table shows that significant interactions including opioid dose-duration terms are found in all patients (P=0.0261) and in males (P=0.0017; Adj. R^2=0.0815), whilst in females this interaction approaches significance (P=0.0516). When a similar exercise is performed on the longitudinal data, significant interactions are seen from P = 0.0011, 0.0013 and 0.0073 and above in each group respectively (Table 6).

For peer review only

Supplementary Table 13 presents the results of final models in which complete multiple regression was performed in the cross-sectional dataset for all pertinent variables. (Log) RA/CA was regressed against interactive terms in opioid dose and duration, height, tobacco use, systolic pressure and high sensitivity CRP together with additive terms in weight, cholesterol and HDL. Opioid dose and duration persist in the final model in males and in all patients, but not in females. Interestingly CRP features in most of the terms of these final models.

These overall results were broadly confirmed by the running of further analyses including by the use of by additivity and variance stabilization (AVAS) in R [36]. In particular we analysed a range of models including different polynomial terms in known predictors, and matching models augmenting the known risk factors with heroin use. Models with and without heroin use were compared. These analyses confirmed that the model fit for the relationship between log (RA/CA) and the predictive variables was significantly improved by the inclusion of terms for opiate exposure (dose and duration) amongst the male opiate dependents, in the opioid dependent groups in both sexes and in all patients; that the relationship between the various model parameters was (unsurprisingly) unlikely to be of a strictly linear form; and that superior model fits for log (RA/CA) were likely to be accompanied by various higher order interactions between the most powerful predictive independent variables namely (log) SP, weight, height, tobacco consumption and opioid exposure dose and duration.

Formatted: Font: (Default) Times New Roman, 11 pt

Formatted: Line spacing: 1.5 lines

Formatted: Font: (Default) Times New Roman, 11 pt

Formatted: Font: (Default) Times New Roman, 11 pt

Formatted: Font: (Default) Times New Roman, 11 pt

For peer review only

Discussion

The major findings of this study are that indices of vascular age and arterial stiffness are worse in opioid dependence compare to opioid naïve controls with mean calculated ages elevated by 1.97% in males and 13.43% in females. The effect was thus more marked in females. A significant effect was found on both vascular age and augmentation index by exposure quartile after correction for CA and BMI. The RA/CA ratio was found to be related to power functions of the opioid duration of exposure in both cross-sectional and longitudinal analyses. A dose-response relationship was demonstrated with lifetime opioid exposure. ~~And~~ In particular the effect of opioid exposure was robust, and remained after multiple adjustment in cross-sectional and longitudinal studies.

These findings should be interpreted in the light of the relatively modest degree of opioid exposure to which these patients were exposed. Whilst the dose and duration of opioids used by patients in this study is typical of that seen in many published series, the treatment of these patients was mostly with the relatively mild partial agonist buprenorphine, with only modest doses employed (mean 6.98±0.21mg): this dose level being much lower than the commonly recommended (18-24mg)[37~~36~~]. Moreover buprenorphine has been shown to be more mild in its cardiovascular effects than the full agonist agent methadone . For this reason the results reported herein should be seen as a lower bound on the effects which may be observed.

Field Code Changed

The findings in relation to central vascular stiffness and age being related to power functions of the treatment duration is of great importance. Chronic opioid agonist treatment is often seen as relatively benign and partly for this reason is frequently recommended for very long term or indefinite administration[38-39~~37-38~~]. However the demonstration here that the effects likely compound with time have major clinical practice implications, and have not been previously noted. The 1.97% effect described here for males, compounded over 30 years equates cumulatively to 179.5% gain and in diminution to 44.95% loss. Similarly the 13.43% gain seen in females compounded over 30 years is a 4349.02% gain and a 98.66% loss in reduction. The effects therefore of these process maintained over the long term on stem cell diminution or immune stimulation are potentially very considerable.

Field Code Changed

The exacerbation of the effect in females was clearly demonstrated in Figures 3-5~~7~~, Tables 1 and 2, and Supplementary Tables 4 and 5 and Supplementary Figures 1 and 2. This finding

For peer review only

has, to the authors knowledge, only been reported previously by an Iranian group[14]. If one looks closely at Figure ~~7~~5 several fascinating findings emerge. The vascular age algorithm used by the SphygmoCor software is based upon the augmentation index C_AGPH_HR75. This index has a clear curvilinear convex upward relationship to CA as shown in Figure ~~7~~5 in the lower panels, unlike the C_AP_HR75 which is directly linear[40~~39~~]. Hence the reduction at the higher ages on the C_AGPH_HR75 curve may be somewhat artefactual. If one looks at the upper panels of Figure ~~7~~5 the rise in RA with age in ODP may be seen to be broadly exponential in form, if the decline in slope above age 45 years is discounted. In females it appears that the rise in RA occurs some 10 years prior to that seen in males, a finding emphasized by the middle panels. This fits with a dramatic clinical transformation which is seen in opioid dependent females at around age 30, and in males at about age 40. Whilst the cause of this is unknown it may possibly be related to a more aggressive immune system in females which is in part hormonally mediated[41~~40~~]. In this context the demonstration that oestradiol directly stimulates telomerase was particularly provocative[42].

Some consideration of the comparability of the control and ~~opiate~~opioid dependent groups is appropriate. Significant differences were found between the control and ~~opiate~~opioid dependent groups in relation to chronological age, weight, BMI, smoking, cholesterol, LDL and hepatic and inflammatory laboratory markers, but these were controlled for by various the multiple regression techniques. The higher mean weight in the ~~opiate~~opioid dependent group may have been related in part to their higher age, and in part to the orexigenic effects of ~~opiate~~opioid agonism[43~~41~~], whilst serial resource diversion may explain the slower rate of gain of weight in this group over time. Higher rates of tobacco consumption may be related to either the common features of the addictive lifestyle or the pro-consumptive effects of opioid analog~~ui~~es[44~~42~~]. Significantly more of the females in the control group (11.4% vs. 5.41%) were aged over 50 years (P=0.029). However as no data was collected relating to hormonal status with regard to the menopause it is not possible to remark on the relative hormonal status of the two groups. ~~Opiate~~Opioid dependency is well known to be associated with hypothalamic hypogonadotrophic oligoamenorrhoea, so that the age-related hormonal suppression in the control group was somewhat balanced by the drug related hormonal suppression in the ~~opiate~~opioid dependent group. Clarification of this issue must await further studies.

Field Code Changed

Field Code Changed

Field Code Changed

Formatted: Font: (Default) Times New Roman, Do not check spelling or grammar, Superscript

Field Code Changed

Field Code Changed

For peer review only

Some consideration is appropriate of the clinical justification for the five-way interaction which was presented in the final multiple regression models of RA (Tables 5 and 6). Height is a key variable which relates to many techniques of measuring of arterial stiffness since it is related to the timing of arterial wave reflections, and it is hardly surprising that this remains in the final model[45-4643-44]. Height and weight will naturally interact and indeed the BMI is one formula which formalizes such interactions. The opioid dose-duration exposure is a standard way in which to account for lifetime opioid exposure, and has been used by WHO[35] and NIH[14] investigators amongst others. Other factors including laboratory parameters were not present in the final models. Therefore it is not surprising that this cluster of factors remains in the final model.

Field Code Changed

Field Code Changed

Field Code Changed

On the other hand CRP was prominent and present in most terms in the final model when RA/CA was interrogated by multiple regression (Supplementary Table 13) suggesting that innate immune processes may be implicated in the observed opioid-vasculopathy effect. The age-related course of high sensitivity CRP has been described in several earlier reports in our patient cohort[6 28 476 28 45]. Supplementary Table 1 shows significant elevation of CRP, ESR, serum globulins, and circulating lymphocytes and monocyte numbers in the opiateopioid dependent group. Consideration of the very flat age-dependent trajectories of these parameters in controls in our earlier reports shows that this effect is much greater in opiateopioid dependence than might be expected on the basis of the higher chronologic age in the opioid dependent group alone. This is in turn consistent with the important chronic inflammatory basis of many vascular diseases[48-5046-48] and the immune theory of organismal ageing[4846 5149].

Field Code Changed

Field Code Changed

Field Code Changed

Field Code Changed

As noted above, vascular age is frequently said to be a surrogate for organismal ageing. The present data are consistent with that view, and also with the literature cited of accelerated ageing in these patients. The present technique provides a mechanism to quantify these changes at the whole human level. Interestingly, while it is well known that telomere length can function as a "mitotic clock" of the biological aging process, it has been shown that oxidative stress can stochastically increase this rate, in part by inducing single strand telomeric breaks related to the high guanosine content of telomeric repeats (TTAGGG)n[52-54]. The heightened inflammatory activity associated with opioid dependence has been previously noted[5-7 28 55], and the involvement of this pathway mechanistically unifies the inflammatory,

Formatted: Font: (Default) Times New Roman, Do not check spelling or grammar, Superscript

Formatted: Font: (Default) Times New Roman, Do not check spelling or grammar, Superscript

For peer review only

oxidative, chromosomal, telomeric and stem cell hypotheses of aging, and accounts for the uniformity of the progeroid findings across organ systems [56] and the high prevalence of multisystem disease seen in opioid dependence [11-12 28]. ~~The present technique provides a mechanism to quantify these changes at the whole human level.~~

Formatted: Font: (Default) Times New Roman, Do not check spelling or grammar, Superscript

Formatted: Font: (Default) Times New Roman, Do not check spelling or grammar, Superscript

As no structural imaging techniques were performed it is not possible to say with certainty if the observed elevations in vascular stiffness were associated with fixed atherosclerotic lesions. However a large and weighty literature confirms the relevance of significance of elevations in vascular stiffness [45-46 57-59]~~[43-44 50-52]~~. Structural correlation must await further work.

Field Code Changed

The consistency of the present results with the previously reported data from various national and international groups [11-18 60]~~[11-18 53]~~ is clear in that we have confirmed the exacerbation of cardiovascular ageing and arterial stiffness in ~~opiate~~opioid dependency . However these earlier results have been extended by demonstrating a measurable major subclinical endophenotype of accelerated ageing antemortem, confirmed a dose-response relationship, demonstrated for the first time the significance of the improved model quadratic in ~~opiate~~opioid exposure duration, and shown that the ~~opiate~~opioid-related effect is robust to adjustment for known cardiovascular risk factors in both sexes. Moreover we confirmed that the effect is worse in females [14], and extended this result by showing that the differential effect commences in women over a decade earlier than in men (29 vs. 41 years). In that the PWA technique directly relates increased arterial stiffness to cardiovascular and therefore organismal ageing the present detailed studies link advanced cardiovascular age to many other indices of accelerated ageing in opioid dependency as have been shown by this [6-7 10 26 28 47 61-65]~~[6-7 10 26 28 45 54-58]~~ and other [66]~~[59]~~ groups. As such the present report is a meaningful and important extension of earlier reports from this project on the cardiovascular implications of opioid dependency [30-33], its treatment [19] and withdrawal [23] and the antagonist-induced reversal of these effects [20]. Hence the phenomenology of the present descriptive report is consistent with, and extends, extant reports in the published literature.

Field Code Changed

Field Code Changed

Field Code Changed

Field Code Changed

Field Code Changed

Field Code Changed

Field Code Changed

Field Code Changed

Whilst this study is not designed to be mechanistic in nature some brief comment on this issue may be appropriate, in part for its relevance to the consideration of possibly aetiologically causal pathways. Some of the most interesting questions to emerge from the

For peer review only

For peer review only - http://bmjopen.bmj.com/site/about/guidelines.xhtml

present work relate to the possible mechanisms underlying the plethora of changes which have been identified in opioid dependence as cardiovascular risk factors, as potentially atherogenic processes and as accelerants of ageing processes. Opioids have been linked with blockade of the cell cycle at cyclin dependent kinases 2, 4 and 6 by P16^{INK4A} and P21$^{WAF1/CIP1}$ [67-68~~60-61~~]. P16 is the gene product of CDKN2A located at the senescence locus on Chromosome 9p21.3 which is a hotspot for many GWAS studies, and from which three senescence related proteins and one senescence associated long non-coding RNA are transcribed [69~~62~~]. Furthermore opioid agonists have been shown to directly ligate the myeloid differentiation factor 2 (MD2)- toll-like receptor (TLR) 4 heterodimer leading to the downstream activation of the numerous powerful pathways linked to stimulation of the endotoxin receptor [70~~63~~]. Indeed systemic endotoxaemia was recently shown to modulate the expression of 6,025 genes [71~~64~~]. Some of the pathways activated downstream by TLR4 signalling include the cyclooxygenase, lipooxygenase, MAP kinase (JNK and P38), AP-1, inducible nitric oxide synthase (iNOS), phosphoinositol-3 kinase (PI3K), and TGFβ pathways [72~~65~~], many of which have been noted to be stimulated within the first few minutes of TLR4 ligation [73~~66~~], to be stimulated by opioids [70 72~~63-65~~], and to be associated with potentiating the effects of ageing [51 74~~49-67~~]. Many of these immune products directly exacerbate the stem cell blockade [75~~68~~]. This then implies that opioid dependency may be conceptualized as chronically, if largely subclinically, ill.

It is very exciting that new research has identified that these processes also act particularly in proopioimelanocortin (POMC) neurons of the paraventricular and arcuate nuclei of the mediobasal hypothalamus which are key determinants of the body's metabolic homeostasis, glucostatic and lipostatic functions, energy balance, appetitive drives, sympathoadrenal activation state, and even lifespan modulation in some organisms [76-78~~69-71~~], and that derangements in this region cause metabolic syndrome and its numerous related disorders in model organisms including mammals and likely also humans [79~~72~~]. These workers have shown that activation of the MD2/TLR4/IKKβ/NF-κB cascade induces endoplasmic reticulum, mitochondrial and autophagic stress, which along with reduced neurogenesis, increased POMC neuronal apoptosis [77~~70~~], and glial activation and reactive gliosis [79-80~~72-73~~] underlie these numerous disorders and derangements. Whilst this research focuses on TLR4 ligation of unsaturated fatty acids and oxidized low density lipoproteins, opioids have been shown to act similarly as xenobiotics through MD2-TLR4 [81~~74~~], and opioid agonists have been shown to produce POMC cell hyperpolarization and relative refractoriness [44~~42~~], whilst opioid

antagonists have the reverse effect [8275]. Small molecule TLR4 inhibitors are under development for several therapeutic applications [83-8476-77]. The confluence then of these two important lines of recent research has the potential to produce major conceptual and therapeutic advances in this field. A clear understanding of direct links from xenobiotic-TLR4-NF-κB signalling to sympathoadrenal tone and hypothalamic lifespan control has the potential to revolutionize the direction of therapeutic endeavours in the treatment of opioid dependence.

It is fascinating that the promoters for two of the key stem cell genes, Oct4 and Nanog have response elements in their promoters for STAT-1 and -3 which are commonly produced as a result of cytokine signalling, particularly by gp-130 cytokines such as IL-6 and leukaemia inhibitory factor (LIF) [85-8678-79]. Furthermore there is three way cross-talk between stem cell and metabolic –immune genes including STAT-3 and PPARγ [8780]. Other cytokines, such as IL-1 have been noted in opioid dependency [8] and are usually indicative of inflammasome activation, and fractalkine and MCP-1/CCL2 have also been found to be elevated and are relevant both to neuropathological and atherogenic processes [7063]. As TLR4 shares many ligands and co-chaperones with the receptor for advanced glycation end (RAGE) products this auto-amplificatory system may also be involved [88-9181-84].

This study had various strengths and limitations. The application of sophisticated cardiovascular measurement technology to the problem of drug addiction is unique to this group. Extended follow-up and recruitment of opioid dependent patients is also notoriously difficult. The statistical techniques employed herein are advanced and robust. Design strengths include its complimentary cross-sectional and longitudinal design. Limitations of the present work relate to difficulties quantitating frequently variable amounts of past drug use in the absence of a formal measurement instrument. Use of a formal instrument such as the addiction severity index, and spot tests for drugs and alcohol are indicated in future replications of this study. Employment of structural tests, and mechanistic studies to investigate pathophysiological pathways, would be advisable in future iterations of this work.

It is important to remember that the models and results described in this work are not represented as reliable and generalizable calibrations of the cardiovascular effects of opioid exposure, in combination with other well established risk factors. These models are the result of an empirical investigation of many correlated risk factors from a particular clinical cohort. It is most unlikely that similar model

Field Code Changed
Field Code Changed
Field Code Changed
Field Code Changed
Field Code Changed
Field Code Changed
Field Code Changed
Formatted: Font: Not Italic
Formatted: Indent: Left: 0", Line spacing: 1.5 lines

For peer review only

parameter estimates would be reproduced in a study of a different clinical cohort. The results serve a much more limited objective: is there evidence of an opioid effect cardiovascular health, taking account of other known risk factors. We suggest that there is evidence for such an effect; though we must accept that this evidence is in part contingent of the adequacy of the model for these known risk factors. This is a general problem facing all studies of correlated risk factors, in the absence of a well established mechanistic model. We would consider the impact of opioids to be established only if similar results were obtained in analysis of data from an independent case series. In this context the confirmatory nature of the present data to essentially identical results from the cross-sectional (only) data from Iran amongst males [16-17], and the various other findings noted in the literature review in the Introductory section of the present report are particularly pertinent.

Formatted: Font: Not Italic

Formatted: Do not check spelling or grammar, Superscript

Formatted: Font: Not Italic

In summary the present study documents elevated vascular stiffness and age associated with opioid use which is robust to multiple adjustment to other known risk factors. A strong dose-response effect was noted, and the duration of treatment administration was noted to be particularly important, being related to elevations in vascular age of the first second, third and fourth order. This finding indicates limitation of treatment duration wherever clinically possible. The effect was also worse in females. Overall this study raises many further mechanistic questions which indicate further study on this complex but important model condition.

For peer review only

Acknowledgements

The authors would like to thank Dr Mervyn Thomas of Emphron for assistance with the statistics and graphical design.

Conflict of Interest

Nil declared.

Sources of Funding

Nil.

Statement of Authorship

~~This work is the work of the authors in its entirety.~~The study was designed, the data collected, the analyses performed, the literature reviewed and the first draft written by ASR. GKH provided important intellectual input, assisted with study design, and revised and reviewed the manuscript. Both authors confirm the accuracy and integrity of the work and data collection and analysis.

For peer review only

References

1. Rosen D, Smith ML, Reynolds CF, 3rd. The prevalence of mental and physical health disorders among older methadone patients. *Am J Geriatr Psychiatry* 2008;16(6):488-97.
2. Cooper OB, Brown TT, Dobs AS. Opiate drug use: a potential contributor to the endocrine and metabolic complications in human immunodeficiency virus disease. *Clin Infect Dis* 2003;37 Suppl 2:S132-6.
3. Ceriello A, Quatraro A, Giugliano D. Opiate addict as diabetic patient? *Diabetes Care* 1988;11(5):443.
4. Kolarzyk E, Pach D, Wojtowicz B, Szpanowska-Wohn A, Szurkowska M. Nutritional status of the opiate dependent persons after 4 years of methadone maintenance treatment. *Przeglad lekarski* 2005;62(6):373-7.
5. Brunton LL, Lazo JS, Parker KL, editors. *Goodman and Gilman's the Pharmacologic Basis of Therapeutics*. Eleventh Edition ed. New York: McGraw Hill, 2006.
6. Reece A. S. Relative and Age Dependent Stimulation of Soluble and Cellular Immunity in Opiate Dependence. *Journal of Addiction Medicine* 2012;6(1):10-17.
7. Reece A. S. High Sensitivity CRP in Opiate Addiction: Relative and Age-Dependent Elevations. *Cardiovascular Toxicology* 2012;12(2):149-57.
8. Neri S, Bruno CM, Pulvirenti D, Malaguarnera M, Italiano C, Mauceri B, et al. Randomized clinical trial to compare the effects of methadone and buprenorphine on the immune system in drug abusers. *Psychopharmacology (Berl)* 2005;179(3):700-4.
9. Reece A. S. Absolute and Age Dependent Elevations of Serum Calcium and Phosphate and their Products in Clinical Opiate Dependence. *J. Subs. Abuse* 2013;Accepted 27th November 2012.
10. Reece AS, Davidson P. Deficit of circulating stem--progenitor cells in opiate addiction: a pilot study. *Substance abuse treatment, prevention, and policy* 2007;2:19-28.
11. Darke S, Duflou J, Torok M. The comparative toxicology and major organ pathology of fatal methadone and heroin toxicity cases. *Drug Alcohol Depend* 2010;106(1):1-6.
12. Darke S, Kaye S, Duflou J. Systemic disease among cases of fatal opioid toxicity. *Addiction* 2006;101(9):1299-305.
13. Degenhardt L., Randall D., Hall W., Law M., Butler T., Burns L. Mortality among clients of a state-wide opioid pharmacotherapy program over 20 years: Risk factors and lives saved. *Drug Alcohol Depend* 2009;105(1-2):9-15.
14. Khademi H, Malekzadeh R, Pourshams A, Jafari E, Salahi R, Semnani S, et al. Opium use and mortality in Golestan Cohort Study: prospective cohort study of 50,000 adults in Iran. *BMJ (Clinical research ed* 2012;344:e2502.
15. Sadeghian S, Darvish S, Davoodi G, Salarifar M, Mahmoodian M, Fallah N, et al. The association of opium with coronary artery disease. *Eur J Cardiovasc Prev Rehabil* 2007;14(5):715-7.
16. Sadeghian S, Dowlatshahi S, Karimi A, Tazik M. Epidemiology of opium use in 4398 patients admitted for coronary artery bypass graft in Tehran Heart Center. *J Cardiovasc Surg (Torino)* 2011;52(1):140-1.
17. Sadeghian S, Graili P, Salarifar M, Karimi AA, Darvish S, Abbasi SH. Opium consumption in men and diabetes mellitus in women are the most important risk factors of premature coronary artery disease in Iran. *International journal of cardiology* 2010;141(1):116-8.
18. Smyth B, Hoffman V, Fan J, Hser YI. Years of potential life lost among heroin addicts 33 years after treatment. *Preventive medicine* 2007;44(4):369-74.
19. Reece A.S., Hulse G.K. Impact of Opioid Pharmacotherapy on Arterial stiffness and Vascular Ageing: Cross-sectional and Longitudinal Studies. *Cardiovascular Toxicology* 2013;13(3):254-66.
20. Reece A. S., Hulse G.K. Reduction in Arterial Stiffness and Vascular Age by Naltrexone-Induced Interruption of Opiate Agonism. *British Medical Journal - Open* 2013.

Formatted: Font: Bold, Underline, Do not check spelling or grammar

Formatted: Font: Not Bold, No underline

Formatted: Font: Not Bold, Italic, No

Formatted: Font: Not Bold, No underline

Formatted: Font: Not Bold, Italic, No

Formatted: Font: Not Bold, No underline

Formatted: Font: Not Bold, Italic, No

Formatted: Font: Not Bold, No underline

Formatted: Font: Not Bold, Italic, No

Formatted: Font: Not Bold, No underline

Formatted: Font: Not Bold, Italic, No

Formatted: Font: Not Bold, No underline

Formatted: Font: Not Bold, Italic, No

Formatted: Font: Not Bold, No underline

Formatted: Font: Not Bold, Italic, No

Formatted: Font: Not Bold, No underline

Formatted: Font: Not Bold, Italic, No

Formatted: Font: Not Bold, No underline

Formatted: Font: Not Bold, Italic, No

Formatted: Font: Not Bold, No underline

Formatted: Font: Not Bold, Italic, No

Formatted: Font: Not Bold, No underline

Formatted: Font: Not Bold, Italic, No

Formatted: Font: Not Bold, No underline

Formatted: Font: Not Bold, Italic, No

Formatted: Font: Not Bold, No underline

Formatted: Font: Not Bold, Italic, No

Formatted: Font: Not Bold, No underline

Formatted: Font: Not Bold, Italic, No

Formatted: Font: Not Bold, No underline

Formatted: Font: Not Bold, Italic, No

Formatted: Font: Not Bold, No underline

Formatted: Font: Not Bold, Italic, No

Formatted: Font: Not Bold, No underline

Formatted: Font: Not Bold, Italic, No

Formatted: Font: Not Bold, No underline

Formatted: Font: Not Bold, Italic, No

Formatted: Font: Not Bold, No underline

Formatted: Font: Not Bold, Italic, No

Formatted: Font: Not Bold, No underline

Formatted: Font: Not Bold, Italic, No

For peer review only

21. Hosseini SK, Masoudkabir F, Vasheghani-Farahani A, Alipour-Parsa S, Sheikh Fathollahi M, Rahimi-Foroushani A, et al. Opium consumption and coronary atherosclerosis in diabetic patients: a propensity score-matched study. *Planta Med* 2011;77(17):1870-5.
22. Charney DS, Redmond DE, Jr., Galloway MP, Kleber HD, Heninger GR, Murberg M, et al. Naltrexone precipitated opiate withdrawal in methadone addicted human subjects: evidence for noradrenergic hyperactivity. *Life Sci* 1984;35(12):1263-72.
23. Reece AS, Hulse GK. Elevation of Central Arterial Stiffness and Vascular Ageing in Opiate Withdrawal: Cross-sectional and Longitudinal Studies. *Cardiovasc Toxicol* 2012;13(1):55-67.
24. Chien KR, Karsenty G. Longevity and lineages: toward the integrative biology of degenerative diseases in heart, muscle, and bone. *Cell* 2005;120(4):533-44.
25. Lakatta EG, Wang M, Najjar SS. Arterial aging and subclinical arterial disease are fundamentally intertwined at macroscopic and molecular levels. *Med Clin North Am* 2009;93(3):583-604, Table of Contents.
26. Reece AS. Hair graying in substance addiction. *Archives of dermatology* 2007;143(1):116-8.
27. Kim TW, Alford DP, Malabanan A, Holick MF, Samet JH. Low bone density in patients receiving methadone maintenance treatment. *Drug Alcohol Depend* 2006;85(3):258-62.
28. Reece AS. Evidence of Accelerated Ageing in Clinical Drug Addiction from Immune, Hepatic and Metabolic Biomarkers. *Immun Ageing* 2007;4(1):6-15.
29. Randall D, Degenhardt L, Vajdic CM, Burns L, Hall WD, Law M, et al. Increasing cancer mortality among opioid-dependent persons in Australia: a new public health challenge for a disadvantaged population. *Australian and New Zealand journal of public health* 2011;35(3):220-5.
30. Reece A.S., Hulse G.K. Lifetime Opiate Exposure as an Independent and Interactive Cardiovascular Risk Factor in Males: A Cross-Sectional Clinical Study. *Vascular Health and Risk Management* 2013;9(55):551-59.
31. Reece A.S., Hulse G.K. Opiate Exposure Increases Arterial Stiffness, Advances Vascular Age and is an Independent Cardiovascular Risk Factor in Females: A Cross-Sectional Clinical Study. *World J. Cardiovascular Diseases* 2013;In Press(DOI: 10.4236/wjcd.2013.35056).
32. Reece A. S., Hulse G.K. Duration of Opiate Exposure as a Determinant of Arterial Stiffness and Vascular Age in Male Opiate Dependence: A Longitudinal Study. *Journal of Clinical Pharmacy and Therapeutics* 2013;39(2):158-67.
33. Reece A. S., Hulse G.K. Opiate Dependence as an Independent and Interactive Risk Factor for Arterial Stiffness and Cardiovascular Ageing - A Longitudinal Study in Females. *J Clinical Medical Research* 2013;5(5):356-67.
34. Reece AS. Psychosocial and treatment correlates of opiate free success in a clinical review of a naltrexone implant program. *Subst Abuse Treat Prev Policy* 2007;2:35-49.
35. Breslow N.E., Day N.E. *Statistical Methods in Cancer Research Volume II The Design and Analysis of Cohort Studies*. 1 ed. Lyon: International Agency for Cancer Research., 1987.
36. Tibshirani R. Estimating transformations for regression via additivity and variance stabilization. *Journal of the American Statistical Association* 1988;83(402):394-405.
37. Kacinko SL, Jones HE, Johnson RE, Choo RE, Huestis MA. Correlations of maternal buprenorphine dose, buprenorphine, and metabolite concentrations in meconium with neonatal outcomes. *Clinical pharmacology and therapeutics* 2008;84(5):604-12.
38. Hulse G., White J., Cape G., editors. *Management of Drug and Alcohol Problems*. Sydney: Oxford University Press, 2002.
39. Oviedo-Joekes E, Brissette S, Marsh DC, Lauzon P, Guh D, Anis A, et al. Diacetylmorphine versus methadone for the treatment of opioid addiction. *N Engl J Med* 2009;361(8):777-86.
40. SphygmoCor, editor. *Software Operator's Guide, Pulse Wave Analysis System, SCOR-Px*. Sydney: AtCor Medical, 2006.
41. Klein SL. Sex influences immune responses to viruses, and efficacy of prophylaxis and treatments for viral diseases. *Bioessays* 2012;34(12):1050-9.
42. Sato R, Maesawa C, Fujisawa K, Wada K, Oikawa K, Takikawa Y, et al. Prevention of critical telomere shortening by oestradiol in human normal hepatic cultured cells and carbon tetrachloride induced rat liver fibrosis. *Gut* 2004;53(7):1001-9.

Formatted: Font: Not Bold, Italic, No
Formatted: Font: Not Bold, No underline
Formatted: Font: Not Bold, Italic, No
Formatted: Font: Not Bold, No underline
Formatted: Font: Not Bold, Italic, No
Formatted: Font: Not Bold, No underline
Formatted: Font: Not Bold, Italic, No
Formatted: Font: Not Bold, No underline
Formatted: Font: Not Bold, Italic, No
Formatted: Font: Not Bold, No underline
Formatted: Font: Not Bold, Italic, No
Formatted: Font: Not Bold, No underline
Formatted: Font: Not Bold, Italic, No
Formatted: Font: Not Bold, No underline
Formatted: Font: Not Bold, Italic, No
Formatted: Font: Not Bold, No underline
Formatted: Font: Not Bold, Italic, No
Formatted: Font: Not Bold, No underline
Formatted: Font: Not Bold, Italic, No
Formatted: Font: Not Bold, No underline
Formatted: Font: Not Bold, Italic, No
Formatted: Font: Not Bold, No underline
Formatted: Font: Not Bold, Italic, No
Formatted: Font: Not Bold, No underline
Formatted: Font: Not Bold, Italic, No
Formatted: Font: Not Bold, No underline
Formatted: Font: Not Bold, Italic, No
Formatted: Font: Not Bold, No underline
Formatted: Font: Not Bold, Italic, No
Formatted: Font: Not Bold, No underline
Formatted: Font: Not Bold, Italic, No
Formatted: Font: Not Bold, No underline
Formatted: Font: Not Bold, Italic, No
Formatted: Font: Not Bold, No underline
Formatted: Font: Not Bold, Italic, No
Formatted: Font: Not Bold, No underline
Formatted: Font: Not Bold, Italic, No
Formatted: Font: Not Bold, No underline
Formatted: Font: Not Bold, Italic, No
Formatted: Font: Not Bold, No underline
Formatted: Font: Not Bold, Italic, No
Formatted: Font: Not Bold, No underline
Formatted: Font: Not Bold, Italic, No
Formatted: Font: Not Bold, No underline

For peer review only

43. Reece AS. Hypothalamic opioid-melanocortin appetitive balance and addictive craving. *Medical hypotheses* 2011;76(1):132-7.
44. Cowley MA, Smart JL, Rubinstein M, Cerdan MG, Diano S, Horvath TL, et al. Leptin activates anorexigenic POMC neurons through a neural network in the arcuate nucleus. *Nature* 2001;411(6836):480-4.
45. Nichols W.W., O'Rourke M.F., editors. *McDonald's Blood Flow in Arteries: Theoretical, Experimental and Clinical Principals*. London: Hodder Arnold, 2005.
46. O'Rourke MF, Pauca AL. Augmentation of the aortic and central arterial pressure waveform. *Blood Press Monit* 2004;9(4):179-85.
47. Reece AS. High-sensitivity CRP in opiate addiction: relative and age-dependent elevations. *Cardiovasc Toxicol* 2012;12(2):149-57.
48. Fauci AS, Braunwald E, Kapser DL, Hauser SL, Longo DL, Jameson JL, editors. *Harrison's Principles of Internal Medicine, 17th Edition*. 17th Edition ed. New York: McGraw Hill, 2008.
49. Lakatta EG. Arterial aging is risky. *J Appl Physiol* 2008;105(4):1321-2.
50. Le Couteur DG, Lakatta EG. A vascular theory of aging. *J Gerontol A Biol Sci Med Sci* 2010;65(10):1025-7.
51. Hadley EC, Lakatta EG, Morrison-Bogorad M, Warner HR, Hodes RJ. The future of aging therapies. *Cell* 2005;120(4):557-67.
52. Furumoto K, Inoue E, Nagao N, Hiyama E, Miwa N. Age-dependent telomere shortening is slowed down by enrichment of intracellular vitamin C via suppression of oxidative stress. *Life Sci* 1998;63(11):935-48.
53. von Zglinicki T. Oxidative stress shortens telomeres. *Trends Biochem Sci* 2002;27(7):339-44.
54. von Zglinicki T, Pilger R, Sitte N. Accumulation of single-strand breaks is the major cause of telomere shortening in human fibroblasts. *Free Radic Biol Med* 2000;28(1):64-74.
55. Reece A. S. Chronic Immune Stimulation as a Contributing Cause of Chronic Disease in Opiate Addiction Including Multi-System Ageing *Medical hypotheses* 2010;In Press.
56. Reece AS. Differing age related trajectories of dysfunction in several organ systems in opiate dependence. *Aging Clin Exp Res* 2011;24(1):85-96.
57. Weber T, Auer J, O'Rourke MF, Kvas E, Lassnig E, Berent R, et al. Arterial stiffness, wave reflections, and the risk of coronary artery disease. *Circulation* 2004;109(2):184-9.
58. Williams B, Lacy PS, Thom SM, Cruickshank K, Stanton A, Collier D, et al. Differential impact of blood pressure-lowering drugs on central aortic pressure and clinical outcomes: principal results of the Conduit Artery Function Evaluation (CAFE) study. *Circulation* 2006;113(9):1213-25.
59. Nichols WW. Clinical measurement of arterial stiffness obtained from noninvasive pressure waveforms. *Am J Hypertens* 2005;18(1 Pt 2):3S-10S.
60. Degenhardt L, Larney S, Randall D, Burns L, Hall W. Causes of death in a cohort treated for opioid dependence between 1985 and 2005. *Addiction* 2013.
61. Reece A. S. Manifold Implications of Forgotten Hyperglycaemia in Clinical Opiate Dependence. *Drug and Chemical Toxicology* 2012.
62. Reece AS. Relative and Age-dependent Stimulation of Soluble and Cellular Immunity in Opiate Dependence. *J Addict Med* 2012;6(1):10-7.
63. Reece AS. Differing age related trajectories of dysfunction in several organ systems in opiate dependence. *Aging Clin Exp Res* 2012;24(1):85-96.
64. Reece AS. Dentition of addiction in Queensland: poor dental status and major contributing drugs. *Australian dental journal* 2007;52(2):144-9.
65. Reece A. Lifetime prevalence of cervical neoplasia in addicted and medical patients. *Aust N Z J Obstet Gynaecol* 2007;47(5):419-23.
66. Tatia M.C. Lee, Gordon L.F. Cheng, Hung Zeng, Meikei Leung, Hui-Jun Zhang, Benson W.M. Lau, et al. Heroin abuse accelerates biological aging: A novel insight from telomerase and brain imaging interaction. *Translational Psychiatry* 2013.
67. Cheng F, McLaughlin PJ, Verderame MF, Zagon IS. The OGF-OGFr axis utilizes the p16INK4a and p21WAF1/CIP1 pathways to restrict normal cell proliferation. *Molecular biology of the cell* 2009;20(1):319-27.

Formatted ... [1]
Formatted ... [2]
Formatted ... [3]
Formatted ... [4]
Formatted ... [5]
Formatted ... [6]
Formatted ... [7]
Formatted ... [8]
Formatted ... [9]
Formatted ... [10]
Formatted ... [11]
Formatted ... [12]
Formatted ... [13]
Formatted ... [14]
Formatted ... [15]
Formatted ... [16]
Formatted ... [17]
Formatted ... [18]
Formatted ... [19]
Formatted ... [20]
Formatted ... [21]
Formatted ... [22]
Formatted ... [23]
Formatted ... [24]
Formatted ... [25]
Formatted ... [26]
Formatted ... [27]
Formatted ... [28]
Formatted ... [29]
Formatted ... [30]
Formatted ... [31]
Formatted ... [32]
Formatted ... [33]
Formatted ... [34]
Formatted ... [35]
Formatted ... [36]
Formatted ... [37]
Formatted ... [38]
Formatted ... [39]
Formatted ... [40]
Formatted ... [41]
Formatted ... [42]
Formatted ... [43]
Formatted ... [44]
Formatted ... [45]
Formatted ... [46]
Formatted ... [47]
Formatted ... [48]
Formatted ... [49]
Formatted ... [50]

For peer review only

68. Zagon IS, Verderame MF, McLaughlin PJ. The biology of the opioid growth factor receptor (OGFr). *Brain research* 2002;38(3):351-76.
69. Pasmant E, Sabbagh A, Vidaud M, Bieche I. ANRIL, a long, noncoding RNA, is an unexpected major hotspot in GWAS. *FASEB J* 2010;25(2):444-48.
70. Hutchinson MR, Shavit Y, Grace PM, Rice KC, Maier SF, Watkins LR. Exploring the neuroimmunopharmacology of opioids: an integrative review of mechanisms of central immune signaling and their implications for opioid analgesia. *Pharmacological reviews* 2011;63(3):772-810.
71. Starr ME, Hu Y, Stromberg AJ, Carmical JR, Wood TG, Evers BM, et al. Gene expression profile of mouse white adipose tissue during inflammatory stress: age-dependent upregulation of major pro-coagulant factors. *Aging Cell* 2012:n/a-n/a.
72. Coller JK, Hutchinson MR. Implications of central immune signaling caused by drugs of abuse: mechanisms, mediators and new therapeutic approaches for prediction and treatment of drug dependence. *Pharmacol Ther* 2012;134(2):219-45.
73. Grzanna R., Brown R.M. NIDA Research Monograph 125: Activation of Intermediate Early Genes by Drugs of Abuse. In: National Institutes of Drug Abuse, editor. Bethesda, Maryland: NIH, 1993:1-220.
74. Kirkwood TB. Understanding the odd science of aging. *Cell* 2005;120(4):437-47.
75. Hoing S, Rudhard Y, Reinhardt P, Glatza M, Stehling M, Wu G, et al. Discovery of inhibitors of microglial neurotoxicity acting through multiple mechanisms using a stem-cell-based phenotypic assay. *Cell Stem Cell* 2012;11(5):620-32.
76. Zhang G, Li J, Purkayastha S, Tang Y, Zhang H, Yin Y, et al. Hypothalamic programming of systemic ageing involving IKK-[bgr], NF-[kgr]B and GnRH. *Nature* 2013;497(7448):211-16.
77. Li J, Tang Y, Cai D. IKKbeta/NF-kappaB disrupts adult hypothalamic neural stem cells to mediate a neurodegenerative mechanism of dietary obesity and pre-diabetes. *Nat Cell Biol* 2012;14(10):999-1012.
78. Purkayastha S, Zhang G, Cai D. Uncoupling the mechanisms of obesity and hypertension by targeting hypothalamic IKK-beta and NF-kappaB. *Nat Med* 2011;17(7):883-7.
79. Thaler JP, Yi CX, Schur EA, Guyenet SJ, Hwang BH, Dietrich MO, et al. Obesity is associated with hypothalamic injury in rodents and humans. *J Clin Invest* 2012;122(1):153-62.
80. Czapski GA, Gajkowska B, Strosznajder JB. Systemic administration of lipopolysaccharide induces molecular and morphological alterations in the hippocampus. *Brain Res* 2010;1356:85-94.
81. Wang X, Loram LC, Ramos K, de Jesus AJ, Thomas J, Cheng K, et al. Morphine activates neuroinflammation in a manner parallel to endotoxin. *Proc Natl Acad Sci U S A* 2012;109(16):6325-30.
82. Billes SK, Greenway FL. Combination therapy with naltrexone and bupropion for obesity. *Expert opinion on pharmacotherapy* 2011;12(11):1813-26.
83. Neal MD, Jia H, Eyer B, Good M, Guerriero CJ, Sodhi CP, et al. Discovery and validation of a new class of small molecule Toll-like receptor 4 (TLR4) inhibitors. *PLoS ONE* 2013;8(6):e65779.
84. Theberge FR, Li X, Kambhampati S, Pickens CL, St Laurent R, Bossert JM, et al. Effect of chronic delivery of the Toll-like receptor 4 antagonist (+)-naltrexone on incubation of heroin craving. *Biol Psychiatry* 2013;73(8):729-37.
85. Kim CG, Chung IY, Lim Y, Lee YH, Shin SY. A Tcf/Lef element within the enhancer region of the human NANOG gene plays a role in promoter activation. *Biochem Biophys Res Commun* 2011;410(3):637-42.
86. Yang HM, Do HJ, Oh JH, Kim JH, Choi SY, Cha KY, et al. Characterization of putative cis-regulatory elements that control the transcriptional activity of the human Oct4 promoter. *J Cell Biochem* 2005;96(4):821-30.
87. Folmes CD, Dzeja PP, Nelson TJ, Terzic A. Metabolic plasticity in stem cell homeostasis and differentiation. *Cell Stem Cell* 2012;11(5):596-606.
88. Zurolo E, Iyer A, Maroso M, Carbonell C, Anink JJ, Ravizza T, et al. Activation of Toll-like receptor, RAGE and HMGB1 signalling in malformations of cortical development. *Brain* 2011;134(Pt 4):1015-32.

Formatted: Font: Not Bold, Italic, No
Formatted: Font: Not Bold, No underline
Formatted: Font: Not Bold, Italic, No
Formatted: Font: Not Bold, No underline
Formatted: Font: Not Bold, Italic, No
Formatted: Font: Not Bold, No underline
Formatted: Font: Not Bold, Italic, No
Formatted: Font: Not Bold, No underline
Formatted: Font: Not Bold, Italic, No
Formatted: Font: Not Bold, No underline
Formatted: Font: Not Bold, Italic, No
Formatted: Font: Not Bold, No underline
Formatted: Font: Not Bold, Italic, No
Formatted: Font: Not Bold, No underline
Formatted: Font: Not Bold, Italic, No
Formatted: Font: Not Bold, No underline
Formatted: Font: Not Bold, Italic, No
Formatted: Font: Not Bold, No underline
Formatted: Font: Not Bold, Italic, No
Formatted: Font: Not Bold, No underline
Formatted: Font: Not Bold, Italic, No
Formatted: Font: Not Bold, No underline
Formatted: Font: Not Bold, Italic, No
Formatted: Font: Not Bold, No underline
Formatted: Font: Not Bold, Italic, No
Formatted: Font: Not Bold, No underline
Formatted: Font: Not Bold, Italic, No
Formatted: Font: Not Bold, No underline
Formatted: Font: Not Bold, Italic, No
Formatted: Font: Not Bold, No underline
Formatted: Font: Not Bold, Italic, No
Formatted: Font: Not Bold, No underline
Formatted: Font: Not Bold, Italic, No
Formatted: Font: Not Bold, No underline
Formatted: Font: Not Bold, Italic, No
Formatted: Font: Not Bold, No underline
Formatted: Font: Not Bold, Italic, No
Formatted: Font: Not Bold, No underline
Formatted: Font: Not Bold, Italic, No
Formatted: Font: Not Bold, No underline
Formatted: Font: Not Bold, Italic, No
Formatted: Font: Not Bold, No underline

89. Nogueira-Machado JA, Volpe CM, Veloso CA, Chaves MM. HMGB1, TLR and RAGE: a functional tripod that leads to diabetic inflammation. *Expert opinion on therapeutic targets* 2011;15(8):1023-35.
90. Sakaguchi M, Murata H, Yamamoto K, Ono T, Sakaguchi Y, Motoyama A, et al. TIRAP, an adaptor protein for TLR2/4, transduces a signal from RAGE phosphorylated upon ligand binding. *PLoS ONE* 2011;6(8):e23132.
91. Veloso CA, Fernandes JS, Volpe CM, Fagundes-Netto FS, Reis JS, Chaves MM, et al. TLR4 and RAGE: similar routes leading to inflammation in type 2 diabetic patients. *Diabetes Metab* 2011;37(4):336-42.

Formatted: Font: Not Bold, Italic, No
Formatted: Font: Not Bold, No underline
Formatted: Font: Not Bold, Italic, No
Formatted: Font: Not Bold, No underline
Formatted: Font: Not Bold, Italic, No
Formatted: Font: Not Bold, No underline
Formatted: Left, Indent: Left: 0", Hanging: 0.5"

~~References~~

~~1. Rosen D, Smith ML, Reynolds CF, 3rd. The prevalence of mental and physical health disorders among older methadone patients. *Am J Geriatr Psychiatry* 2008;16(6):488-97.~~
~~2. Cooper OB, Brown TT, Dobs AS. Opiate drug use: a potential contributor to the endocrine and metabolic complications in human immunodeficiency virus disease. *Clin Infect Dis* 2003;37 Suppl 2:S132-6.~~
~~3. Ceriello A, Quatraro A, Giugliano D. Opiate addict as diabetic patient? *Diabetes Care* 1988;11(5):443.~~
~~4. Kolarzyk E, Pach D, Wojtowicz B, Szpanowska-Wohn A, Szurkowska M. Nutritional status of the opiate dependent persons after 4 years of methadone maintenance treatment. *Przeglad lekarski* 2005;62(6):373-7.~~
~~5. Brunton LL, Lazo JS, Parker KL, editors. *Goodman and Gilman's the Pharmacologic Basis of Therapeutics*. Eleventh Edition ed. New York: McGraw Hill, 2006.~~
~~6. Reece A. S. Relative and Age Dependent Stimulation of Soluble and Cellular Immunity in Opiate Dependence. *Journal of Addiction Medicine* 2012;6(1):10-17.~~
~~7. Reece A. S. High Sensitivity CRP in Opiate Addiction: Relative and Age-Dependent Elevations. *Cardiovascular Toxicology* 2012;12(2):149-57.~~
~~8. Neri S, Bruno CM, Pulvirenti D, Malaguarnera M, Italiano C, Mauceri B, et al. Randomized clinical trial to compare the effects of methadone and buprenorphine on the immune system in drug abusers. *Psychopharmacology (Berl)* 2005;179(3):700-4.~~
~~9. Reece A. S. Absolute and Age Dependent Elevations of Serum Calcium and Phosphate and their Products in Clinical Opiate Dependence. *J. Subs. Abuse* 2013;Accepted 27th November 2012.~~
~~10. Reece AS, Davidson P. Deficit of circulating stem--progenitor cells in opiate addiction: a pilot study. *Substance abuse treatment, prevention, and policy* 2007;2:19-28.~~
~~11. Darke S, Duflou J, Torok M. The comparative toxicology and major organ pathology of fatal methadone and heroin toxicity cases. *Drug Alcohol Depend* 2010;106(1):1-6.~~
~~12. Darke S, Kaye S, Duflou J. Systemic disease among cases of fatal opioid toxicity. *Addiction* 2006;101(9):1299-305.~~
~~13. Degenhardt L., Randall D., Hall W., Law M., Butler T., Burns L. Mortality among clients of a state-wide opioid pharmacotherapy program over 20 years: Risk factors and lives saved. *Drug Alcohol Depend* 2009;105(1-2):9-15.~~
~~14. Khademi H, Malekzadeh R, Pourshams A, Jafari E, Salahi R, Semnani S, et al. Opium use and mortality in Golestan Cohort Study: prospective cohort study of 50,000 adults in Iran. *BMJ (Clinical research ed* 2012;344:e2502.~~
~~15. Sadeghian S, Darvish S, Davoodi G, Salarifar M, Mahmoodian M, Fallah N, et al. The association of opium with coronary artery disease. *Eur J Cardiovasc Prev Rehabil* 2007;14(5):715-7.~~
~~16. Sadeghian S, Dowlatshahi S, Karimi A, Tazik M. Epidemiology of opium use in 4398 patients admitted for coronary artery bypass graft in Tehran Heart Center. *J Cardiovasc Surg (Torino)* 2011;52(1):140-1.~~
~~17. Sadeghian S, Graili P, Salarifar M, Karimi AA, Darvish S, Abbasi SH. Opium consumption in men and diabetes mellitus in women are the most important risk factors of premature coronary artery disease in Iran. *International journal of cardiology* 2010;141(1):116-8.~~

For peer review only

18. Smyth B, Hoffman V, Fan J, Hser YI. Years of potential life lost among heroin addicts 33 years after treatment. *Preventive medicine* 2007;44(4):369-74.
19. Reece A.S., Hulse G.K. Impact of Opioid Pharmacotherapy on Arterial stiffness and Vascular Ageing: Cross-sectional and Longitudinal Studies. *Cardiovascular Toxicology* 2013;13(3):254-66.
20. Reece A. S., Hulse G.K. Reduction in Arterial Stiffness and Vascular Age by Naltrexone-Induced Interruption of Opiate Agonism. *British Medical Journal - Open* 2013.
21. Hosseini SK, Masoudkabir F, Vasheghani-Farahani A, Alipour-Parsa S, Sheikh Fathollahi M, Rahimi-Foroushani A, et al. Opium consumption and coronary atherosclerosis in diabetic patients: a propensity score-matched study. *Planta Med* 2011;77(17):1870-5.
22. Charney DS, Redmond DE, Jr., Galloway MP, Kleber HD, Heninger GR, Murberg M, et al. Naltrexone precipitated opiate withdrawal in methadone addicted human subjects: evidence for noradrenergic hyperactivity. *Life Sci* 1984;35(12):1263-72.
23. Reece AS, Hulse GK. Elevation of Central Arterial Stiffness and Vascular Ageing in Opiate Withdrawal: Cross-sectional and Longitudinal Studies. *Cardiovasc Toxicol* 2012;13(1):55-67.
24. Chien KR, Karsenty G. Longevity and lineages: toward the integrative biology of degenerative diseases in heart, muscle, and bone. *Cell* 2005;120(4):533-44.
25. Lakatta EG, Wang M, Najjar SS. Arterial aging and subclinical arterial disease are fundamentally intertwined at macroscopic and molecular levels. *Med Clin North Am* 2009;93(3):583-604, Table of Contents.
26. Reece AS. Hair graying in substance addiction. *Archives of dermatology* 2007;143(1):116-8.
27. Kim TW, Alford DP, Malabanan A, Holick MF, Samet JH. Low bone density in patients receiving methadone maintenance treatment. *Drug Alcohol Depend* 2006;85(3):258-62.
28. Reece AS. Evidence of Accelerated Ageing in Clinical Drug Addiction from Immune, Hepatic and Metabolic Biomarkers. *Immun Ageing* 2007;4(1):6-15.
29. Randall D, Degenhardt L, Vajdic CM, Burns L, Hall WD, Law M, et al. Increasing cancer mortality among opioid-dependent persons in Australia: a new public health challenge for a disadvantaged population. *Australian and New Zealand journal of public health* 2011;35(3):220-5.
30. Reece A.S., Hulse G.K. Lifetime Opiate Exposure as an Independent and Interactive Cardiovascular Risk Factor in Males: A Cross-Sectional Clinical Study. *Vascular Health and Risk Management* 2013;9(55):551-59.
31. Reece A.S., Hulse G.K. Opiate Exposure Increases Arterial Stiffness, Advances Vascular Age and is an Independent Cardiovascular Risk Factor in Females: A Cross-Sectional Clinical Study. *World J. Cardiovascular Diseases* 2013;In Press(DOI: 10.4236/wjcd.2013.35056).
32. Reece A. S., Hulse G.K. Duration of Opiate Exposure as a Determinant of Arterial Stiffness and Vascular Age in Male Opiate Dependence: A Longitudinal Study. *Journal of Clinical Pharmacy and Therapeutics* 2013;39(2):158-67.
33. Reece A. S., Hulse G.K. Opiate Dependence as an Independent and Interactive Risk Factor for Arterial Stiffness and Cardiovascular Ageing - A Longitudinal Study in Females. *J Clinical Medical Research* 2013;5(5):356-67.
34. Reece AS. Psychosocial and treatment correlates of opiate free success in a clinical review of a naltrexone implant program. *Subst Abuse Treat Prev Policy* 2007;2:35-49.
35. Breslow N.E., Day N.E. *Statistical Methods in Cancer Research Volume II -The Design and Analysis of Cohort Studies*. 1 ed. Lyon: International Agency for Cancer Research,, 1987.
36. Kacinko SL, Jones HE, Johnson RE, Choo RE, Huestis MA. Correlations of maternal buprenorphine dose, buprenorphine, and metabolite concentrations in meconium with neonatal outcomes. *Clinical pharmacology and therapeutics* 2008;84(5):604-12.
37. Hulse G., White J., Cape G., editors. *Management of Drug and Alcohol Problems*. Sydney: Oxford University Press, 2002.
38. Oviedo-Joekes E, Brissette S, Marsh DC, Lauzon P, Guh D, Anis A, et al. Diacetylmorphine versus methadone for the treatment of opioid addiction. *N Engl J Med* 2009;361(8):777-86.
39. SphygmoCor, editor. *Software Operator's Guide, Pulse Wave Analysis System, SCOR-Px*. Sydney: AtCor Medical, 2006.

For peer review only

40. Klein SL. Sex influences immune responses to viruses, and efficacy of prophylaxis and treatments for viral diseases. *Bioessays* 2012;34(12):1050-9.
41. Reece AS. Hypothalamic opioid-melanocortin appetitive balance and addictive craving. *Medical hypotheses* 2011;76(1):132-7.
42. Cowley MA, Smart JL, Rubinstein M, Cerdan MG, Diano S, Horvath TL, et al. Leptin activates anorexigenic POMC neurons through a neural network in the arcuate nucleus. *Nature* 2001;411(6836):480-4.
43. Nichols W.W., O'Rourke M.F., editors. *McDonald's Blood Flow in Arteries: Theoretical, Experimental and Clinical Principals*. London: Hodder Arnold, 2005.
44. O'Rourke MF, Pauca AL. Augmentation of the aortic and central arterial pressure waveform. *Blood Press Monit* 2004;9(4):179-85.
45. Reece AS. High-sensitivity CRP in opiate addiction: relative and age-dependent elevations. *Cardiovasc Toxicol* 2012;12(2):149-57.
46. Fauci AS, Braunwald E, Kapser DL, Hauser SL, Longo DL, Jameson JL, editors. *Harrison's Principles of Internal Medicine, 17th Edition*. 17th Edition ed. New York: McGraw Hill, 2008.
47. Lakatta EG. Arterial aging is risky. *J Appl Physiol* 2008;105(4):1321-2.
48. Le Couteur DG, Lakatta EG. A vascular theory of aging. *J Gerontol A Biol Sci Med Sci* 2010;65(10):1025-7.
49. Hadley EC, Lakatta EG, Morrison-Bogorad M, Warner HR, Hodes RJ. The future of aging therapies. *Cell* 2005;120(4):557-67.
50. Weber T, Auer J, O'Rourke MF, Kvas E, Lassnig E, Berent R, et al. Arterial stiffness, wave reflections, and the risk of coronary artery disease. *Circulation* 2004;109(2):184-9.
51. Williams B, Lacy PS, Thom SM, Cruickshank K, Stanton A, Collier D, et al. Differential impact of blood pressure-lowering drugs on central aortic pressure and clinical outcomes: principal results of the Conduit Artery Function Evaluation (CAFE) study. *Circulation* 2006;113(9):1213-25.
52. Nichols WW. Clinical measurement of arterial stiffness obtained from noninvasive pressure waveforms. *Am J Hypertens* 2005;18(1 Pt 2):3S-10S.
53. Degenhardt L, Larney S, Randall D, Burns L, Hall W. Causes of death in a cohort treated for opioid dependence between 1985 and 2005. *Addiction* 2013.
54. Reece A. S. Manifold Implications of Forgotten Hyperglycaemia in Clinical Opiate Dependence. *Drug and Chemical Toxicology* 2012.
55. Reece AS. Relative and Age-dependent Stimulation of Soluble and Cellular Immunity in Opiate Dependence. *J Addict Med* 2012;6(1):10-7.
56. Reece AS. Differing age related trajectories of dysfunction in several organ systems in opiate dependence. *Aging Clin Exp Res* 2012;24(1):85-96.
57. Reece AS. Dentition of addiction in Queensland: poor dental status and major contributing drugs. *Australian dental journal* 2007;52(2):144-9.
58. Reece A. Lifetime prevalence of cervical neoplasia in addicted and medical patients. *Aust N Z J Obstet Gynaecol* 2007;47(5):419-23.
59. Tatia M.C. Lee, Gordon L.F. Cheng, Hung Zeng, Meikei Leung, Hui-Jun Zhang, Benson W.M. Lau, et al. Heroin abuse accelerates biological aging: A novel insight from telomerase and brain imaging interaction. *Translational Psychiatry* 2013.
60. Cheng F, McLaughlin PJ, Verderame MF, Zagon IS. The OGF-OGFr axis utilizes the p16INK4a and p21WAF1/CIP1 pathways to restrict normal cell proliferation. *Molecular biology of the cell* 2009;20(1):319-27.
61. Zagon IS, Verderame MF, McLaughlin PJ. The biology of the opioid growth factor receptor (OGFr). *Brain research* 2002;38(3):351-76.
62. Pasmant E, Sabbagh A, Vidaud M, Bieche I. ANRIL, a long, noncoding RNA, is an unexpected major hotspot in GWAS. *FASEB J* 2010;25(2):444-48.
63. Hutchinson MR, Shavit Y, Grace PM, Rice KC, Maier SF, Watkins LR. Exploring the neuroimmunopharmacology of opioids: an integrative review of mechanisms of central immune signaling and their implications for opioid analgesia. *Pharmacological reviews* 2011;63(3):772-810.

For peer review only

64. Starr ME, Hu Y, Stromberg AJ, Carmical JR, Wood TG, Evers BM, et al. Gene expression profile of mouse white adipose tissue during inflammatory stress: age-dependent upregulation of major pro-coagulant factors. *Aging Cell* 2012:n/a-n/a.
65. Coller JK, Hutchinson MR. Implications of central immune signaling caused by drugs of abuse: mechanisms, mediators and new therapeutic approaches for prediction and treatment of drug dependence. *Pharmacol Ther* 2012;134(2):219-45.
66. Grzanna R., Brown R.M. NIDA Research Monograph 125: Activation of Intermediate Early Genes by Drugs of Abuse. In: National Institutes of Drug Abuse, editor. Bethesda, Maryland: NIH, 1993:1-220.
67. Kirkwood TB. Understanding the odd science of aging. *Cell* 2005;120(4):437-47.
68. Hoing S, Rudhard Y, Reinhardt P, Glatza M, Stehling M, Wu G, et al. Discovery of inhibitors of microglial neurotoxicity acting through multiple mechanisms using a stem-cell-based phenotypic assay. *Cell Stem Cell* 2012;11(5):620-32.
69. Zhang G, Li J, Purkayastha S, Tang Y, Zhang H, Yin Y, et al. Hypothalamic programming of systemic ageing involving IKK-[bgr], NF-[kgr]B and GnRH. *Nature* 2013;497(7448):211-16.
70. Li J, Tang Y, Cai D. IKKbeta/NF-kappaB disrupts adult hypothalamic neural stem cells to mediate a neurodegenerative mechanism of dietary obesity and pre-diabetes. *Nat Cell Biol* 2012;14(10):999-1012.
71. Purkayastha S, Zhang G, Cai D. Uncoupling the mechanisms of obesity and hypertension by targeting hypothalamic IKK-beta and NF-kappaB. *Nat Med* 2011;17(7):883-7.
72. Thaler JP, Yi CX, Schur EA, Guyenet SJ, Hwang BH, Dietrich MO, et al. Obesity is associated with hypothalamic injury in rodents and humans. *J Clin Invest* 2012;122(1):153-62.
73. Czapski GA, Gajkowska B, Strosznajder JB. Systemic administration of lipopolysaccharide induces molecular and morphological alterations in the hippocampus. *Brain Res* 2010;1356:85-94.
74. Wang X, Loram LC, Ramos K, de Jesus AJ, Thomas J, Cheng K, et al. Morphine activates neuroinflammation in a manner parallel to endotoxin. *Proc Natl Acad Sci U S A* 2012;109(16):6325-30.
75. Billes SK, Greenway FL. Combination therapy with naltrexone and bupropion for obesity. *Expert opinion on pharmacotherapy* 2011;12(11):1813-26.
76. Neal MD, Jia H, Eyer B, Good M, Guerriero CJ, Sodhi CP, et al. Discovery and validation of a new class of small molecule Toll-like receptor 4 (TLR4) inhibitors. *PLoS ONE* 2013;8(6):e65779.
77. Theberge FR, Li X, Kambhampati S, Pickens CL, St Laurent R, Bossert JM, et al. Effect of chronic delivery of the Toll-like receptor 4 antagonist (+)-naltrexone on incubation of heroin craving. *Biol Psychiatry* 2013;73(8):729-37.
78. Kim CG, Chung IY, Lim Y, Lee YH, Shin SY. A Tcf/Lef element within the enhancer region of the human NANOG gene plays a role in promoter activation. *Biochem Biophys Res Commun* 2011;410(3):637-42.
79. Yang HM, Do HJ, Oh JH, Kim JH, Choi SY, Cha KY, et al. Characterization of putative cis-regulatory elements that control the transcriptional activity of the human Oct4 promoter. *J Cell Biochem* 2005;96(4):821-30.
80. Folmes CD, Dzeja PP, Nelson TJ, Terzic A. Metabolic plasticity in stem cell homeostasis and differentiation. *Cell Stem Cell* 2012;11(5):596-606.
81. Zurolo E, Iyer A, Maroso M, Carbonell C, Anink JJ, Ravizza T, et al. Activation of Toll-like receptor, RAGE and HMGB1 signalling in malformations of cortical development. *Brain* 2011;134(Pt 4):1015-32.
82. Nogueira-Machado JA, Volpe CM, Veloso CA, Chaves MM. HMGB1, TLR and RAGE: a functional tripod that leads to diabetic inflammation. *Expert opinion on therapeutic targets* 2011;15(8):1023-35.
83. Sakaguchi M, Murata H, Yamamoto K, Ono T, Sakaguchi Y, Motoyama A, et al. TIRAP, an adaptor protein for TLR2/4, transduces a signal from RAGE phosphorylated upon ligand binding. *PLoS ONE* 2011;6(8):e23132.

For peer review only

84. Veloso CA, Fernandes JS, Volpe CM, Fagundes-Netto FS, Reis JS, Chaves MM, et al. TLR4 and RAGE: similar routes leading to inflammation in type 2 diabetic patients. *Diabetes Metab* 2011;37(4):336-42.

For peer review only

Figure Captions:

Figure 1: Ageing and Stiffness Indices by Chronologic Age by Opioid Dependency Status
RA – Vascular Reference Age, CA – Chronologic Age, C_AP_HR75 – central augmentation pressure at Heart Rate 75 bpm, C_AGPH_HR75 – Central Augmentation Pressure / Pulse Height Ratio at Heart Rate 75 bpm.

Figure 2: Corrected Ageing and Stiffness Indices by Time by Opioid Dependency Status
RA – Vascular Reference Age, CA – Chronologic Age, C_AP_HR75 – central augmentation pressure at Heart Rate 75 bpm, C_AGPH_HR75 – Central Augmentation Pressure / Pulse Height Ratio at Heart Rate 75 bpm.

~~Figure 3: Ageing Indices by Chronologic Age by Addictive Status by Sex~~

~~Figure 4: Ageing Indices by Chronologic Age by Sex by Addictive Status~~

Figure ~~5~~3: BMI-Related Ageing Indices by Chronologic Age by Addictive Status by Sex
RA – Vascular Reference Age, CA – Chronologic Age, C_AP_HR75 – central augmentation pressure at Heart Rate 75 bpm, C_AGPH_HR75 – Central Augmentation Pressure / Pulse Height Ratio at Heart Rate 75 bpm.

Figure ~~4~~6: BMI-Related Ageing Indices by Chronologic Age by Sex by Addictive Status
RA – Vascular Reference Age, CA – Chronologic Age, C_AP_HR75 – central augmentation pressure at Heart Rate 75 bpm, C_AGPH_HR75 – Central Augmentation Pressure / Pulse Height Ratio at Heart Rate 75 bpm.

Figure ~~7~~5: Ageing Indices by Chronologic Age by Sex by Addictive Status – Loess Curves
RA – Vascular Reference Age, CA – Chronologic Age.

Figure ~~8~~6: Vascular Age Indices by Chronologic Age by Opioid Dependency ~~Level, Loess curves~~Quartile
RA – Vascular Reference Age, CA – Chronologic Age. Opioid Exposure Quartiles as Defined in Methods section.

Supplementary Figure Captions:

Suppl. Figure 1~~3~~: Ageing Indices by Chronologic Age by Addictive Status by Sex
RA – Vascular Reference Age, CA – Chronologic Age, C_AP_HR75 – central augmentation pressure at Heart Rate 75 bpm, C_AGPH_HR75 – Central Augmentation Pressure / Pulse Height Ratio at Heart Rate 75 bpm.

For peer review only

Suppl. Figure 24: Ageing Indices by Chronologic Age by Sex by Addictive Status
RA – Vascular Reference Age, CA – Chronologic Age.

Suppl. Fig. 31: Vascular Age Indices by Time by Opioid Dependency Level
RA – Vascular Reference Age, CA – Chronologic Age.

For peer review only

TABLES

For peer review only

Table 1.: Effects of Gender on Key Measures of Arterial Stiffness - Cross-sectional Dataset

Dependent Variable	Group	Parameter	Adj. R^2	F	dF	Model P	Estimate	Std. Error	t-Value	P
-	-	-	-	-	-	-	-	-	-	-
RA-CA Difference	Opioid	Chron.Age :Female.Sex	0.0189	7.596	2, 684	0.0005459	0.6324	0.3469	1.823	0.0688
BMI	Control	Chron.Age :Female.Sex	0.1523	52.58	2, 572	<2.2E-16	-0.0092	0.0040	-2.310	0.0213
BMI	Opioid	Female.Sex	0.0713	27.35	2, 684	3.75E-12	-0.0827	0.0139	-5.940	4.6E-09
RA/BMI	Opioid	Chron.Age :Female.Sex	0.2617	122.6	2, 684	<2.2E-16	0.0361	0.0091	3.948	8.7E-05
C_AP_HR75/BMI	Control	Chron.Age :Female.Sex	0.4882	274.3	2, 571	<2.2E-16	0.0312	0.0040	7.737	4.6E-14
C_AP_HR75/BMI	Control	Chron.Age :Female.Sex	0.4882	274.3	2, 571	<2.2E-16	0.0312	0.0040	7.737	4.6E-14
C_AP_HR75/BMI	Opioid	Chron.Age :Female.Sex	0.3712	203.4	2, 684	<2.2E-16	0.0467	0.0039	11.950	<2e-16
C_AGPH_HR75/BMI	Control	Chron.Age :Female.Sex	0.4603	245.4	2, 571	<2.2E-16	0.0924	0.0112	8.283	8.6E-16
C_AGPH_HR75/BMI	Opioid	Chron.Age :Female.Sex	0.3980	227.8	2, 684	<2.2E-16	0.1265	0.0095	13.370	<2e-16
C_AP_HR75/(CA*BMI)	Control	Female.Sex	0.2809	112.9	2, 571	<2.2E-16	0.0033	0.0005	6.286	6.5E-10
C_AP_HR75/(CA*BMI)	Opioid	Female.Sex	0.2489	114.7	2, 684	<2.2E-16	0.0047	0.0004	11.428	<2e-16
C_AGPH_HR75/(CA*BMI)	Control	Female.Sex	0.2567	99.96	2, 571	<2.2E-16	0.0096	0.0015	6.350	4.4E-10
C_AGPH_HR75/(CA*BMI)	Opioid	Female.Sex	0.2604	121.8	2, 684	<2.2E-16	0.0130	0.0011	12.307	<2e-16

Formatted: Centered

Formatted: Centered

Formatted: Centered

Formatted: Centered

Formatted: Centered

Formatted: Centered

Formatted: Centered

Formatted: Centered

Formatted: Centered

Formatted: Centered

Formatted: Centered

Formatted: Centered

Formatted: Centered

Formatted: Centered

Formatted: Centered

Formatted: Centered

Table 2.:

Age: Addictive Status Relationships with CA Discretized

About 41 Years in Males and 29 Years in Females

With Females <55 Years of Age

Parameter	Variable Statistics				Model Summaries		
-	Estimate	Std. Error	t Value	P	F	dF	P
-	-	-	-	-	-	-	-
Males	-	-	-	-	-	-	-
Older Age	-0.1837	0.0557	-3.296	0.0010	5.492	3, 862	0.00097
Opioids	-0.0846	0.0275	-3.082	0.0021	-	-	-
Older Age: Opioids	0.2310	0.0687	3.364	0.0008	-	-	-
-	-	-	-	-	-	-	-
Females	-	-	-	-	-	-	-
Older Age: Opioids	0.1248	0.0532	2.346	0.0195	2.169	3, 378	0.09123

Formatted: Centered

Formatted: Centered

Formatted: Centered

Formatted: Centered

Formatted: Centered

Formatted: Centered

Formatted: Centered

Formatted: Centered

Formatted: Centered

Formatted: Centered

Formatted: Centered

Formatted: Centered

Formatted: Centered

For peer review only

Table 3.: Cross-sectional Models Polynomial in Heroin Duration

-	Parameter				Model		
Model / Parameter	Value	Std.Error	t-value	P-value	F	dF	P
-	-	-	-	-	-	-	-
1 Order- Linear	-	-	-	-	-	-	-
H.Dura.n	-0.0059	0.0021	-2.779	0.0056	8.628	2, 664	0.0002
H.Dose: H.Dura.n	0.0091	0.0022	4.152	3.7.E-05	-	-	-
-	-	-	-	-	-	-	-
2 Order- Quadratic	-	-	-	-	-	-	-
H.Dura.n	-0.0171	0.0055	-3.136	0.0018	7.443	3, 663	6.60E-05
H.Dura.n^2	0.0004	0.0002	2.229	0.0261	-	-	-
H.Dose: H.Dura.n	0.0084	0.0022	3.802	0.0002	-	-	-
-	-	-	-	-	-	-	-
3 Order - Cubic	-	-	-	-	-	-	-
H.Dura.n	-0.0116	0.0035	-3.263	0.0012	7.102	3 663	0.00011
H.Dura.n^3	6.3E-06	3.1E-06	1.993	0.0466	-	-	-
H.Dose: H.Dura.n	0.0084	0.0022	3.836	0.0001	-	-	-
-	-	-	-	-	-	-	-
4 Order- Quartic	-	-	-	-	-	-	-
H.Dura.n	-0.0059	0.0021	-2.779	0.0056	8.628	2, 664	0.0002
H.Dose: H.Dura.n	0.0091	0.0022	4.152	3.7.E-05	-	-	-
-	-	-	-	-	-	-	-
1, 2, 3 & 4 Order	-	-	-	-	-	-	-
H.Dose: H.Dura.n^3	6.5E-05	2.0E-05	3.252	0.0012	3.353	7, 659	0.0016
H.Dura.n^2 : H.Dura.n^3	-1.0E-06	3.1E-07	-3.398	0.0007	-	-	-
H.Dura.n^2 : H.Dura.n^4	9.2E-08	2.9E-08	3.216	0.0014	-	-	-
H.Dura.n^3: H.Dura.n^4	-2.7E-09	8.8E-10	-3.029	0.0026	-	-	-
H.Dose: H.Dura.n^2 : H.Dura.n^4	-4.5E-09	1.8E-09	-2.452	0.0145	-	-	-
H.Dura.n: H.Dura.n^3: H.Dura.n^4	2.5E-11	9.0E-12	2.831	0.0048	-	-	-
H.Dose: H.Dura.n: H.Dura.n^3: H.Dura.n^4	2.6E-12	1.2E-12	2.159	0.0312	-	-	-

Formatted: Centered
Formatted: Centered
Formatted: Centered
Formatted: Centered
Formatted: Centered
Formatted: Centered
Formatted: Centered
Formatted: Centered
Formatted: Centered
Formatted: Centered
Formatted: Centered
Formatted: Centered
Formatted: Centered
Formatted: Centered
Formatted: Centered
Formatted: Centered
Formatted: Centered
Formatted: Centered
Formatted: Centered
Formatted: Centered
Formatted: Centered
Formatted: Centered
Formatted: Centered
Formatted: Centered
Formatted: Centered
Formatted: Centered
Formatted: Centered
Formatted: Centered
Formatted: Centered

For peer review only

Table 4: Dose-Response Relationships

Cross-Sectional Studies	-	-	-	-	-	-	-
-	-	-	-	-	-	-	-
Parameter	Variable Statistics				Model Summaries		
-	Estimate	Std. Error	t Value	Pr(>|t|)	F	dF	P
-	-	-	-	-	-	-	-
Both Sexes	-	-	-	-	-	-	-
H.Dura.n	-0.0059	0.0021	-2.779	0.0056	8.628	2, 664	0.0002
H.Dose: H.Dura.n	0.0091	0.0022	4.152	0.0000	-	-	-
-	-	-	-	-	-	-	-
Males	-	-	-	-	-	-	-
H.Dura.n	-0.0089	0.0028	-3.147	0.0018	7.105	2, 441	0.0009
H.Dose: H.Dura.n	0.0124	0.0034	3.658	0.0003	-	-	-
-	-	-	-	-	-	-	-
Females	-	-	-	-	-	-	-
H.Dose: H.Dura.n	0.0052	0.0023	2.281	0.0236	5.201	1, 210	0.0236
-	-	-	-	-	-	-	-
Longitudinal Studies							-
-							-
Group	Variable Statistics					Model	
Parameter	Value	Std.Error	DF	t-value	P-value	AIC*	LogLik*
-	-	-	-	-	-	-	-
Both Sexes	-	-	-	-	-	-	-
H.Dose:H.Dura.n	0.0045	0.0013	814	3.5512	0.0004	1068.74	-530.37
-	-	-	-	-	-	-	-
Males	-	-	-	-	-	-	-
H.Dura.n	-0.0036	0.0016	552	-2.2069	0.0277	753.15	-371.58
H.Dose:H.Dura.n	0.0075	0.0022	552	3.3754	0.0008	-	-
-	-	-	-	-	-	-	-
Females	-	-	-	-	-	-	-
H.Dose:H.Dura.n	0.0052	0.0021	260	2.5091	0.0127	347.43	-169.72

Formatted: Centered
Formatted: Centered
Formatted: Centered
Formatted: Centered
Formatted: Centered
Formatted: Centered
Formatted: Centered
Formatted: Centered
Formatted: Centered
Formatted: Centered
Formatted: Centered
Formatted: Centered
Formatted: Centered
Formatted: Centered
Formatted: Centered
Formatted: Centered
Formatted: Centered
Formatted: Centered
Formatted: Centered
Formatted: Centered
Formatted: Centered
Formatted: Centered
Formatted: Centered
Formatted: Centered
Formatted: Centered
Formatted: Centered
Formatted: Centered
Formatted: Centered
Formatted: Centered
Formatted: Centered
Formatted: Centered

For peer review only

Table 5.: Cross-Sectional Adjusted Multiple Regression Models

Group	Variable Statistics				Model		
Parameter	Estimate	Std. Error	t Value	Pr(>|t|)	F	dF	P
-	-	-	-	-	-	-	-
Both Sexes	-	-	-	-	-	-	-
H.Dura.n: Cigs.d: SP : Weight	0.00023	0.00006	3.919	0.0001	7.561	9, 657	1.38E-10
H.Dura.n: Height: Cigs.d: SP	0.00030	0.00011	2.840	0.0047	-	-	-
H.Dura.n: Height: Cigs.d	-0.00143	0.00051	-2.828	0.0048	-	-	-
H.Dura.n: Height: Cigs.d: SP : Weight	-0.00007	0.00002	-2.808	0.0051	-	-	-
H.Dura.n: Height: Cigs.d: Weight	0.00032	0.00012	2.741	0.0063	-	-	-
H.Dose: H.Dura.n: Height: Cigs.d: Weight	-0.00037	0.00016	-2.229	0.0261	-	-	-
H.Dose: H.Dura.n: Height: Cigs.d: SP : Weight	0.00008	0.00003	2.229	0.0262	-	-	-
H.Dose: H.Dura.n: Height: Cigs.d	0.00155	0.00071	2.194	0.0286	-	-	-
H.Dose: H.Dura.n: Height: Cigs.d: SP	-0.00032	0.00015	-2.188	0.0290	-	-	-
-	-	-	-	-	-	-	-
Males	-	-	-	-	-	-	-
H.Dura.n: Weight	-0.0024	0.0007	-3.434	0.0007	5.485	10, 438	1.07E-07
H.Dose: H.Dura.n: Weight	0.0032	0.0010	3.158	0.0017	-	-	-
H.Dose: Cigs.d: SP : Weight	-4.4770	1.7470	-2.563	0.0107	-	-	-
H.Dose: Height: Cigs.d: SP : Weight	0.0253	0.0099	2.557	0.0109	-	-	-
H.Dose: Cigs.d: SP	19.460	7.6160	2.556	0.0109	-	-	-
H.Dose: Cigs.d: Weight	21.370	8.3900	2.548	0.0112	-	-	-
H.Dose: Height: Cigs.d: Weight	-0.1209	0.0476	-2.542	0.0114	-	-	-
H.Dose: Height: Cigs.d: SP	-0.1098	0.0432	-2.540	0.0114	-	-	-
H.Dose: Cigs.d	-92.680	36.520	-2.538	0.0115	-	-	-
H.Dose: Height: Cigs.d	0.5230	0.2073	2.523	0.0120	-	-	-
-	-	-	-	-	-	-	-
Females	-	-	-	-	-	-	-
Height: Cigs.d: Weight	-0.00041	0.00009	-4.327	0.00002	8.25	3, 214	3.20E-05
Height: Cigs.d: SP : Weight	0.00008	0.00002	4.272	0.00003	-	-	-
H.Dose: H.Dura.n: Height: Cigs.d	2.2E-06	1.1E-06	1.957	0.05160	-	-	-

Formatted: Centered
Formatted: Centered
Formatted: Centered
Formatted: Centered
Formatted: Centered
Formatted: Centered
Formatted: Centered
Formatted: Centered
Formatted: Centered
Formatted: Centered
Formatted: Centered
Formatted: Centered
Formatted: Centered
Formatted: Centered
Formatted: Centered
Formatted: Centered
Formatted: Centered
Formatted: Centered
Formatted: Centered
Formatted: Centered
Formatted: Centered
Formatted: Centered
Formatted: Centered
Formatted: Centered
Formatted: Centered
Formatted: Centered
Formatted: Centered
Formatted: Centered
Formatted: Centered
Formatted: Centered
Formatted: Centered
Formatted: Centered

For peer review only

Table 6.: Longitudinal Adjusted Multiple Models

Group	Variable Statistics					Model	
Model / Parameter	Value	Std.Error	DF	t-value	P-value	AIC*	LogLik*
-	-	-	-	-	-	-	-
Both Sexes	-	-	-	-	-	-	-
Height	-0.0304	0.0026	606	-11.7198	0.0E+00	1238.239	592.1197
SP	0.7947	0.0949	606	8.3772	0.0E+00	-	-
Days:H.Dura.n	0.0003	6.9E-05	606	4.3556	0.0E+00	-	-
CA:Height	0.0066	0.0006	606	10.9049	0.0E+00	-	-
Days: CA:H.Dura.n	-0.0001	0.0000	606	-4.2946	0.0E+00	-	-
H.Dura.n: Cigs.d:Height	-5.3E-05	1.3E-05	606	-4.1566	0.0E+00	-	-
Days:H.Dose: Cigs.d:Height	2.1E-06	5.0E-07	606	4.3108	0.0E+00	-	-
Days: CA:H.Dose: Cigs.d:Height	-6.0E-07	2.0E-07	606	-4.2968	0.0E+00	-	-
CA:H.Dura.n: Cigs.d:Height	1.4E-05	3.4E-06	606	4.0217	0.0001	-	-
Days: CA: Cigs.d:Height	0.0E+00	0.0E+00	606	3.7701	0.0002	-	-
Days:H.Dura.n: Cigs.d	-1.1E-05	3.1E-06	606	-3.4709	0.0006	-	-
Cigs.d:Height	0.0007	0.0002	606	3.3714	0.0008	-	-
Days: CA:H.Dose:H.Dura.n: Cigs.d:Height	0.0E+00	0.0E+00	606	3.2705	0.0011	-	-
Days: CA:H.Dura.n: Cigs.d	2.5E-06	8.0E-07	606	3.2365	0.0013	-	-
CA: Cigs.d:Height	-0.0002	0.0001	606	-3.2045	0.0014	-	-
Days	-0.0022	0.0007	606	-2.9194	0.0036	-	-
H.Dose:H.Dura.n: Cigs.d	0.0102	0.0036	606	2.8304	0.0048	-	-
-CA:H.Dose:H.Dura.n: Cigs.d	-0.0026	0.0010	606	-2.6804	0.0076	-	-
Days: CA	0.0005	0.0002	606	2.2438	0.0252	-	-
Days: CA:H.Dose:Height	6.8E-06	3.1E-06	606	2.1984	0.0283	-	-
Days:H.Dose:Height	-2.2E-05	1.0E-05	606	-2.1747	0.0300	-	-
Days:H.Dose:H.Dura.n	-3.5E-05	1.7E-05	606	-2.1086	0.0354	-	-
H.Dose: Cigs.d	-0.1067	0.0511	606	-2.0888	0.0371	-	-
CA:H.Dose: Cigs.d	0.0290	0.0148	606	1.9645	0.0499	-	-
-	-	-	-	-	-	-	-
Males	-	-	-	-	-	-	-
Height	-0.0302	0.0035	436	-8.7423	0.0E+00	913.00	435.50
SP	0.6376	0.1105	436	5.7717	0.0E+00	-	-
CA:Height	0.0064	0.0008	436	8.4711	0.0E+00	-	-
Days:H.Dura.n	0.0003	0.0001	436	3.8907	0.0001	-	-
Days: CA:H.Dura.n	-0.0001	0.0000	436	-3.8395	0.0001	-	-
Days:H.Dose: Cigs.d:Height	1.6E-06	4.0E-07	436	3.8181	0.0002	-	-
Days: CA:H.Dose: Cigs.d:Height	-5.0E-07	1.0E-07	436	-3.7217	0.0002	-	-
Days	-0.0029	0.0008	436	-3.6624	0.0003	-	-
Days: CA	0.0007	0.0002	436	3.3251	0.0010	-	-
H.Dose:H.Dura.n: Cigs.d:Height	2.7E-06	8.0E-07	436	3.2331	0.0013	-	-
Days: CA: Cigs.d	5.0E-06	1.6E-06	436	3.1839	0.0016	-	-
H.Dura.n: Cigs.d:Height	-2.8E-05	9.1E-06	436	-3.1058	0.0020	-	-
CA:H.Dura.n: Cigs.d:Height	7.1E-06	2.4E-06	436	2.9496	0.0034	-	-
Days:H.Dura.n: Cigs.d	-8.3E-06	3.1E-06	436	-2.6824	0.0076	-	-
Days: CA:H.Dura.n: Cigs.d	2.0E-06	8.0E-07	436	2.5562	0.0109	-	-
Days: CA:H.Dose:H.Dura.n: Cigs.d:Height	0.0E+00	0.0E+00	436	2.2838	0.0229	-	-
Cigs.d:Height	0.0004	0.0002	436	2.2799	0.0231	-	-
CA: Cigs.d:Height	-0.0001	5.0E-05	436	-2.1938	0.0288	-	-
-	-	-	-	-	-	-	-

Formatted ... [51]
Formatted ... [52]
Formatted ... [53]
Formatted ... [54]
Formatted ... [55]
Formatted ... [56]
Formatted ... [57]
Formatted ... [58]
Formatted ... [59]
Formatted ... [60]
Formatted ... [61]
Formatted ... [62]
Formatted ... [63]
Formatted ... [64]
Formatted ... [65]
Formatted ... [66]
Formatted ... [67]
Formatted ... [68]
Formatted ... [69]
Formatted ... [70]
Formatted ... [71]
Formatted ... [72]
Formatted ... [73]
Formatted ... [74]
Formatted ... [75]
Formatted ... [76]
Formatted ... [77]
Formatted ... [78]
Formatted ... [79]
Formatted ... [80]
Formatted ... [81]
Formatted ... [82]
Formatted ... [83]
Formatted ... [84]
Formatted ... [85]
Formatted ... [86]
Formatted ... [87]
Formatted ... [88]
Formatted ... [89]
Formatted ... [90]
Formatted ... [91]
Formatted ... [92]
Formatted ... [93]
Formatted ... [94]
Formatted ... [95]
Formatted ... [96]
Formatted ... [97]
Formatted ... [98]
Formatted ... [99]

For peer review only

Females	-	-	-	-	-	-	-
Height	-0.0279	0.0036	169	-7.6753	0.0E+00	394.69	-187.35
SP	1.2880	0.1930	169	6.6744	0.0E+00	-	-
CA:Height	0.0064	0.0005	169	11.7493	0.0E+00	-	-
Days: CA:H.Dose:H.Dura.n:Height	1.3E-06	5.0E-07	169	2.7371	0.0069	-	-
Days:H.Dose:H.Dura.n:Height	-4.5E-06	1.7E-06	169	-2.7147	0.0073	-	-
Days: CA:H.Dose:H.Dura.n: Cigs.d:Height	-1.0E-07	0.0E+00	169	-2.6052	0.0100	-	-
Days:H.Dose:H.Dura.n: Cigs.d:Height	2.0E-07	1.0E-07	169	2.5589	0.0114	-	-

Formatted: Centered
Formatted: Centered
Formatted: Centered
Formatted: Centered
Formatted: Centered
Formatted: Centered
Formatted: Centered
Formatted: Centered
Formatted: Centered

For peer review only

TABLES

For peer review only

Table 1.: Effects of Gender on Key Measures of Arterial Stiffness - Cross-Sectional Dataset

**Dependent Variable**	**Group**	**Parameter**	**Adj. R^2**	**F**	**dF**	**Model P**	**Estimate**	**Upper Limit**	**Lower Limit**	**P**
RA-CA Difference	Opioid	Chron.Age :Female.Sex	0.0189	7.596	2, 684	0.0005	0.6324	1.3123	-0.0475	0.0688
BMI	Control	Chron.Age :Female.Sex	0.1523	52.58	2, 572	<0.0001	-0.0092	-0.0014	-0.0170	0.0213
BMI	Opioid	Female.Sex	0.0713	27.35	2, 684	<0.0001	-0.0827	-0.0555	-0.1099	<0.0001
RA/BMI	Opioid	Chron.Age :Female.Sex	0.2617	122.6	2, 684	<0.0001	0.0361	0.0539	0.0183	<0.0001
C_AP_HR75/BMI	Control	Chron.Age :Female.Sex	0.4882	274.3	2, 571	<0.0001	0.0312	0.0390	0.0234	<0.0001
C_AP_HR75/BMI	Control	Chron.Age :Female.Sex	0.4882	274.3	2, 571	<0.0001	0.0312	0.0390	0.0234	<0.0001
C_AP_HR75/BMI	Opioid	Chron.Age :Female.Sex	0.3712	203.4	2, 684	<0.0001	0.0467	0.0543	0.0391	<0.0001
C_AGPH_HR75/BMI	Control	Chron.Age :Female.Sex	0.4603	245.4	2, 571	<0.0001	0.0924	0.1144	0.0704	<0.0001
C_AGPH_HR75/BMI	Opioid	Chron.Age :Female.Sex	0.3980	227.8	2, 684	<0.0001	0.1265	0.1451	0.1079	<0.0001
C_AP_HR75/(CA*BMI)	Control	Female.Sex	0.2809	112.9	2, 571	<0.0001	0.0033	0.0043	0.0023	<0.0001
C_AP_HR75/(CA*BMI)	Opioid	Female.Sex	0.2489	114.7	2, 684	<0.0001	0.0047	0.0055	0.0039	<0.0001
C_AGPH_HR75/(CA*BMI)	Control	Female.Sex	0.2567	99.96	2, 571	<0.0001	0.0096	0.0125	0.0067	<0.0001
C_AGPH_HR75/(CA*BMI)	Opioid	Female.Sex	0.2604	121.8	2, 684	<0.0001	0.0130	0.0152	0.0108	<0.0001

For peer review only

Table 2.:

Age: Addictive Status Relationships with CA Discretized

About 41 Years in Males and 29 Years in Females

With Females <55 Years of Age

Parameter	Variable Statistics				Model Summaries		
	Estimate	Upper Limit	Lower Limit	P	F	dF	P
Males							
Older Age	-0.1837	-0.0745	-0.2929	0.0010	5.492	3, 862	0.0010
Opioids	-0.0846	-0.0307	-0.1385	0.0021			
Older Age: Opioids	0.2310	0.3657	0.0963	0.0008			
Females							
Older Age: Opioids	0.1248	0.2291	0.0205	0.0195	2.169	3, 378	0.0912

For peer review only

Table 3.: Cross-sectional Models Polynomial in Heroin Duration

	Parameter				Model		
**Model / Parameter**	**Value**	**Upper Limit**	**Lower Limit**	**P-value**	**F**	**dF**	**P**
1 Order- Linear							
H.Dura.n	-0.0059	-0.0018	-0.0100	0.0056	8.628	2, 664	0.0002
H.Dose: H.Dura.n	0.0091	0.0134	0.0048	3.7.E-05			
2 Order- Quadratic							
H.Dura.n	-0.0171	-0.0063	-0.0279	0.0018	7.443	3, 663	<0.0001
H.Dura.n^2	0.0004	0.0008	0.0000	0.0261			
H.Dose: H.Dura.n	0.0084	0.0127	0.0041	0.0002			
3 Order - Cubic							
H.Dura.n	-0.0116	-0.0047	-0.0185	0.0012	7.102	3 663	0.0001
H.Dura.n^3	6.3E-06	0.0000	0.0000	0.0466			
H.Dose: H.Dura.n	0.0084	0.0127	0.0041	0.0001			
4 Order- Quartic							
H.Dura.n	-0.0059	-0.0018	-0.0100	0.0056	8.628	2, 664	0.0002
H.Dose: H.Dura.n	0.0091	0.0134	0.0048	3.7.E-05			
1, 2, 3 & 4 Order							
H.Dose: H.Dura.n^3	6.5E-05	1.0E-04	2.6E-05	0.0012	3.353	7, 659	0.0016
H.Dura.n^2 : H.Dura.n^3	-1.0E-06	-3.9E-07	-1.6E-06	0.0007			
H.Dura.n^2 : H.Dura.n^4	9.2E-08	1.5E-07	3.5E-08	0.0014			
H.Dura.n^3: H.Dura.n^4	-2.7E-09	-9.8E-10	-4.4E-09	0.0026			
H.Dose: H.Dura.n^2 : H.Dura.n^4	-4.5E-09	-9.7E-10	-8.0E-09	0.0145			
H.Dura.n: H.Dura.n^3: H.Dura.n^4	2.5E-11	4.3E-11	7.4E-12	0.0048			
H.Dose: H.Dura.n: H.Dura.n^3: H.Dura.n^4	2.6E-12	5.0E-12	2.5E-13	0.0312			

For peer review only

Table 4: Dose-Response Relationships

Cross-Sectional Studies

Parameter	Variable Statistics				Model Summaries		
	Estimate	Upper Limit	Lower Limit	Pr(>|t|)	F	dF	P
Both Sexes							
H.Dura.n	-0.0059	-0.0018	-0.0100	0.0056	8.628	2, 664	0.0002
H.Dose: H.Dura.n	0.0091	0.0134	0.0048	0.0000			
Males							
H.Dura.n	-0.0089	-0.0034	-0.0144	0.0018	7.105	2, 441	0.0009
H.Dose: H.Dura.n	0.0124	0.0191	0.0057	0.0003			
Females							
H.Dose: H.Dura.n	0.0052	0.0097	0.0007	0.0236	5.201	1, 210	0.0236

Longitudinal Studies

Group	Variable Statistics					Model	
Parameter	Value	Upper Limit	Lower Limit	t-value	P-value	AIC*	LogLik*
Both Sexes (DF=814)							
H.Dose:H.Dura.n	0.0045	0.0070	0.0020	3.5512	0.0004	1068.74	-530.37
Males (DF=552)							
H.Dura.n	-0.0036	-0.0005	-0.0067	-2.2069	0.0277	753.15	-371.58
H.Dose:H.Dura.n	0.0075	0.0118	0.0032	3.3754	0.0008		
Females (DF=260)							
H.Dose:H.Dura.n	0.0052	0.0093	0.0011	2.5091	0.0127	347.43	-169.72

For peer review only

Table 5.: Cross-Sectional Adjusted Regression Models

Group	Variable Statistics				Model		
**Parameter**	**Estimate**	**Upper Limit**	**Lower Limit**	**Pr(>|t|)**	**F**	**dF**	**P**
Both Sexes							
H.Dura.n: Cigs.d: SP : Weight	0.00023	0.0003	0.0001	0.0001	7.561	9, 657	<0.0001
H.Dura.n: Height: Cigs.d: SP	0.00030	0.0005	0.0001	0.0047			
H.Dura.n: Height: Cigs.d	-0.00143	-0.0004	-0.0024	0.0048			
H.Dura.n: Height: Cigs.d: SP : Weight	-0.00007	0.0000	-0.0001	0.0051			
H.Dura.n: Height: Cigs.d: Weight	0.00032	0.0006	0.0001	0.0063			
H.Dose: H.Dura.n: Height: Cigs.d: Weight	-0.00037	-0.0001	-0.0007	0.0261			
H.Dose: H.Dura.n: Height: Cigs.d: SP : Weight	0.00008	0.0001	0.0000	0.0262			
H.Dose: H.Dura.n: Height: Cigs.d	0.00155	0.0029	0.0002	0.0286			
H.Dose: H.Dura.n: Height: Cigs.d: SP	-0.00032	0.0000	-0.0006	0.0290			
Males							
H.Dura.n: Weight	-0.0024	-0.0010	-0.0038	0.0007	5.485	10, 438	<0.0001
H.Dose: H.Dura.n: Weight	0.0032	0.0052	0.0012	0.0017			
H.Dose: Cigs.d: SP : Weight	-4.4770	-1.0529	-7.9011	0.0107			
H.Dose: Height: Cigs.d: SP : Weight	0.0253	0.0447	0.0059	0.0109			
H.Dose: Cigs.d: SP	19.460	34.3874	4.5326	0.0109			
H.Dose: Cigs.d: Weight	21.370	37.8144	4.9256	0.0112			
H.Dose: Height: Cigs.d: Weight	-0.1209	-0.0276	-0.2142	0.0114			
H.Dose: Height: Cigs.d: SP	-0.1098	-0.0251	-0.1945	0.0114			
H.Dose: Cigs.d	-92.680	-21.100	-164.25	0.0115			
H.Dose: Height: Cigs.d	0.5230	0.9293	0.1167	0.0120			
Females							
Height: Cigs.d: Weight	-0.00041	-0.0002	-0.0006	0.00002	8.25	3, 214	<0.0001
Height: Cigs.d: SP : Weight	0.00008	0.0001	0.0000	0.00003			
H.Dose: H.Dura.n: Height: Cigs.d	2.2E-06	0.0000	0.0000	0.05160			

For peer review only

Table 6.: Longitudinal Adjusted Regression Models

Group	Variable Statistics					Model	
**Model / Parameter**	**Value**	**Upper Limit**	**Lower Limit**	**t-value**	**P-value**	**AIC***	**LogLik***
Both Sexes (DF=606)							
Height	-0.0304	-0.0253	-0.0355	-11.7198	0.0E+00	1238.239	-592.1197
SP	0.7947	0.9807	0.6087	8.3772	0.0E+00		
Days:H.Dura.n	0.0003	0.0004	0.0002	4.3556	0.0E+00		
CA:Height	0.0066	0.0078	0.0054	10.9049	0.0E+00		
Days: CA:H.Dura.n	-0.0001	-0.0001	-0.0001	-4.2946	0.0E+00		
H.Dura.n: Cigs.d:Height	-5.3E-05	-2.8E-05	-7.8E-05	-4.1566	0.0E+00		
Days:H.Dose: Cigs.d:Height	2.1E-06	3.1E-06	1.1E-06	4.3108	0.0E+00		
Days: CA:H.Dose: Cigs.d:Height	-6.0E-07	-2.1E-07	-9.9E-07	-4.2968	0.0E+00		
CA:H.Dura.n: Cigs.d:Height	1.4E-05	2.1E-05	7.3E-06	4.0217	0.0001		
Days: CA: Cigs.d:Height	0.0E+00	0.0E+00	0.0E+00	3.7701	0.0002		
Days:H.Dura.n: Cigs.d	-1.1E-05	-4.9E-06	-1.7E-05	-3.4709	0.0006		
Cigs.d:Height	0.0007	0.0011	0.0003	3.3714	0.0008		
Days: CA:H.Dose:H.Dura.n: Cigs.d:Height	0.0E+00	0.0E+00	0.0E+00	3.2705	0.0011		
Days: CA:H.Dura.n: Cigs.d	2.5E-06	4.1E-06	9.3E-07	3.2365	0.0013		
CA: Cigs.d:Height	-0.0002	0.0000	-0.0004	-3.2045	0.0014		
Days	-0.0022	-0.0008	-0.0036	-2.9194	0.0036		
H.Dose:H.Dura.n: Cigs.d	0.0102	0.0173	0.0031	2.8304	0.0048		
CA:H.Dose:H.Dura.n: Cigs.d	-0.0026	-0.0006	-0.0046	-2.6804	0.0076		
Days: CA	0.0005	0.0009	0.0001	2.2438	0.0252		
Days: CA:H.Dose:Height	6.8E-06	1.3E-05	7.2E-07	2.1984	0.0283		
Days:H.Dose:Height	-2.2E-05	-2.4E-06	-4.2E-05	-2.1747	0.0300		
Days:H.Dose:H.Dura.n	-3.5E-05	-1.7E-06	-6.8E-05	-2.1086	0.0354		
H.Dose: Cigs.d	-0.1067	-0.0065	-0.2069	-2.0888	0.0371		
CA:H.Dose: Cigs.d	0.0290	0.0580	0.0000	1.9645	0.0499		
Males (DF=436)							
Height	-0.0302	-0.0233	-0.0371	-8.7423	0.0E+00	913.00	-435.50
SP	0.6376	0.8542	0.4210	5.7717	0.0E+00		
CA:Height	0.0064	0.0080	0.0048	8.4711	0.0E+00		
Days:H.Dura.n	0.0003	0.0005	0.0001	3.8907	0.0001		
Days: CA:H.Dura.n	-0.0001	-0.0001	-0.0001	-3.8395	0.0001		
Days:H.Dose: Cigs.d:Height	1.6E-06	2.4E-06	8.2E-07	3.8181	0.0002		
Days: CA:H.Dose: Cigs.d:Height	-5.0E-07	-3.0E-07	-7.0E-07	-3.7217	0.0002		
Days	-0.0029	-0.0013	-0.0045	-3.6624	0.0003		
Days: CA	0.0007	0.0011	0.0003	3.3251	0.0010		
H.Dose:H.Dura.n: Cigs.d:Height	2.7E-06	4.3E-06	1.1E-06	3.2331	0.0013		
Days: CA: Cigs.d	5.0E-06	8.1E-06	1.9E-06	3.1839	0.0016		
H.Dura.n: Cigs.d:Height	-2.8E-05	-1.0E-05	-4.6E-05	-3.1058	0.0020		
CA:H.Dura.n: Cigs.d:Height	7.1E-06	1.2E-05	2.4E-06	2.9496	0.0034		
Days:H.Dura.n: Cigs.d	-8.3E-06	-2.2E-06	-1.4E-05	-2.6824	0.0076		
Days: CA:H.Dura.n: Cigs.d	2.0E-06	3.6E-06	4.3E-07	2.5562	0.0109		
Days: CA:H.Dose:H.Dura.n: Cigs.d:Height	0.0E+00	0.0E+00	0.0E+00	2.2838	0.0229		
Cigs.d:Height	0.0004	0.0008	0.0000	2.2799	0.0231		
CA: Cigs.d:Height	-0.0001	-2.0E-06	-2.0E-04	-2.1938	0.0288		

Females (DF=169)	_	_	_	_	_	_	_
Height	-0.0279	-0.0208	-0.0350	-7.6753	0.0E+00	394.69	-187.35
SP	1.2880	1.6663	0.9097	6.6744	0.0E+00	_	_
CA:Height	0.0064	0.0074	0.0054	11.7493	0.0E+00	_	_
Days: CA:H.Dose:H.Dura.n:Height	1.3E-06	2.3E-06	3.2E-07	2.7371	0.0069	_	_
Days:H.Dose:H.Dura.n:Height	-4.5E-06	-1.2E-06	-7.8E-06	-2.7147	0.0073	_	_
Days: CA:H.Dose:H.Dura.n: Cigs.d:Height	-1.0E-07	-1.0E-07	-1.0E-07	-2.6052	0.0100	_	_
Days:H.Dose:H.Dura.n: Cigs.d:Height	2.0E-07	4.0E-07	4.0E-09	2.5589	0.0114	_	_

For peer review only

4/29/2014 9:48:00 PM

4/29/2014 9:48:00 PM

4/29/2014 9:48:00 PM

4/29/2014 9:48:00 PM

4/29/2014 9:48:00 PM

4/29/2014 9:48:00 PM

4/29/2014 9:48:00 PM

4/29/2014 9:48:00 PM

4/29/2014 9:48:00 PM

For peer review only

4/29/2014 9:48:00 PM
4/29/2014 9:48:00 PM
4/29/2014 9:48:00 PM
4/29/2014 9:48:00 PM
4/29/2014 9:48:00 PM
4/29/2014 9:48:00 PM
4/29/2014 9:48:00 PM
4/29/2014 9:48:00 PM
4/29/2014 9:48:00 PM

For peer review only

4/21/2014 4:19:00 PM

4/21/2014 4:19:00 PM

4/21/2014 4:19:00 PM

4/21/2014 4:19:00 PM

4/21/2014 4:19:00 PM

4/21/2014 4:19:00 PM

4/21/2014 4:19:00 PM

4/21/2014 4:19:00 PM

4/21/2014 4:19:00 PM

For peer review only

4/21/2014 4:19:00 PM

4/21/2014 4:19:00 PM
4/21/2014 4:19:00 PM
4/21/2014 4:19:00 PM
4/21/2014 4:19:00 PM
4/21/2014 4:19:00 PM
4/21/2014 4:19:00 PM
4/21/2014 4:19:00 PM
4/21/2014 4:19:00 PM

For peer review only

4/21/2014 4:19:00 PM
4/21/2014 4:19:00 PM
4/21/2014 4:19:00 PM
4/21/2014 4:19:00 PM
4/21/2014 4:19:00 PM
4/21/2014 4:19:00 PM
4/21/2014 4:19:00 PM
4/21/2014 4:19:00 PM

For peer review only

Figure 1

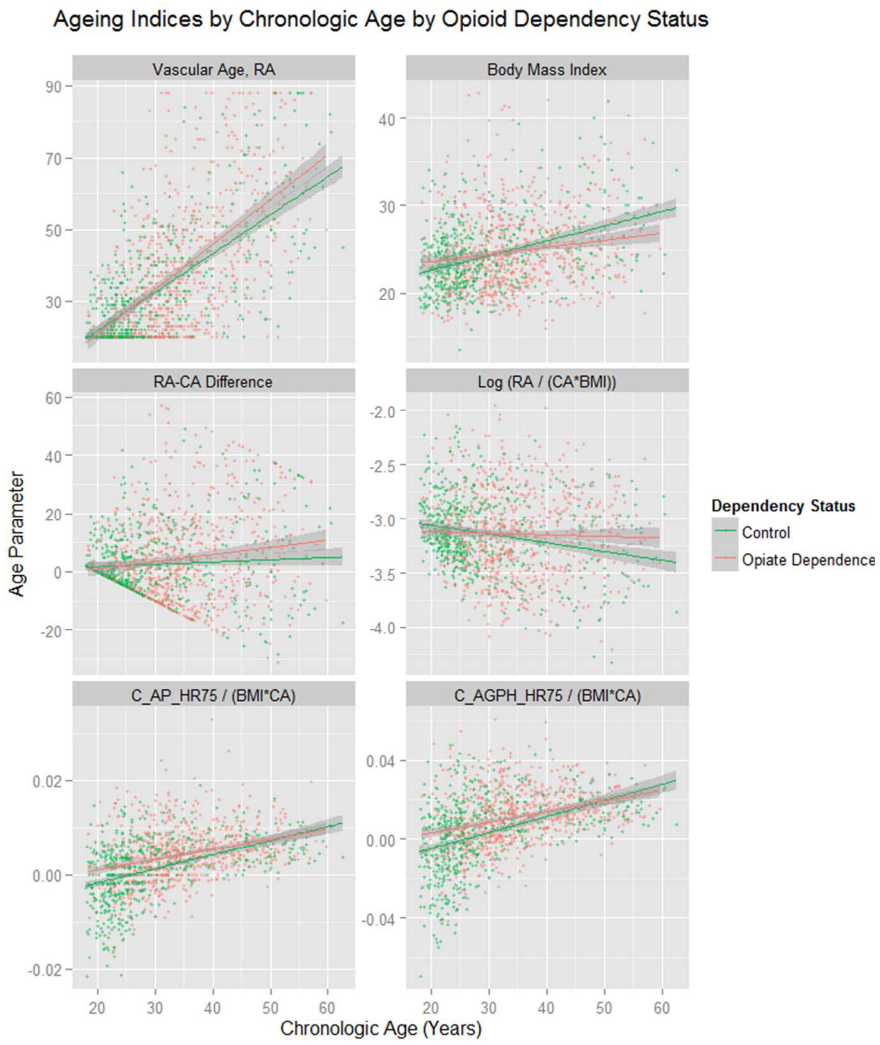

Ageing and Stiffness Indices by Chronologic Age by Opioid Dependency Status
190x254mm (300 x 300 DPI)

Figure 2

Opioid

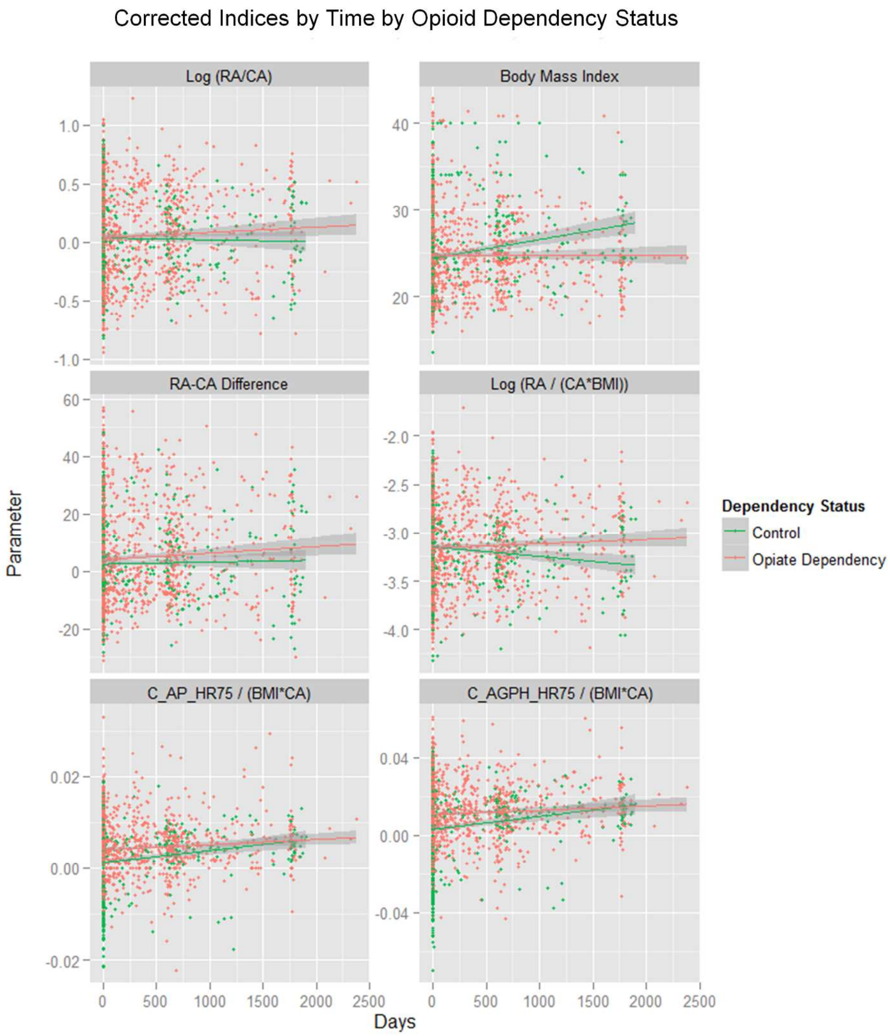

Corrected Ageing and Stiffness Indices by Time by Opioid Dependency Status
190x254mm (300 x 300 DPI)

Figure 3

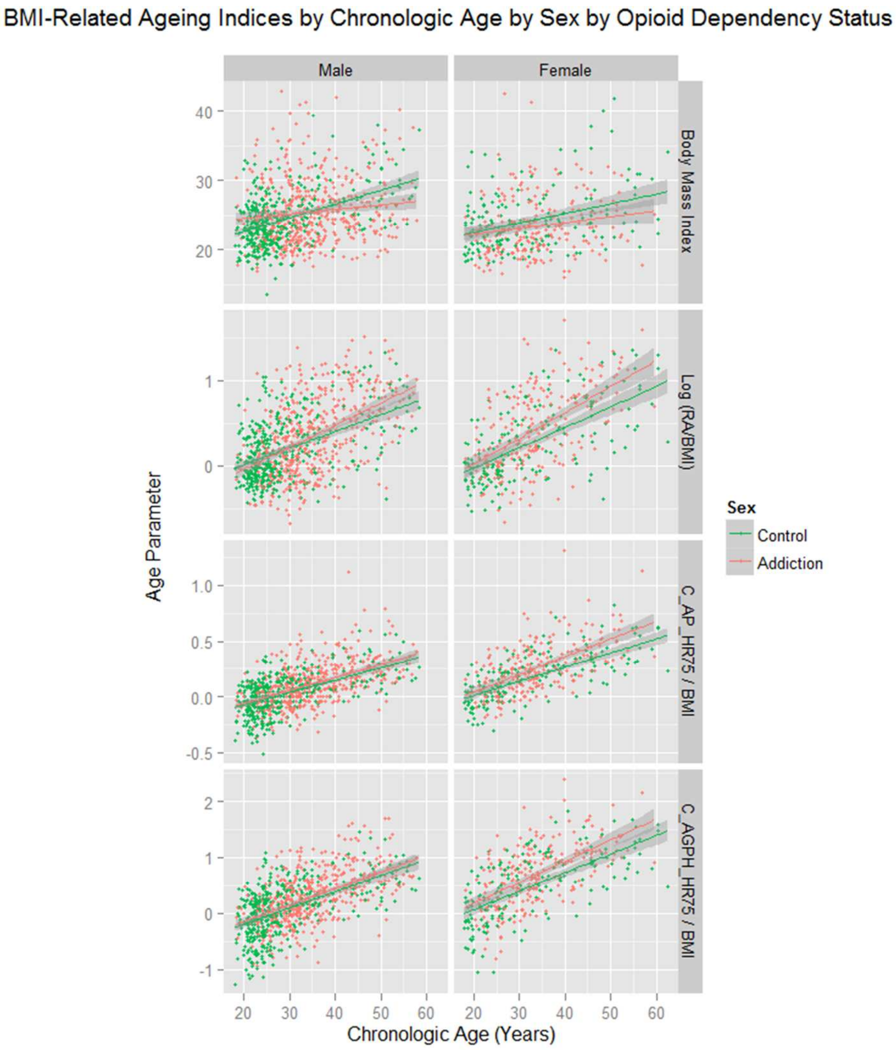

BMI-Related Ageing Indices by Chronologic Age by Addictive Status by Sex
190x254mm (300 x 300 DPI)

Figure 4

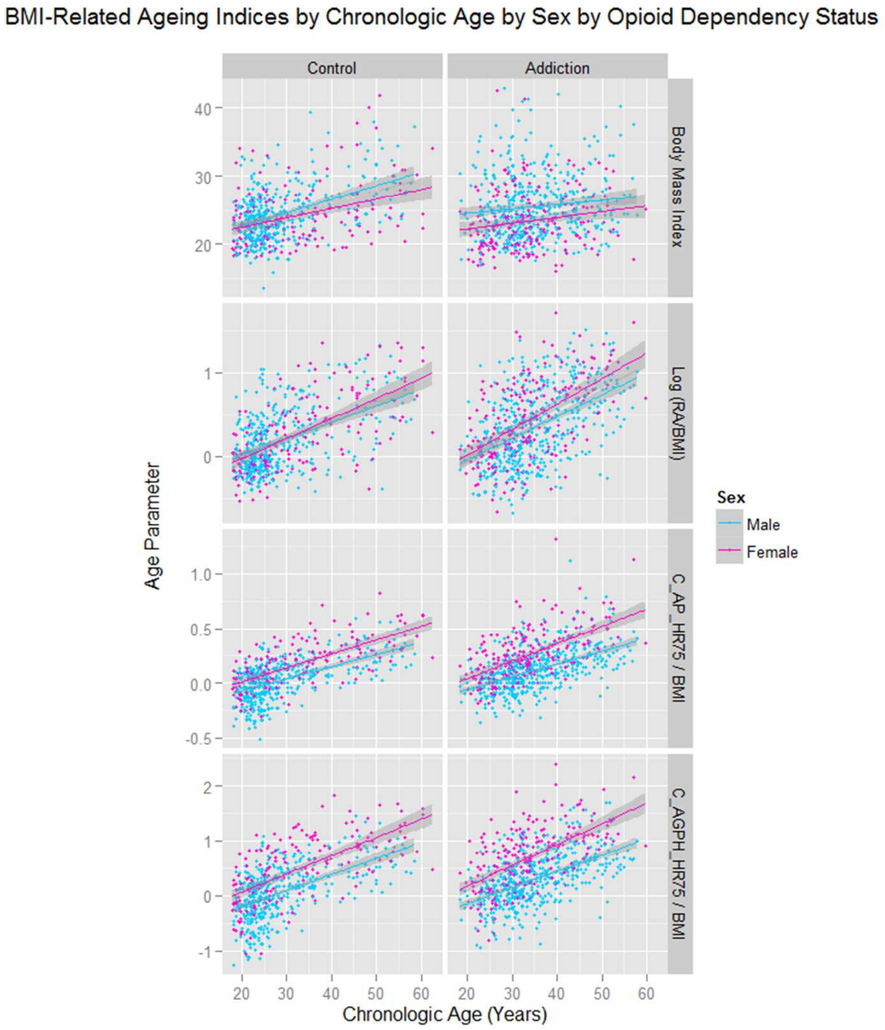

BMI-Related Ageing Indices by Chronologic Age by Sex by Addictive Status
190x254mm (300 x 300 DPI)

Figure 5

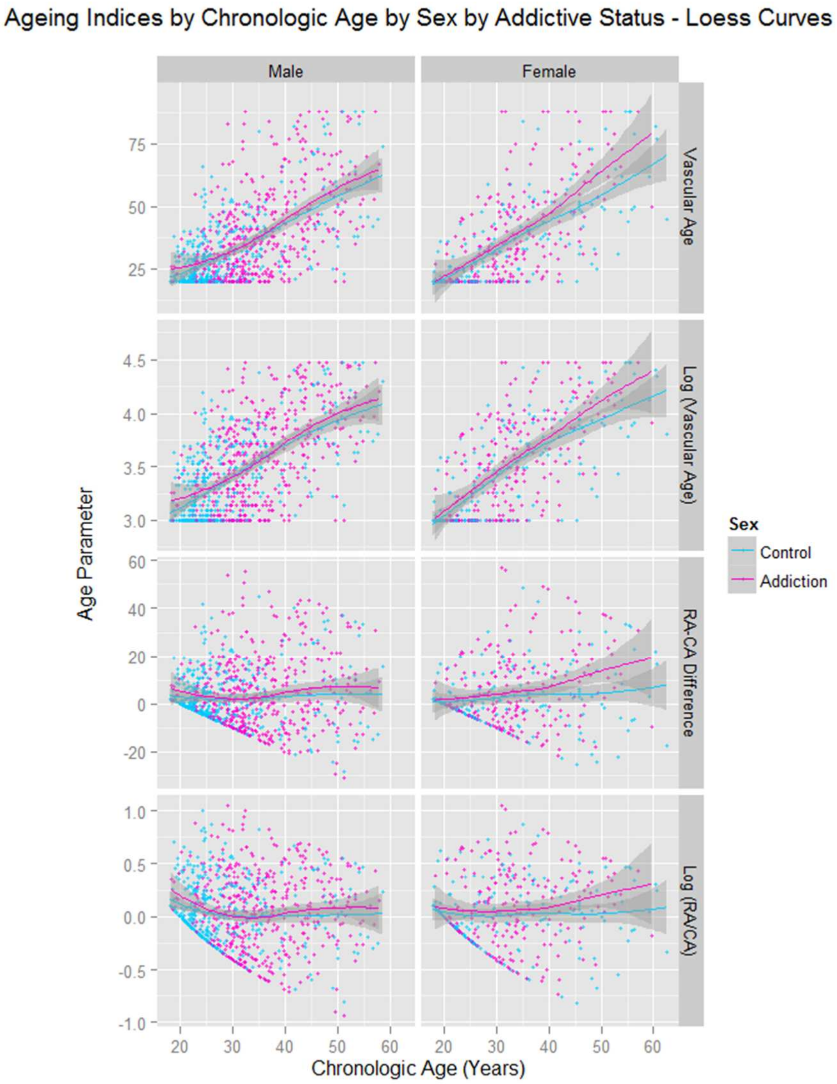

Ageing Indices by Chronologic Age by Sex by Addictive Status – Loess Curves
190x254mm (300 x 300 DPI)

Figure 6

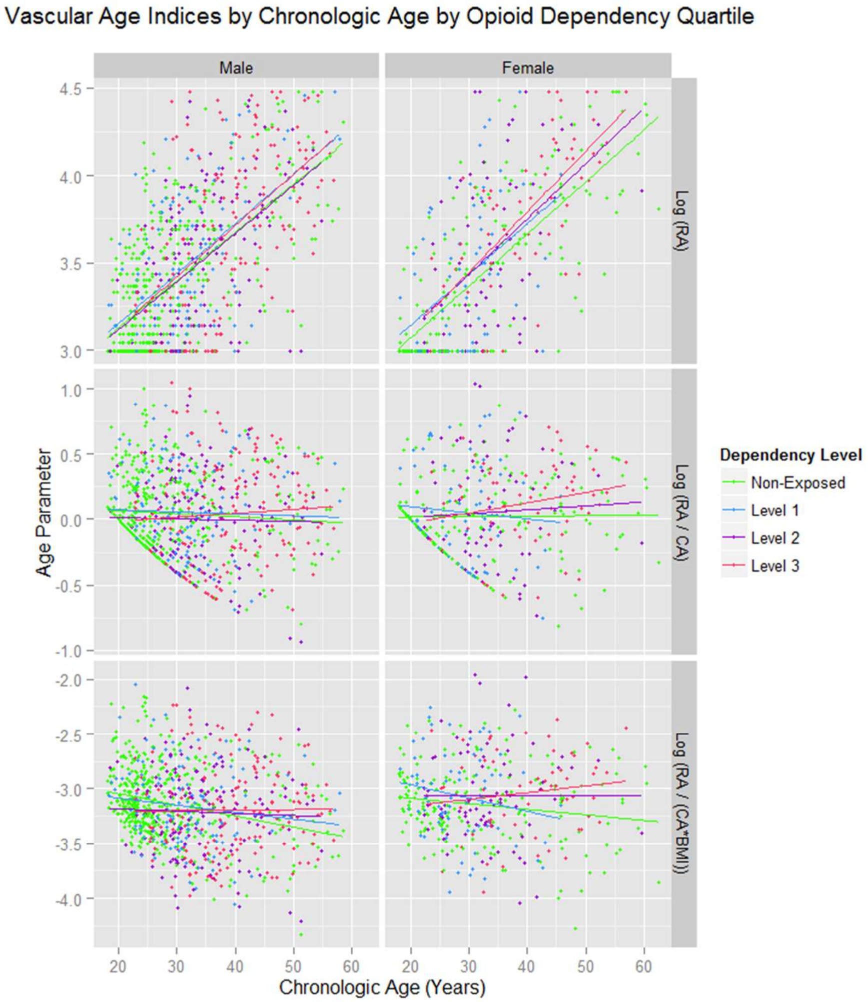

Vascular Age Indices by Chronologic Age by Opioid Dependency Quartile
190x254mm (300 x 300 DPI)

SUPPLEMENTARY TABLE

Supplementary Table 1.: Bivariate Comparisons of Baseline Sociodemographic, Laboratory and Drug Use Variables

	Control	Opiate Dependence	t-value	df	P
Chronologic_Age (Yrs)	29.96_(0.45)	34.55_(0.32)	-8.262	1082.22	0.0000
Biometrics					
Height_(cm)	173.59_(0.37)	173.51_(0.33)	0.172	1261.00	0.8633
Weight_(kg)	73.21_(0.58)	74.98_(0.63)	-2.071	1260.00	0.0385
BMI_(kg/m^2)	24.25_(0.17)	24.8_(0.17)	-2.263	1256.60	0.0238
Drug Use					
Tobacco (%Smokers)	0.16_(0.02)	0.92_(0.01)	-41.558	1029.42	0.0000
Cigarettes/d	1.78_(0.22)	16.64_(0.37)	-34.982	1089.65	0.0000
Minutes After Smoking	121.98_(8.48)	96.48_(12.98)	1.645	757.57	0.1004
Heroin Dose (g/d)	0_(0)	0.53_(0.02)	-21.717	682.28	0.0000
Heroin Duration (Yr)	0.09_(0.05)	13.01_(0.33)	-38.783	695.26	0.0000
Lifetime Heroin Exposure (g-Yr)	0.03_(0.02)	7.81_(0.65)	-11.987	666.93	0.0000
Buprenorphine Dose (mg/d)	_()	6.98_(0.21)		586.00	
Methadone Dose (mg/d)	_()	63_(4.01)		66.00	
Laboratory Values					
Cholesterol (mmol/l)	4.78_(0.08)	4.45_(0.04)	3.497	781.00	0.0005
Triglyceride (mmol/l)	1.43_(0.08)	1.4_(0.04)	0.439	781.00	0.6608
HDL (mmol/l)	1.31_(0.04)	1.24_(0.02)	1.551	522.00	0.1215
LDL (mmol/l)	2.87_(0.1)	2.47_(0.04)	3.698	119.75	0.0003
ALT (IU/L)	32.8_(2.07)	65.59_(4.04)	-7.214	778.88	0.0000
AST (IU/L)	30.92_(3.9)	48.04_(2.26)	-3.793	318.80	0.0002
Glucose (mmol/l)	5.03_(0.14)	5.49_(0.2)	-1.231	785.00	0.2185
Creatinine (mcmol/l)	78.86_(1.28)	79.75_(0.68)	-0.634	785.00	0.5263
Urea (mmol/l)	5.18_(0.09)	5.12_(0.21)	0.155	783.00	0.8765
Albumin (g/L)	44.23_(0.32)	43.6_(0.2)	1.533	784.00	0.1256
Globulin (g/L)	29.3_(0.26)	31.53_(0.2)	-6.735	421.08	0.0000
CRP (mg/L)	3.43_(0.65)	6.11_(0.42)	-3.459	269.11	0.0006
ESR (mm/hr)	7.85_(0.59)	12.76_(0.5)	-6.374	365.75	0.0000
Lymphocytes (x10^9/ml)	2.32_(0.05)	2.54_(0.04)	-2.754	759.00	0.0060
Monocytes (x10^9/ml)	0.56_(0.02)	0.63_(0.01)	-2.404	629.00	0.0165
Calcium (mmol/l)	2.37_(0.01)	2.37_(0)	0.111	784.00	0.9119
Phosphate (mmol/l)	1.18_(0.02)	1.22_(0.01)	-2.491	334.08	0.0132
HBcAb Positivity Rate	0.17_(0.05)	0.25_(0.02)	-1.514	70.28	0.1344
HCV Ab Positivity Rate	0.52_(0.07)	0.82_(0.02)	-4.161	56.84	0.0001

Data presented as mean $\pm$ S.E.M.

For peer review only

Supplementary Table 2.:

Numbers of Longitudinal Studies

No. Studies	Groups		No. Studies		
	Control	Opiate Dependent	Control	Opiate Dependent	Total
1	456	388	456	388	844
2	45	144	90	288	378
3	12	68	36	204	240
4	6	36	24	144	168
5	3	15	15	75	90
6	4	10	24	60	84
7	2	5	14	35	49
8	2	5	16	40	56
9	0	1	0	9	9
10	1	1	10	10	20
11	0	1	0	11	11
12	0	1	0	12	12
13	0	1	0	13	13
16	0	1	0	16	16
25	1	0	25	0	25
Totals			710	1305	2015

Numbers of stuidies performed relate to the ease of follow-up of patients.

For peer review only

Supplementary Table 3.:

Bivariate Comparisons of Central and Peripheral Cardiovascular Parameters

	Control	Opiate Dependence	t-value	df	p
**Operator Index**	88.58_(0.26)	88.34_(0.25)	0.683	1261.00	0.4946
Ages					
Chronologic_Age	29.96_(0.45)	34.55_(0.32)	-8.262	1082.22	0.0000
Vascular_Age	32.57_(0.68)	39.12_(0.7)	-6.729	1255.67	0.0000
Log(CA)	3.35_(0.01)	3.51_(0.01)	-10.375	1076.20	0.0000
Log(RA)	3.39_(0.02)	3.57_(0.02)	-7.448	1242.33	0.0000
RA-CA_Difference	2.61_(0.47)	4.57_(0.57)	-2.652	1245.27	0.0081
RA/CA	1.09_(0.01)	1.13_(0.02)	-1.821	1259.62	0.0688
Augmentation					
C_AP_HR75	1.86_(0.21)	3.94_(0.19)	-7.351	1260.00	0.0000
C_AGPH_HR75	4.78_(0.56)	10.17_(0.46)	-7.503	1260.00	0.0000
C_PH	35.29_(0.29)	37.36_(0.28)	-5.123	1261.00	0.0000
C_T1R	154.76_(0.65)	152.42_(0.54)	2.783	1233.00	0.0055
C_AL	8.25_(0.23)	9.14_(0.18)	-3.078	1037.00	0.0021
C_ATI	49.83_(1.6)	57.02_(1.32)	-3.488	1037.00	0.0005
PPAmpRatio	156.97_(0.79)	150.79_(0.74)	5.686	1260.00	0.0000
P_MAX_DPDT	844.69_(7.35)	850.89_(7.16)	-0.601	1261.00	0.5478
Timing					
HR	69.26_(0.47)	69.99_(0.45)	-1.100	1261.00	0.2714
ED	324.74_(0.84)	323.5_(0.82)	1.054	1261.00	0.2923
C_SVI	144.72_(1.29)	142.43_(1.11)	1.356	1261.00	0.1754
C_DTI	3015.42_(16.3)	3023.56_(15.82)	-0.358	1243.62	0.7201
C_TTI	2151.42_(15.84)	2186.99_(15.28)	-1.609	1261.00	0.1079
Pressure					
SP	123.29_(0.47)	123.77_(0.51)	-0.696	1260.87	0.4862
DP	68.6_(0.38)	68.18_(0.38)	0.775	1255.83	0.4383
C_SP	105.16_(0.45)	106.99_(0.46)	-2.836	1254.81	0.0046
C_DP	69.92_(0.38)	69.69_(0.39)	0.432	1256.15	0.6657
C_ESP	92.72_(0.47)	94.54_(0.46)	-2.769	1261.00	0.0057
C_MEANP	86.19_(0.39)	86.93_(0.4)	-1.305	1256.22	0.1920

Data presented as mean ± S.E.M.

For peer review only

Supplementary Table 4:

Cross-sectional Factor Analysis

lm(log (RA/BMI) ~ log(CA)*Addictive Status)

Parameter	Variable Statistics				Model Summaries		
	Estimate	Std. Error	t Value	Pr(>|t|)	F	dF	P
All Patients							
Opiates	-0.58071	0.2615	-2.221	0.0265	184.8	3, 1258	<2.2E-16
CA: Opiates	0.1756	0.0757	2.32	0.0205			
Males							
Opiates	-0.73731	0.3270	-2.255	0.0244	109.1	3, 862	<2.2E-16
CA: Opiates	0.2128	0.0950	2.24	0.0253			
Females							
CA: Opiates	0.0300	0.0109	2.747	0.0063	116.7	2, 393	<2.2E-16

For peer review only

Supplementary Table 5.:

Longitudinal Factor Analysis

lme(log (RA/CA) ~ log(CA)*Days*Addictive Status)

Group / Parameter	Parameter Values					Model Parameters	
	Value	Std.Error	DF	t-value	Pr(>|t|)	AIC	LogLik
All Patients							
CA:Days	0.00015	0.00006	811	2.5762	0.0102	1124.528	-555.2641
Days:Opiates	0.00010	0.00003	811	2.9978	0.0028		
Males							
Days:Control	-0.00001	0.00003	551	-0.2042	0.8383	780.7702	-384.3851
Days:Opiates	0.00005	0.00002	551	2.6199	0.009		
Females							
Days:Opiates	-0.00115	0.00052	256	-2.2160	0.0276	413.4612	-198.7306
CA:Days:Opiates	0.00032	0.00014	256	2.2185	0.0274		

For peer review only

Supplementary Table 6.:

Cross-sectional Factor Analysis

Interactions of Sex with CA.

Variable	Variable Statistics					Model Summaries	
	Parameter	Estimate	Std. Error	z - Value	P, Prob.(>|z|)	LogLik / F	Wald / dF
RA	Male.Sex	0.94149	0.32377	2.908	0.00364	-787.6	813.7
RA	CA: Male.Sex	-0.26694	0.09293	-2.873	0.00407	-787.6	813.7
C_AP_HR75	CA: Male.Sex	-0.87594	0.06461	-13.56	<2E-16	580.8	2, 1259
C_AGPH_HR75	CA: Male.Sex	-2.4701	0.1626	-15.2	<2E-16	75.55	3, 1259
C_PH	Male.Sex	21.987	4.682	4.696	2.94E-06	75.55	2, 1259
C_PH	CA: Male.Sex	-5.63	1.354	-4.158	3.43E-05	75.55	2, 1259
PPAmpRatio	CA: Male.Sex	2.6901	0.2569	10.47	<2E-16	483.6	2, 1259
C_T1R	Male.Sex	9.6358	0.8174	11.79	<2E-16	130.6	2, 1232

All Interactive Models, except for C_T1R
All model P-values < 2.0E-16
Model for RA is tobit (with LogLik and Wald Statistics). All others are standard linear regression models with CA.

For peer review only

Supplementary Table 7.:

Longitudinal Factor Analysis

Interactions of Time, CA and Sex.

**Group**	**Variable Statistics**					**Model**	
**Parameter**	**Value**	**Std.Error**	**DF**	**t-value**	**p-value**	**AIC**	**LogLik**
RA							
CA: Days: Opiates	2.5E-05	9.0E-06	809	2.7328	0.0064	1149.41	-564.7
Days	-0.0005	0.0002	809	-2.5125	0.0122		
CA: Days	0.0001	0.0001	809	2.3973	0.0167		
C_AGPH_HR75							
CA	30.8204	1.6476	804	18.7068	0.0000	14393.8	-7181.9
Days: Male.Sex	-0.0356	0.0117	804	-3.0428	0.0024		
CA: Days: Male.Sex	0.0087	0.0030	804	2.8711	0.0042		
CA: Male.Sex	-5.1991	2.0124	804	-2.5836	0.0100		
Days: Male.Sex: Opiates	0.0335	0.0142	804	2.3631	0.0184		
CA: Days: Female.Sex: Opiates	0.0124	0.0058	804	2.1522	0.0317		
CA: Days: Male.Sex: Opiates	-0.0079	0.0037	804	-2.1274	0.0337		
Days: Female.Sex: Opiates	-0.0440	0.0218	804	-2.0213	0.0436		
C_AP_HR75							
Days: Male.Sex	-0.0198	0.0049	806	-4.0071	0.0001	10866.4	-5420.2
CA: Days: Male.Sex	0.0050	0.0013	806	3.9398	0.0001		
CA: Days: Opiates	0.0005	0.0002	806	2.8924	0.0039		
Days: Female.Sex	-0.0121	0.0045	806	-2.6830	0.0074		
CA: Days: Male.Sex: Opiates	-0.0041	0.0016	806	-2.5760	0.0102		
CA: Days: Female.Sex	0.0030	0.0012	806	2.5498	0.0110		
Days: Male.Sex: Opiates	0.0150	0.0060	806	2.5092	0.0123		
CA: Male.Sex	-2.0296	0.8114	806	-2.5013	0.0126		
C_PH							
Male.Sex	27.1005	5.0888	1198	5.3255	0.0E+00	13297.5	-6633.7
CA: Male.Sex	-7.0598	1.4690	804	-4.8058	0.0E+00		
Days	-0.0306	0.0085	804	-3.6178	0.0003		
CA: Days	0.0080	0.0022	804	3.6326	0.0003		
Days: Male.Sex: Opiates	0.0298	0.0106	804	2.8028	0.0052		
Opiates	13.5294	4.8766	804	2.7743	0.0057		
CA: Days: Male.Sex: Opiates	-0.0079	0.0029	804	-2.6945	0.0072		
CA: Opiates	-3.6162	1.4128	804	-2.5596	0.0107		

For peer review only

Supplementary Table 8.:

Significance Levels for Quartile P Values <0.1

Cross-sectional and Longitudinal Studies

Cross-sectional Studies

Group / Parameter	Parameter Values				Model Parameters		
	Estimate	Std. Error	t Value	Pr(>|t|)	F	dF	P
All Patients							
H.Exposure.Level.3	-0.7103	0.3845	-1.847	0.0650	1.126	7, 1235	0.3437
CA: H.Exposure.Level.3	0.2056	0.1069	1.924	0.0546			
Males							
Females							
CA: H.Exposure.Level.3	0.0265	0.0139	1.911	0.0568	1.193	4, 388	0.3133

Longitudinal Studies

Group / Parameter	Parameter Values					Model Parameters	
	Value	Std.Error	DF	t-value	Pr(>|t|)	AIC	LogLik
All Patients							
Days: H.Exposure.Level.1	0.00013	0.00004	810	2.9797	0.0030	1152.85	-568.42
Days: H.Exposure.Level.2	0.00016	0.00004	810	3.6254	0.0003		
Days: H.Exposure.Level.3	0.00006	0.00004	810	1.7297	0.0841		
Males							
Days: H.Exposure.Level.1	0.00011	0.00005	549	2.2010	0.0281	828.94	-406.47
Days: H.Exposure.Level.2	0.00014	0.00005	549	2.5507	0.0110		
Females							
Days: H.Exposure.Level.1	-0.00176	0.00100	253	-1.7637	0.0790	475.43	-226.71
Days: H.Exposure.Level.3	-0.00203	0.00103	253	-1.9681	0.0501		
Days: CA: H.Exposure.Level.1	0.00050	0.00028	253	1.7653	0.0787		
Days: CA: H.Exposure.Level.3	0.00054	0.00028	253	1.9298	0.0547		

For peer review only

Supplementary Table 9.:

Summary of Longitudinal Models of Quartiles of Opiate Exposure

(lme (Variable/CA)~ Time*Opiate.Exposure.Quartile)

**Parameter**		**Variable Statistics**					**Model**	
**Group**	**Variable**	**Value**	**Std.Error**	**DF**	**t-value**	**P-value**	**AIC**	**LogLik**
RA/CA								
All Patients	Days: H.Exposure.Level.2	0.00007	0.00003	811	2.26807	0.0236	1143.78	-564.89
Males	Days: H.Exposure.Level.2	0.00007	0.00004	550	2.066028	0.0393	813.62	-399.81
Females	-							
C_AGPH_HR75 / CA								
All Patients	Days	0.00004	0.00001	811	3.372907	8E-04	1176.95	-581.48
	H.Exposure.Level.1	0.15146	0.02842	811	5.329696	0E+00		
	H.Exposure.Level.2	0.17167	0.02721	811	6.308965	0E+00		
	H.Exposure.Level.3	0.20433	0.02761	811	7.400001	0E+00		
Males	Days	0.00004	0.00001	550	2.861191	0.0044	645.42	-315.71
	H.Exposure.Level.1	0.14683	0.03182	550	4.614867	0E+00		
	H.Exposure.Level.2	0.15382	0.03017	550	5.099172	0E+00		
	H.Exposure.Level.3	0.21693	0.02999	550	7.233373	0E+00		
Females	Days	0.00004	0.00003	257	1.689176	0.0924	418.64	-202.3216
	H.Exposure.Level.1	0.09204	0.04998	257	1.841536	0.0667		
	H.Exposure.Level.2	0.17788	0.04887	257	3.640107	0.0003		
	H.Exposure.Level.3	0.17765	0.05221	257	3.402395	0.0008		
C_AP_HR75 / CA								
All Patients	Days	0.00002	0.00001	811	4.778954	0E+00	-2674.138	1344.069
	H.Exposure.Level.1	0.05655	0.01070	811	5.284006	0E+00		
	H.Exposure.Level.2	0.06166	0.01024	811	6.019479	0E+00		

For peer review only

	H.Exposure.Level.3	0.08000	0.01040	811	7.696224	0E+00		
Males	Days	0.00002	0.00001	550	4.104246	0E+00	-1943.355	978.6774
	H.Exposure.Level.1	0.05617	0.01229	550	4.570086	0E+00		
	H.Exposure.Level.2	0.05482	0.01164	550	4.710096	0E+00		
	H.Exposure.Level.3	0.08586	0.01157	550	7.424323	0E+00		
Females	Days	0.00002	0.00001	257	2.369984	0.0185	-806.9698	410.4849
	H.Exposure.Level.1	0.03297	0.01826	257	1.805227	0.0722		
	H.Exposure.Level.2	0.06411	0.01786	257	3.589983	0.0004		
	H.Exposure.Level.3	0.06855	0.01909	257	3.590865	0.0004		
C_PH / CA								
All Patients	Days	-0.00004	0.00001	811	-4.989103	0E+00	-215.7032	114.8516
	H.Exposure.Level.1	-0.01120	0.02041	811	-0.548578	0.5834		
	H.Exposure.Level.2	-0.10747	0.01955	811	-5.497201	0E+00		
	H.Exposure.Level.3	-0.18195	0.01984	811	-9.171153	0E+00		
Males	Days	-0.00004	0.00001	550	-3.439231	0.0006	-151.6348	82.81741
	H.Exposure.Level.1	-0.06511	0.02450	550	-2.657892	0.0081		
	H.Exposure.Level.2	-0.13868	0.02327	550	-5.958834	0E+00		
	H.Exposure.Level.3	-0.22848	0.02315	550	-9.86833	0E+00		
Females	Days	-0.00006	0.00002	257	-3.811813	0.0002	-76.14242	45.07121
	H.Exposure.Level.1	0.12933	0.03421	257	3.78028	0.0002		
	H.Exposure.Level.2	-0.02369	0.03343	257	-0.708426	0.4793		
	H.Exposure.Level.3	-0.06000	0.03567	257	-1.681998	0.0938		

Supplementary Table 10.:

ANOVA Comparisons of Cross-Sectional Models Polynomial in Opiate Duration

In Table 1.

**Model**	**RSS**	
m1	84.517	
m2	83.888	
m3	84.014	
m4	84.517	
m1234	83.731	
**Comparison**	**F**	**P-value**
m1 v m2	4.9694	0.02613
m1 v m3	3.9729	0.04665
m1 v m4	-	-
m2 v m3	-	-
m2 v m4	4.9694	0.02613
m3 v m4	3.9729	0.04665
m1234 v m1	1.2372	0.2899
m1234 v m2	0.3093	0.8718
m1234 v m3	0.5559	0.6948
m1234 v m4	1.2372	0.2899

RSS – Residual Sum of Squares
m1 - Opiate Exposure Duration - Linear Model
m2 - Opiate Exposure Duration - Quadratic Model
m3 - Opiate Exposure Duration - Cubic Model
m4 - Opiate Exposure Duration - Quartic Model
m1234 - Opiate Exposure Duration – Combined Linear, Quadratic, Cubic and Quartic Model

For peer review only

Supplementary Table 11.:

Summary of Longitudinal Models Polynomial in Opiate Duration

Model	Variable Statistics					Model	
**Parameter**	**Value**	**Std.Error**	**DF**	**t-value**	**p-value**	**AIC**	**LogLik**
1 Order- Linear							
H.Dose:H.Dura.n	0.0045	0.0013	814	3.5512	0.0004	1068.74	-530.37
2 Order- Quadratic							
H.Dura.n^2	-0.0009	0.0003	809	-3.5503	0.0004	1138.29	-560.15
H.Dura.n: H.Dura.n^2	2.9E-05	0.00000838	809	3.4714	0.0005		
H.Dose:H.Dura.n: H.Dura.n^2	-0.0001	0.00002745	809	-3.2360	0.0013		
H.Dose: H.Dura.n^2	0.0041	0.0014	809	2.9052	0.0038		
H.Dose	0.1659	0.0830	809	1.9994	0.0459		
H.Dose:H.Dura.n	-0.0407	0.0204	809	-1.9982	0.0460		
3 Order - Cubic							
H.Dura.n^3	0.0000	1.2526E-05	811	-3.3596	0.0008	1156.22	-571.11
H.Dose: H.Dura.n^3	0.0001	1.9606E-05	811	4.0694	0.0001		
H.Dura.n: H.Dura.n^3	1.3E-06	3.94E-07	811	3.3670	0.0008		
H.Dose:H.Dura.n: H.Dura.n^3	-2.4E-06	6.14E-07	811	-3.8553	0.0001		
4 Order- Quartic							
H.Dose	0.0924	0.0358	810	2.5811	0.0100	1171.45	-577.73
H.Dura.n	-0.0065	0.0019	810	-3.3983	0.0007		
H.Dose: H.Dura.n^4	1.9E-06	0.0000006	810	3.0901	0.0021		
H.Dura.n: H.Dura.n^4	1.0E-08	0.0E+00	810	2.9797	0.003		
H.Dose:H.Dura.n: H.Dura.n^4	-6.0E-08	0.00000002	810	-3.0810	0.0021		
1, 2 & 4 Order							
H.Dura.n: H.Dura.n^2	-0.0023	0.0008	804	-3.0322	0.0025	1310.95	-641.48
H.Dose:H.Dura.n: H.Dura.n^2	0.0050	0.0017	804	3.0052	0.0027		
H.Dura.n^4	0.0002	0.00005279	804	3.0026	0.0028		
H.Dose: H.Dura.n^2	-0.0270	0.0092	804	-2.9399	0.0034		
H.Dose: H.Dura.n^4	-0.0003	0.0001	804	-2.9034	0.0038		
H.Dura.n: H.Dura.n^4	-4.5E-06	0.00000156	804	-2.8937	0.0039		
H.Dura.n^2	0.0110	0.0039	804	2.8136	0.0050		
H.Dura.n^2 : H.Dura.n^4	5.0E-08	0.00000002	804	2.7676	0.0058		
H.Dose:H.Dura.n: H.Dura.n^4	9.0E-06	0.00000327	804	2.7629	0.0059		
H.Dose: H.Dura.n^2 : H.Dura.n^4	-9.0E-08	0.00000003	804	-2.6261	0.0088		
H.Dose	0.1584	0.0714	804	2.2175	0.0269		

Supplementary Table 12.:

ANOVA Comparison of Longitudinal Models of Table 10

Model	AIC	LogLik
m1	1049.67	-520.83
m2	1040.85	-511.42
m3	1043.21	-514.60
m4	1043.11	-513.56
m124	1041.09	-506.54
**Comparison**	**L.Ratio**	**P-value**
m1 v m2	18.82	0.0021
m1 v m3	12.46	0.0060
m1 v m4	14.55	0.0057
m2 v m3	6.36	0.0416
m2 v m4	4.27	0.0388
m3 v m4	2.09	0.1482
m124 v m1	28.58	0.0015
m124 v m2	9.76	0.0824
m124 v m3	16.12	0.0241
m124 v m4	14.03	0.0294

AIC – Akiane Information Criteria
Log Lik. - Log Likelihood Ratio
L. Ratio - Log Likelihood Ratio
m1 - Opiate Exposure Duration - Linear Model
m2 - Opiate Exposure Duration - Quadratic Model
m3 - Opiate Exposure Duration - Cubic Model
m4 - Opiate Exposure Duration - Quartic Model
m124 - Opiate Exposure Duration – Combined Linear, Quadratic, and Quartic Model

For peer review only

Supplementary Table 13.:

Full Multivariate Analysis of Log (RA/CA) Including All Biochemical Variables

Parameter	Variable Statistics				Model Summaries		
	Estimate	Std. Error	t Value	Pr(>|t|)	F	dF	P
Both Sexes							
H.Dura.n: Height: SP : CRP	0.0003	0.0001	2.835	0.0048	3.030	12, 381	0.00044
H.Dura.n: Height: CRP	-0.0014	0.0005	-2.82	0.0051			
H.Dose: CRP	6.2416	2.3936	2.608	0.0095			
H.Dose: SP : CRP	-1.2903	0.4964	-2.599	0.0097			
H.Dose: Height: Cigs.d: SP	0.0004	0.0001	2.487	0.0133			
H.Dose: Height: Cigs.d	-0.0017	0.0007	-2.432	0.0155			
H.Dose: Cigs.d: SP : CRP	0.5385	0.2346	2.295	0.0223			
H.Dose: Cigs.d: CRP	-2.5285	1.1265	-2.245	0.0254			
H.Dose: Height: Cigs.d: SP : CRP	-0.0029	0.0013	-2.137	0.0332			
H.Dura.n: Cigs.d: SP : CRP	-0.0020	0.0010	-2.103	0.0361			
H.Dose: Height: Cigs.d: CRP	0.0134	0.0065	2.082	0.0380			
H.Dura.n: Cigs.d: CRP	0.0096	0.0046	2.079	0.0383			
Males							
H.Dose: Height: Cigs.d: CRP	-0.0007	0.0002	-3.534	0.0005	3.323	10, 264	0.0004
H.Dose: Cigs.d: SP : CRP	0.0214	0.0066	3.224	0.0014			
H.Dura.n: Height: CRP	-0.0008	0.0003	-2.984	0.0031			
H.Dura.n: Height: SP : CRP	0.0002	0.0001	2.969	0.0033			
H.Dose: Height: CRP	0.0231	0.0081	2.863	0.0045			
H.Dose: Height: SP : CRP	-0.0046	0.0017	-2.723	0.0069			
H.Dose: Height: Cigs.d: SP	0.0004	0.0001	2.716	0.0070			
H.Dose: Height: Cigs.d	-0.0019	0.0007	-2.583	0.0103			
Cigs.d: CRP	0.1028	0.0450	2.284	0.0232			
Cigs.d: SP : CRP	-0.0208	0.0093	-2.236	0.0262			
Females							
Cigs.d: CRP	-0.12299	0.04178	-2.944	0.00392	4.484	2, 116	0.01331
Cigs.d: SP : CRP	0.02583	0.00883	2.926	0.00413			

For peer review only

Suppl. Figure 1

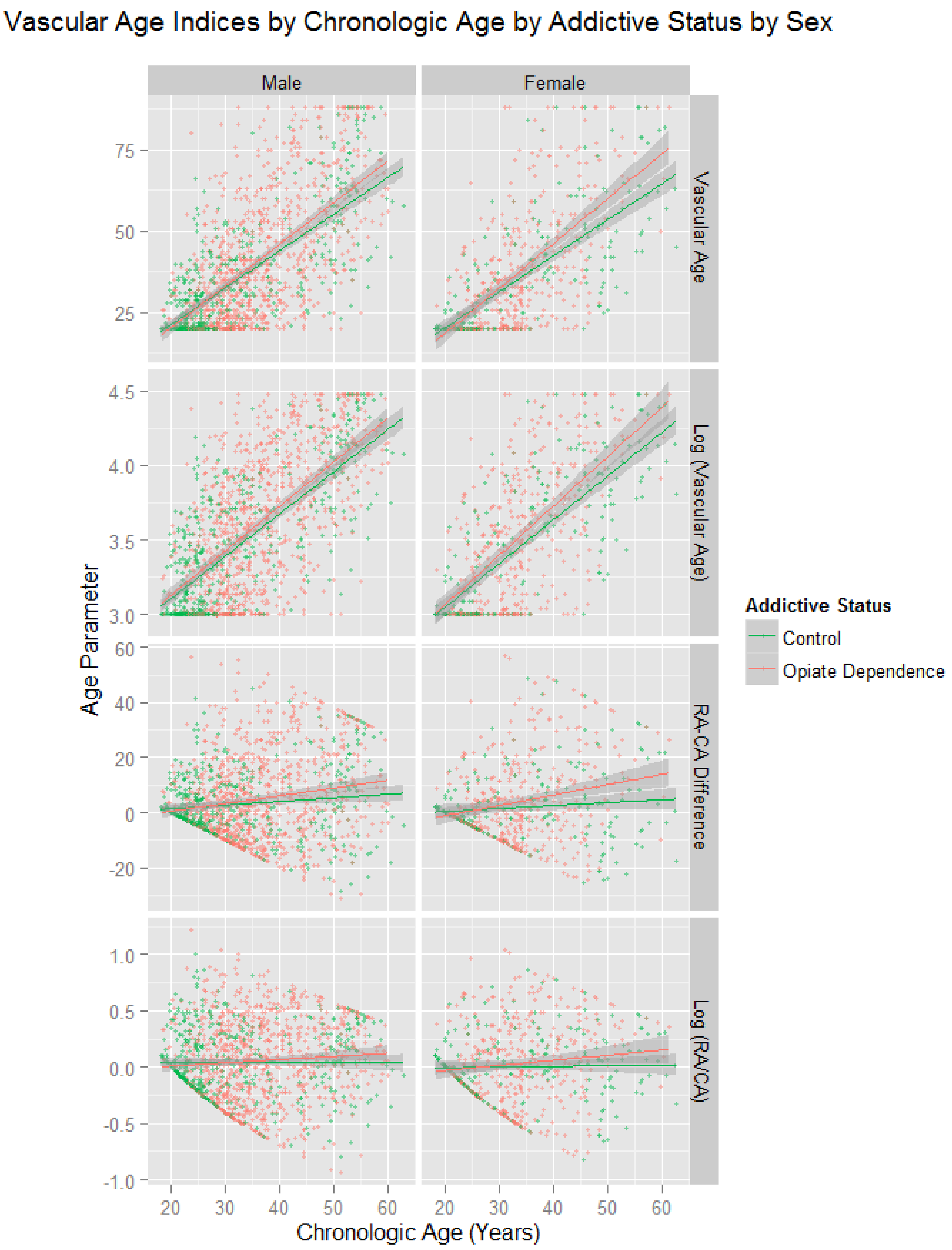

Suppl. Figure 2

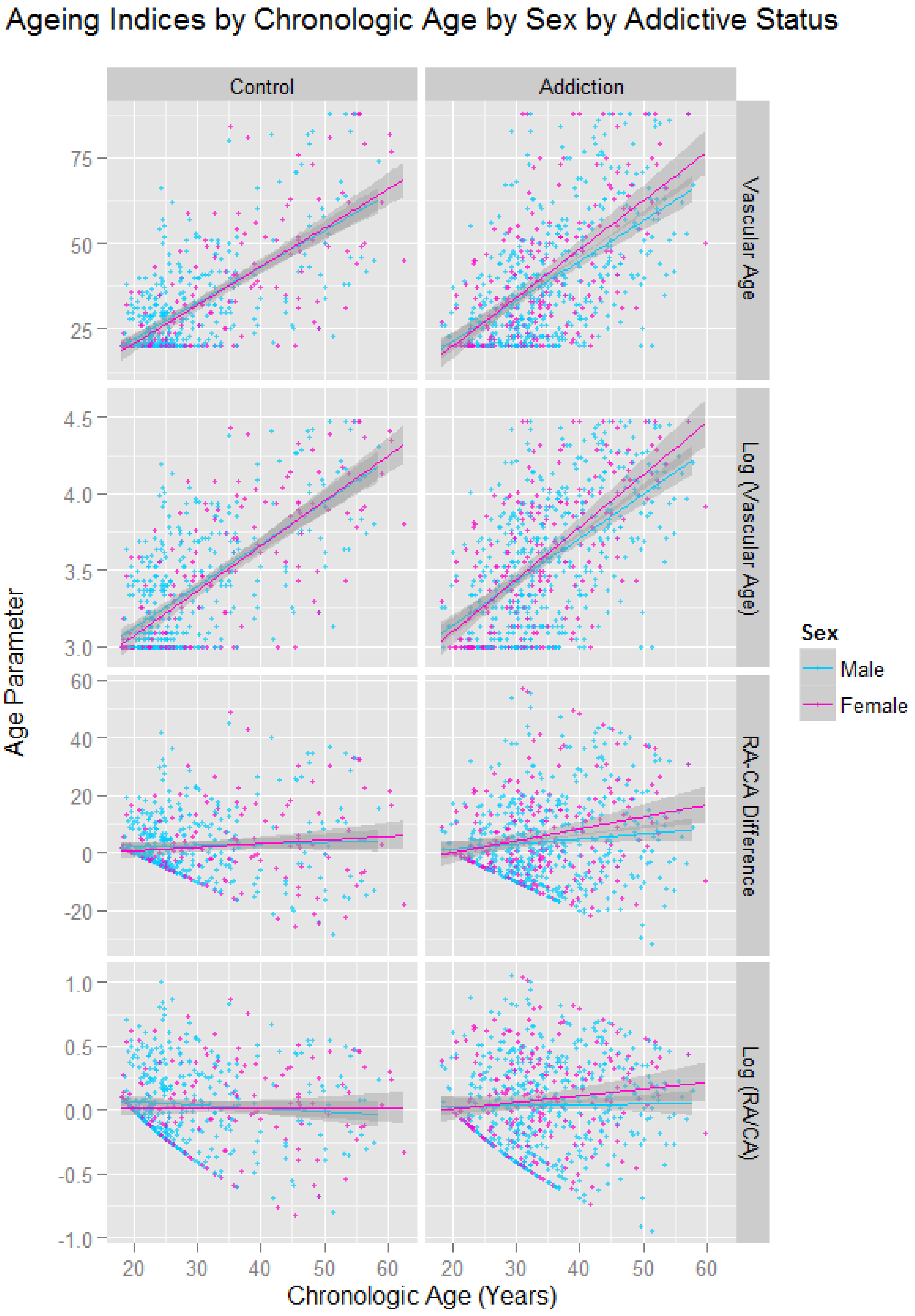

Suppl. Fig 3: Quartiles Long Logs by

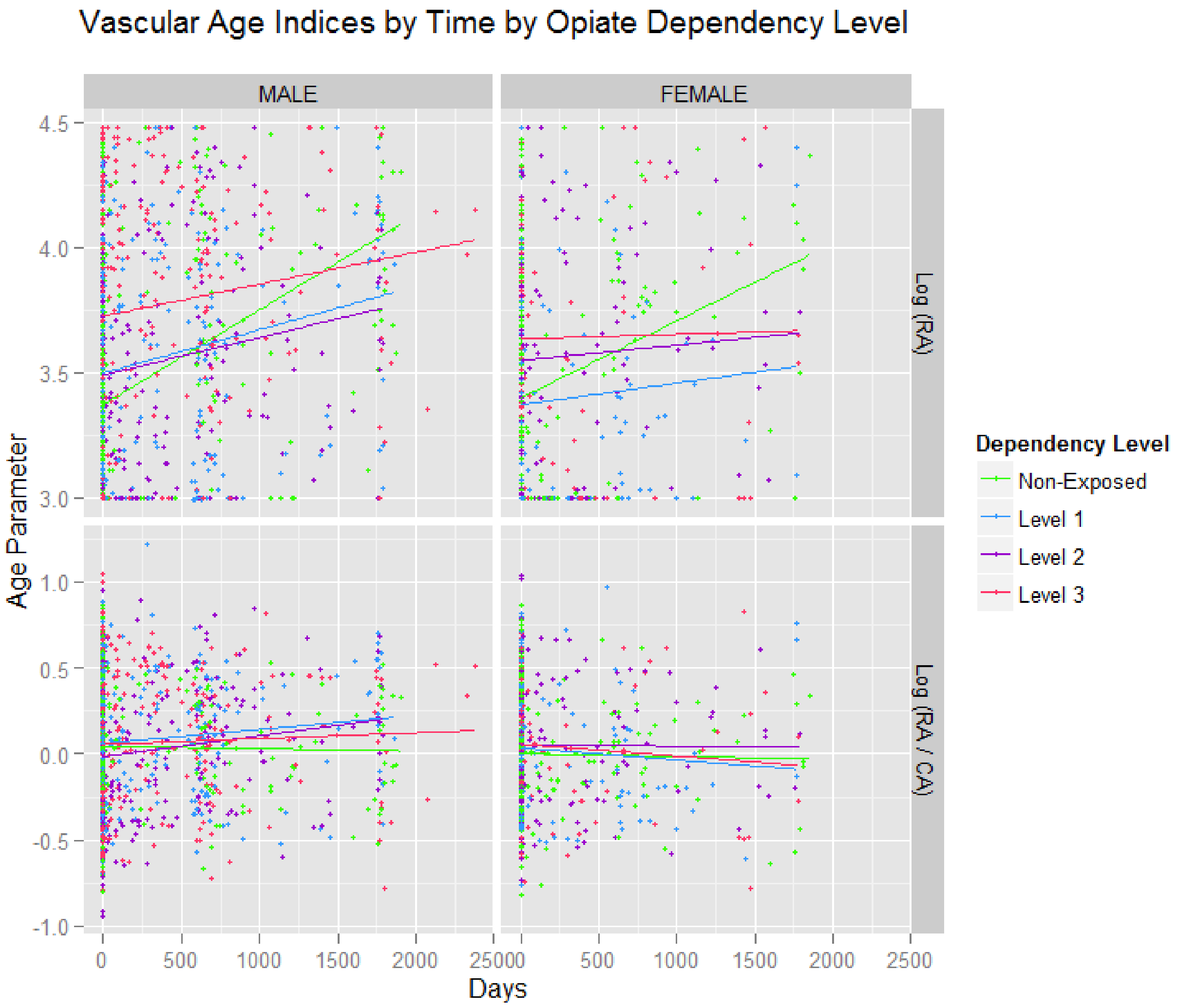

STROBE Statement—checklist of items that should be included in reports of observational studies

DEAR EDITOR,

PLEASE NOTE THAT ALKLK OF THESE HAVE BEEN MET IN OUR ACCOMPANYING ARTILCE.

THANKYOU,

DR STUART REECE.

	Item No	Recommendation
**Title and abstract**	1	(*a*) Indicate the study's design with a commonly used term in the title or the abstract
		(*b*) Provide in the abstract an informative and balanced summary of what was done and what was found
**Introduction**		
Background/rationale	2	Explain the scientific background and rationale for the investigation being reported
Objectives	3	State specific objectives, including any prespecified hypotheses
**Methods**		
Study design	4	Present key elements of study design early in the paper
Setting	5	Describe the setting, locations, and relevant dates, including periods of recruitment, exposure, follow-up, and data collection
Participants	6	(*a*) *Cohort study*—Give the eligibility criteria, and the sources and methods of selection of participants. Describe methods of follow-up<br>*Case-control study*—Give the eligibility criteria, and the sources and methods of case ascertainment and control selection. Give the rationale for the choice of cases and controls<br>*Cross-sectional study*—Give the eligibility criteria, and the sources and methods of selection of participants
		(*b*) *Cohort study*—For matched studies, give matching criteria and number of exposed and unexposed<br>*Case-control study*—For matched studies, give matching criteria and the number of controls per case
Variables	7	Clearly define all outcomes, exposures, predictors, potential confounders, and effect modifiers. Give diagnostic criteria, if applicable
Data sources/ measurement	8*	For each variable of interest, give sources of data and details of methods of assessment (measurement). Describe comparability of assessment methods if there is more than one group
Bias	9	Describe any efforts to address potential sources of bias
Study size	10	Explain how the study size was arrived at
Quantitative variables	11	Explain how quantitative variables were handled in the analyses. If applicable, describe which groupings were chosen and why
Statistical methods	12	(*a*) Describe all statistical methods, including those used to control for confounding
		(*b*) Describe any methods used to examine subgroups and interactions
		(*c*) Explain how missing data were addressed
		(*d*) *Cohort study*—If applicable, explain how loss to follow-up was addressed

For peer review only

Case-control study—If applicable, explain how matching of cases and controls was addressed

Cross-sectional study—If applicable, describe analytical methods taking account of sampling strategy

(*e*) Describe any sensitivity analyses

Continued on next page

For peer review only

**Results**		
Participants	13*	(a) Report numbers of individuals at each stage of study—eg numbers potentially eligible, examined for eligibility, confirmed eligible, included in the study, completing follow-up, and analysed
		(b) Give reasons for non-participation at each stage
		(c) Consider use of a flow diagram
Descriptive data	14*	(a) Give characteristics of study participants (eg demographic, clinical, social) and information on exposures and potential confounders
		(b) Indicate number of participants with missing data for each variable of interest
		(c) *Cohort study*—Summarise follow-up time (eg, average and total amount)
Outcome data	15*	*Cohort study*—Report numbers of outcome events or summary measures over time
		Case-control study—Report numbers in each exposure category, or summary measures of exposure
		Cross-sectional study—Report numbers of outcome events or summary measures
Main results	16	(*a*) Give unadjusted estimates and, if applicable, confounder-adjusted estimates and their precision (eg, 95% confidence interval). Make clear which confounders were adjusted for and why they were included
		(*b*) Report category boundaries when continuous variables were categorized
		(*c*) If relevant, consider translating estimates of relative risk into absolute risk for a meaningful time period
Other analyses	17	Report other analyses done—eg analyses of subgroups and interactions, and sensitivity analyses
**Discussion**		
Key results	18	Summarise key results with reference to study objectives
Limitations	19	Discuss limitations of the study, taking into account sources of potential bias or imprecision. Discuss both direction and magnitude of any potential bias
Interpretation	20	Give a cautious overall interpretation of results considering objectives, limitations, multiplicity of analyses, results from similar studies, and other relevant evidence
Generalisability	21	Discuss the generalisability (external validity) of the study results
**Other information**		
Funding	22	Give the source of funding and the role of the funders for the present study and, if applicable, for the original study on which the present article is based

*Give information separately for cases and controls in case-control studies and, if applicable, for exposed and unexposed groups in cohort and cross-sectional studies.

Note: An Explanation and Elaboration article discusses each checklist item and gives methodological background and published examples of transparent reporting. The STROBE checklist is best used in conjunction with this article (freely available on the Web sites of PLoS Medicine at http://www.plosmedicine.org/, Annals of Internal Medicine at http://www.annals.org/, and Epidemiology at http://www.epidem.com/). Information on the STROBE Initiative is available at www.strobe-statement.org.

For peer review only
